# Expression of *PmACRE1* in *Arabidopsis thaliana* enables host defence against *Bursaphelenchus xylophilus* infection

**DOI:** 10.1186/s12870-022-03929-7

**Published:** 2022-11-22

**Authors:** Wanfeng Xie, Xiaomei Xu, Wenjing Qiu, Xiaolin Lai, Mengxia Liu, Feiping Zhang

**Affiliations:** 1 Jinshan College, Fujian Agriculture and Forestry University, Fuzhou, 350002 People’s Republic of China; 2 Key Laboratory of Integrated Pest Management in Ecological Forests, Fujian Province University, Fujian Agriculture and Forestry University, Fuzhou, 350000 People’s Republic of China; 3 Forestry College, Fujian Agriculture and Forestry University, Fuzhou, 350000 People’s Republic of China

**Keywords:** Pine wilt disease, *PmACRE1*, Metabolites, Disease resistance, Protein interactions

## Abstract

**Background:**

Pine wilt disease (PWD) is a destructive disease that endangers pine trees, resulting in the wilting, with yellowing and browning of the needles, and eventually the death of the trees. Previous studies showed that the *Avr9/Cf-9* rapidly elicited (*PmACRE1*) gene was downregulated by *Bursaphelenchus xylophilus* infection, suggesting a correlation between *PmACRE1* expression and pine tolerance. Here, we used the expression of *PmACRE1* in *Arabidopsis thaliana* to evaluate the role of *PmACRE1* in the regulation of host defence against *B. xylophilus* infection.

**Results:**

Our results showed that the transformation of *PmACRE1* into *A. thaliana* enhanced plant resistance to the pine wood nematode (PWN); that is, the leaves of the transgenic line remained healthy for a longer period than those of the blank vector group. Ascorbate peroxidase (APX) activity and total phenolic acid and total flavonoid contents were higher in the transgenic line than in the control line. Widely targeted metabolomics analysis of the global secondary metabolites in the transgenic line and the vector control line showed that the contents of 30 compounds were significantly different between these two lines; specifically, the levels of crotaline, neohesperidin, nobiletin, vestitol, and 11 other compounds were significantly increased in the transgenic line. The studies also showed that the ACRE1 protein interacted with serine hydroxymethyltransferase, catalase domain-containing protein, myrosinase, dihydrolipoyl dehydrogenase, ketol-acid reductoisomerase, geranylgeranyl diphosphate reductase, S-adenosylmethionine synthase, glutamine synthetase, and others to comprehensively regulate plant resistance.

**Conclusions:**

Taken together, these results indicate that *PmACRE1* has a potential role in the regulation of plant defence against PWNs.

**Supplementary Information:**

The online version contains supplementary material available at 10.1186/s12870-022-03929-7.

## Background

Pine wilt disease (PWD) is a devastating epidemic of pine tree species [1, 2]. It initially invaded Nanjing in 1982 [3]. Since that time, the spread rate and occurrence area of the disease have continually increased, and it has been found in more than 700 cities in China to date [4, 5], posing a serious threat to forest ecology. Therefore, it is urgent to develop sustainable and effective techniques to enhance pine defences against pine wood nematodes (PWNs).

Plant disease resistance is closely related to the expression levels of resistance genes, and the expression levels of these genes are discriminative between resistant and susceptible phenotypes. Xu et al. (2013) detected 124 differentially expressed genes (DEGs) after PWN infection, which were involved in signal transduction, transcription, translation and secondary metabolism [6]. Studies have also identified differentially expressed proteins between resistant and susceptible phenotypes, showing that the expression levels of Cu–Zn superoxide dismutase, glutathione S-transferase, ascorbate peroxidase (APX), and photosynthesis-related ATP synthase were significantly higher in resistant samples than in susceptible samples [7]. Activated antioxidases might contribute to improving the resistance of Masson pine [7]. The results of high-throughput transcriptome sequencing indicated that the DEGs in susceptible phenotypes were mainly involved in stress response and terpenoid synthesis, whereas those from resistant phenotypes mainly participated in the formation of symplasm [8]. Similarly, the majority of the DEGs from *Pinus pinaster* after infection with PWN were relevant to the secondary metabolism, oxidative stress, and pathogen defensive pathways [9].

In our previous study, mRNA-Seq and miRNA-Seq techniques were applied to explore the genes involved in the regulation of *Pinus massoniana* resistance to PWN. The results revealed that 51, 75, and 44 miRNAs were differentially expressed in *P. massoniana* at 1, 2, and 3 days, respectively, after nematode inoculation [10]. Among them, 10 miRNAs were highly expressed in all three inoculated samples compared with the control group. Meanwhile, a total of 1760, 1806, and 1725 DEGs were detected in the three inoculated *P. massoniana* samples, and these genes were significantly enriched in phenylalanine metabolism, secondary metabolite biosynthesis, phenylpropanoid biosynthesis, metabolic pathways, and plant hormone signal transduction [11]. Notably, a disease resistance (R) gene encoding the *Avr9/Cf-9* rapidly elicited (*PmACRE1*) gene in *P. massoniana* was suppressed after PWN inoculation, and the transcript level of *PmACRE1* was negatively regulated by *miR946*, *novel-m0136-3* and *novel-m0051-3p* [12]. Decreasing expression levels of *PmACRE1* were observed in inoculated groups with increases in the PWN population, suggesting a potential correlation between *PmACRE1* transcript abundance and disease resistance.

R genes are regarded as the vital genes in the regulation of plant disease resistance, and the first identified R gene, *Hm1,* was found in maize in 1992 [13]. Thereafter, a number of R genes were cloned from several species, including rice, maize, tomato, *Arabidopsis,* and fruit trees, including *Xa43(t)*, *Ptr*, *Xa21*, and *Pib* from rice, *Htn1* from maize, *RPS2* from *Arabidopsis thaliana*, and *Pto* from tomato [14–21]. Although each R gene responds especially strongly to particular pathogens, most R genes from different plants have typical domains and motifs and are highly conserved in gymnosperms, angiosperms, and animals [22, 23]. It has been documented that most R genes in plants contain sequences encoding nucleotide-binding site (NBS) and a region encoding leucine-rich repeats (LRRs) at the C-terminus. Most of these R genes also have a CC (coiled-coil) domain at the N-end [24–27]. In *P. sylvestris*, the R gene *PsACRE*, which contains an LRR motif, was documented to regulate *P. sylvestris* resistance to the root rot fungus *Heterobasidion annosum* [28].

Antioxidase and antioxidants play roles in the regulation of plant resistance. For example, the antioxidase APX cleaves reactive oxygen species (ROS) to reduce oxidase damage, which is usually caused by pathogen infestation. In addition, particular flavonoids and phenolic antioxidants that belong to plant secondary metabolites also play important roles in improving stress resistance. Recently, the development of metabonomic techniques has enabled the global identification and quality of secondary metabolites in plants. The current major metabolomics methodologies include traditional targeted and nontargeted metabolomics, pseudotargeted metabolomics, and widely targeted metabolomics [29]. Among these techniques, widely targeted metabolomics integrates the advantages of the nontarget and targeted methodology, enables a high-throughput analysis, exhibits a high sensitivity and wide coverage, and allows the simultaneous detection of thousands of metabolites [30]; therefore, it has been widely applied to reveal the role of particular secondary metabolites in regulating plant tolerance [31, 32].

In this study, to validate the positive function of the *PmACRE1* gene in the regulation of plant resistance to PWN, the *PmACRE1* gene was constitutively expressed in *Arabidopsis thaliana*, and the resistance of transgenic *A. thaliana* to PWN was evaluated to explore the potential role of the gene according to the comparison of the leaf phenotype and determination of antioxidase and antioxidants. The protein encoded by the *PmACRE1* gene also contains a leucine repeat motif, which is involved in protein‒protein interactions and is a critical gene for the specific recognition of pathogen activators [33, 34]. Coimmunoprecipitation (co-IP) was then conducted to obtain proteins that interact with ACRE1, and the changes in the contents and types of secondary metabolites between *PmACRE1* transgenic *A. thaliana* and the vector control group were elucidated by using a widely targeted metabonomic technique. We aimed to clarify the role and regulatory network of *PmACRE1* in plant defence against PWN.

## Results

### *PmACRE1* gene and domains

The CDS of the *PmACRE1* gene (Accession number: MF630966) contains 624 bp nucleotides encoding 207 amino acids. The ACRE1 protein contains the **L**PV**L**QK**L**SP**L** peptide, which forms a leucine-rich repeat (LRR) structural motif (Fig. S1) and is mostly involved in the formation of protein‒protein interactions [34, 35].

### *PmACRE1* transgenic *A*. *thaliana* resistance to PWNs

**Fig. 1:**
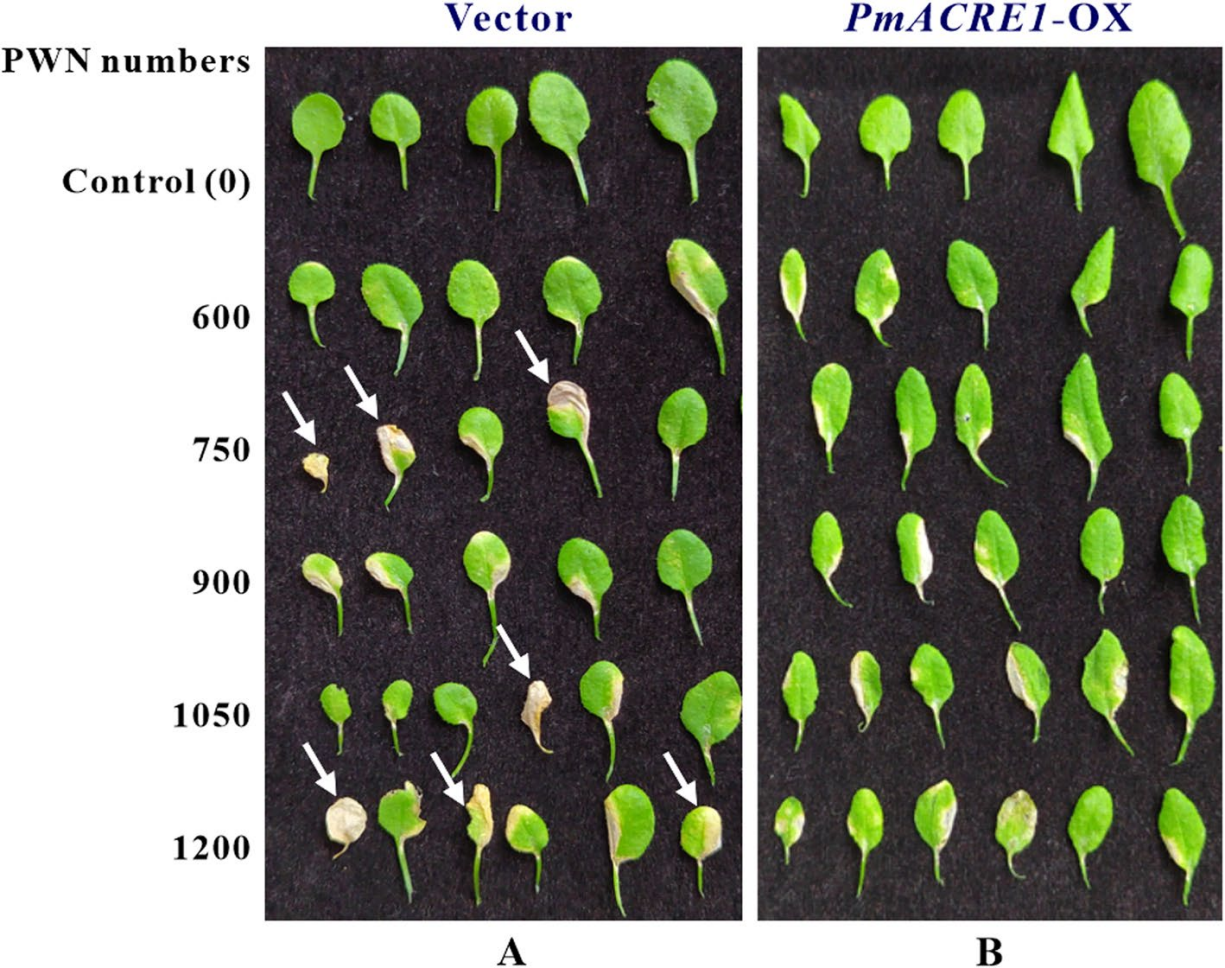
Leaves from *PmACRE1* transgenic *Arabidopsis thaliana* and the vector control line after inoculation with pine wood nematodes for 12 days. A total of 600, 750, 900, 1050, and 1200 pine wood nematodes were used to inoculate vector control **A** and *PmACRE1* transgenic *A. thaliana* **B**. White arrows indicate withered leaves after PWN inoculation

The identity of *PmACRE1* transgenic *A. thaliana* was confirmed by western blotting with a FLAG antibody (Fig. S2). These transgenic *A. thaliana* plants and their vector-control samples were subjected to PWN (*Bursaphelenchus xylophilus*) infection (Fig. S3). We found more withered and brown leaves with 37–58% of the total leaves in the vector control group compared with the transgenic line at 28–36% (Fig. 1, Fig. S4). These results indicated that constitutive expression of exogenous *PmACRE1* in *A. thaliana* increased tolerance to PWN infection.

**Fig. 2:**
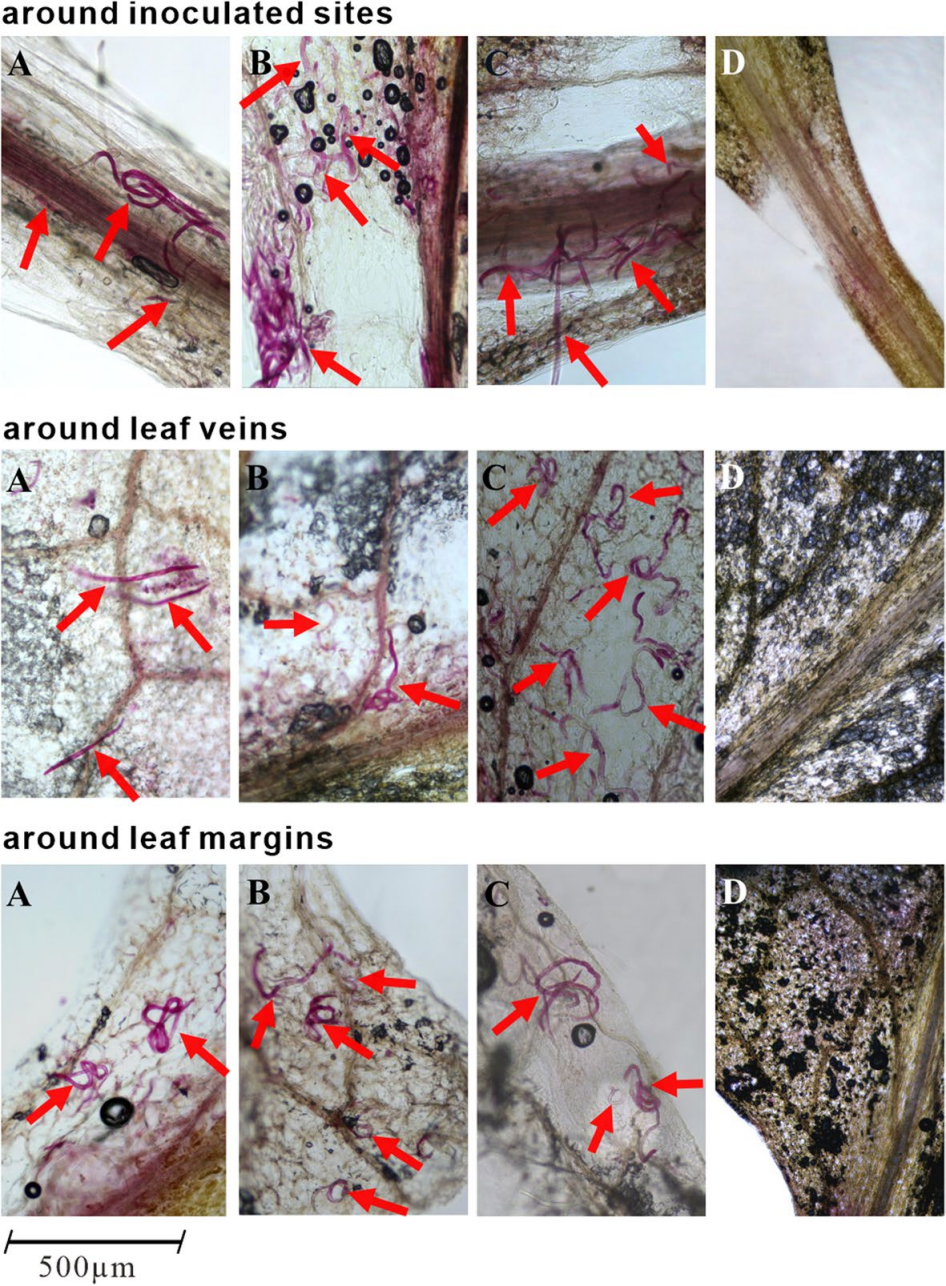
Distribution of PWN in the leaves of *A. thaliana.* PWN was found in the inoculated sites, leaf veins, and leaf margins. Red arrows indicate the PWN distribution in different parts of the leaf after artificial inoculation. **A**, **B**, and **C** show three independent leaves, and **D** shows leaves from the control group inoculated with sterilized ddH_2_O

After inoculation, the PWNs were randomly distributed around the inoculated sites, leaf veins, and leaf margins (Fig. 2), demonstrating that these PWNs were capable of colonization and survival in the leaves of *A. thaliana* and consequent production of disease symptoms.

### Phenol and flavonoid accumulation in *PmACRE1* transgenic *A*. *thaliana*

**Fig. 3:**
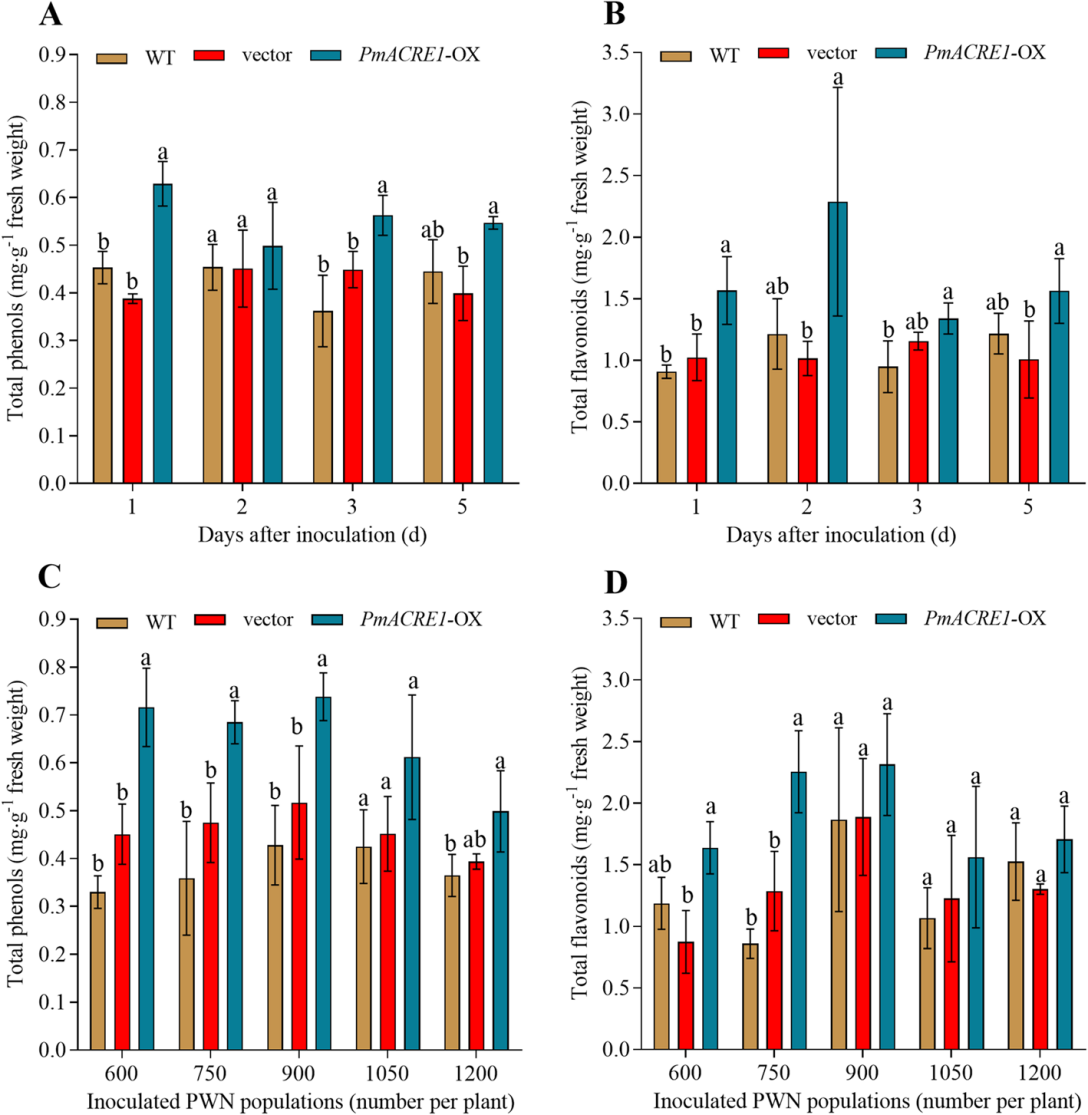
Total phenol and flavonoid contents in *PmACRE1* transgenic *A. thaliana* and the control group. The total phenol and total flavonoid contents were measured in *PmACRE1* transgenic *A. thaliana* and the control group after inoculation with 600 PWN for 1, 2, 3, and 5 days **A**, **B**. Meanwhile, these antioxidants were also measured in *PmACRE1* transgenic *A. thaliana* and the control group inoculated with 600, 750, 900, 1050, and 1200 PWNs for 5 days. All of the data were subjected to least significant difference analysis at *p* < 0.05 (LSD_0.05_). A comparison of *PmACRE1-*OX, the vector control group, and the wild type under the same treatment was conducted. For each treatment, different superscript letters on the column indicate significant differences (*p* < 0.05)

To clarify the relationship between antioxidant accumulation and plant resistance, the total phenolic acid and total flavonoid contents of the plants were determined. The phenolic acid and flavonoid contents were higher in the *PmACRE1* transgenic group than in the vector control group after inoculation with 600 PWNs for 1, 2, 3, and 5 days (Fig. 3A, B). When different amounts of PWNs were used in the inoculation, the *PmACRE1* transgenic group also contained higher contents of phenols and flavonoids than the vector control group and the wild type (Fig. 3C, D). The highest contents of phenols and flavonoids were observed in plants inoculated with 900 PWNs. The total phenol and total flavonoid accumulation in the *PmACRE1* transgenic line would promote plant resistance to PWN infection.

### APX activity in *PmACRE1* transgenic *A*. *thaliana*

**Fig. 4:**
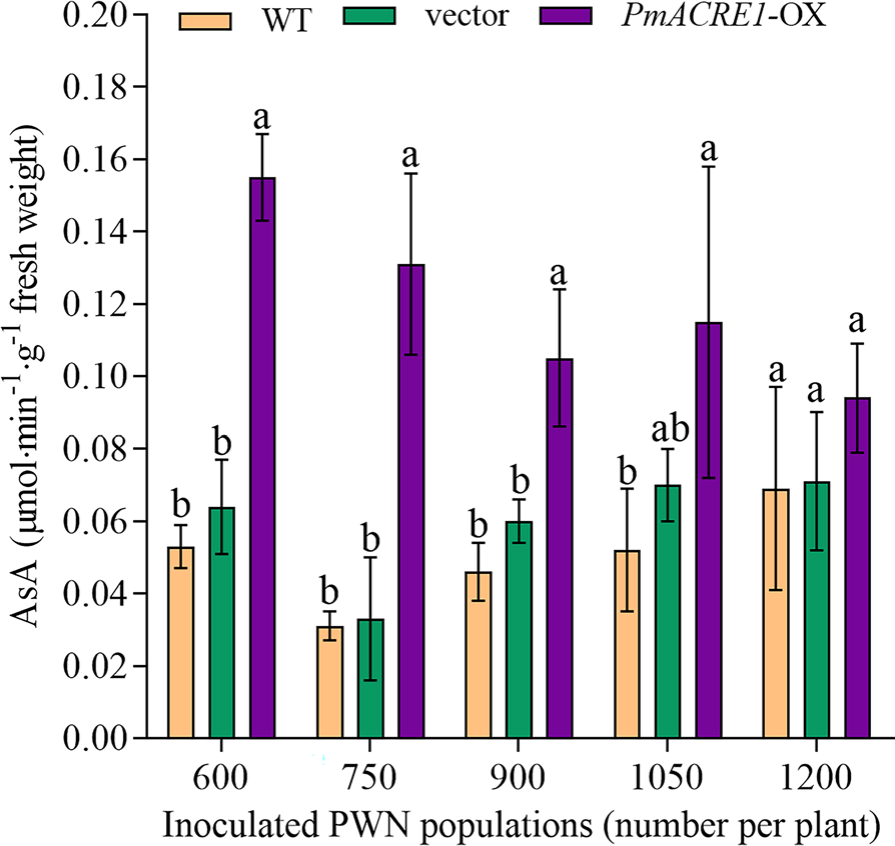
Ascorbate peroxidase activity in *PmACRE1* transgenic *A. thaliana* and controls. Ascorbate peroxidase activity was detected in *PmACRE1* transgenic *A. thaliana* and control plants inoculated with 600, 750, 900, 1050, and 1200 PWNs for 5 days. All of the data were subjected to least significant difference analysis at *p* < 0.05 (LSD_0.05_). A comparison of *PmACRE1-*OX, the vector control group, and the wild type under the same treatment was conducted. For each treatment, different superscript letters on the column indicate significant differences (*p* < 0.05)

Determination of APX activity showed that the overexpression of *PmACRE1* in *A. thaliana* enhanced the APX activity in the plant. APX activity was significantly increased in the *PmACRE1* transgenic group compared with the vector control group and the wild type after inoculation with 600, 750, 900, or 1050 PWNs (Fig. 4). Increasing APX activity in the *PmACRE1* transgenic line contributed to scavenging ROS produced from hypersensitive responses (HR) to pathogen infection.

### Metabolite differences between the *PmACRE1* transgenic *A*. *thaliana* and control groups

**Fig. 5:**
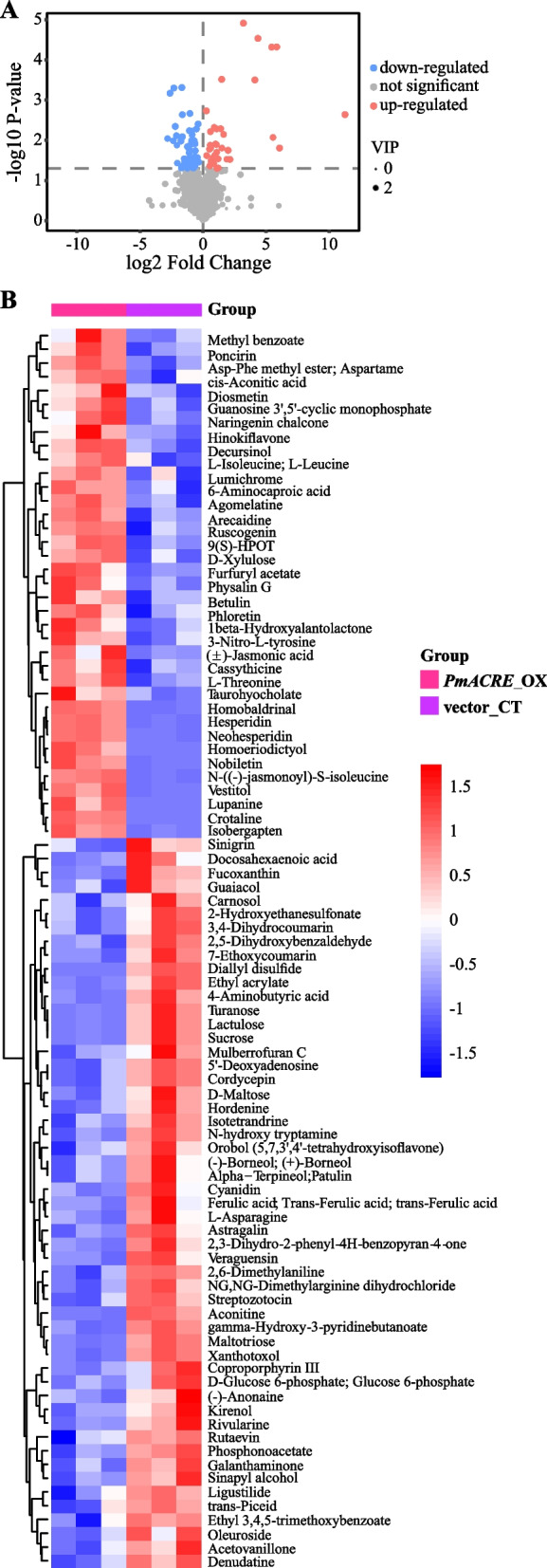
Differences in secondary metabolites between *PmACRE1* transgenic *A. thaliana* and the vector control. The differences in the types and contents of secondary metabolites between *PmACRE1* transgenic *A. thaliana* and the vector control group were visualized in volcano plots **A** and heatmaps **B**

Global secondary metabolites were analysed and compared between the transgenic line and vector group. In the widely targeted metabolomics of *PmACRE1* transgenic *A. thaliana* and the vector control leaves, a total of 1109 individual secondary metabolites were identified (Fig. 5A; Table S1). Among these compounds, 37 were upregulated in the *PmACRE1* transgenic plants, while 53 were downregulated (Fig. 5B).

**Fig. 6:**
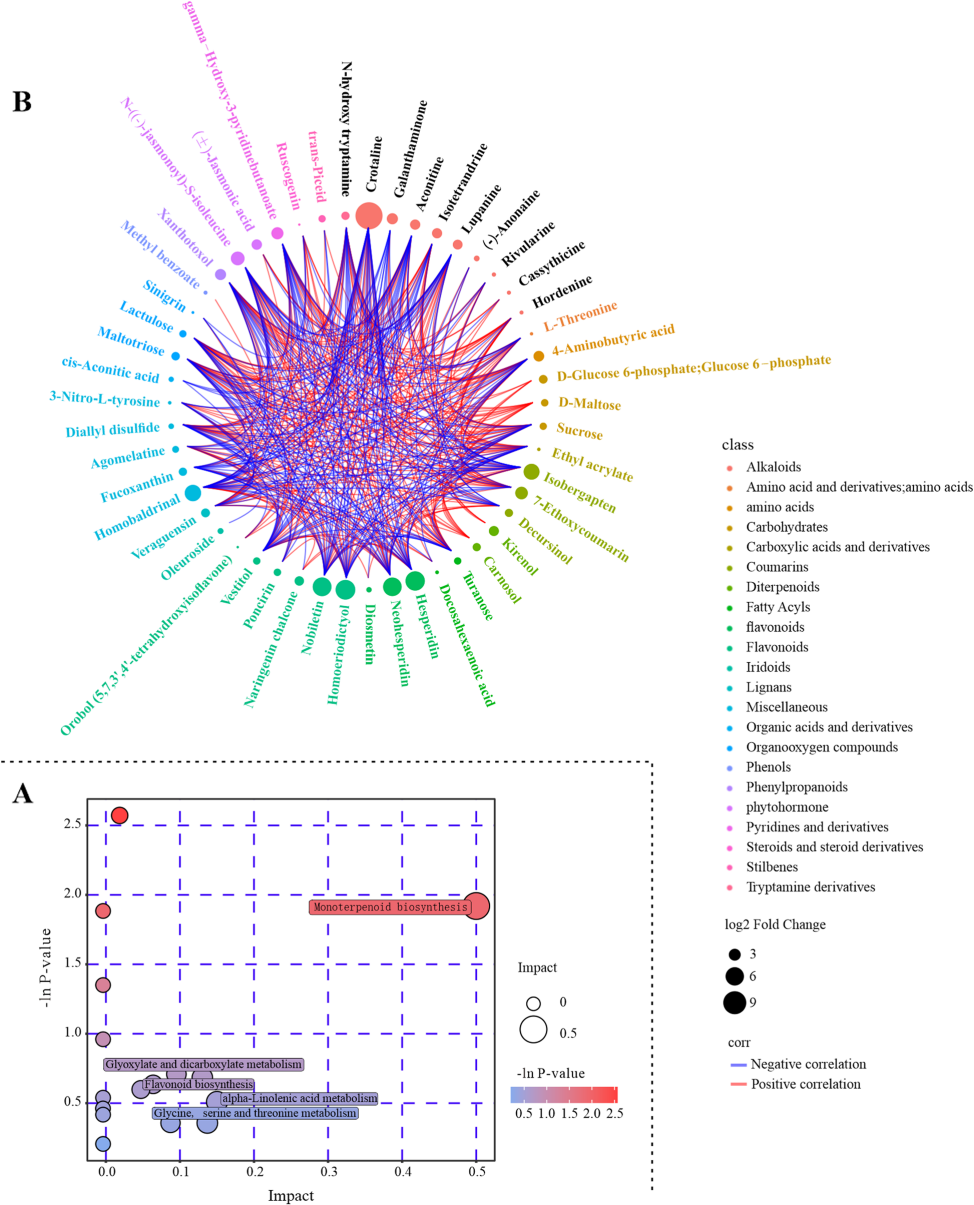
KEGG pathway enrichment and correlation analysis of the differentially expressed metabolites. Differentially expressed metabolites between *PmACRE1* transgenic *A. thaliana* and the vector control group were annotated by KEGG, and enrichment analysis of the relevant pathways was performed. Each bubble represents a metabolic pathway, and the pathways highlighted by enrichment analysis and topological analysis are labelled **A**. In the correlation analysis of the differentially expressed metabolites, red and blue connecting lines represent the positive and negative correlations between two compounds, respectively **B**

Among the 90 compounds with different contents in the *PmACRE1* transgenic *A. thaliana* and vector control groups, 15 were functionally annotated to be associated with 16 pathways, including valine, leucine and isoleucine biosynthesis, monoterpenoid biosynthesis, tyrosine metabolism, aminoacyl-tRNA biosynthesis, valine, leucine and isoleucine degradation, glyoxylate and dicarboxylate metabolism, flavonoid biosynthesis, and phenylpropanoid biosynthesis, among others (Table S2). Monoterpenoid biosynthesis and flavonoid biosynthesis, which are critical for plant defence, were regulated by *PmACRE1* (Fig. 6A). These compounds showed positive and negative correlations with each other, while carnosol, kirenol, D-maltose, D-glucose 6-phosphate, and methyl benzoate had positive correlations with other compounds (Fig. 6B).

Analysis of the differentially expressed metabolites (DEMs) indicated that the contents of crotaline, homoeriodictyol, hesperidin, nobiletin, neohesperidin, homobaldrinal, isobergapten, N-((-)-jasmonoyl)-S-isoleucine, ( ±)-jasmonic acid, lupanine, naringenin chalcone, decursinol, poncirin, and vestitol were the top 14 compounds that were significantly increased in abundance in *PmACRE1* transgenic *A. thaliana*, whereas 7-ethoxycoumarin, gamma-hydroxy-3-pyridinebutanoate, galanthaminone, xanthotoxol, 4-aminobutyric acid, aconitine, isotetrandrine, kirenol, veraguensin, D-glucose 6-phosphate (glucose 6-phosphate), maltotriose, N-hydroxy tryptamine, fucoxanthin, carnosol, and D-maltose abundances were significantly decreased in *PmACRE1* transgenic *A. thaliana*. Among the upregulated DEMs, crotaline and lupanine are alkaloids; homoeriodictyol, hesperidin, nobiletin, neohesperidin, naringenin chalcone, poncirin, and vestitol are flavonoids and isoflavonoids; and isobergapten and decursinol are coumarins. The analysis of these notably changed DEMs indicated that *PmACRE1* functions in the regulation of flavonoid synthesis and that the upregulated metabolites include a number of phytoalexins with antioxidant effects that facilitate plant defence against disease.

**Fig. 7:**
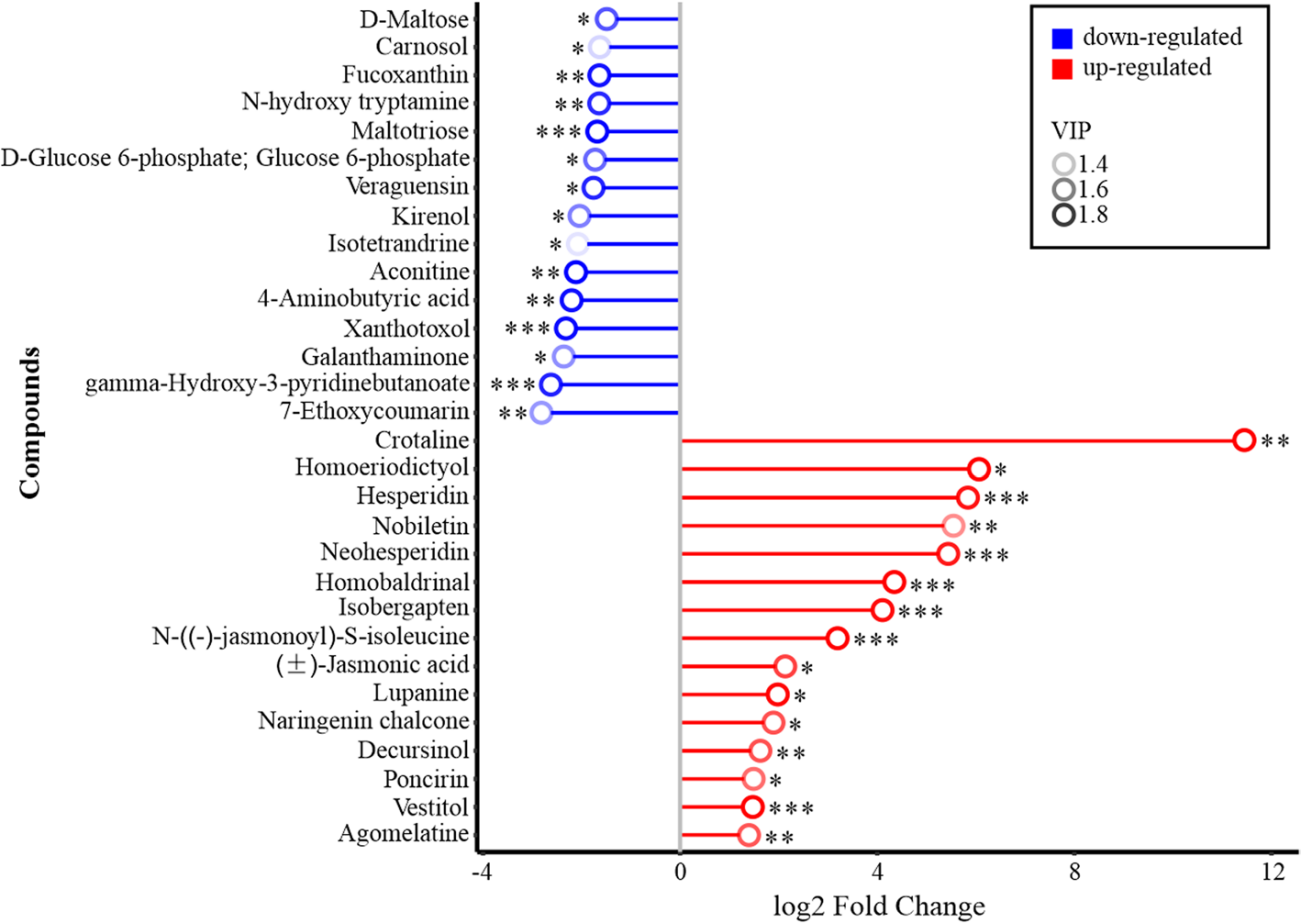
Fold changes of the top 15 compounds with differential abundance between *PmACRE1* transgenic *A.* thaliana and the vector control group. The fold change of the top 15 upregulated and downregulated compounds is illustrated in a matchstick graph. The log_2_-transformed fold change is presented on the X-axis, and the Y-axis presents the compounds. Asterisks (*) indicate a significant fold change in the *PmACRE1* transgenic *A. thaliana* and vector control groups (* 0.01 < *p* < 0.05, **0.001 < *p* < 0.01, *** *p* < 0.001)

Further analysis of the fold change of DEMs between the *PmACRE1* transgenic line and vector control line showed that crotaline in the *PmACRE1* transgenic line had the highest fold increase relative to the vector control, showing 2432.5-fold upregulation compared with the vector control. In addition, ( ±)-jasmonic acid and N-((-)-jasmonoyl)-S-isoleucine, known as endogenous signalling molecules in the activation of the plant defence response against pests, were upregulated 4.38- and 9.15-fold in the *PmACRE1* transgenic line, respectively (Fig. 7).

### Protein interaction network of ACRE1

**Fig. 8:**
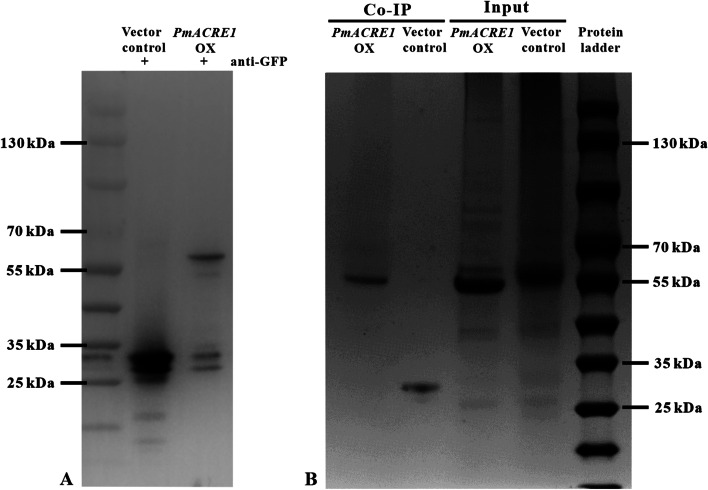
Protein interactions with ACRE1 in *Arabidopsis thaliana*. The proteins interacting with ACRE1 were isolated from T_3_ homozygous *PmACRE1*-OX transgenic lines. The vector control line was used as a control. Endogenous leaf proteins were extracted from these two lines and incubated with GFP-Trap agarose beads to identify the proteins that could interact with ACRE1. The interacting proteins that were isolated from *PmACRE1*-OX and the vector control were detected by western blotting with anti-GFP antibody **A** and Coomassie Brilliant Blue staining **B**

Further exploration of the protein interaction network of ACRE1 in *A. thaliana* showed that ACRE1 interacted with numerous proteins (Fig. 8; Table S3). The functions of these interacting proteins were enriched in the biosynthesis of secondary metabolites, carbon metabolism, metabolic pathways, and biosynthesis of amino acids, among others (Fig. S5). Among these factors, many proteins relevant to plant resistance, such as catalase, serine hydroxymethyltransferase, and geranylgeranyl diphosphate reductase, are involved in the biosynthesis of secondary metabolites and photosynthesis and ATP synthesis (Table 1). The interactions among these proteins might contribute to the regulation of disease resistance to PWN.

**Table 1:** The target proteins from *A. thaliana* interacted with ACRE1 identified by LC–MS

**Accession**	**Description**	**Coverage**	**Peptides**	**PSMs**	**Peptides Sequest HT**
A0A1B1W4U8	Ribulose bisphosphate carboxylase large chain	54.90605	26	78	26
O04771	Ribulose bisphosphate carboxylase large chain (Fragment)	54.29234	23	73	23
P19366	ATP synthase subunit beta, chloroplastic	60.04016	21	32	21
A0A1B1W4S8	ATP synthase subunit alpha, chloroplastic	43.3925	15	34	15
F4JTQ0	Vacuolar proton pump subunit B	42.71255	14	18	14
A0A654ECE2	Catalase domain-containing protein	37.09981	13	23	13
A0A654FDF6	(thale cress) hypothetical protein	39.01961	13	17	13
A0A178UYX1	Serine hydroxymethyltransferase	35.78337	13	15	13
Q8W4E2	V-type proton ATPase subunit B3	40.65708	12	16	12
A0A7G2EF98	(thale cress) hypothetical protein	7.687651	11	13	11
A0A178UU99	Tubulin beta chain	32.43243	11	15	11
A0A178UFZ5	ATP synthase subunit beta	31.84258	11	11	11
Q9LFX8	Glycine-rich protein	38.33333	10	14	10
A0A178VT47	Phosphopyruvate hydratase	38.73874	10	11	10
A0A178W9M8	Tubulin beta chain	27.39421	10	15	10
A0A178WG36	(thale cress) hypothetical protein	27.85863	10	11	10
A0A178UXP7	Tubulin alpha chain	32.44444	9	11	9
A0A178UUD0	Catalase	20.3252	7	12	7
Q9C5C2	Myrosinase 2	13.34552	6	7	6
Q9CA67	Geranylgeranyl diphosphate reductase, chloroplastic	10.92077	3	3	3
A0A178V1X0	Serine hydroxymethyltransferase	8.917197	3	3	3
Q676U0	Disease resistance protein (ACRE)	9.661836	1	1	1

## Discussion

*ACRE* genes are a group of defence genes that play predominant roles in the plant defence response. These genes were first identified in the *Avr9-* and *CF-9*-mediated defence response in tobacco suspension cells [36]. The cDNA-AFLP analysis demonstrated that 290 cDNA fragments were differentially expressed, and 13 of these cDNA clones encoded *ACRE* genes. The expression of *ACRE* genes in leaves was induced by *Avr9*, whereas other stresses induced only transient expression of the *ACRE* genes [36].

The rapid induction of *ACRE* expression enables this protein to function in the plant defence response. Ni et al. (2010) cloned a new ring-H2 finger protein gene (*StRFP1*) from potato that was found to be homologous to the *NtACRE132* gene of tobacco. Gene expression of *StRFP1* could be induced by *Phytophthora infestans*, salicylic acid, abscisic acid, and methyl jasmonate. Overexpression of *StRFP1* in potato plants increased their resistance to *P. infestans*. In contrast, transgenic potato plants with *StRFP1* expression inhibited by RNAi were more susceptible to *P. infestans* infection. These results suggest that the *StRFP1* gene, which is homologous to *NtACRE132,* plays a role in potato resistance to late blight [37].

Among conifer species, the first report of an *ACRE* gene was from *Pinus sylvestris* (*PsACRE*). The peptide encoded by *PsACRE* has high homology with the ACRE 146 protein in potato and contains leucine-rich repeat sequence (LRR) motifs. *PsACRE* can regulate *P. sylvestris* resistance to the root rot fungus *Heterobasidion annosum* [28]. There was only one amino acid difference between the ACRE protein in *P. massoniana* and the PsACRE protein, indicating that the *ACRE1* gene is highly conserved in pine. LRR motifs mediate protein‒protein interactions, and their high-affinity domains provide effective sites for protein‒protein interactions [13, 38], which is a key function for the specific recognition of pathogen elicitors [13, 33]. Smakowska-luzan et al. (2018) studied the interactions of 40,000 potential extracellular domains of cell surface receptors by highly sensitive and high-throughput interaction analysis, constructing a cell surface interaction network consisting of 567 interactions involving LRR. This work predicted and validated the functions of unidentified LRR-RK in plant growth and immunity [39].

Gene expression of *PmACRE1* in Masson pine was suppressed after infection by *B. xylophilus* [11], indicating the impacts of *PmACRE1* gene expression on PWD resistance. Heterogeneous expression of *PmACRE1* in *A. thaliana* can improve resistance against *B. xylophilus*, which validates the essential role of *PmACRE1* in the regulation of plant PWD resistance.

Elevation of the total phenol and total flavonoid levels is regarded as the direct and robust foundation of the defence response. These metabolites include several compounds that act as phytoalexins and play important roles in plant disease resistance [40]. Isoflavonoids are characterized by a shared phenyl ring structure, occur principally in legumes, and are regarded as phytoalexins [41]. The *PmACRE1* transgenic *A. thaliana* had higher total phenol and total flavonoid contents than the vector control line, regardless of the amount of PWN applied or duration of the inoculation. Phytoalexins enhance the disease resistance of *PmACRE1* transgenic *A. thaliana*, with less severe symptoms of withering on the leaves. In cotton, higher expression of flavonoid biosynthetic genes and the enrichment of flavonoids in a spontaneous mutant with red colouration resulted in significantly increased resistance to a fungal pathogen, *Verticillium dahlia* [42]. In *Malus crabapple* leaves, the of flavonoid compound contents of rust-infected symptomatic tissue were significantly increased, and flavonoid accumulation enhanced plant rust resistance [43].

Additionally, the antioxidase APX contributes to the PWD resistance of transgenic *A. thaliana*, and increasing the capacity of APX in the plant facilitates the reduction of ROS from the hypersensitive response (HR). HR is a direct reaction during host and pathogen interactions. ROS in the infected tissue usually induce programmed cell death to prevent the unrestrained spread of diseases [44]. The findings support the weaker disease symptoms in transgenic *A. thaliana* compared with the vector control plant. ACRE also interacts with several proteins to establish a protein regulation network, and the functions of these proteins were enriched in the biosynthesis of secondary metabolites. The increased total phenolic acids and total flavonoids in the *PmACRE1* transgenic line serves as direct or indirect evidence of pathway enrichment among the interacting proteins.

In particular, the levels of individual flavonoids or isoflavonoids, i.e., neohesperidin, homoeriodictyol, naringenin chalcone, nobiletin, vestitol and phloretin, were increased in the *PmACRE1* transgenic line, and neohesperidin has been reported to be related to defence against brown leaf spot disease in grapes [45]. Phloretin was documented as a phytoalexin that affects the virulence and fitness of *Pectobacterium brasiliense* in apple [46], while vestitol is a phytoalexin that is induced in the leaves of *Lotus corniculatus* in response to inoculation with *Helminthosporium turcicum* Pass [47].

Likewise, the levels of terpenoids, including 1-β-hydroxyalantolactone, and of botulin, crotaline and lupanine, known as alkaloids, were higher in the *PmACRE1* transgenic line, as were the levels of the biotic stress signal compound jasmonic acid and its derivative, N-((-)-jasmonoyl)-S-isoleucine. Monocrotaline isolated from *Crotalaria spectabilis* exhibited toxicity towards root-knot nematodes [48]. Overall, the combined bioactivity of these compounds enables the transgenic plant to exhibit increasing resistance to the PWN*.*

## Conclusion

This study showed that *PmACRE1* plays an important role in the regulation of plant resistance to *B. xylophilus*. Heterologous expression of *PmACRE1* in *A. thaliana* induced APX activity and enhanced phytoalexin contents, strengthening the disease resistance of the plant. The ACRE1-interacting proteins in *A. thaliana* are considered to be the mediating elements that regulate phytoalexin synthesis, thereby contributing to plant resistance. *PmACRE1* can be considered a candidate gene for enhancing plant resistance.

## Methods

### Plant materials

In this study, the *PmACRE1* gene was cloned from two-year-old Masson pine (*P. massoniana*) to construct a recombinant vector, and *A. thaliana* with an RNA-dependent RNA polymerase 6 gene mutation (*rdr6*) [49, 50] was genetically transformed with the *PmACRE1* recombinant vector.

### *PmACRE1* gene cloning and construction of a constitutive expression vector

An RNAprep Pure Plant Kit (TIANGEN Biotech (Beijing) Co., Ltd.) was used for the extraction of total RNA from the stems of *P. massoniana*. One microgram of total RNA was reverse transcribed into cDNA by using TransScript One-Step gDNA Removal and cDNA Synthesis SuperMix (TransGen Biotech Co., Ltd.). The coding sequence (CDS) of the *PmACRE1* gene was amplified from cDNA by using KOD-Plus-Neo (TOYOBO (Shanghai) Biotech Co., Ltd.) and the primers (F: 5'-*TCCAGCTCCA*GGATCCATGGAGGTCCATTCTACTGTAAATC-3'; R: 5'-GAG AAAGCTTGGATCCTTAGGCTGTACCCCTTTCAAGCA-3'; the underlined sequences represent *BamH* I sites) and fused with GFP in pCambia3301 to construct the recombinant p△*ACT2*::*GFP*-*PmACRE1* vector for genetic transformation of *A. thaliana*.

### *A*. *thaliana* transformation

The recombinant p△*ACT2*::*GFP*-*PmACRE1* vector was transformed into *Agrobacterium tumefaciens* AGL0. Genetic transformation of the *PmACRE1* gene into *A. thaliana* was performed following the floral dip protocols described by Clough and Bent [51]. Seeds were harvested from transformed *A. thaliana* and screened for positives to establish the T_0_ transgenic generation. These germinated seeds were grown in 0.075‰ Basta (glufosinate)-containing peat substrate (PINDSTRUP, Denmark). Leaves of the surviving T_0_ transgenic *A. thaliana* were ground into powder with liquid nitrogen, and then the soluble protein was extracted by using a non-grinding protein extraction buffer (0.1 M EDTA, pH 8.0; 0.12 M Tris·HCl, pH 6.8; 4% SDS; 10% β-mercaptoethanol; 5% glycerol; 0.005% bromophenol blue; and a final pH of 7.4). Genomic DNA was extracted from the leaves according to the CTAB method [52, 53].

### Identification of transgenic *A*. *thaliana*

Primers (F: 5'-ATGGAGGTCCATTCTACTGTAAATC-3'; R: 5'-TTAGGCTGTAC CCCTTTCAAGCA-3') were used to amplify the *PmACRE1* gene from genomic DNA, and *A. thaliana* genomic DNA with *PmACRE1* gene insertion was regarded to validate a positive transgenic line. An antibody against FLAG was used for western blotting to determine the expression of the ACRE1 protein, which was fused with FLAG tags and GFP. These transgenic lines of *A. thaliana* were then maintained for seed harvesting, and transgenic plants were continually identified at each generation to obtain homozygotes. All these seeds are preserved in the Key Laboratory of Integrated Pest Management in Ecological Forests, Fujian Agriculture and Forestry University.

### Inoculation of *A*. *thaliana* with PWN

A 10-μl droplet of a sterile nematode suspension containing 200, 250, 300, 350 or 400 nematodes was applied separately to 21- to 25-day-old *PmACRE1* transgenic *A. thaliana* and vector control plants with six fully open true leaves, following the protocol described by Zhao et al. [54]. The control group was treated in the same manner except that sterile distilled water was used. A total of three leaves of each plant were inoculated, and six replicate pots per treatment with 9 seedlings per pot were used. Therefore, a total of 600, 750, 900, 1050 or 1200 nematodes were applied separately on each plant. Changes in the leaf phenotypes of all *A*. *thaliana* plants were recorded to evaluate the function of *PmACRE1* in the regulation of disease resistance.

### Antioxidase and antioxidant measurement

To compare the physiological characteristics of the *PmACRE1* transgenic *A*. *thaliana* group and the control group, the activities of APX and phenylalanine ammonia-lyase (PAL) from these two groups were determined by using the relevant detection kits (Item No. G0203W used for APX, G0208W used for GST, and G0114W used for PAL; Suzhou Grace Biotechnology Co., Ltd.). The total flavonoid and total phenol contents were also detected by using a detection kit following the manufacturer’s instructions (Item No. G0117W used for total phenols and G0118W used for flavonoids; Suzhou Grace Biotechnology Co., Ltd.).

### Capture of the proteins interacting with ACRE1 in *A*. *thaliana*

Natural leaf proteins were extracted from a T_3_ generation homozygote of *PmACRE1* transgenic *A. thaliana* and incubated with GFP-Trap agarose (ChromoTek) to collect putative interacting proteins [50]. The proteins were separated by SDS polyacrylamide gel electrophoresis (SDS‒PAGE), and the proteins on the gel were sampled and enzymolysed by trypsin. The hydrolysed peptides were identified by LC‒MS/MS (Orbitrap Fusion Lumos, Thermo Fisher Scientific) and analysed on Proteome Discoverer software and a protein database from UniProt (https://www.uniprot.org/).

### Extraction of metabolites from *A*. *thaliana* leaves

The leaves of *PmACRE1* transgenic *A. thaliana* and vector control line were frozen in liquid nitrogen and crushed with a mixer mill, and 50 mg of the powder was taken for metabolite extraction using 700 μl of extract solution (methanol/water = 3:1, precooled at -40 °C, containing 2-chloro-DL-phenylalanine as internal standard). The mixture was vortexed for 30 s, homogenized at 35 Hz for 4 min, and then sonicated in an ice water bath for 5 min, and both homogenization and sonication were repeated 3 times. Then, the samples were extracted overnight at 4 ℃ 60 rpm on a shaker (Mode: MX-RD-E, DLAB Scientific Co., Ltd. Beijing, China). The mixture was centrifuged at 12,000 rpm (RCF = 13,800 (× g), R = 8.6 cm) at 4 ℃ for 15 min, and the supernatant was separated and then filtered through a 0.22 μm microporous membrane. The filtered supernatant was kept at -80 ℃ until UHPLC‒MS analysis. A quality control (QC) sample was prepared by mixing 60 μl of supernatant from the transgenic line and the control group.

### UHPLC‒MS identification of the metabolites

UHPLC separation was conducted by using an ExionLC System (Sciex). Mobile phase A contained 0.1% formic acid in water, and mobile phase B contained acetonitrile; the gradient elution program is shown in Table S1. The column temperature was set at 40 ℃. The autosampler temperature was set at 4 ℃, and the injection volume was 2 μL. A Sciex QTRAP 6500^+^ (Sciex Technologies) was applied for assay development. The MS analysis was performed in both positive ionization and negative ionization modes to obtain higher metabolite coverage. Typical ion source parameters were as follows: ion spray voltage: + 5500/-4500 V, curtain gas: 35 psi, temperature: 400 ℃, ion source gas 1:60 psi, ion source gas 2: 60 psi, and DP: ± 100 V.

SCIEX Analyst Work Station software (Version 1.6.3) was employed for MRM data acquisition and processing. Raw MS data (.wiff files) were converted to.txt format using an MS converter. An in-house R program and database were applied for peak detection and annotation. Compound peaks were detected and captured after relative standard deviation denoising. Then, missing values were filled as half of the minimum value. Accurate mass matching (< 25 ppm) and secondary spectrum matching methods were used to search the KEGG database and HMDB for metabolite structure identification.

Supervised orthogonal projections to latent structures discriminant analysis (OPLS-DA) was applied to visualize group separation and find significantly changed metabolites. Furthermore, the variable importance in the projection (VIP) value of the first principal component in OPLS-DA was obtained. VIP summarizes the contribution of each variable to the model. The peak area ratio (peak area metabolite/peak area internal standard) was calculated for each metabolite peak associated with each internal standard, and the relative standard deviation (RSD) was also calculated for each of these peaks. The internal standard providing the lowest RSD was chosen as the internal standard for that metabolite. Metabolites with VIP > 1 and *p* < 0.05 (Student’s t test) were considered significantly changed metabolites. In addition, commercial databases including KEGG (http://www.genome.jp/kegg/) and MetaboAnalyst (http://www.metaboanalyst.ca/) were used for pathway enrichment analysis.

### Statistical analysis

All of the experiments were conducted at least three times. The results in the figures are presented as the means ± SD. Statistical analyses were based on the least significant difference (LSD) test (*p* < 0.05).

All were carried out in accordance with relevant guidelines.

## Supplementary Information

**Additional file 1:** **Table S1.** The elution gradient used for UHPLC separation. **Table S1.** Total secondary metabolites identified from the leaves of *PmACRE1* transgenic plants and the vector control line. **Table S2.** KEGG pathway enrichment of the differentially expressed metabolites. **Table S3.** Differentially expressed metabolites between *PmACRE1*-OX and the vector control line. **Fig. S1.** The coding sequence of *PmACRE1. ***Fig. S2.** PmACRE1 protein expression in the transgenic *Arabidopsis thaliana. ***Fig. S3.** Inoculation of PWN on the *Arabidopsis thaliana*. **Fig. S4.** The incidence of *Arabidopsis thaliana* inoculated with different population of pine wood nematodes. **Fig. S5.** KEGG enrichment of the interacted proteins of ACRE1.

## Data Availability

All data generated during this study are included in this published article and its supplementary information files, and the raw data used or analysed during the current study available from the corresponding author on reasonable request.

## Abbreviations

APX: Ascorbate peroxidase
*ACRE1*: *Avr9/Cf-9* rapidly elicited gene
LRRs: Leucine-rich repeats
DEMs: Differentially expressed metabolites
PWN: Pine wood nematode
PWD: Pine wilt disease
ROS: Reactive oxygen species
RSD: Relative standard deviation
VIP: Variable importance in the projection
