# Supplementary material for: Expression of PmACRE1 in Arabidopsis thaliana enables host defence against Bursaphelenchus xylophilus infection
Source: BMC Plant Biol. 2022 Nov 22;22:541. doi: 10.1186/s12870-022-03929-7 (PMC9682698; doi:10.1186/s12870-022-03929-7)
Supplement: Supplementary file 1 — Additional file 1: Table S1. The elution gradient used for UHPLC separation. Table S1. Total secondary metabolites identified from the leaves of PmACRE1 transgenic plants and the vector control line. Table S2. KEGG pathway enrichment of the differentially expressed metabolites. Table S3. Differentially expressed metabolites between PmACRE1-OX and the vector control line. Fig. S1. The coding sequence of PmACRE1. Fig. S2. PmACRE1 protein expression in the transgenic Arabidopsis thaliana. Fig. S3. Inoculation of PWN on the Arabidopsis thaliana. Fig. S4. The incidence of Arabidopsis thaliana inoculated with different population of pine wood nematodes. Fig. S5. KEGG enrichment of the interacted proteins of ACRE1. [file 12870_2022_3929_MOESM1_ESM.docx]

**Supplementary information**

Table S1 The elution gradient used for UHPLC separation

| **Time**  **(min)** | **Flow rate**  **(μL/min)** | **Solvents A（%）**  **Solvents A: 0.1% formic acid solution** | **Solvents B（%）**  **Solvents B: acetonitrile** |
| --- | --- | --- | --- |
| **0** | 400 | 98 | 2 |
| **0.5** | 400 | 98 | 2 |
| **10** | 400 | 50 | 50 |
| **11** | 400 | 5 | 95 |
| **13** | 400 | 5 | 95 |
| **13.1** | 400 | 98 | 2 |
| **15** | 400 | 98 | 2 |

Table S1 Total secondary metabolites identified from the leaves of *PmACRE1* transgenic plants and the vector control line

| **id** | **compound name** | **CAS** | **KEGG_ID** | **FORMULA** | **EXACT_MASS** | **CLASS_EN** | **Q1** | **Q3** | **ionmode** | **rt** | **PmACRE_OX_1** | **PmACRE_OX_2** | **PmACRE_OX_3** | **Mean PmACRE_OX** | **vector_CT_1** | **vector_CT_2** | **vector_CT_3** | **Mean vector_CT** | **qc-01** | **qc-02** | **Mean QC** |
| --- | --- | --- | --- | --- | --- | --- | --- | --- | --- | --- | --- | --- | --- | --- | --- | --- | --- | --- | --- | --- | --- |
| 1 | (-)-Anonaine | 1862-41-5 | C09339 | C17H15NO2 | 265.11027899999999 | Alkaloids | 266.1 | 192.1 | + | 7.4233 | 3.9466E-05 | 2.83228E-05 | 4.76465E-05 | 3.84784E-05 | 7.6619E-05 | 0.000127077 | 6.988E-05 | 9.11919E-05 | 4.09589E-05 | 4.63672E-05 | 4.36631E-05 |
| 2 | (-)-Borneol;(+)-Borneol | 464-45-9;464-43-7 | C01766;C01765 | C10H18O | 154.13576499999999 | Monoterpenoids | 155.1 | 137.1 | + | 3.423966667 | 0.002238177 | 0.001911058 | 0.001497217 | 0.001882151 | 0.003953845 | 0.002546299 | 0.003130276 | 0.00321014 | 0.002660543 | 0.002607674 | 0.002634109 |
| 3 | (-)-Carvone | 6485-40-1 | C01767 | C10H14O | 150.104465 | Monoterpenoids | 151.1 | 53 | + | 10.86156667 | 0.000125858 | 7.50936E-05 | 0.000109486 | 0.000103479 | 9.28091E-05 | 6.30126E-05 | 6.75186E-05 | 7.44468E-05 | 0.000214197 | 0.000162533 | 0.000188365 |
| 4 | (-)-Catechin gallate(CG) | 130405-40-2 |  | C22H18O10 | 442.09 | Flavonoids | 443.1 | 123 | + | 5.974966667 | 0.000264823 | 5.44708E-05 | 4.5284E-05 | 0.000121526 | 3.16427E-05 | 8.85594E-05 | 6.37649E-05 | 6.13223E-05 | 0.000154948 | 0.000151723 | 0.000153335 |
| 5 | (-)-Cinchonidine | 485-71-2 | C11379 | C19H22N2O | 294.17321299999998 | Alkaloids | 295.2 | 81.1 | + | 11.99408333 | 0.009256505 | 0.004594482 | 0.003570083 | 0.005807023 | 0.006434825 | 0.005030398 | 0.004180358 | 0.005215194 | 0.006491518 | 0.006649852 | 0.006570685 |
| 6 | (-)-Epicatechin gallate | 1257-08-5 |  | C22H18O10 | 442.08999699999998 | flavonoids | 440.9 | 288.9 | - | 6.05 | 0.000294786 | 1.27649E-06 | 1.12648E-06 | 9.9063E-05 | 1.59752E-06 | 4.07862E-05 | 1.23455E-06 | 1.45394E-05 | 6.53484E-05 | 0.000245319 | 0.000155334 |
| 7 | (-)-Epigallocatechin | 970-74-1 | C12136 | C15H14O7 | 306.07400000000001 | flavonoids | 306.9 | 180.9 | + | 4.12 | 2.0493E-05 | 1.65298E-05 | 2.92796E-05 | 2.21008E-05 | 1.90109E-05 | 4.82118E-05 | 1.73847E-05 | 2.82025E-05 | 8.71197E-05 | 7.34341E-05 | 8.02769E-05 |
| 8 | (-)-Maackiain | 2035-15-6 | C10502 | C16H12O5 | 284.06847499999998 | Flavonoids | 285.1 | 95 | + | 8.475616667 | 7.90229E-05 | 0.000371618 | 0.000178878 | 0.00020984 | 0.000191684 | 0.000310513 | 0.000168411 | 0.000223536 | 0.000287301 | 0.000325233 | 0.000306267 |
| 9 | (-)-Salsoline | 89-31-6 | C09640 | C11H15NO2 | 193.11027899999999 | Tetrahydroisoquinolines | 194.1 | 117.1 | + | 4.251083333 | 8.94409E-05 | 4.34795E-05 | 7.40069E-05 | 6.89758E-05 | 0.000217513 | 7.10547E-05 | 8.65958E-05 | 0.000125055 | 4.04407E-05 | 8.76394E-05 | 6.40401E-05 |
| 10 | (-)-Sativan | 41743-86-6 | C10526 | C17H18O4 | 286.12051000000002 | Flavonoids | 287.1 | 123 | + | 7.888216667 | 0.000159727 | 0.000217462 | 0.000143938 | 0.000173709 | 0.000412059 | 0.00039314 | 0.000168099 | 0.000324432 | 0.000129495 | 0.000272173 | 0.000200834 |
| 11 | (+)-Abscisic acid | 21293-29-8 | C06082 | C15H20O4 | 264.13619999999997 | phytohormone | 263 | 153 | - | 7.86 | 0.000543937 | 0.000637337 | 0.000604313 | 0.000595196 | 0.000962685 | 0.000775942 | 0.000677974 | 0.000805534 | 0.000810864 | 0.000671547 | 0.000741205 |
| 12 | (+)-Bicuculline | 485-49-4;485-50-7 | C09372;C09364 | C20H17NO6 | 367.10558900000001 | Alkaloids | 368.1 | 307.1 | + | 5.8759 | 0.000119253 | 0.00021657 | 3.60829E-05 | 0.000123969 | 0.000177165 | 8.70819E-05 | 0.000152208 | 0.000138818 | 0.000204089 | 8.04076E-05 | 0.000142248 |
| 13 | (+)-Carvone;5-Isopropyl-2-methylphenol;Thymol | 2244-16-8;499-75-2;89-83-8 | C11383;C09840;C09908 | C10H14O | 150.104465 | Monoterpenoids;Phenols | 151.1 | 109.1 | + | 10.86149444 | 7.60188E-05 | 0.000255444 | 0.000150549 | 0.000160671 | 0.000129052 | 0.00027279 | 0.000174482 | 0.000192108 | 9.17847E-05 | 0.000245159 | 0.000168472 |
| 14 | (+)-Glaucarubinone | 1259-86-5 | C08763 | C25H34O10 | 494.21519999999998 | Diterpenoids | 495.2 | 477.2 | + | 5.79035 | 0.000113423 | 0.000264058 | 0.000339742 | 0.000239074 | 0.000306878 | 0.000214198 | 0.000219693 | 0.000246923 | 0.000237131 | 0.000256791 | 0.000246961 |
| 15 | (+)-Lyoniresinol 9'-O-glucoside | 87585-32-8 |  | C28H38O13 | 582.23124499999994 | Lignans | 583.2 | 403.2 | + | 12.79633333 | 0.007887581 | 0.008856435 | 0.006200077 | 0.007648031 | 0.012075887 | 0.003161178 | 0.006271163 | 0.007169409 | 0.009707045 | 0.007359403 | 0.008533224 |
| 16 | (+)-Pteryxin | 17944-23-9;13161-75-6 | C09307 | C21H22O7 | 386.13655499999999 | Coumarins | 387.1 | 55.1 | + | 11.10641667 | 0.001027515 | 0.001337576 | 0.001088905 | 0.001151332 | 0.001700901 | 0.001357574 | 0.000611388 | 0.001223288 | 0.000881278 | 0.001188508 | 0.001034893 |
| 17 | (±)-Jasmonic acid | 77026-92-7 | C08491 | C12H18O3 | 210.12559999999999 | phytohormone | 209 | 59 | - | 8.94 | 0.004845307 | 0.011526187 | 0.009758181 | 0.008709892 | 0.002481239 | 0.002271933 | 0.001205737 | 0.001986303 | 0.003970919 | 0.00448099 | 0.004225955 |
| 18 | (10E,12Z)-(9S)-9-Hydroperoxyoctadeca-10,12-dienoic acid | 29774-12-7 | C14827 | C18H32O4 | 312.23005999999998 | Fatty Acyls | 295.2 | 55.1 | + | 11.95935 | 0.00223318 | 0.00137761 | 0.00164467 | 0.00175182 | 0.001534438 | 0.00125306 | 0.001317043 | 0.001368181 | 0.001661886 | 0.001460414 | 0.00156115 |
| 19 | (13E)-11a-Hydroxy-9,15-dioxoprost-13-enoic acid | 22973-19-9 | C04654 | C20H32O5 | 352.22497499999997 | Fatty Acyls | 353.2 | 335.2 | + | 11.43616667 | 0.003017768 | 0.002884845 | 0.003820365 | 0.003240993 | 0.003923838 | 0.002515449 | 0.002586338 | 0.003008542 | 0.00343669 | 0.004567616 | 0.004002153 |
| 20 | (23S)-23,25-dihdroxy-24-oxovitamine D3 23-(beta-glucuronide) | | C03033 | C33H50O10 | 606.34040000000005 | Organooxygen compounds | 607.3 | 413.3 | + | 11.79861667 | 0.000172054 | 5.07466E-05 | 0.001066296 | 0.000429699 | 0.001900192 | 0.002239643 | 0.000934684 | 0.001691506 | 0.001568612 | 0.000621388 | 0.001095 |
| 21 | (2S,3R,4E)-2-Amino-4-heptadecene-1,3-diol | 6918-48-5 |  | C17H35NO2 | 285.26677899999999 | Alkaloids | 286.3 | 268.3 | + | 11.14146667 | 0.000905642 | 0.00130444 | 0.000905156 | 0.001038413 | 0.001516506 | 0.000867126 | 0.001250187 | 0.001211273 | 0.001149483 | 0.001051535 | 0.001100509 |
| 22 | (E)-3-Hydroxy-5-methoxystilbene | 35302-70-6 | C10276 | C15H14O2 | 226.09938 | Phenols | 227.1 | 91.1 | + | 8.744133333 | 9.24938E-05 | 0.000400392 | 0.000484126 | 0.00032567 | 0.00015483 | 0.000143085 | 0.00022278 | 0.000173565 | 0.000232417 | 0.000241199 | 0.000236808 |
| 23 | (R)-2-Hydroxy-2H-1,4-benzoxazin-3(4H)-one | | C15769 | C8H7NO3 | 165.04259400000001 | Benzoxazines | 166 | 108 | + | 5.0519 | 0.000116217 | 0.000172836 | 9.74391E-05 | 0.000128831 | 0.000390992 | 0.000137439 | 0.000307849 | 0.00027876 | 0.000117722 | 9.2588E-05 | 0.000105155 |
| 24 | (R)-Acetoin | 513-86-0;53584-56-8 | C00810;C00466 | C4H8O2 | 88.052430000000001 | Miscellaneous | 89.1 | 71 | + | 0.5281 | 0.001604413 | 0.001815388 | 0.002125858 | 0.001848553 | 0.001363916 | 0.001717372 | 0.000784356 | 0.001288548 | 0.001645544 | 0.001459614 | 0.001552579 |
| 25 | (R)-Menthofuran | 494-90-6 | C09868 | C10H14O | 150.104465 | Prenol lipids | 151.1 | 55.1 | + | 5.14695 | 0.001031701 | 0.00090192 | 0.000887404 | 0.000940342 | 0.001169108 | 0.00102017 | 0.001050762 | 0.001080013 | 0.000611879 | 0.0007024 | 0.000657139 |
| 26 | (S)-10-Hydroxycamptothecin | 19685-09-7 | C17939 | C20H16N2O5 | 364.10592300000002 | Alkaloids | 365.1 | 321.1 | + | 8.038433333 | 3.75188E-05 | 6.31964E-05 | 5.28443E-05 | 5.11865E-05 | 4.54162E-05 | 2.40738E-05 | 6.17853E-05 | 4.37584E-05 | 7.55782E-05 | 6.67923E-05 | 7.11853E-05 |
| 27 | (S)-2-Acetolactate |  | C06010 | C5H8O4 | 132.04226 | Keto acids and derivatives | 133 | 87 | + | 1.319083333 | 0.001160391 | 0.001450212 | 0.000467442 | 0.001026015 | 0.002294827 | 0.000798524 | 0.000421437 | 0.001171596 | 0.000576208 | 0.001553432 | 0.00106482 |
| 28 | (S)-N-Methylcoclaurine | 3423-07-2 | C05176 | C18H21NO3 | 299.15214400000002 | Isoquinolines and derivatives | 300.2 | 107 | + | 7.586116667 | 0.000145133 | 0.000120466 | 5.13184E-05 | 0.000105639 | 0.00011169 | 0.000112159 | 8.93128E-05 | 0.000104387 | 6.91292E-05 | 5.15447E-05 | 6.0337E-05 |
| 30 | 1-(alpha-Methyl-4-(2-methylpropyl)benzeneacetate)-beta-D-Glucopyranuronic acid | 115075-59-7 | C03033 | C19H26O8 | 382.16277000000002 | Organooxygen compounds | 383.2 | 189.1 | + | 12.18463333 | 0.00577729 | 0.004898031 | 0.004226069 | 0.00496713 | 0.004282447 | 0.003922951 | 0.003717817 | 0.003974405 | 0.004291803 | 0.002597825 | 0.003444814 |
| 31 | 1-(beta-D-Ribofuranosyl)-1,4-dihydronicotinamide | | C15497 | C11H16N2O5 | 256.10592300000002 | Organooxygen compounds | 257.1 | 108 | + | 2.803 | 8.33553E-05 | 7.56225E-05 | 5.65616E-05 | 7.18465E-05 | 9.92969E-05 | 0.000139851 | 8.86058E-05 | 0.000109251 | 0.000156927 | 0.000125025 | 0.000140976 |
| 32 | 1-Aminocyclopropanecarboxylic acid | 22059-21-8 |  | C4H7NO2 | 101.047678 | phytohormone | 102 | 56 | + | 0.7 | 0.001111824 | 0.001090998 | 0.000809321 | 0.001004048 | 0.001169022 | 0.04803528 | 0.000860681 | 0.016688328 | 0.000874762 | 0.000992968 | 0.000933865 |
| 33 | 1-Caffeoylquinic acid | 1241-87-8 |  | C16H18O9 | 354.09508499999998 | Phenylpropanoids | 355.1 | 121 | + | 4.632333333 | 9.23286E-05 | 8.22852E-05 | 9.42133E-05 | 8.9609E-05 | 0.000167877 | 8.535E-05 | 7.14435E-05 | 0.000108223 | 7.36946E-05 | 0.000195926 | 0.00013481 |
| 34 | 1-Cinnamoylpyrrolidine | 52438-21-8 |  | C13H15NO | 201.115364 | Alkaloids | 202.1 | 72.1 | + | 7.06585 | 0.001246895 | 0.001630807 | 0.001387316 | 0.001421673 | 0.002617683 | 0.001736125 | 0.001748692 | 0.002034167 | 0.00201973 | 0.001711564 | 0.001865647 |
| 35 | 1-Isomangostin hydrate | 26063-95-6 |  | C24H28O7 | 428.18350500000003 | Xanthones | 429.2 | 355.1 | + | 13.4987 | 0.000195915 | 0.000114254 | 0.000279551 | 0.000196573 | 0.00038746 | 0.000116834 | 0.000275909 | 0.000260068 | 0.000317175 | 0.000223797 | 0.000270486 |
| 36 | 1-Kestose | 470-69-9 | C03661 | C18H32O16 | 504.16904 | Organooxygen compounds | 527.2 | 203.1 | + | 0.738166667 | 4.10316E-05 | 8.31653E-05 | 2.57951E-05 | 4.99973E-05 | 0.000221727 | 8.86487E-05 | 8.81891E-05 | 0.000132855 | 7.2974E-05 | 8.5748E-05 | 7.9361E-05 |
| 37 | 1-Methy-L-histidine | 332-80-9 | C01152 | C7H11N3O2 | 169.08510000000001 | amino acids | 170.1 | 124 | + | 0.61 | 0.000410476 | 0.000403669 | 0.000425334 | 0.00041316 | 0.000822899 | 0.000507282 | 0.000236896 | 0.000522359 | 0.000271364 | 0.000262023 | 0.000266694 |
| 38 | 1-Methyl-2-nonylquinolin-4(1H)-one | 68353-24-2 |  | C19H27NO | 285.20926400000002 | Alkaloids | 286.2 | 43.1 | + | 11.14408333 | 5.21284E-05 | 5.25322E-05 | 7.21308E-05 | 5.89305E-05 | 3.87333E-05 | 0.000122941 | 1.70909E-05 | 5.95885E-05 | 4.70308E-05 | 0.000115148 | 8.10894E-05 |
| 39 | 1-Methyladenine | 5142-22-3 | C02216 | C6H7N5 | 149.070145 | Nucleotide and its derivates | 150.1 | 94 | + | 1.309316667 | 0.028022947 | 0.032043564 | 0.030406866 | 0.030157792 | 0.03335841 | 0.031226893 | 0.029759624 | 0.031448309 | 0.027605232 | 0.030601379 | 0.029103306 |
| 40 | 1-Naphthol | 90-15-3 | C11714 | C10H8O | 144.057515 | Naphthalenes | 145.1 | 127.1 | + | 8.735741667 | 0.000132621 | 0.000383577 | 0.00021837 | 0.000244856 | 0.000189935 | 0.000297938 | 0.000203451 | 0.000230441 | 0.00020276 | 0.000269654 | 0.000236207 |
| 41 | 1-O-Caffeoylglucose | 14364-08-0 | C10433 | C15H18O9 | 342.09508499999998 | Carbohydrates | 343.1 | 163 | + | 4.917383333 | 0.001449422 | 0.002504155 | 0.001062757 | 0.001672111 | 0.001023714 | 0.002075645 | 0.00130159 | 0.001466983 | 0.000686554 | 0.000642258 | 0.000664406 |
| 42 | 1-Octacosanol | 557-61-9 | C08387 | C28H58O | 410.44876499999998 | Miscellaneous | 411.5 | 393.4 | + | 13.2026 | 0.000136989 | 0.000121303 | 0.000151249 | 0.000136514 | 0.000109044 | 0.000141155 | 0.000140608 | 0.000130269 | 0.000147644 | 0.00012051 | 0.000134077 |
| 43 | 1,2-Dihydroxy-6-methylcyclohexa-3,5-dienecarboxylate | | C06731 | C8H10O4 | 170.05790999999999 |  | 171.1 | 39 | + | 4.195966667 | 2.64325E-05 | 3.41146E-05 | 1.92769E-05 | 2.6608E-05 | 3.91263E-05 | 4.20016E-05 | 2.69613E-05 | 3.60297E-05 | 3.84287E-05 | 4.54536E-05 | 4.19411E-05 |
| 44 | 1,3-Diphenyl-2-propen-1-one;Chalcone | 94-41-7 | C01484 | C15H12O | 208.08881500000001 | Flavonoids | 209.1 | 103.1 | + | 8.251 | 5.86003E-05 | 0.000161317 | 5.45702E-05 | 9.14958E-05 | 0.000202205 | 3.59011E-05 | 4.81791E-05 | 9.54283E-05 | 0.000168955 | 0.00028912 | 0.000229038 |
| 45 | 1,3,6-Trihydroxy-5-methoxyxanthone | 41357-84-0 |  | C14H10O6 | 274.04773999999998 | Xanthones | 275.1 | 257 | + | 12.08293333 | 0.00045203 | 0.000322411 | 0.000153205 | 0.000309215 | 0.000212956 | 0.000417131 | 0.000211925 | 0.000280671 | 0.000446838 | 0.000324838 | 0.000385838 |
| 46 | 1,4-Cineole | 470-67-7 | C16909 | C10H18O | 154.13576499999999 | Monoterpenoids | 155.1 | 139.1 | + | 3.373616667 | 3.28779E-05 | 1.91892E-05 | 3.79306E-05 | 2.99992E-05 | 0.00013331 | 6.04123E-05 | 5.78246E-05 | 8.3849E-05 | 3.98147E-05 | 3.83763E-05 | 3.90955E-05 |
| 47 | 1,5-Anhydro-D-glucitol;trans-3-Hydroxycinnamic acid;p-Hydroxy-cinnamic acid | 154-58-5;588-30-7;14755-02-3;501-98-4;7400-08-0 | C16538;C07326;C12621;C00811 | C6H12O5;C9H8O3 | 164.06847500000001;164.04734500000001 | Alcohols and polyols;Phenylpropanoids | 165.1 | 147.0333333 | + | 5.0678 | 7.99024E-05 | 0.000140383 | 5.33031E-05 | 9.11962E-05 | 0.000117851 | 0.000196749 | 7.86776E-05 | 0.000131093 | 3.60074E-05 | 0.000104572 | 7.02896E-05 |
| 48 | 1,5,6-Trihydroxyxanthone | 5042-03-5 | C10082 | C13H8O5 | 244.03717499999999 | Xanthones | 245 | 227 | + | 10.48178333 | 6.7785E-05 | 4.90319E-05 | 1.12648E-06 | 3.93145E-05 | 0.000101937 | 8.89934E-05 | 8.20097E-05 | 9.09802E-05 | 0.000119419 | 7.88548E-05 | 9.91371E-05 |
| 49 | 1'-Acetoxychavicol acetate | 52946-22-2 | C10426 | C13H14O4 | 234.08921000000001 | Phenols | 235.1 | 175.1 | + | 10.55093333 | 0.00020791 | 0.000154717 | 7.32353E-05 | 0.000145288 | 0.000257183 | 0.000250528 | 0.000144471 | 0.000217394 | 0.00023694 | 0.000159314 | 0.000198127 |
| 51 | 11-Hydroxycanthin-6-one | 75969-83-4 | C09212 | C14H8N2O2 | 236.05857800000001 | Alkaloids | 237.1 | 209.1 | + | 8.7955 | 0.000128846 | 0.0001359 | 5.21244E-05 | 0.000105623 | 0.000182762 | 3.75346E-05 | 0.000146316 | 0.000122204 | 0.000174807 | 7.04899E-05 | 0.000122648 |
| 52 | 11-Keto-beta-boswellic acid | 17019-92-0 |  | C30H46O4 | 470.33960999999999 | Triterpenoids | 471.3 | 453.3 | + | 9.239225 | 0.000100636 | 0.000129384 | 9.36615E-05 | 0.000107894 | 9.22624E-05 | 0.000131063 | 8.5886E-05 | 0.00010307 | 0.000153835 | 7.78956E-05 | 0.000115865 |
| 53 | 11(R)-HETE | 73347-43-0 | C14780 | C20H32O3 | 320.23514499999999 | Fatty Acyls | 321.2 | 285.2 | + | 5.404333333 | 9.55122E-05 | 0.000176904 | 4.24079E-05 | 0.000104941 | 0.000152697 | 8.95265E-05 | 5.41315E-05 | 9.87851E-05 | 0.000187145 | 0.000101513 | 0.000144329 |
| 54 | 12-O-tetradecanoylphorbol-13-acetate | 16561-29-8 | C05151 | C36H56O8 | 616.39751999999999 | Diterpenoids | 617.4 | 581.4 | + | 12.48671667 | 0.011827 | 0.009812519 | 0.007033661 | 0.009557727 | 0.018690317 | 0.010259889 | 0.012330651 | 0.013760286 | 0.015103574 | 0.010680269 | 0.012891921 |
| 55 | 12-OPDA | 85551-10-6 | C01226 | C18H28O3 | 292.203845 |  | 293.2 | 275.2 | + | 11.39583333 | 0.000713031 | 0.000614656 | 0.000854114 | 0.000727267 | 0.000573501 | 0.000470563 | 0.000789246 | 0.000611103 | 0.000603028 | 0.000804694 | 0.000703861 |
| 56 | 12(13)-EpOME |  | C14826 | C18H32O3 | 296.23514499999999 | Fatty Acyls | 297.2 | 279.2 | + | 11.88253333 | 0.000152329 | 0.000202387 | 0.000212922 | 0.000189213 | 0.0003907 | 0.000249956 | 0.000133637 | 0.000258098 | 0.000232883 | 0.000209804 | 0.000221343 |
| 57 | 13-HPODE;13-L-Hydroperoxylinoleic acid | 33964-75-9 | C04717 | C18H32O4 | 312.23005999999998 | Lipids | 295.2 | 55.1 | + | 11.97805 | 0.00223318 | 0.00137761 | 0.00164467 | 0.00175182 | 0.001534438 | 0.00125306 | 0.001317043 | 0.001368181 | 0.001661886 | 0.001460414 | 0.00156115 |
| 58 | 16-Hydroxy hexadecanoic acid | 506-13-8 | C18218 | C16H32O3 | 272.23514499999999 | Lipids | 273.2 | 55.1 | + | 6.514016667 | 0.000149658 | 8.36365E-05 | 5.28484E-05 | 9.53809E-05 | 0.000132748 | 4.56123E-05 | 5.36037E-05 | 7.73214E-05 | 6.92634E-05 | 6.8747E-05 | 6.90052E-05 |
| 59 | 16(R)-HETE | 183509-22-0 | C14778 | C20H32O3 | 320.23514499999999 | Fatty Acyls | 303.2 | 91.1 | + | 6.874483333 | 0.000553156 | 0.000304395 | 0.000374093 | 0.000410548 | 0.000685796 | 0.000448775 | 0.000438119 | 0.00052423 | 0.000353438 | 0.000334606 | 0.000344022 |
| 60 | 17-Hydroxyprogesterone | 3168-01-2;68-96-2 | C01176 | C21H30O3 | 330.21949499999999 | Steroids and steroid derivatives | 331.2 | 97.1 | + | 11.77258333 | 0.001044763 | 0.001422865 | 0.001314347 | 0.001260658 | 0.000526452 | 0.001209197 | 0.001021351 | 0.000919 | 0.000826304 | 0.00081878 | 0.000822542 |
| 61 | 17alpha-Estradiol | 57-91-0 | C02537 | C18H24O2 | 272.17763000000002 | Steroids and steroid derivatives | 273.2 | 255.2 | + | 10.94786667 | 0.000283762 | 0.000466732 | 0.000147094 | 0.000299196 | 0.000483509 | 0.000432169 | 0.000216772 | 0.000377483 | 0.000162699 | 0.000242106 | 0.000202403 |
| 62 | 18alpha-Glycyrrhetinic acid | 1449-05-4 |  | C30H46O4 | 470.33960999999999 | Triterpenoids | 471.3 | 177.2 | + | 9.179766667 | 3.12768E-05 | 3.10702E-05 | 6.2465E-05 | 4.1604E-05 | 3.16304E-05 | 3.75385E-05 | 4.61378E-05 | 3.84356E-05 | 6.22783E-05 | 3.81391E-05 | 5.02087E-05 |
| 63 | 1beta-Hydroxyalantolactone | 68776-47-6 |  | C15H20O3 | 248.141245 | Sesquiterpenoids | 249.1 | 231.1 | + | 5.25755 | 0.000230264 | 0.000178762 | 0.000268521 | 0.000225849 | 0.000109645 | 0.000151471 | 0.000103266 | 0.000121461 | 0.000422821 | 0.000110958 | 0.00026689 |
| 64 | 1H-Indole-2,3-dione | 91-56-5 | C11129 | C8H5NO2 | 147.03202899999999 | Indoles and derivatives | 148 | 120 | + | 2.794233333 | 0.000408424 | 0.001044104 | 0.000782951 | 0.00074516 | 0.000926875 | 0.000762912 | 0.00050272 | 0.000730835 | 0.00059766 | 0.00081648 | 0.00070707 |
| 65 | 2-(Methylamino)benzoic acid | 119-68-6 | C03005 | C8H9NO2 | 151.06332900000001 | Benzene and substituted derivatives | 152.1 | 134.1 | + | 1.5816 | 0.001697012 | 0.002462913 | 0.001785332 | 0.001981752 | 0.001674194 | 0.000258333 | 0.00176385 | 0.001232126 | 0.00158809 | 0.001951532 | 0.001769811 |
| 66 | 2-Aminomuconate semialdehyde | | C03824 | C6H7NO3 | 141.04259400000001 | Carboxylic acids and derivatives | 142 | 96 | + | 1.191833333 | 0.001188286 | 0.001055715 | 0.000994338 | 0.001079446 | 0.001698632 | 0.000896743 | 0.001153981 | 0.001249785 | 0.001378082 | 0.001755691 | 0.001566886 |
| 67 | 2-Benzal-4-hydroxyacetophenone | 2657-25-2 | C14232 | C15H12O2 | 224.08373 | Flavonoids | 225.1 | 103.1 | + | 5.097983333 | 0.00023906 | 0.001457802 | 0.000218134 | 0.000638332 | 0.00178062 | 0.000276589 | 0.001761699 | 0.001272969 | 0.001648443 | 0.001765329 | 0.001706886 |
| 68 | 2-Biphenylol | 90-43-7 | C02499 | C12H10O | 170.07316499999999 | Benzene and substituted derivatives | 171.1 | 152.1 | + | 8.72735 | 3.89226E-05 | 2.38319E-05 | 8.74637E-05 | 5.00727E-05 | 3.14514E-05 | 2.41499E-05 | 6.21625E-05 | 3.92546E-05 | 5.91134E-05 | 4.96861E-05 | 5.43998E-05 |
| 69 | 2-Carboxybenzaldehyde | 119-67-5 | C03057 | C8H6O3 | 150.03169500000001 | Phenols | 151 | 105 | + | 3.071516667 | 0.000208841 | 0.000367487 | 0.000326613 | 0.00030098 | 0.0001663 | 0.000198402 | 0.000179863 | 0.000181522 | 0.000143916 | 0.000375528 | 0.000259722 |
| 72 | 2-Hydroxy-3-methylbenzalpyruvate | | C14086 | C11H10O4 | 206.05790999999999 |  | 207.1 | 133.1 | + | 5.068683333 | 0.005697491 | 0.005794863 | 0.00524337 | 0.005578575 | 0.009994156 | 0.006105691 | 0.004675201 | 0.006925016 | 0.00575032 | 0.005082719 | 0.005416519 |
| 74 | 2-Hydroxy-6-oxo-6-(2-carboxyphenyl)-hexa-2,4-dienoate | | C16264 | C13H10O6 | 262.04773999999998 |  | 263.1 | 171 | + | 8.72735 | 0.000214212 | 0.000209301 | 0.000298996 | 0.000240836 | 0.000288639 | 0.000219074 | 0.000500399 | 0.000336037 | 0.0005957 | 0.000354961 | 0.00047533 |
| 75 | 2-Hydroxybutanoic acid | 3347-90-8 | C05984 | C4H8O3 | 104.04734500000001 | Organic acids | 105.1 | 59 | + | 0.6372 | 0.014811719 | 0.017412717 | 0.01546228 | 0.015895572 | 0.025018857 | 0.018650729 | 0.016075668 | 0.019915085 | 0.015228652 | 0.018421192 | 0.016824922 |
| 76 | 2-Hydroxycinnamic acid | 583-17-5;614-60-8 | C01772 | C9H8O3 | 164.04734500000001 | Phenylpropanoids | 165.1 | 103.1 | + | 5.442483333 | 0.000259762 | 0.000716342 | 0.000455088 | 0.000477064 | 0.000382803 | 0.000402028 | 0.000668851 | 0.00048456 | 0.000316936 | 0.000664985 | 0.00049096 |
| 77 | 2-Hydroxyethanesulfonate | 107-36-8 | C05123 | C2H6O4S | 125.998681 | Organic acids | 127 | 109 | + | 0.755483333 | 0.000468587 | 0.000546162 | 0.000627545 | 0.000547431 | 0.000966348 | 0.000911184 | 0.000708845 | 0.000862126 | 0.000193862 | 0.000559486 | 0.000376674 |
| 78 | 2-Hydroxypyridine | 142-08-5 | C02502 | C5H5NO | 95.037114000000003 | Alkaloids | 96 | 39 | + | 3.575 | 0.000397613 | 0.000423119 | 0.000218121 | 0.000346284 | 0.000570492 | 0.000695469 | 0.000383308 | 0.000549756 | 0.000413314 | 0.000297482 | 0.000355398 |
| 79 | 2-Hydroxyxanthone | 1915-98-6 |  | C13H8O3 | 212.04734500000001 | Xanthones | 213.1 | 128.1 | + | 7.269033333 | 0.000289954 | 0.000135039 | 0.00020843 | 0.000211141 | 0.000187909 | 0.00026064 | 0.000455408 | 0.000301319 | 0.000228984 | 0.00020049 | 0.000214737 |
| 80 | 2-Isopropyl-3-oxosuccinate | | C04236 | C7H10O5 | 174.05282500000001 | Keto acids and derivatives | 175.1 | 129.1 | + | 0.800866667 | 0.000120105 | 5.70145E-05 | 0.000117505 | 9.82082E-05 | 0.000232717 | 0.000141816 | 0.000120748 | 0.000165094 | 0.000125117 | 0.000182242 | 0.00015368 |
| 81 | 2-Keto-6-acetamidocaproate | | C05548 | C8H13NO4 | 187.08445900000001 | Keto acids and derivatives | 188.1 | 100.1 | + | 1.47715 | 7.38358E-05 | 8.25483E-05 | 0.000116768 | 9.10507E-05 | 0.000517163 | 0.000189117 | 8.48498E-05 | 0.00026371 | 0.000218199 | 0.000323465 | 0.000270832 |
| 82 | 2-Picolinic acid | 14639-25-9;98-98-6 | C10164 | C6H5NO2 | 123.03202899999999 | Organic acids | 124 | 78 | + | 1.3261 | 0.024549876 | 0.02060121 | 0.027416889 | 0.024189325 | 0.029845551 | 0.026423828 | 0.023460428 | 0.026576602 | 0.022097054 | 0.019224492 | 0.020660773 |
| 83 | 2,3-Dehydrosilybin A | 25166-14-7 |  | C25H20O10 | 480.10565000000003 | Flavonoids | 481.1 | 463.1 | + | 5.531083333 | 0.000209724 | 0.00010854 | 0.000205675 | 0.000174646 | 0.000396766 | 8.6685E-05 | 0.000236148 | 0.000239866 | 0.000253127 | 0.000209138 | 0.000231133 |
| 84 | 2,3-Dihydro-2-phenyl-4H-benzopyran-4-one | 487-26-3 | C02099 | C15H12O2 | 224.08373 | Flavonoids | 225.1 | 121 | + | 4.928733333 | 0.003005757 | 0.002834495 | 0.003172518 | 0.003004257 | 0.005625505 | 0.00422181 | 0.004880111 | 0.004909142 | 0.004099274 | 0.003967279 | 0.004033276 |
| 85 | 2,5-Dihydroxybenzaldehyde | 1194-98-5 | C05585 | C7H6O3 | 138.03169500000001 | Phenols | 139 | 65 | + | 5.085466667 | 0.001204109 | 0.000841898 | 0.00109545 | 0.001047153 | 0.00214509 | 0.001925542 | 0.00167826 | 0.001916297 | 0.001395072 | 0.001480401 | 0.001437736 |
| 86 | 2,6-Dimethyl-7-octene-2,3,6-triol | 73815-21-1 |  | C10H20O3 | 188.141245 | Monoterpenoids | 189.1 | 153.1 | + | 3.819233333 | 6.1851E-05 | 3.94869E-05 | 5.8187E-05 | 5.3175E-05 | 0.000120274 | 5.32967E-05 | 4.71503E-05 | 7.35735E-05 | 8.86147E-05 | 0.000139488 | 0.000114051 |
| 87 | 2,6-Dimethylaniline | 87-62-7 | C11004 | C8H11N | 121.08914900000001 | Benzene and substituted derivatives | 122.1 | 105.1 | + | 2.01845 | 9.08068E-05 | 0.000147386 | 0.000120187 | 0.00011946 | 0.000257028 | 0.000231524 | 0.000249571 | 0.000246041 | 0.000156338 | 8.74345E-05 | 0.000121886 |
| 89 | 2'-Deoxyuridine;Deoxyuridine | 951-78-0 | C00526 | C9H12N2O5 | 228.074623 | Nucleotide and its derivates | 229.1 | 113 | + | 2.668733333 | 0.00042907 | 0.000464767 | 0.000176311 | 0.000356716 | 0.000154998 | 9.26826E-05 | 7.45633E-05 | 0.000107415 | 0.000446347 | 0.000107228 | 0.000276787 |
| 90 | 2'-Hydroxydaidzein | 7678-85-5 | C02495 | C15H10O5 | 270.05282499999998 | Flavonoids | 271.1 | 121 | + | 8.794483333 | 0.000199785 | 0.000147951 | 0.000114897 | 0.000154211 | 0.000208808 | 0.000146815 | 0.00020822 | 0.000187948 | 0.000207534 | 8.86754E-05 | 0.000148105 |
| 91 | 2'-O-Methyladenosine | 2140-79-6 | C04779 | C11H15N5O4 | 281.11240500000002 | Purine nucleosides | 282.1 | 136.1 | + | 2.719083333 | 0.001222616 | 0.000918375 | 0.001236429 | 0.001125807 | 0.001261696 | 0.00116629 | 0.000850284 | 0.001092757 | 0.000960791 | 0.001047198 | 0.001003995 |
| 92 | 2',6'-Dihydroxy 4'-methoxydihydrochalcone | 35241-55-5 | C09644 | C16H16O4 | 272.10485999999997 | Chalcones | 273.1 | 105.1 | + | 10.716 | 0.00037736 | 0.000373121 | 0.000240493 | 0.000330324 | 0.000358625 | 0.000360143 | 0.000237086 | 0.000318618 | 0.000575126 | 0.000415016 | 0.000495071 |
| 93 | 2(3H)-Benzothiazolethione | 149-30-4 | C14437 | C7H5NS2 | 166.98634100000001 | Benzothiazoles | 168 | 124 | + | 2.568033333 | 0.000210254 | 0.00018866 | 7.18379E-05 | 0.000156917 | 4.64315E-05 | 0.000101575 | 0.000232636 | 0.000126881 | 0.000200744 | 0.000158013 | 0.000179379 |
| 94 | 20-HETE | 79551-86-3 | C14748 | C20H32O3 | 320.23514499999999 | Fatty Acyls | 321.2 | 91.1 | + | 13.27901667 | 0.002986907 | 0.001577551 | 0.002000692 | 0.002188383 | 0.013227063 | 0.010945394 | 0.002325893 | 0.008832783 | 0.01182546 | 0.009860464 | 0.010842962 |
| 95 | 22-Dehydroclerosterol | 26315-07-1 |  | C29H46O | 410.35486500000002 | Steroids | 411.4 | 93.1 | + | 12.58741667 | 0.033115014 | 0.020993758 | 0.016481533 | 0.023530102 | 0.022456104 | 0.01843525 | 0.016042102 | 0.018977819 | 0.024389765 | 0.026704919 | 0.025547342 |
| 96 | 25-Hydroxyvitamin D2-25-glucuronide;25-Hydroxyvitamin D2 25-(beta-glucuronide) | | C03033 | C34H52O8 | 588.36622 | Organooxygen compounds | 589.4 | 395.3 | + | 13.69508333 | 0.000346296 | 0.001004369 | 0.000691083 | 0.000680583 | 0.001161392 | 0.000802073 | 0.00057186 | 0.000845108 | 0.000998745 | 0.000923827 | 0.000961286 |
| 97 | 3-(2,3-Dihydroxyphenyl)propanoate | | C04044 | C9H10O4 | 182.05790999999999 |  | 183.1 | 137.1 | + | 7.9889 | 2.1796E-05 | 6.72155E-05 | 7.20229E-05 | 5.36781E-05 | 0.000119978 | 5.58688E-05 | 4.10335E-05 | 7.22935E-05 | 3.8543E-05 | 4.91708E-05 | 4.38569E-05 |
| 98 | 3-(3,4-Dihydroxy-5-methoxy)-2-propenoic acid | 2041-35-2 | C05619 | C10H10O5 | 210.05282500000001 | Cinnamic acids and derivatives | 211.1 | 193 | + | 4.100283333 | 9.91284E-05 | 0.000146629 | 9.5071E-05 | 0.000113609 | 0.00020735 | 7.73008E-05 | 8.88879E-05 | 0.000124513 | 0.000267542 | 0.000133999 | 0.00020077 |
| 99 | 3-(4-Hydroxyphenyl)-1-propanol | 10210-17-0 |  | C9H12O2 | 152.08373 | Phenylpropanoids | 153.1 | 135.1 | + | 3.0883 | 8.10296E-05 | 0.000203536 | 0.00010683 | 0.000130465 | 0.000226846 | 0.000194508 | 0.000166691 | 0.000196015 | 0.000134682 | 0.000192401 | 0.000163541 |
| 100 | 3-(Carboxymethylamino)propanoic acid | 505-72-6 |  | C5H9NO4 | 147.05315899999999 | Alkaloids | 148.1 | 130.1 | + | 0.671566667 | 2.38476E-05 | 0.000126224 | 4.92497E-05 | 6.64404E-05 | 6.53482E-05 | 2.77058E-05 | 7.56865E-05 | 5.62468E-05 | 7.12156E-05 | 9.47545E-05 | 8.29851E-05 |
| 101 | 3-alpha-hydroxy-5-alpha-androstane-17-one 3-D-glucuronide | | C03033 | C25H38O8 | 466.25666999999999 | Organooxygen compounds | 467.3 | 273.2 | + | 12.35245 | 8.96568E-05 | 0.000130297 | 0.000280852 | 0.000166935 | 0.000139556 | 6.46086E-05 | 6.75137E-05 | 9.05594E-05 | 0.000178767 | 0.000408284 | 0.000293526 |
| 102 | 3-Aminoisobutanoic acid | 144-90-1 | C05145 | C4H9NO2 | 103.063329 | Amino acid and derivatives | 104.1 | 30 | + | 0.9401 | 0.00099908 | 0.000389817 | 0.001291396 | 0.000893431 | 0.001641469 | 0.000774611 | 0.001055795 | 0.001157292 | 0.00050347 | 0.001517057 | 0.001010264 |
| 103 | 3-Butylidenephthalide | 551-08-6 | C16924 | C12H12O2 | 188.08373 | Miscellaneous | 189.1 | 79.1 | + | 8.744133333 | 0.000143611 | 0.000389291 | 0.00025806 | 0.000263654 | 0.000255789 | 0.000244043 | 0.000333686 | 0.000277839 | 0.000408128 | 0.000605781 | 0.000506954 |
| 104 | 3-Galloylquinic acid | 17365-11-6 | C10834 | C14H16O10 | 344.07434999999998 | Phenols | 345.1 | 153 | + | 2.903683333 | 3.30091E-05 | 2.12875E-05 | 4.9712E-05 | 3.46696E-05 | 3.26789E-05 | 5.14971E-05 | 8.62975E-05 | 5.68245E-05 | 6.72269E-05 | 6.71016E-05 | 6.71643E-05 |
| 105 | 3-Hydroxy-2-methylpyridine | 1121-25-1 |  | C6H7NO | 109.052764 | Alkaloids | 110.1 | 39 | + | 1.43665 | 5.71354E-05 | 0.000148308 | 0.000131262 | 0.000112235 | 0.000192827 | 0.000178835 | 0.000191254 | 0.000187639 | 7.44415E-05 | 0.000109379 | 9.19102E-05 |
| 106 | 3-Hydroxy-4-methoxycinnamic acid;Isoferulic acid | 537-73-5 | C10470 | C10H10O4 | 194.05790999999999 | Phenylpropanoids | 195.1 | 149.1 | + | 4.867283333 | 0.000445996 | 0.000399432 | 0.000185423 | 0.000343617 | 0.000658919 | 0.000425561 | 0.000525049 | 0.00053651 | 0.000492571 | 0.000265682 | 0.000379126 |
| 107 | 3-Hydroxybenzoic acid | 99-06-9 | C00587 | C7H6O3 | 138.03169500000001 | Phenols | 139 | 121 | + | 8.39115 | 0.00018777 | 0.000207681 | 0.000169524 | 0.000188325 | 0.000149734 | 0.000171708 | 0.000238081 | 0.000186508 | 0.000195433 | 0.000191057 | 0.000193245 |
| 108 | 3-Hydroxycoumarin | 939-19-5 |  | C9H6O3 | 162.03169500000001 | Coumarins | 163 | 107 | + | 9.935716667 | 0.00145035 | 0.0012698 | 0.000986098 | 0.001235416 | 0.002164575 | 0.001545701 | 0.0016985 | 0.001802926 | 0.0017633 | 0.001517072 | 0.001640186 |
| 109 | 3-Hydroxypicolinic acid | 874-24-8 | C18620 | C6H5NO3 | 139.02694399999999 | Pyridines and derivatives | 140 | 94 | + | 4.145616667 | 0.001993663 | 0.002397415 | 0.002051185 | 0.002147421 | 0.002113984 | 0.001788579 | 0.001398005 | 0.001766856 | 0.002637121 | 0.001731714 | 0.002184418 |
| 110 | 3-Indolebutyric acid | 133-32-4 | C11284 | C12H13NO2 | 203.09460000000001 | phytohormone | 204 | 186 | + | 9 | 8.27076E-05 | 0.000347321 | 8.64514E-05 | 0.00017216 | 0.000165406 | 0.000195189 | 0.000145894 | 0.000168829 | 0.000195143 | 0.000271026 | 0.000233085 |
| 111 | 3-Isomangostin | 19275-46-8 |  | C24H26O6 | 410.17293999999998 | Xanthones | 411.2 | 55.1 | + | 13.09391667 | 9.94807E-05 | 2.51278E-05 | 4.85424E-05 | 5.7717E-05 | 9.24695E-05 | 6.64327E-05 | 3.49425E-05 | 6.46149E-05 | 5.21598E-05 | 7.41161E-05 | 6.31379E-05 |
| 112 | 3-Isopropylmalate | 921-28-8 | C04411 | C7H12O5 | 176.06847500000001 | Fatty Acyls | 177.1 | 131.1 | + | 10.79165 | 0.0002113 | 0.000427694 | 0.000184202 | 0.000274399 | 0.000260773 | 0.000138768 | 0.000186151 | 0.000195231 | 8.27402E-05 | 0.000152263 | 0.000117501 |
| 113 | 3-Methoxy-4-hydroxyphenylglycolaldehyde | 17592-23-3 | C05583 | C9H10O4 | 182.05790999999999 | Phenols | 183.1 | 123 | + | 7.9889 | 7.29349E-05 | 7.59608E-05 | 0.000112033 | 8.69761E-05 | 0.000170076 | 5.75535E-05 | 4.20131E-05 | 8.98808E-05 | 6.20075E-05 | 0.000147695 | 0.000104851 |
| 114 | 3-Methoxy-4,5-methylenedioxycinnamaldehyde | 74683-19-5 |  | C11H10O4 | 206.05790999999999 | Phenylpropanoids | 207.1 | 189.1 | + | 5.042633333 | 0.000401839 | 0.000854321 | 0.000451371 | 0.000569177 | 0.00130981 | 0.000992551 | 0.000516252 | 0.000939538 | 0.000891749 | 0.000656291 | 0.00077402 |
| 115 | 3-Methy-L-histidine | 368-16-1 | C01152 | C7H11N3O2 | 169.08510000000001 | amino acids | 170.1 | 96.1 | + | 0.61 | 7.91362E-05 | 0.000136847 | 0.000286654 | 0.000167546 | 0.000392125 | 0.000325814 | 0.000230182 | 0.00031604 | 0.000372453 | 0.000461817 | 0.000417135 |
| 116 | 3-Methylindole | 83-34-1 | C08313 | C9H9N | 131.073499 | Alkaloids | 132.1 | 130.1 | + | 3.809966667 | 0.003140401 | 0.000104254 | 0.002453395 | 0.00189935 | 0.000248424 | 0.00328694 | 0.003050048 | 0.002195137 | 0.003039211 | 0.003639711 | 0.003339461 |
| 117 | 3-Methylxanthine | 1076-22-8 | C16357 | C6H6N4O2 | 166.04907600000001 | Nucleotide and its derivates | 167.1 | 42 | + | 2.710216667 | 0.000165325 | 0.000136645 | 0.000162439 | 0.000154803 | 0.000213551 | 0.000191758 | 0.000154665 | 0.000186658 | 0.000168488 | 0.000163628 | 0.000166058 |
| 118 | 3-Nitro-L-tyrosine | 621-44-3 |  | C9H10N2O5 | 226.05897300000001 | Miscellaneous | 227.1 | 181.1 | + | 8.72735 | 0.000117299 | 0.0001137 | 0.000151557 | 0.000127519 | 5.33052E-05 | 8.5797E-05 | 4.16837E-05 | 6.0262E-05 | 0.000123911 | 0.000152225 | 0.000138068 |
| 119 | 3-O-Acetyl-11-keto-beta-boswellic acid | 67416-61-9 |  | C32H48O5 | 512.35017500000004 | Triterpenoids | 513.4 | 95.1 | + | 12.30211667 | 0.007422767 | 0.008168025 | 0.006973609 | 0.007521467 | 0.007827997 | 0.006152858 | 0.005229236 | 0.006403364 | 0.005094853 | 0.007765239 | 0.006430046 |
| 120 | 3-O-Acetylpinobanksin | 52117-69-8 | C16418 | C17H14O6 | 314.07904000000002 | Flavonoids | 315.1 | 91.1 | + | 10.209 | 7.995E-05 | 0.000137052 | 8.22847E-05 | 9.97623E-05 | 0.000110231 | 0.000119786 | 8.32967E-05 | 0.000104438 | 0.00012774 | 0.000218049 | 0.000172895 |
| 121 | 3-Octyl alcohol | 589-98-0 | C17144 | C8H18O | 130.13576499999999 | Miscellaneous | 131.1 | 113.1 | + | 1.2254 | 0.000317832 | 0.000205163 | 0.000190557 | 0.000237851 | 0.000366139 | 0.00021487 | 0.000154128 | 0.000245045 | 0.000212976 | 0.000218927 | 0.000215951 |
| 122 | 3-Oxoadipic acid | 689-31-6 | C00846 | C6H8O5 | 160.03717499999999 | Keto acids and derivatives | 161 | 125 | + | 2.827283333 | 3.06053E-05 | 4.74485E-05 | 3.25992E-05 | 3.68843E-05 | 2.33272E-05 | 4.0272E-05 | 3.9738E-05 | 3.44457E-05 | 4.86514E-05 | 2.79482E-05 | 3.82998E-05 |
| 123 | 3-Oxopomolic acid | 13849-90-6 |  | C30H46O4 | 470.33960999999999 | Triterpenoids | 471.3 | 407.3 | + | 12.26015833 | 1.18251E-06 | 4.25853E-05 | 0.000101427 | 4.83982E-05 | 0.000155523 | 6.33958E-05 | 1.23455E-06 | 7.33844E-05 | 0.000106806 | 0.000112669 | 0.000109738 |
| 124 | 3,4-Dihydrocoumarin | 119-84-6 | C02274 | C9H8O2 | 148.05242999999999 | Coumarins | 149.1 | 105.1 | + | 6.679833333 | 0.003043237 | 0.003382902 | 0.003796534 | 0.003407558 | 0.005749907 | 0.005535884 | 0.004400237 | 0.005228676 | 0.004629393 | 0.002906413 | 0.003767903 |
| 125 | 3,4-Dihydroxyphenylacetaldehyde | 5707-55-1 | C04043 | C8H8O3 | 152.04734500000001 | Benzene and substituted derivatives | 153.1 | 135 | + | 4.0537 | 0.000224856 | 0.000292802 | 0.000193348 | 0.000237002 | 0.00042164 | 0.000365048 | 0.000226907 | 0.000337865 | 0.000267641 | 0.000269263 | 0.000268452 |
| 126 | 3,4,5-Trimethoxycinnamyl alcohol | 30273-62-2 |  | C12H16O4 | 224.10486 | Phenylpropanoids | 225.1 | 207.1 | + | 10.40463333 | 0.000327147 | 0.000391525 | 0.000500478 | 0.000406384 | 0.00044408 | 0.000588119 | 0.000360151 | 0.000464117 | 0.000609181 | 0.000391926 | 0.000500554 |
| 127 | 3,8'-Biapigenin | 101140-06-1 |  | C30H18O10 | 538.09 | Flavonoids | 539.1 | 521.1 | + | 11.9161 | 0.001972201 | 0.001476053 | 0.001305562 | 0.001584605 | 0.0018373 | 0.000802358 | 0.001017835 | 0.001219164 | 0.002378322 | 0.002233833 | 0.002306078 |
| 128 | 3,9-Dihydroxypterocarpan | 61135-91-9 | C04271 | C15H12O4 | 256.07355999999999 | Flavonoids | 257.1 | 95 | + | 9.650416667 | 0.000199751 | 0.00013164 | 0.000114222 | 0.000148538 | 0.000312432 | 0.000159365 | 0.000135485 | 0.000202427 | 8.96519E-05 | 0.000160645 | 0.000125149 |
| 129 | 3',4'-Anhydrovinblastine | 38390-45-3 | C11641 | C46H56N4O8 | 792.40981599999998 | Alkaloids | 793.4 | 733.4 | + | 11.31191667 | 1.18251E-06 | 1.27649E-06 | 1.12648E-06 | 1.19516E-06 | 1.59752E-06 | 1.26522E-06 | 3.04528E-05 | 1.11052E-05 | 0.000109303 | 3.93405E-05 | 7.43218E-05 |
| 130 | 3',4',7-Trihydroxyisoflavone | 485-63-2 | C14313 | C15H10O5 | 270.05282499999998 | Flavonoids | 271.1 | 109 | + | 8.9791 | 0.000167699 | 0.000182274 | 9.82435E-05 | 0.000149405 | 0.000295046 | 0.000550766 | 0.000295153 | 0.000380322 | 0.000152737 | 0.000147318 | 0.000150027 |
| 131 | 4-(Ethoxymethyl)phenol | 57726-26-8 |  | C9H12O2 | 152.08373 | Phenols | 153.1 | 107 | + | 2.6294 | 0.002648163 | 0.002405296 | 0.002258392 | 0.002437284 | 0.004300684 | 0.003700315 | 0.002847698 | 0.003616232 | 0.002995396 | 0.002914966 | 0.002955181 |
| 132 | 4-Aminobutyric acid | 56-12-2 | C00334 | C4H9NO2 | 103.0633 | amino acids | 104.1 | 87.1 | + | 0.66 | 4.5222E-05 | 6.20848E-05 | 7.26916E-05 | 5.99995E-05 | 0.0003493 | 0.000241988 | 0.000236685 | 0.000275991 | 0.000122769 | 0.000125521 | 0.000124145 |
| 133 | 4-Guanidinobutyric acid | 463-00-3 | C01035 | C5H11N3O2 | 145.085127 | Organic acids | 146.1 | 86.1 | + | 1.314 | 0.003591795 | 0.004063302 | 0.003900633 | 0.00385191 | 0.003709778 | 0.003345341 | 0.002946227 | 0.003333782 | 0.004533435 | 0.004498045 | 0.00451574 |
| 134 | 4-Hydroxybenzaldehyde | 123-08-0 | C00633 | C7H6O2 | 122.03677999999999 | Phenols | 123 | 77 | + | 1.905108333 | 0.074981852 | 0.067093836 | 0.067619985 | 0.069898557 | 0.067118579 | 0.059752256 | 0.003367321 | 0.043412719 | 0.067108495 | 0.073714573 | 0.070411534 |
| 135 | 4-Hydroxybutanoic acid | 591-81-1 | C00989 | C4H8O3 | 104.04734500000001 | Fatty Acyls | 105.1 | 87 | + | 0.6372 | 0.000191672 | 0.000514028 | 0.00023091 | 0.000312203 | 0.000536491 | 0.000307667 | 0.000208827 | 0.000350995 | 0.000289842 | 0.000314576 | 0.000302209 |
| 136 | 4-Hydroxyphenyl-2-propionic acid | | C03080 | C9H10O3 | 166.062995 | Phenylpropanoic acids | 167.1 | 121.1 | + | 10.08676667 | 0.000142186 | 9.1352E-05 | 0.000149363 | 0.000127634 | 0.000213827 | 0.00016326 | 0.000185233 | 0.00018744 | 0.00019449 | 0.000182968 | 0.000188729 |
| 137 | 4-Hydroxyphenylacetylglutamic acid | | C05595 | C13H15NO6 | 281.08993900000002 |  | 282.1 | 107 | + | 12.9902 | 0.391379632 | 0.444172628 | 0.312825253 | 0.382792504 | 0.461619052 | 0.33657506 | 0.35550604 | 0.384566717 | 0.342977342 | 0.387176915 | 0.365077128 |
| 138 | 4-Isopropylbenzoic acid | 536-66-3 | C06578 | C10H12O2 | 164.08373 | Prenol lipids | 165.1 | 119.1 | + | 8.995883333 | 0.000655376 | 0.001621355 | 0.000480575 | 0.000919102 | 0.001123762 | 0.000693864 | 0.000993871 | 0.000937165 | 0.000670744 | 0.00054263 | 0.000606687 |
| 139 | 4-Methoxycinnamaldehyde | 1963-36-6 | C10475 | C10H10O2 | 162.06808000000001 | Phenylpropanoids | 163.1 | 145.1 | + | 6.797316667 | 0.000150367 | 4.48841E-05 | 0.000132109 | 0.00010912 | 0.000140456 | 0.000173377 | 6.83124E-05 | 0.000127382 | 5.1026E-05 | 0.00012997 | 9.04981E-05 |
| 140 | 4-Methoxyphenyl beta-D-glucopyranoside | 6032-32-2 | C17599 | C13H18O7 | 286.105255 | Phenols | 287.1 | 125.1 | + | 5.521816667 | 3.51306E-05 | 0.000213238 | 0.000346191 | 0.000198187 | 0.000280314 | 0.000124562 | 0.000348277 | 0.000251051 | 0.000415901 | 0.000210262 | 0.000313082 |
| 141 | 4-Methyl-5-thiazoleethanol | 137-00-8 | C04294 | C6H9NOS | 143.04048499999999 | Azoles | 144 | 113 | + | 2.655883333 | 0.00073961 | 0.000667231 | 0.000493488 | 0.000633443 | 2.40143E-05 | 0.000450476 | 0.000453865 | 0.000309452 | 0.000598699 | 0.000644628 | 0.000621663 |
| 142 | 4-Methylumbelliferone | 90-33-5 | C03081 | C10H8O3 | 176.04734500000001 | Coumarins | 177.1 | 149.1 | + | 7.25830811 | 0.000246836 | 9.19257E-05 | 0.000122006 | 0.000153589 | 0.000330728 | 0.000549286 | 0.000177157 | 0.00035239 | 0.000135012 | 0.000178913 | 0.000156963 |
| 143 | 4-Methylumbelliferyl acetate | 2747-05-9 | C03837 | C12H10O4 | 218.05790999999999 | Coumarins | 219.1 | 159 | + | 10.06246667 | 0.000150296 | 0.00026128 | 0.000143478 | 0.000185018 | 0.00018344 | 0.00018124 | 0.000278656 | 0.000214446 | 0.000245121 | 0.000179063 | 0.000212092 |
| 144 | 4-O-alpha-D-Galactopyranuronosyl-D-galacturonic acid | 5894-59-7 | C02273 | C12H18O13 | 370.07474500000001 | Organooxygen compounds | 353.1 | 97 | + | 11.4294 | 0.010324024 | 0.012271903 | 0.013155517 | 0.011917148 | 0.010728389 | 0.010267227 | 0.008085311 | 0.009693642 | 0.013886644 | 0.012919054 | 0.013402849 |
| 146 | 4-Pyridoxolactone | 4753-19-9 | C00971 | C8H7NO3 | 165.04259400000001 | Pyridines and derivatives | 166 | 106 | + | 4.917633333 | 0.000216357 | 8.8824E-05 | 0.000149248 | 0.000151476 | 0.000252336 | 0.000228064 | 0.000223132 | 0.000234511 | 0.000181606 | 0.000112948 | 0.000147277 |
| 147 | 4-Sulfobenzoate | 636-78-2 | C02236 | C7H6O5S | 201.993596 |  | 203 | 157 | + | 4.682683333 | 0.000164058 | 0.000178445 | 0.000258366 | 0.00020029 | 0.000230136 | 0.000120595 | 0.00022949 | 0.000193407 | 0.000139437 | 0.000272108 | 0.000205773 |
| 148 | 4,4'-Methylenediphenol | 620-92-8 | C14298 | C13H12O2 | 200.08373 | Phenols | 201.1 | 107 | + | 8.72735 | 5.21811E-05 | 8.82573E-05 | 0.000138729 | 9.30559E-05 | 6.40869E-05 | 0.000142977 | 0.000102607 | 0.000103224 | 0.000209378 | 0.00015194 | 0.000180659 |
| 149 | 4'-Demethylepipodophyllotoxin | 6559-91-7 | C21185 | C21H20O8 | 400.11581999999999 | Lignans | 399.1 | 369.1 | - | 8.049583333 | 5.91932E-05 | 1.27649E-06 | 6.66174E-05 | 4.23624E-05 | 0.000114744 | 0.000111595 | 4.74572E-05 | 9.12653E-05 | 0.000105079 | 9.83048E-05 | 0.000101692 |
| 150 | 4'-Demethylpodophyllotoxin | 40505-27-9 | C10553 | C21H20O8 | 400.11581999999999 | Lignans | 401.1 | 383.1 | + | 6.429166667 | 0.000474925 | 0.000327403 | 0.00036563 | 0.000389319 | 0.000484805 | 0.000358593 | 0.000346167 | 0.000396522 | 0.000332817 | 0.000321372 | 0.000327095 |
| 152 | 4'-Prenyloxyresveratrol | 69065-16-3 | C10283 | C19H20O4 | 312.13616000000002 | Phenols | 313.1 | 55.1 | + | 11.84896667 | 0.001938254 | 0.001553128 | 0.002233967 | 0.001908449 | 0.002393436 | 0.001091654 | 0.001414233 | 0.001633108 | 0.001985115 | 0.001165741 | 0.001575428 |
| 153 | 4',5,6,7-Tetramethoxyflavone;5,6,7-trimethoxy-2-(4-methoxyphenyl)chromen-4-one | 1168-42-9 | C14472 | C19H18O6 | 342.11034000000001 | Flavonoids | 343.1 | 107 | + | 11.66436667 | 0.009361361 | 0.018564003 | 0.017527943 | 0.015151102 | 0.025678959 | 0.014704764 | 1.23455E-06 | 0.013461652 | 0.019810918 | 0.01961726 | 0.019714089 |
| 155 | 5-(3-Pyridyl)-2-hydroxytetrahydrofuran | 53798-73-5 | C19578 | C9H11NO2 | 165.078979 | Pyridines and derivatives | 166.1 | 104 | + | 2.803 | 0.000470813 | 0.000472244 | 0.000404635 | 0.000449231 | 0.000593452 | 0.000407311 | 0.000526851 | 0.000509205 | 0.000393194 | 0.000390074 | 0.000391634 |
| 156 | 5-Aminolevulinate | 106-60-5 | C00430 | C5H9NO3 | 131.058244 | Organic acids | 132.1 | 55 | + | 1.5553 | 0.011993972 | 0.017895984 | 0.011589624 | 0.013826527 | 0.022630387 | 0.014712584 | 0.015835968 | 0.017726313 | 0.02094113 | 0.016671172 | 0.018806151 |
| 157 | 5-Aminovaleric acid | 660-88-8 | C00431 | C5H11NO2 | 117.078979 | Amino acid and derivatives | 118.1 | 55.1 | + | 1.107916667 | 0.031980136 | 0.037684595 | 0.041878995 | 0.037181242 | 0.037989001 | 0.028832914 | 0.037476195 | 0.034766037 | 0.039502011 | 0.040580462 | 0.040041236 |
| 158 | 5-Carboxyvanillic acid | 2134-91-0 | C18338 | C9H8O6 | 212.03209000000001 |  | 213 | 167 | + | 3.52165 | 5.72815E-05 | 8.43629E-05 | 3.32379E-05 | 5.82941E-05 | 6.73923E-05 | 6.67357E-05 | 4.89134E-05 | 6.10138E-05 | 5.09786E-05 | 0.000150086 | 0.000100532 |
| 159 | 5-Heneicosylresorcinol | 70110-59-7 |  | C27H48O2 | 404.36543 | Phenols | 405.4 | 123 | + | 12.05036667 | 0.000407053 | 0.000471727 | 0.000292708 | 0.000390496 | 0.000565417 | 0.00026073 | 0.000197735 | 0.000341294 | 0.000236055 | 0.000138169 | 0.000187112 |
| 160 | 5-Hydroxyconiferaldehyde | 249647-14-1 | C12204 | C10H10O4 | 194.05790999999999 |  | 195.1 | 177.1 | + | 4.612783333 | 2.75073E-05 | 6.95365E-05 | 0.000202074 | 9.9706E-05 | 6.50465E-05 | 0.000192107 | 7.49083E-05 | 0.000110687 | 7.43008E-05 | 8.19763E-05 | 7.81385E-05 |
| 161 | 5-Hydroxylysine | 1190-94-9 | C16741 | C6H14N2O3 | 162.10040000000001 | amino acids | 163.1 | 128 | + | 0.55 | 3.30092E-05 | 2.35323E-05 | 3.34648E-05 | 3.00021E-05 | 0.000106388 | 3.60702E-05 | 5.45295E-05 | 6.56628E-05 | 0.000111715 | 6.8793E-05 | 9.0254E-05 |
| 162 | 5-Hydroxymethylfurfural | 67-47-0 | C11101 | C6H6O3 | 126.031695 | Carbonyl compounds | 127 | 53 | + | 2.954033333 | 0.000779485 | 0.000844154 | 0.001221075 | 0.000948238 | 0.000737869 | 0.000940178 | 0.000961528 | 0.000879858 | 0.001063677 | 0.001381409 | 0.001222543 |
| 163 | 5-Methyl-2-furaldehyde | 620-02-0 | C11115 | C6H6O2 | 110.03677999999999 |  | 111 | 55.1 | + | 0.755483333 | 0.000932533 | 0.001046189 | 0.001059526 | 0.001012749 | 0.001277001 | 0.001023166 | 0.001042461 | 0.001114209 | 0.000914927 | 0.001177585 | 0.001046256 |
| 164 | 5-Methyldeoxycytidine | 838-07-3 | C03592 | C10H15N3O4 | 241.106257 | Pyrimidine nucleosides | 242.1 | 126.1 | + | 0.5373 | 0.000338441 | 0.000365869 | 0.000280239 | 0.000328183 | 0.000659813 | 0.000417784 | 0.000430335 | 0.000502644 | 0.000333344 | 0.000363549 | 0.000348447 |
| 166 | 5-Nonadecylresorcinol | 35176-46-6 |  | C25H44O2 | 376.33413000000002 | Phenols | 377.3 | 123 | + | 9.012666667 | 0.000167799 | 0.000138011 | 0.000125728 | 0.000143846 | 0.000322538 | 0.000184859 | 0.000184822 | 0.00023074 | 0.000404583 | 0.000265263 | 0.000334923 |
| 167 | 5-O-Caffeoylshikimic acid | 73263-62-4 | C10434 | C16H16O8 | 336.08452 | Phenylpropanoids | 337.1 | 121 | + | 9.9525 | 0.000173447 | 0.00033859 | 0.000231992 | 0.00024801 | 0.000171918 | 0.000303288 | 0.000159157 | 0.000211454 | 0.000250054 | 0.000406253 | 0.000328154 |
| 168 | 5-oxoproline | 98-79-3 | C01879 | C5H7NO3 | 129.04259400000001 | Amino acid and derivatives | 152 | 69 | + | 0.560733333 | 0.008385392 | 0.007645478 | 0.007112055 | 0.007714308 | 0.01218535 | 0.009201848 | 0.008247543 | 0.009878247 | 0.008341384 | 0.007318908 | 0.007830146 |
| 169 | 5-Phenyl-1,3-oxazinane-2,4-dione | | C16596 | C10H9NO3 | 191.058244 | Benzene and substituted derivatives | 192.1 | 103.1 | + | 2.803 | 0.001133051 | 0.000987218 | 0.001195474 | 0.001105248 | 0.00150884 | 0.001233948 | 0.001250158 | 0.001330982 | 0.001251997 | 0.001176975 | 0.001214486 |
| 170 | 5,6-DHET | 213382-49-1 | C14772 | C20H34O4 | 338.24570999999997 | Fatty Acyls | 339.3 | 303.2 | + | 13.03376667 | 9.16589E-05 | 0.000324776 | 0.000327452 | 0.000247962 | 0.000367226 | 0.000384412 | 0.000237512 | 0.000329717 | 0.00029198 | 0.000345118 | 0.000318549 |
| 171 | 5,6-Dihydroxyindole | 3131-52-0 | C05578 | C8H7NO2 | 149.04767899999999 | Indoles and derivatives | 150.1 | 78 | + | 6.679833333 | 0.000298739 | 0.000303692 | 0.000360749 | 0.00032106 | 0.000591268 | 0.000244743 | 0.000576379 | 0.000470796 | 0.000523067 | 0.00046458 | 0.000493824 |
| 172 | 5,6,7-Trimethoxyflavone | 973-67-1 | C10024 | C18H16O5 | 312.09977500000002 | Flavonoids | 313.1 | 281.1 | + | 10.8455 | 4.75636E-05 | 4.50431E-05 | 8.81609E-05 | 6.02558E-05 | 0.000129572 | 1.26522E-06 | 1.60442E-05 | 4.89604E-05 | 3.59056E-05 | 0.000117188 | 7.65466E-05 |
| 173 | 5,7-Dihydroxyisoflavone | 61-68-7 | C02168 | C15H15NO2 | 241.11027899999999 | Flavonoids | 242.1 | 224.1 | + | 14.23916667 | 1.0207E-05 | 1.26936E-05 | 9.58233E-06 | 1.08276E-05 | 1.28974E-05 | 9.33625E-06 | 1.05531E-05 | 1.09289E-05 | 1.98465E-05 | 4.02874E-06 | 1.19376E-05 |
| 174 | 5'-Deoxy-5-fluorouridine | 3094-09-5 | C12739 | C9H11FN2O5 | 246.065201 | 5'-deoxyribonucleosides | 245.1 | 42 | - | 1.882833333 | 7.39866E-05 | 0.000229058 | 1.04958E-05 | 0.000104513 | 1.59752E-06 | 9.75781E-05 | 9.14777E-06 | 3.61078E-05 | 0.000218269 | 3.98567E-05 | 0.000129063 |
| 175 | 5'-Deoxyadenosine | 4754-39-6 | C05198 | C10H13N5O3 | 251.10184000000001 | Nucleotide and its derivates | 252.1 | 136.1 | + | 2.408591667 | 0.006744277 | 0.008258135 | 0.006998963 | 0.007333791 | 0.011149342 | 0.010562858 | 0.009948126 | 0.010553442 | 0.007770974 | 0.007344449 | 0.007557712 |
| 176 | 6-(Furfurylamino)purine;Kinetin | 525-79-1 | C08272 | C10H9N5O | 215.08071000000001;215.08070000000001 | Alkaloids;phytohormone | 216.05 | 81 | + | 4.55 | 5.90783E-05 | 4.45121E-05 | 0.000110576 | 7.13888E-05 | 0.00011336 | 5.17494E-05 | 6.17985E-05 | 7.56361E-05 | 6.93489E-05 | 0.000179759 | 0.000124554 |
| 177 | 6-Amino-2-oxohexanoate | | C03239 | C6H11NO3 | 145.073894 |  | 146.1 | 72.1 | + | 0.772266667 | 0.595090277 | 0.641257278 | 0.610764656 | 0.61570407 | 0.840304953 | 0.725943472 | 0.614832935 | 0.72702712 | 0.412140804 | 0.700678579 | 0.556409692 |
| 178 | 6-Aminocaproic acid | 60-32-2 | C02378 | C6H13NO2 | 131.094629 | Fatty Acyls | 132.1 | 41 | + | 1.8374 | 0.025244541 | 0.025073366 | 0.027246101 | 0.02585467 | 0.020791649 | 0.01369768 | 0.016962838 | 0.017150722 | 0.020451846 | 0.019167599 | 0.019809722 |
| 179 | 6-Deoxyjacareubin | 16265-56-8 | C10059 | C18H14O5 | 310.08412499999997 | Xanthones | 311.1 | 255 | + | 6.57915 | 3.1759E-05 | 4.78868E-05 | 3.06555E-05 | 3.67671E-05 | 0.000103186 | 2.72975E-05 | 5.00408E-05 | 6.01746E-05 | 0.000107128 | 7.06692E-05 | 8.88985E-05 |
| 180 | 6-Gingerol; 6-Gingerol;[6]-Gingerol;Gingerol | 23513-14-6 | C10462 | C17H26O4 | 294.18311 | Phenols | 293.2 | 57 | - | 11.3875034 | 8.20769E-05 | 2.91935E-05 | 5.42922E-05 | 5.51875E-05 | 1.20116E-05 | 3.50728E-05 | 2.63655E-05 | 2.44833E-05 | 2.15048E-05 | 3.12597E-05 | 2.63822E-05 |
| 181 | 6-Hydroxystigmasta-4,22-dien-3-one | 36450-01-8 |  | C29H46O2 | 426.34978000000001 | Steroids | 427.4 | 409.3 | + | 10.58098333 | 0.000113205 | 7.46191E-05 | 6.66218E-05 | 8.48153E-05 | 4.72164E-05 | 0.000164172 | 3.16285E-05 | 8.10057E-05 | 3.35717E-05 | 4.28408E-05 | 3.82063E-05 |
| 182 | 6-Methoxymellein | 13410-15-6 | C02381 | C11H12O4 | 208.07355999999999 | Benzopyrans | 209.1 | 163.1 | + | 8.223866667 | 0.000206195 | 0.000182924 | 7.67E-05 | 0.000155273 | 0.000277792 | 0.000199018 | 0.000141533 | 0.000206114 | 0.000133504 | 0.000118074 | 0.000125789 |
| 184 | 6,7-Dehydroferruginol | 34539-84-9 |  | C20H28O | 284.21401500000002 | Diterpenoids | 285.2 | 269.2 | + | 9.06125 | 0.000290022 | 0.000240456 | 0.000547513 | 0.00035933 | 0.000376502 | 0.000259381 | 0.000181791 | 0.000272558 | 0.000184008 | 0.000247512 | 0.00021576 |
| 185 | 6,7,4'-Trihydroxyisoflavone | 17817-31-1 | C14314 | C15H10O5 | 270.05282499999998 | Flavonoids | 271.1 | 105 | + | 10.75808333 | 0.000166836 | 0.000170565 | 0.000248876 | 0.000195425 | 0.00030959 | 0.000220463 | 0.000171203 | 0.000233752 | 0.000195939 | 0.000275626 | 0.000235782 |
| 186 | 6,8-Diprenylnaringenin | 68236-11-3 | C09724 | C25H28O5 | 408.19367499999998 | Flavonoids | 409.2 | 69.1 | + | 12.62098333 | 0.000545382 | 0.000213389 | 0.000120246 | 0.000293005 | 0.000844185 | 0.000386609 | 0.000271802 | 0.000500865 | 0.000566231 | 0.000271522 | 0.000418876 |
| 187 | 6''-O-Acetylglycitin | 134859-96-4 |  | C24H24O11 | 488.131865 | Flavonoids | 489.1 | 285.1 | + | 12.35245 | 0.002716606 | 0.003595479 | 0.002143923 | 0.00281867 | 0.002182588 | 0.003031074 | 0.002994637 | 0.002736099 | 0.004198103 | 0.003556112 | 0.003877107 |
| 188 | 6(1H)-Azulenone, 2,3-dihydro-1,4-dimethyl | 71305-89-0 |  | C12H14O | 174.104465 | Miscellaneous | 175.1 | 157.1 | + | 8.72735 | 6.78603E-05 | 6.18116E-05 | 7.57846E-05 | 6.84855E-05 | 4.31252E-05 | 4.51076E-05 | 9.41808E-05 | 6.08045E-05 | 0.00011166 | 4.14198E-05 | 7.65398E-05 |
| 189 | 7-(4-Hydroxyphenyl)-1-phenyl-4-hepten-3-one | 100667-52-5 |  | C19H20O2 | 280.14632999999998 | Phenols | 281.2 | 105.1 | + | 12.53181667 | 0.006342107 | 0.006026503 | 0.0036602 | 0.005342937 | 0.00498063 | 0.003431398 | 0.004998136 | 0.004470055 | 0.005544015 | 0.004849798 | 0.005196907 |
| 190 | 7-Ethoxycoumarin | 31005-02-4 | C11052 | C11H10O3 | 190.062995 | Coumarins | 191.1 | 163 | + | 10.5655 | 3.00955E-05 | 1.27649E-06 | 2.92985E-05 | 2.02235E-05 | 0.000183932 | 0.000141408 | 0.000101023 | 0.000142121 | 0.000119613 | 6.76522E-05 | 9.36324E-05 |
| 191 | 7-Ethyl-10-Hydroxycamptothecin | 86639-52-3 | C11173 | C22H20N2O5 | 392.13722300000001 | Alkaloids | 393.1 | 263.1 | + | 6.282566667 | 0.000131072 | 0.000114421 | 6.29476E-05 | 0.000102813 | 9.05651E-05 | 0.000100532 | 5.19781E-05 | 8.1025E-05 | 6.37054E-05 | 7.96514E-05 | 7.16784E-05 |
| 192 | 7-Hydroxyflavone | 6665-86-7 | C11264 | C15H10O3 | 238.062995 | Flavonoids | 239.1 | 137 | + | 0.56235 | 0.001921192 | 0.00206038 | 0.00083795 | 0.001606507 | 0.002762013 | 0.001013944 | 0.003391259 | 0.002389072 | 0.002603748 | 0.002287768 | 0.002445758 |
| 193 | 7-Methylguanine | 578-76-7 | C02242 | C6H7N5O | 165.06505999999999 | Imidazopyrimidines | 166.1 | 149 | + | 1.561066667 | 0.003668604 | 0.004436643 | 0.004383405 | 0.004162884 | 0.006029866 | 0.010863151 | 0.0042736 | 0.007055539 | 0.008792652 | 0.009417122 | 0.009104887 |
| 194 | 7-O-Methylrosmanol | 113085-62-4 |  | C21H28O5 | 360.19367499999998 | Diterpenoids | 361.2 | 55.1 | + | 10.85878333 | 6.43537E-05 | 9.87097E-05 | 4.07587E-05 | 6.79407E-05 | 5.31692E-05 | 3.53589E-05 | 0.000110863 | 6.64638E-05 | 7.23016E-05 | 9.38983E-05 | 8.31E-05 |
| 195 | 7,8-Dihydroxyflavone | 38183-03-8 |  | C15H10O4 | 254.05790999999999 | Flavonoids | 255.1 | 129 | + | 7.37515 | 0.000109011 | 5.36282E-05 | 8.5657E-05 | 8.27655E-05 | 0.000190346 | 6.83785E-05 | 3.61936E-05 | 9.83059E-05 | 7.57676E-05 | 9.05939E-05 | 8.31807E-05 |
| 196 | 7alpha-Hydroxypregnenolone | 30626-96-1 | C18038 | C21H32O3 | 332.23514499999999 | Prenol lipids | 333.2 | 297.2 | + | 7.489933333 | 0.000103781 | 7.76651E-05 | 6.96917E-05 | 8.37126E-05 | 0.000184706 | 0.000106964 | 7.99073E-05 | 0.000123859 | 0.00017123 | 0.000100768 | 0.000135999 |
| 197 | 7beta-(3-Ethyl-cis-crotonoyloxy)-1alpha-(2-methylbutyryloxy)-3,14-dehydro-Z-notonipetranone | 80514-14-3 |  | C26H38O5 | 430.27192500000001 | Sesquiterpenoids | 431.3 | 55.1 | + | 13.86743333 | 5.15816E-05 | 2.79077E-05 | 3.2624E-05 | 3.73711E-05 | 6.6318E-05 | 1.26522E-06 | 2.70131E-05 | 3.15321E-05 | 6.5026E-05 | 2.51323E-05 | 4.50791E-05 |
| 198 | 8-Azabicyclo-3.2.1-octan-3-ol | 501-33-7 |  | C7H13NO | 127.09971400000001 | Alkaloids | 128.1 | 110.1 | + | 14.09186667 | 9.02983E-06 | 5.45431E-06 | 9.82354E-06 | 8.10256E-06 | 3.43785E-05 | 2.07468E-05 | 7.51826E-06 | 2.08812E-05 | 6.55334E-06 | 4.90746E-06 | 5.7304E-06 |
| 199 | 8-Hydroxybergapten | 1603-47-0 |  | C12H8O5 | 232.03717499999999 | Coumarins | 233 | 205.1 | + | 10.55668333 | 0.000472951 | 0.000559285 | 0.000583406 | 0.000538547 | 4.13579E-05 | 0.001100016 | 8.56939E-05 | 0.000409023 | 8.86458E-05 | 7.0565E-05 | 7.96054E-05 |
| 200 | 8-Hydroxypinoresinol | 81426-17-7 |  | C20H22O7 | 374.13655499999999 | Lignans | 375.1 | 135 | + | 8.458833333 | 8.23013E-05 | 6.0937E-05 | 4.97084E-05 | 6.43156E-05 | 5.34233E-05 | 5.6075E-05 | 1.97267E-05 | 4.3075E-05 | 8.18401E-05 | 4.68484E-05 | 6.43443E-05 |
| 201 | 8-Methylnonenoate | 59320-77-3 | C18202 | C10H18O2 | 170.13068000000001 | Fatty Acyls | 171.1 | 153.1 | + | 2.635166667 | 0.000102267 | 0.000132133 | 0.000208803 | 0.000147734 | 0.000534745 | 0.000244072 | 0.000143138 | 0.000307319 | 0.000174906 | 0.000170116 | 0.000172511 |
| 202 | 8,9-DiHETrE | 192461-96-4 | C14773 | C20H34O4 | 338.24570999999997 | Fatty Acyls | 339.3 | 95.1 | + | 13.03376667 | 0.009955359 | 0.113493044 | 0.08719684 | 0.070215081 | 0.149974442 | 0.123466234 | 0.118893036 | 0.130777904 | 0.154944692 | 0.135507187 | 0.145225939 |
| 203 | 9-Hydroxycalabaxanthone | 35349-68-9 |  | C24H24O6 | 408.15728999999999 | Xanthones | 409.2 | 55.1 | + | 13.2903 | 6.99214E-05 | 0.000108858 | 4.55516E-05 | 7.47771E-05 | 9.16825E-05 | 8.56114E-05 | 9.44684E-05 | 9.05874E-05 | 9.01638E-05 | 0.000156114 | 0.000123139 |
| 204 | 9-OxoODE | 54232-59-6 | C14766 | C18H30O3 | 294.21949499999999 | Fatty Acyls | 295.2 | 277.2 | + | 11.97805 | 0.000503004 | 0.000637105 | 0.000198075 | 0.000446061 | 0.000390602 | 0.000305369 | 0.000193827 | 0.000296599 | 0.000539123 | 0.000535294 | 0.000537208 |
| 205 | 9-Riburonosyladenine |  | C11501 | C10H11N5O5 | 281.07602000000003 |  | 282.1 | 136.1 | + | 12.53706667 | 0.000489571 | 0.000238697 | 5.96538E-05 | 0.000262641 | 0.000156245 | 0.000176475 | 0.000213961 | 0.000182227 | 6.4534E-05 | 0.000165498 | 0.000115016 |
| 206 | 9,10-DHOME | 263399-34-4 | C14828 | C18H34O4 | 314.24570999999997 | Fatty Acyls | 337.2 | 43 | + | 13.29651667 | 0.000675195 | 0.00082967 | 0.000866604 | 0.00079049 | 0.001256193 | 0.000510411 | 0.001309788 | 0.001025464 | 0.000937513 | 0.001214149 | 0.001075831 |
| 207 | 9(S)-HPOT | 111004-08-1 | C16321 | C18H30O4 | 310.21440999999999 | Fatty acyls | 309.2 | 121.1 | - | 11.13306667 | 0.000964745 | 0.000956482 | 0.000816294 | 0.000912507 | 0.000587605 | 0.000503203 | 0.00039455 | 0.000495119 | 0.000777443 | 0.000557082 | 0.000667262 |
| 209 | Absinthiin | 1362-42-1 | C09286 | C30H40O6 | 496.28249 | Triterpenoids | 497.3 | 461.3 | + | 12.49875 | 0.0001668 | 0.000282992 | 0.000157387 | 0.000202393 | 0.000317213 | 0.000648516 | 0.000204673 | 0.000390134 | 0.000130411 | 0.000453748 | 0.000292079 |
| 210 | Abyssinone V | 77263-11-7 | C09319 | C25H28O5 | 408.19367499999998 | Flavonoids | 409.2 | 153 | + | 12.6042 | 8.23036E-05 | 5.08277E-05 | 0.000141532 | 9.15543E-05 | 0.000202804 | 0.000138453 | 3.63525E-05 | 0.00012587 | 0.000113015 | 0.000130772 | 0.000121893 |
| 211 | Acacetin | 480-44-4 | C01470 | C16H12O5 | 284.06849999999997 | flavonoids | 285.3 | 269.8 | + | 11.03 | 1.58741E-05 | 6.47249E-05 | 6.16186E-05 | 4.74059E-05 | 1.04011E-05 | 4.19751E-05 | 0.00024409 | 9.88221E-05 | 0.000104714 | 2.56E-05 | 6.51572E-05 |
| 212 | Acanthoside B | 7374-79-0 | C10890 | C28H36O13 | 580.21559500000001 | Lignans | 581.2 | 419.2 | + | 6.514016667 | 0.000103304 | 0.000184089 | 0.00011975 | 0.000135714 | 8.38642E-05 | 0.000174156 | 0.000101421 | 0.000119814 | 8.25646E-05 | 0.000178283 | 0.000130424 |
| 213 | Acetamide | 60-35-5 | C06244 | C2H5NO | 59.037114000000003 | Alkaloids | 60 | 43 | + | 13.94701667 | 0.00846421 | 0.004322591 | 0.010812066 | 0.007866289 | 0.011066289 | 0.00559245 | 0.007306232 | 0.007988324 | 0.006009659 | 0.013151345 | 0.009580502 |
| 214 | Acetovanillone | 498-02-2 | C11380 | C9H10O3 | 166.062995 | Phenols | 167.1 | 43 | + | 5.236516667 | 0.000426922 | 0.000656254 | 0.00048339 | 0.000522189 | 0.00073154 | 0.000968533 | 0.000811966 | 0.000837346 | 0.000877481 | 0.000407019 | 0.00064225 |
| 215 | Acetyl tryptophan | 2280-01-5 | C03137 | C13H14N2O3 | 246.10044300000001 | Amino acid and derivatives | 247.1 | 130.1 | + | 7.972116667 | 0.000149197 | 0.000142173 | 0.0001762 | 0.000155857 | 0.000156859 | 0.000102326 | 0.000149814 | 0.000136333 | 0.000214667 | 8.8343E-05 | 0.000151505 |
| 216 | Acetylcholine chloride | 60-31-1 | C08201 | C7H16ClNO2 | 181.08695700000001 | Alkaloids | 182.1 | 122.1 | + | 3.306483333 | 0.000146976 | 0.000238759 | 0.000109018 | 0.000164917 | 0.000157188 | 0.000160906 | 0.000116693 | 0.000144929 | 9.87442E-05 | 0.000118561 | 0.000108652 |
| 217 | Acetylshikonin | 24502-78-1 | C17413 | C18H18O6 | 330.11034000000001 | Quinones | 331.1 | 55.1 | + | 6.394533333 | 8.30707E-05 | 0.000103994 | 3.88283E-05 | 7.52976E-05 | 9.49204E-05 | 4.03899E-05 | 8.90121E-05 | 7.47741E-05 | 6.59643E-05 | 0.000126072 | 9.60182E-05 |
| 218 | Acitretin | 55079-83-9 | D02754 | C21H26O3 | 326.18819500000001 | Miscellaneous | 327.2 | 131.1 | + | 7.470133333 | 4.72642E-05 | 0.000154943 | 0.000135737 | 0.000112648 | 0.00021095 | 0.000180657 | 7.05278E-05 | 0.000154045 | 3.59284E-05 | 0.000145612 | 9.07703E-05 |
| 219 | Aconitine | 302-27-2 | C06091 | C34H47NO11 | 645.31491400000004 | Alkaloids | 646.3 | 586.3 | + | 12.06715 | 0.003356825 | 0.002910326 | 0.003106304 | 0.003124485 | 0.014094587 | 0.011621355 | 0.014823711 | 0.013513217 | 0.008591669 | 0.006057953 | 0.007324811 |
| 220 | Adenosine 2',3'-cyclic phosphate | 634-01-5 | C02353 | C10H12N5O6P | 329.05252200000001 | Purine nucleotides | 330.1 | 136.1 | + | 1.855583333 | 0.000636256 | 0.000197813 | 0.000105601 | 0.000313223 | 8.78864E-05 | 7.9692E-05 | 7.21963E-05 | 7.99249E-05 | 0.001353941 | 0.001216831 | 0.001285386 |
| 221 | Adenosine 5'-monophosphate | 61-19-8 | C00020 | C10H14N5O7P | 347.063087 | Nucleotide and its derivates | 348.1 | 136.1 | + | 1.383466667 | 0.039992492 | 0.046511124 | 0.033912371 | 0.040138662 | 0.003395158 | 0.066178542 | 0.078198026 | 0.049257242 | 0.070170469 | 0.070080301 | 0.070125385 |
| 223 | Adrenosterone | 382-45-6 | C05285 | C19H24O3 | 300.17254500000001 | Steroids and steroid derivatives | 301.2 | 121.1 | + | 9.319866667 | 0.000121098 | 0.000119948 | 0.000131256 | 0.000124101 | 0.000128237 | 0.000154143 | 0.000104537 | 0.000128972 | 0.000142617 | 0.000160575 | 0.000151596 |
| 224 | Aflatoxin B1 | 1162-65-8 | C06800 | C17H12O6 | 312.06339000000003 | Coumarins and derivatives | 311.1 | 267 | - | 7.769266667 | 3.54553E-05 | 4.97666E-05 | 5.30612E-05 | 4.60944E-05 | 6.16178E-05 | 3.18514E-05 | 2.6728E-05 | 4.00657E-05 | 3.00473E-05 | 5.56081E-05 | 4.28277E-05 |
| 225 | Afzelin;Kaempferol 3-O-rhamnoside (Kaempferin) | 482-39-3 | C16911 | C21H20O10 | 432.10565000000003 | Flavonoids | 433.1 | 287.1 | + | 5.951916667 | 2.331935618 | 2.571930398 | 1.849945898 | 2.251270638 | 3.772301828 | 2.024099614 | 3.485834978 | 3.094078806 | 2.342029188 | 2.834933058 | 2.588481123 |
| 226 | Agnuside | 11027-63-7 | C09765 | C22H26O11 | 466.147515 | Iridoids | 467.2 | 121 | + | 12.36923333 | 0.000768423 | 0.000323961 | 0.000605123 | 0.000565836 | 0.000235969 | 0.000858803 | 0.000411023 | 0.000501932 | 0.000884733 | 0.000639545 | 0.000762139 |
| 227 | Agomelatine | 138112-76-2 |  | C15H17NO2 | 243.12592900000001 | Miscellaneous | 244.1 | 185.1 | + | 10.00285 | 6.92542E-05 | 5.79807E-05 | 6.14466E-05 | 6.28938E-05 | 3.38891E-05 | 1.25654E-05 | 2.51863E-05 | 2.38802E-05 | 5.24437E-05 | 3.302E-05 | 4.27319E-05 |
| 228 | Agrocybenine | 178764-92-6 |  | C12H18N2O | 206.14191299999999 | Alkaloids | 207.1 | 55.1 | + | 5.031333333 | 0.010829681 | 0.012190018 | 0.011008497 | 0.011342732 | 0.019224258 | 0.014526816 | 0.011121349 | 0.014957474 | 0.012664926 | 0.012921837 | 0.012793382 |
| 229 | AICAR | 3031-94-5 | C04677 | C9H15N4O8P | 338.06275299999999 | Imidazole ribonucleosides and ribonucleotides | 339.1 | 110 | + | 13.27901667 | 0.000333083 | 0.00068991 | 0.014492944 | 0.005171979 | 0.000494881 | 0.000788642 | 0.018818588 | 0.006700704 | 0.015963405 | 0.025089972 | 0.020526688 |
| 230 | Ailanthone | 981-15-7 | C08747 | C20H24O7 | 376.15220499999998 | Diterpenoids | 377.2 | 359.1 | + | 3.686983333 | 6.32069E-05 | 8.693E-05 | 2.55368E-05 | 5.85579E-05 | 0.000139695 | 3.93397E-05 | 7.34521E-05 | 8.41623E-05 | 5.35058E-05 | 5.00084E-05 | 5.17571E-05 |
| 231 | Ajmalicine | 483-04-5 | C09024 | C21H24N2O3 | 352.17869300000001 | Alkaloids | 353.2 | 144.1 | + | 7.619683333 | 1.80496E-05 | 3.95983E-05 | 5.34745E-05 | 3.70408E-05 | 5.4753E-05 | 7.2306E-05 | 4.04238E-05 | 5.58276E-05 | 5.34156E-05 | 5.53874E-05 | 5.44015E-05 |
| 232 | Ajmaline | 4360-12-7 | C06542 | C20H26N2O2 | 326.19942800000001 | Alkaloids | 327.2 | 144.1 | + | 11.91258333 | 0.001248688 | 0.002580829 | 1.12648E-06 | 0.001276881 | 0.001795776 | 0.002656983 | 0.002027781 | 0.00216018 | 0.000842201 | 0.002408604 | 0.001625403 |
| 234 | Allicin | 539-86-6 | C07600 | C6H10OS2 | 162.01730699999999 | Miscellaneous | 163 | 41 | + | 10.57346667 | 0.000221099 | 0.000371048 | 0.00031071 | 0.000300952 | 0.000367783 | 0.000182991 | 0.000113569 | 0.000221448 | 9.47486E-05 | 0.000172954 | 0.000133851 |
| 235 | Alloimperatorin | 642-05-7 | C09053 | C16H14O4 | 270.08920999999998 | Coumarins | 271.1 | 55.1 | + | 8.82855 | 0.000196813 | 0.000109253 | 0.000152954 | 0.000153007 | 0.000157086 | 0.000117616 | 0.000140006 | 0.000138236 | 0.000211137 | 0.000156589 | 0.000183863 |
| 237 | Aloeemodin | 481-72-1 | C10294 | C15H10O5 | 270.05282499999998 | Anthraquinones | 271.1 | 253.1 | + | 8.794083333 | 2.29721E-05 | 9.11382E-05 | 6.27736E-05 | 5.89613E-05 | 0.000276706 | 8.09125E-05 | 9.49359E-05 | 0.000150851 | 0.000171687 | 2.96454E-05 | 0.000100666 |
| 238 | Aloperine | 56293-29-9 | C10748 | C15H24N2 | 232.19394800000001 | Alkaloids | 233.2 | 174.1 | + | 7.2169 | 3.88925E-05 | 4.17441E-05 | 0.000115996 | 6.55442E-05 | 0.000280169 | 0.000348829 | 8.34657E-05 | 0.000237488 | 4.98884E-05 | 0.00019159 | 0.000120739 |
| 239 | Alpha-caryophyllene | 6753-98-6 | C09684 | C15H24 | 204.18780000000001 | Sesquiterpenoids | 205.2 | 81.1 | + | 11.58098333 | 0.003542747 | 0.002492231 | 0.002681833 | 0.002905604 | 0.003451907 | 0.002322441 | 0.002443762 | 0.00273937 | 0.002143452 | 0.002263059 | 0.002203255 |
| 240 | alpha-Cyperol;Alismol;Spathulenol | 20084-99-5;87827-55-2;6750-60-3 | C16945;C17462 | C15H24O | 220.182715 | Prenol lipids;Sesquiterpenoids | 221.2 | 203.2 | + | 10.89009444 | 2.41758E-05 | 3.55869E-05 | 1.63652E-05 | 2.5376E-05 | 3.23353E-05 | 3.59555E-05 | 5.16206E-05 | 3.99704E-05 | 2.60327E-05 | 7.93015E-05 | 5.26671E-05 |
| 241 | alpha-Cyperone | 473-08-5 | C17090 | C15H22O | 218.16706500000001 | Sesquiterpenoids | 219.2 | 67.1 | + | 12.20181426 | 0.002714555 | 0.002205765 | 0.002130821 | 0.00235038 | 0.00090562 | 0.002853433 | 0.002937639 | 0.002232231 | 0.000581458 | 0.00285531 | 0.001718384 |
| 243 | alpha-Irone | 79-69-6;35124-13-1 | C09690 | C14H22O | 206.16706500000001 | Prenol lipids | 207.2 | 147.1 | + | 5.0519 | 0.004236166 | 0.013106622 | 0.003157808 | 0.006833532 | 0.029284007 | 0.004688444 | 0.022501506 | 0.018824652 | 0.013536451 | 0.005614416 | 0.009575433 |
| 244 | alpha-Peltatin | 568-53-6 | C10729 | C21H20O8 | 400.11581999999999 | Lignans | 401.1 | 165.1 | + | 12.58741667 | 0.000493756 | 0.000553389 | 0.000433646 | 0.000493597 | 0.000564967 | 0.00033586 | 0.000397605 | 0.000432811 | 0.000353881 | 0.000310104 | 0.000331992 |
| 245 | Alpha-Terpineol;Patulin | 10482-56-1;149-29-1 | C11393;C16748 | C10H18O;C7H6O4 | 154.13576499999999;154.02661000000001 | Monoterpenoids;Pyrans | 155.05 | 137.05 | + | 3.456525 | 0.002238177 | 0.001911058 | 0.001497217 | 0.001882151 | 0.003953845 | 0.002546299 | 0.003130276 | 0.00321014 | 0.002660543 | 0.002607674 | 0.002634109 |
| 246 | alpha-Tocopherol | 59-02-9 | C02477 | C29H50O2 | 430.38108 | Phenols | 431.4 | 165.1 | + | 13.36581667 | 0.000123265 | 0.000389918 | 0.000298037 | 0.000270406 | 0.000553804 | 0.000558762 | 0.000183007 | 0.000431858 | 0.000164175 | 0.000191475 | 0.000177825 |
| 248 | Altechromone A | 38412-47-4 |  | C11H10O3 | 190.062995 | Phenols | 191.1 | 173.1 | + | 8.246916667 | 0.000754765 | 0.000813295 | 0.000600055 | 0.000722705 | 0.000987373 | 0.000549583 | 0.000589646 | 0.000708867 | 0.000720626 | 0.000978062 | 0.000849344 |
| 249 | Amabiline;Supinine | 17958-43-9;551-58-6 | C10263;C10403 | C15H25NO4 | 283.178359 | Alkaloids | 284.2 | 122.1 | + | 12.9902 | 0.004719782 | 0.005203147 | 0.003109171 | 0.004344033 | 0.004616949 | 0.003328052 | 0.003323372 | 0.003756124 | 0.004504429 | 0.004175502 | 0.004339966 |
| 251 | Aminomalonic acid | 1068-84-4 | C00872 | C3H5NO4 | 119.02185900000001 | Organic acids | 120 | 74 | + | 0.654783333 | 0.000426263 | 0.000684554 | 0.000595871 | 0.000568896 | 0.001088303 | 0.000875253 | 0.000704143 | 0.000889233 | 0.000853149 | 0.000741093 | 0.000797121 |
| 252 | Anabasine | 494-52-0;13078-04-1 | C06180;C11357 | C10H14N2 | 162.11569800000001 | Alkaloids | 163.1 | 118.1 | + | 2.8737 | 0.000182234 | 0.000280349 | 0.000201469 | 0.000221351 | 0.000489267 | 0.000220262 | 0.000315205 | 0.000341578 | 0.000173326 | 0.000229905 | 0.000201615 |
| 253 | Anacrotine | 5096-49-1 | C10277 | C18H25NO6 | 351.16818899999998 | Alkaloids | 352.2 | 334.2 | + | 9.549716667 | 1.99882E-05 | 4.84087E-05 | 3.89044E-05 | 3.57671E-05 | 0.000132225 | 3.25125E-05 | 5.74002E-05 | 7.40459E-05 | 7.79635E-05 | 0.000129369 | 0.000103666 |
| 254 | Anatabine | 2743-90-0;581-49-7 | C10126 | C10H12N2 | 160.10004799999999 | Alkaloids | 161.1 | 144.1 | + | 2.819783333 | 0.000182005 | 0.000291476 | 0.000172429 | 0.000215304 | 0.000309734 | 0.000245835 | 0.000110296 | 0.000221955 | 0.000228103 | 0.000170965 | 0.000199534 |
| 255 | Androstenedione | 63-05-8 | C00280 | C19H26O2 | 286.19328000000002 | Steroids and steroid derivatives | 287.2 | 243.2 | + | 6.54725 | 0.000100262 | 0.00013964 | 8.13491E-05 | 0.000107084 | 0.000104081 | 0.000189291 | 0.000271252 | 0.000188208 | 0.000166159 | 7.55785E-05 | 0.000120869 |
| 256 | Androsterone | 53-41-8 | C00523 | C19H30O2 | 290.22458 | Steroids and steroid derivatives | 291.2 | 255.2 | + | 5.0561 | 2.28118E-05 | 0.000197029 | 3.82289E-05 | 8.60233E-05 | 2.15009E-05 | 6.03319E-05 | 3.26999E-05 | 3.81776E-05 | 0.000108244 | 0.000108988 | 0.000108616 |
| 257 | Angelicin | 523-50-2 | C09060 | C11H6O3 | 186.03169500000001 | Phenylpropanoids | 187 | 131 | + | 8.9727245 | 0.000375832 | 0.00020361 | 0.000318246 | 0.000299229 | 0.00049579 | 0.000549769 | 0.000146043 | 0.000397201 | 0.000388844 | 0.000496302 | 0.000442573 |
| 258 | Anisatin | 5230-87-5 | C09294 | C15H20O8 | 328.11581999999999 | Sesquiterpenoids | 329.1 | 311.1 | + | 5.422883333 | 0.000353919 | 0.000539238 | 0.00059661 | 0.000496589 | 0.00135843 | 0.000429049 | 0.000557001 | 0.000781493 | 0.00046332 | 0.001743796 | 0.001103558 |
| 259 | Anthranilic acid | 118-92-3 | C00108 | C7H7NO2 | 137.04767899999999 | Benzoic acid derivatives | 138.1 | 120 | + | 2.87835 | 0.00228008 | 0.002767934 | 0.002117011 | 0.002388342 | 0.003936343 | 0.002827814 | 0.002622148 | 0.003128768 | 0.002810632 | 0.002731188 | 0.00277091 |
| 260 | Anthraquinone | 84-65-1 | C16207 | C14H8O2 | 208.05242999999999 | Anthraquinones | 209.1 | 105 | + | 8.075916667 | 0.003036778 | 0.002450369 | 0.002715826 | 0.002734324 | 0.002497086 | 0.002805298 | 0.002850782 | 0.002717722 | 0.003110358 | 0.003657941 | 0.00338415 |
| 261 | Antirhine | 16049-28-8 | C09033 | C19H24N2O | 296.18886300000003 | Alkaloids | 297.2 | 279.2 | + | 11.61401667 | 0.000101005 | 2.94321E-05 | 0.000212922 | 0.000114453 | 0.000147723 | 0.000249956 | 4.08083E-05 | 0.000146162 | 0.000232883 | 0.000120658 | 0.00017677 |
| 262 | Anwulignan | 107534-93-0 |  | C20H24O4 | 328.16746000000001 | Lignans | 329.2 | 135 | + | 11.59723333 | 0.000797228 | 0.000509086 | 0.000533158 | 0.000613158 | 0.00159853 | 1.26522E-06 | 0.000538619 | 0.000712805 | 0.000498786 | 0.000426864 | 0.000462825 |
| 264 | Arachidonic acid | 506-32-1 | C00219 | C20H32O2 | 304.24023 | Fatty Acyls | 305.2 | 93.1 | + | 12.40275 | 0.000879649 | 0.001234219 | 0.000762375 | 0.000958747 | 0.00278691 | 0.001149124 | 0.000759007 | 0.001565013 | 0.001649668 | 0.001841014 | 0.001745341 |
| 265 | Arctiin | 20362-31-6 | C16915 | C27H34O11 | 534.21011499999997 | Lignans;Others | 535.2 | 177.1 | + | 8.238892878 | 0.01199916 | 0.015278222 | 0.018439011 | 0.015238798 | 0.020055777 | 0.000205524 | 0.019496096 | 0.013252466 | 0.008710748 | 0.023361093 | 0.016035921 |
| 266 | Arecaidine | 499-04-7 | C10128 | C7H11NO2 | 141.078979 | Alkaloids | 142.1 | 96.1 | + | 1.544283333 | 0.00237187 | 0.002142773 | 0.002276183 | 0.002263608 | 0.001688308 | 0.00157242 | 0.001266098 | 0.001508942 | 0.00209009 | 0.001944207 | 0.002017149 |
| 267 | Aristolindiquinone | 86533-36-0 | C10300 | C12H10O4 | 218.05790999999999 | Quinones | 219.1 | 105 | + | 11.14408333 | 0.000275785 | 0.000279409 | 0.001116256 | 0.00055715 | 0.001336056 | 0.000811918 | 0.000647163 | 0.000931713 | 0.001523166 | 0.000292495 | 0.000907831 |
| 268 | Aromadendrin | 480-20-6 | C00974 | C15H12O6 | 288.06339000000003 | Flavonoids | 289.1 | 107 | + | 11.21151667 | 0.000521739 | 0.000536798 | 0.000243634 | 0.000434057 | 0.000871422 | 0.000355178 | 0.000572627 | 0.000599742 | 0.000474409 | 0.000851805 | 0.000663107 |
| 269 | Artemisinin | 63968-64-9 | C09538 | C15H22O5 | 282.146725 | Sesquiterpenoids | 283.2 | 110.1 | + | 13.02376667 | 0.027756588 | 0.029772172 | 0.020873816 | 0.026134192 | 0.029054453 | 0.019945925 | 0.022878443 | 0.023959607 | 0.024682817 | 0.028749073 | 0.026715945 |
| 270 | Ascorbic acid | 50-81-7 | C00072 | C6H8O6 | 176.03209000000001 | Vitamins | 177 | 95 | + | 1.1282 | 0.001215229 | 0.001178348 | 0.000957515 | 0.001117031 | 0.001349617 | 0.001339769 | 0.001199225 | 0.001296204 | 0.001171728 | 0.00130533 | 0.001238529 |
| 271 | Asebogenin | 520-42-3 | C09471 | C16H16O5 | 288.09977500000002 | Chalcones | 289.1 | 139 | + | 9.069766667 | 1.56231E-05 | 7.42019E-05 | 2.28509E-05 | 3.75586E-05 | 2.54784E-05 | 3.40793E-05 | 5.31697E-06 | 2.16249E-05 | 2.91371E-05 | 4.49854E-05 | 3.70612E-05 |
| 272 | Asiatic acid;Euscaphic acid | 464-92-6;53155-25-2 | C08617;C17890 | C30H48O5 | 488.35017499999998 | Triterpenoids | 489.4 | 425.3 | + | 12.35245 | 0.000106252 | 8.98029E-05 | 8.87382E-05 | 9.49312E-05 | 0.00010712 | 1.86406E-05 | 0.000101353 | 7.57045E-05 | 0.000201135 | 0.00014371 | 0.000172423 |
| 273 | Asp-Phe methyl ester;Aspartame | 22839-47-0 | C11045 | C14H18N2O5 | 294.12157300000001 | Carboxylic acids and derivatives | 295.1 | 120.1 | + | 3.05875 | 0.009246283 | 0.008944342 | 0.008857595 | 0.009016073 | 0.007196424 | 0.007766924 | 0.007581482 | 0.007514944 | 0.00914445 | 0.00759473 | 0.00836959 |
| 274 | Astaxanthin | 7542-45-2;472-61-7 | C08580 | C40H52O4 | 596.38656000000003 | Miscellaneous | 597.4 | 147.1 | + | 13.55891667 | 0.007244364 | 0.00743667 | 0.006836366 | 0.007172467 | 0.008258842 | 0.002264005 | 0.003531154 | 0.004684667 | 0.004619583 | 0.007061402 | 0.005840493 |
| 275 | Astragalin | 480-10-4 | C12249 | C21H20O11 | 448.10059999999999 | flavonoids | 449 | 286.9 | + | 6.52 | 0.008913691 | 0.008104332 | 0.007023065 | 0.008013696 | 0.017252041 | 0.012118793 | 0.016068171 | 0.015146335 | 0.014053633 | 0.012315689 | 0.013184661 |
| 277 | Atherosperminine | 5531-98-6 |  | C20H23NO2 | 309.17287900000002 | Alkaloids | 310.2 | 265.1 | + | 6.142783333 | 4.52915E-05 | 7.65417E-05 | 5.17333E-05 | 5.78555E-05 | 2.58734E-05 | 0.000117521 | 6.56131E-05 | 6.96692E-05 | 4.881E-05 | 0.000262102 | 0.000155456 |
| 278 | Atranorin | 479-20-9 |  | C19H18O8 | 374.10016999999999 | Phenols | 375.1 | 179 | + | 5.255583333 | 0.000298928 | 0.000239008 | 0.000318642 | 0.000285526 | 0.00107374 | 0.000463823 | 0.000545807 | 0.000694457 | 0.000581326 | 0.000484497 | 0.000532911 |
| 279 | Aurantio-obtusin | 67979-25-3 | C17670 | C17H14O7 | 330.07395500000001 | Anthraquinones | 331.1 | 313.1 | + | 10.27991295 | 0.000324481 | 0.00039412 | 0.000303457 | 0.000340686 | 0.000527648 | 0.000207572 | 0.000608663 | 0.000447961 | 0.000363764 | 0.000392411 | 0.000378087 |
| 280 | Auriculine | 22595-00-2 | C10280 | C31H45NO8 | 559.31451900000002 | Alkaloids | 560.3 | 124.1 | + | 7.518983333 | 9.94736E-06 | 4.4015E-05 | 6.73222E-05 | 4.04282E-05 | 2.75405E-05 | 2.65845E-05 | 2.25723E-05 | 2.55657E-05 | 1.44174E-05 | 2.56669E-05 | 2.00421E-05 |
| 281 | Azacitidine | 320-67-2 | C11262 | C8H12N4O5 | 244.080771 | Organooxygen compounds | 243.1 | 110 | - | 2.584033333 | 2.44456E-05 | 4.55917E-05 | 2.81307E-05 | 3.27227E-05 | 2.25589E-05 | 9.36454E-06 | 2.63047E-05 | 1.94094E-05 | 1.43335E-05 | 7.09587E-05 | 4.26461E-05 |
| 283 | Baccatin III | 27548-93-2 | C11900 | C31H38O11 | 586.24141499999996 | Diterpenoids | 587.2 | 105 | + | 9.9486174 | 1.466E-05 | 2.50797E-05 | 3.90678E-05 | 2.62692E-05 | 7.40246E-06 | 4.07509E-05 | 1.70588E-05 | 2.17374E-05 | 4.94586E-05 | 2.42592E-05 | 3.68589E-05 |
| 284 | Baicalein | 491-67-8 | C10023 | C15H10O5 | 270.05282499999998 | Flavonoids | 271.1 | 123 | + | 9.38 | 4.65176E-05 | 4.95787E-05 | 3.13061E-05 | 4.24675E-05 | 6.30996E-05 | 3.45651E-05 | 4.75569E-05 | 4.84072E-05 | 4.73658E-05 | 6.38885E-05 | 5.56272E-05 |
| 285 | Baicalin | 21967-41-9 | C10025 | C21H18O11 | 446.0849 | flavonoids | 447 | 430.9 | + | 7.26 | 5.76699E-05 | 0.000119789 | 0.00013941 | 0.000105623 | 0.000119076 | 0.000321971 | 0.000121196 | 0.000187414 | 0.00023234 | 0.000117179 | 0.00017476 |
| 286 | Bakkenolide A | 19906-72-0 | C09350 | C15H22O2 | 234.16198 | Miscellaneous | 235.2 | 149.1 | + | 11.84896667 | 0.000436788 | 0.000424989 | 0.000125699 | 0.000329158 | 1.59752E-06 | 1.26522E-06 | 0.000375353 | 0.000126072 | 0.000433495 | 0.000521989 | 0.000477742 |
| 287 | Baldrinal | 18234-46-3 | C16811 | C12H10O4 | 218.05790999999999 | Miscellaneous | 219.1 | 159 | + | 8.352866667 | 0.000236823 | 0.000202504 | 0.000153112 | 0.00019748 | 0.000184874 | 0.0001597 | 0.000277352 | 0.000207309 | 0.001048208 | 0.000351556 | 0.000699882 |
| 288 | Baptifoline | 732-50-3 | C10755 | C15H20N2O2 | 260.15247799999997 | Alkaloids | 261.2 | 243.1 | + | 9.986066667 | 9.96518E-05 | 4.00757E-05 | 0.000112345 | 8.4024E-05 | 9.33681E-05 | 4.11524E-05 | 2.17223E-05 | 5.2081E-05 | 7.84002E-05 | 0.000166083 | 0.000122242 |
| 289 | Batatasin III | 56684-87-8 |  | C15H16O3 | 244.10994500000001 | Phenols | 245.1 | 107 | + | 8.72735 | 4.52893E-05 | 0.000128934 | 0.000219972 | 0.000131399 | 0.00011776 | 9.81639E-05 | 0.000111489 | 0.000109138 | 0.0001617 | 0.000157797 | 0.000159749 |
| 290 | Bellendine | 32152-73-1 | C10846 | C12H15NO2 | 205.11027899999999 | Alkaloids | 206.1 | 148.1 | + | 5.672866667 | 0.000589377 | 0.000510541 | 0.000446578 | 0.000515499 | 0.000622529 | 0.000411811 | 0.000593387 | 0.000542576 | 0.000545517 | 0.000426546 | 0.000486031 |
| 291 | Bellidifolin | 2798-25-6 | C10053 | C14H10O6 | 274.04773999999998 | Xanthones | 275.1 | 257 | + | 12.05036667 | 0.00045203 | 0.000322411 | 0.000153205 | 0.000309215 | 0.000212956 | 0.000417131 | 0.000211925 | 0.000280671 | 0.000446838 | 0.000324838 | 0.000385838 |
| 292 | Benzaldehyde | 100-52-7 | C00261 | C7H6O | 106.041865 | Phenols | 107 | 79 | + | 2.819783333 | 0.004780061 | 0.006117403 | 0.005191089 | 0.005362851 | 0.006645986 | 0.005939669 | 0.006039178 | 0.006208278 | 0.005928732 | 0.005429014 | 0.005678873 |
| 293 | Benzoin | 119-53-9 | C01408 | C14H12O2 | 212.08373 | Stilbenes | 213.1 | 105 | + | 4.6659 | 0.000138803 | 0.000174323 | 0.000116687 | 0.000143271 | 0.000120658 | 0.0002164 | 0.000260206 | 0.000199088 | 7.23042E-05 | 6.95994E-05 | 7.09518E-05 |
| 294 | Benzoyl glucuronide (Benzoic acid);2-Phenylethanol glucuronide | 19237-53-7 | C03033 | C13H14O8;C14H18O7 | 298.06887;298.105255 | Organooxygen compounds | 299.1 | 105.05 | + | 12.06715 | 0.000351781 | 0.000722436 | 0.00040438 | 0.000492866 | 0.000455781 | 0.000585604 | 0.000684835 | 0.000575407 | 0.000931663 | 0.000322395 | 0.000627029 |
| 295 | Benzoylagmatine |  | C02253 | C12H18N4O | 234.14806100000001 |  | 235.2 | 105 | + | 10.55668333 | 0.000418214 | 0.000629635 | 0.000636684 | 0.000561511 | 0.000834481 | 0.000418983 | 0.000340469 | 0.000531311 | 0.000981091 | 0.000567608 | 0.00077435 |
| 296 | Benzyl cinnamate | 103-41-3 |  | C16H14O2 | 238.09938 | Phenylpropanoids | 239.1 | 91.1 | + | 4.553166667 | 0.000164499 | 0.000387875 | 0.000244616 | 0.000265664 | 0.000106202 | 0.00016228 | 0.000168168 | 0.00014555 | 0.000303713 | 0.0002721 | 0.000287906 |
| 297 | Berberine hydrochloride | 633-65-8;2086-83-1 | C00757;C12679 | C20H17NO4 | 335.11575900000003 | Alkaloids | 336.1 | 320.1 | + | 8.347466667 | 0.000174055 | 0.000241485 | 0.000129332 | 0.000181624 | 0.000121754 | 9.95198E-05 | 0.000180792 | 0.000134022 | 0.000133718 | 0.000139029 | 0.000136373 |
| 298 | Bergamotine | 7380-40-7 | C22152 | C21H22O4 | 338.15181000000001 | Coumarins | 339.2 | 203 | + | 13.0338 | 5.78787E-05 | 0.000321765 | 0.000177502 | 0.000185715 | 0.000155416 | 0.000245862 | 0.000135272 | 0.00017885 | 6.36042E-05 | 0.000151622 | 0.000107613 |
| 299 | Bergaptol | 486-60-2 | C00758 | C11H6O4 | 202.02661000000001 | Coumarins | 203 | 175 | + | 7.435066667 | 5.15122E-05 | 9.68026E-05 | 0.000115967 | 8.80941E-05 | 0.000143243 | 0.000108939 | 0.000125877 | 0.00012602 | 0.000100955 | 9.12276E-05 | 9.60912E-05 |
| 300 | beta-Alanine | 107-95-9 | C00099 | C3H7NO2 | 89.047700000000006 | amino acids | 90.1 | 72.1 | + | 0.64 | 0.015499593 | 0.023770837 | 0.025391494 | 0.021553975 | 0.019829661 | 0.026718706 | 0.015586059 | 0.020711475 | 0.023299776 | 0.014835888 | 0.019067832 |
| 301 | beta-Asarone | 5273-86-9 | C10430 | C12H16O3 | 208.10994500000001 | Phenylpropanoids | 209.1 | 41 | + | 4.943433333 | 0.00011844 | 0.000180532 | 0.000223839 | 0.00017427 | 0.000272928 | 0.0001379 | 0.000223508 | 0.000211446 | 0.000114732 | 0.000120744 | 0.000117738 |
| 302 | Beta-Carotene | 7235-40-7 | C02094 | C40H56 | 536.43820000000005 | Vitamins | 536.4 | 444.4 | + | 13.59433333 | 4.23466E-06 | 3.8914E-05 | 3.34376E-05 | 2.55288E-05 | 5.39532E-05 | 4.46768E-05 | 4.13458E-05 | 4.66586E-05 | 4.23133E-05 | 1.34613E-05 | 2.78873E-05 |
| 303 | beta-Citronellol;(S)-(-)-beta-Citronellol | 106-22-9;1117-61-9;7540-51-4 | C09849;C11386 | C10H20O | 156.15141499999999 | Monoterpenoids | 157.2 | 139.1 | + | 1.410016667 | 0.000106565 | 0.000232075 | 9.98454E-05 | 0.000146162 | 0.000147572 | 0.000211549 | 0.000153376 | 0.000170832 | 6.08887E-05 | 0.000176097 | 0.000118493 |
| 304 | beta-Costic acid | 3650-43-9 |  | C15H22O2 | 234.16198 | Sesquiterpenoids | 235.2 | 189.2 | + | 8.895183333 | 0.000242488 | 0.000118764 | 0.000214789 | 0.000192014 | 0.000142289 | 0.000253247 | 7.25892E-05 | 0.000156042 | 0.000133576 | 0.000108992 | 0.000121284 |
| 305 | Beta-D-Fructose 2-phosphate | | C03267 | C6H13O9P | 260.02972199999999 | Organooxygen compounds | 261 | 99 | + | 0.749216667 | 0.001111158 | 0.00047753 | 0.001604934 | 0.001064541 | 0.001561321 | 0.000451628 | 0.000989847 | 0.001000932 | 0.001806202 | 0.001602792 | 0.001704497 |
| 306 | Beta-D-Glucose;alpha-D-Glucose | 492-61-5;492-62-6 | C00221;C00267 | C6H12O6 | 180.06339 | Organooxygen compounds | 181.1 | 163.1 | + | 3.272916667 | 0.000130619 | 0.000106381 | 0.000104458 | 0.000113819 | 9.09738E-05 | 0.000174711 | 9.63545E-05 | 0.00012068 | 0.000109413 | 0.000108575 | 0.000108994 |
| 308 | beta-Glucogallin | 13405-60-2 |  | C13H16O10 | 332.07434999999998 | Phenols | 333.1 | 153 | + | 2.416983333 | 0.000127916 | 0.000135174 | 8.73496E-05 | 0.000116813 | 9.43869E-05 | 0.00010955 | 0.000106136 | 0.000103358 | 0.000120317 | 0.000113916 | 0.000117116 |
| 309 | beta-Lactose | 5965-66-2 | C01970 | C12H22O11 | 342.11621500000001 | Carbohydrates | 343.1 | 163.1 | + | 4.5652 | 0.001449422 | 0.002504155 | 0.001062757 | 0.001672111 | 0.002026732 | 0.002075645 | 0.001652987 | 0.001918455 | 0.001614731 | 0.002264521 | 0.001939626 |
| 310 | Beta-Leucine | 5699-54-7 | C02486 | C6H13NO2 | 131.094629 | Carboxylic acids and derivatives | 132.1 | 72.1 | + | 1.796016667 | 0.003014334 | 0.001713593 | 0.007474306 | 0.004067411 | 0.015547189 | 0.00311538 | 0.015356669 | 0.011339746 | 0.016010909 | 0.003537669 | 0.009774289 |
| 311 | Beta-mangostin | 20931-37-7 |  | C25H28O6 | 424.18858999999998 | Xanthones | 425.2 | 69.1 | + | 13.20838333 | 0.000240163 | 0.000284147 | 0.00011667 | 0.00021366 | 0.000286665 | 0.000262354 | 0.000207867 | 0.000252295 | 0.000226623 | 0.00012472 | 0.000175672 |
| 312 | beta-Nicotinamide mononucleotide | 1094-61-7 | C00455 | C11H15N2O8P | 334.05660499999999 | Nucleotide and its derivates | 335.1 | 123.1 | + | 0.729483333 | 0.001965446 | 0.001735959 | 0.001537724 | 0.001746376 | 0.001699835 | 0.001567706 | 0.001617744 | 0.001628428 | 0.001816516 | 0.001245388 | 0.001530952 |
| 313 | Beta-Tocopherol | 148-03-8 | C14152 | C28H48O2 | 416.36543 | Phenols | 417.4 | 151.1 | + | 12.0702 | 0.000183321 | 0.000258067 | 0.000170874 | 0.000204087 | 0.000321948 | 0.000200172 | 0.000179621 | 0.000233914 | 0.000246566 | 0.000429142 | 0.000337854 |
| 314 | Beta-Tocotrienol | 490-23-3 | C14154 | C28H42O2 | 410.31848000000002 | Phenols | 411.3 | 151.1 | + | 12.18463333 | 0.000409304 | 0.000190282 | 0.000236906 | 0.000278831 | 0.000304297 | 0.000287649 | 0.000152155 | 0.000248034 | 0.000326027 | 0.00035603 | 0.000341028 |
| 315 | Beta-Tyrosine |  | C04368 | C9H11NO3 | 181.073894 | Carboxylic acids and derivatives | 182.1 | 123 | + | 2.416983333 | 0.000925813 | 0.001042026 | 0.000657806 | 0.000875215 | 0.001209299 | 0.000632267 | 0.000937877 | 0.000926481 | 0.00130019 | 0.000831633 | 0.001065912 |
| 316 | Betaine aldehyde | 7418-61-3 | C00576 | C5H11NO | 101.084064 | Organonitrogen compounds | 102.1 | 58.1 | + | 0.753433333 | 0.000703883 | 0.000767897 | 0.000647359 | 0.00070638 | 0.00059172 | 0.000660893 | 0.000550812 | 0.000601142 | 0.00043223 | 0.000564051 | 0.000498141 |
| 317 | Betulin | 473-98-3 | C08618 | C30H50O2 | 442.38108 | Triterpenoids | 425.4 | 95.1 | + | 12.3169 | 0.000621988 | 0.000678549 | 0.000821585 | 0.000707374 | 0.000477376 | 0.000468492 | 0.000279022 | 0.000408297 | 0.000436027 | 0.000464841 | 0.000450434 |
| 318 | Biliverdin | 114-25-0 | C00500 | C33H34N4O6 | 582.24783600000001 | Tetrapyrroles and derivatives | 583.3 | 547.2 | + | 12.82238333 | 0.00580677 | 0.004888055 | 0.004917358 | 0.005204061 | 0.005531591 | 0.004743129 | 0.004137141 | 0.004803953 | 0.006315871 | 0.004424807 | 0.005370339 |
| 319 | Biochanin A | 491-80-5 | C00814 | C16H12O5 | 284.06849999999997 | flavonoids | 285.3 | 269.3 | + | 11.21 | 0.000622053 | 0.000482679 | 0.000225694 | 0.000443475 | 0.000919149 | 0.000463366 | 0.000416367 | 0.000599627 | 0.000254249 | 0.000360293 | 0.000307271 |
| 320 | Bisabolol oxide A | 22567-36-8 | C16773 | C15H26O2 | 238.19327999999999 | Sesquiterpenoids | 239.2 | 55.1 | + | 11.49528333 | 0.000741145 | 0.000774139 | 0.000528386 | 0.000681224 | 0.001114447 | 0.000670345 | 0.000822756 | 0.000869183 | 0.000646143 | 0.000451021 | 0.000548582 |
| 321 | Boldione | 897-06-3 | C20144 | C19H24O2 | 284.17763000000002 | Steroids and steroid derivatives | 285.2 | 121.1 | + | 9.0665 | 0.000306218 | 0.000539629 | 0.000264941 | 0.000370263 | 0.000393282 | 0.000224497 | 0.000123269 | 0.000247016 | 0.000393239 | 0.000540784 | 0.000467012 |
| 322 | Bornyl acetate | 5655-61-8 | C09837 | C12H20O2 | 196.14633000000001 | Monoterpenoids | 197.2 | 137.1 | + | 6.541066667 | 0.000611543 | 0.000717046 | 0.000712535 | 0.000680375 | 0.000817509 | 0.00075771 | 0.000654565 | 0.000743261 | 0.000923709 | 0.000701907 | 0.000812808 |
| 323 | Bovinic acid | 2540-56-9 | C04056 | C18H32O2 | 280.24023 | Fatty Acyls | 303.2 | 183 | + | 6.856383333 | 0.000409207 | 0.00032827 | 0.000252038 | 0.000329838 | 0.000864958 | 0.000323331 | 0.000392424 | 0.000526904 | 0.000346256 | 0.000532653 | 0.000439455 |
| 325 | Brazilin | 474-07-7 | C09920 | C16H14O5 | 286.08412499999997 | Flavonoids | 287.1 | 121 | + | 6.201633333 | 0.002933101 | 0.003835422 | 0.001375554 | 0.002714692 | 0.005374014 | 0.003103154 | 0.004961794 | 0.004479654 | 0.004531609 | 0.004563075 | 0.004547342 |
| 326 | Bromocriptine | 25614-03-3 | C06856 | C32H40BrN5O5 | 653.22128299999997 | Ergoline and derivatives | 654.2 | 636.2 | + | 12.03518333 | 0.000471463 | 0.002635674 | 0.00126847 | 0.001458536 | 0.003076255 | 0.002617139 | 0.001580097 | 0.002424497 | 0.002529018 | 0.000905731 | 0.001717375 |
| 327 | Bruceine D | 21499-66-1 | C08752 | C20H26O9 | 410.15768500000001 | Diterpenoids | 411.2 | 393.2 | + | 4.835430767 | 9.45952E-05 | 0.000131618 | 7.24149E-05 | 9.95426E-05 | 0.000344075 | 7.46441E-05 | 0.000189613 | 0.000202777 | 9.85727E-05 | 0.000264888 | 0.00018173 |
| 328 | Brucine | 4845-99-2;357-57-3 | C09084 | C23H26N2O4 | 394.189258 | Alkaloids | 395.2 | 244.1 | + | 4.376633333 | 1.59742E-05 | 9.9182E-06 | 4.50691E-05 | 2.36538E-05 | 1.71588E-05 | 3.85172E-05 | 1.58032E-05 | 2.38264E-05 | 3.47976E-05 | 2.69869E-05 | 3.08922E-05 |
| 329 | Bufalin | 465-21-4 | C16922 | C24H34O4 | 386.24570999999997 | Steroids and steroid derivatives | 387.3 | 107.1 | + | 5.045816667 | 0.002618723 | 0.00253002 | 0.004592801 | 0.003247181 | 0.003199075 | 0.00159139 | 0.00197256 | 0.002254342 | 0.003249963 | 0.003378531 | 0.003314247 |
| 331 | Butein | 487-52-5 | C08578 | C15H12O5 | 272.06847499999998 | Chalcones | 273.1 | 137 | + | 7.06635 | 6.44952E-05 | 5.54274E-05 | 7.87732E-05 | 6.62319E-05 | 8.82857E-05 | 0.000122922 | 0.000160303 | 0.000123837 | 8.06792E-05 | 8.26802E-05 | 8.16797E-05 |
| 332 | Butin;2'-Hydroxydihydrodaidzein | 492-14-8 | C09614;C03567 | C15H12O5 | 272.06847499999998 | Flavonoids;Isoflavonoids | 273.1 | 121 | + | 8.82805 | 0.001269842 | 0.000665298 | 0.00081736 | 0.0009175 | 0.001764265 | 0.000900437 | 0.000766808 | 0.001143837 | 0.000899648 | 0.000719642 | 0.000809645 |
| 333 | Butyl 4-Hydroxybenzoate | 94-26-8 |  | C11H14O3 | 194.09429499999999 | Lignans | 195.1 | 57.1 | + | 4.5632 | 5.5417E-05 | 8.3929E-05 | 7.33424E-05 | 7.08961E-05 | 0.000109812 | 6.59759E-05 | 0.000109925 | 9.52376E-05 | 6.64266E-05 | 4.38315E-05 | 5.5129E-05 |
| 334 | Byakangelicol | 26091-79-2 | C16925 | C17H16O6 | 316.09469000000001 | Coumarins | 317.1 | 85.1 | + | 12.06715 | 0.000124355 | 0.000102196 | 0.000253896 | 0.000160149 | 0.000128399 | 0.000200402 | 0.000226561 | 0.000185121 | 8.52007E-05 | 0.00016465 | 0.000124926 |
| 335 | C-Veratroylglycol | 168293-10-5 |  | C10H12O5 | 212.06847500000001 | Phenylpropanoids | 213.1 | 123 | + | 3.474433333 | 0.000234195 | 0.000174217 | 0.000244999 | 0.000217804 | 0.000192421 | 0.000198232 | 0.000258641 | 0.000216431 | 0.000230094 | 0.000138065 | 0.000184079 |
| 336 | Caffeic acid | 331-39-5 | C01481 | C9H8O4 | 180.04226 | Phenylpropanoids | 181 | 163 | + | 4.4394025 | 0.000128187 | 6.07328E-05 | 8.26427E-05 | 9.05207E-05 | 0.000211758 | 9.85843E-05 | 4.1377E-05 | 0.00011724 | 4.19426E-05 | 8.59628E-05 | 6.39527E-05 |
| 337 | Caffeine | 58-08-2 | C07481 | C8H10N4O2 | 194.080376 | Alkaloids | 195.1 | 138.1 | + | 4.502233333 | 0.006066587 | 0.001712913 | 0.000717555 | 0.002832352 | 0.000652302 | 0.004637863 | 0.000524738 | 0.001938301 | 0.00261875 | 0.001894029 | 0.002256389 |
| 338 | Cajanol | 61020-70-0 | C10204 | C17H16O6 | 316.09469000000001 | Isoflavonoids | 317.1 | 123 | + | 12.05036667 | 0.000937324 | 0.000779716 | 0.000471679 | 0.000729573 | 0.000549709 | 0.000284139 | 0.000642792 | 0.000492213 | 0.000547384 | 0.000569351 | 0.000558368 |
| 339 | Calenduloside E | 26020-14-4 | C08964 | C36H56O9 | 632.39243499999998 | Triterpenoids | 633.4 | 439.4 | + | 12.55385 | 1.18251E-06 | 4.95173E-05 | 9.97919E-05 | 5.01639E-05 | 1.59752E-06 | 9.10983E-05 | 4.72498E-05 | 4.66485E-05 | 0.000134117 | 6.21928E-05 | 9.81548E-05 |
| 340 | Calycosin | 20575-57-9 | C01562 | C16H12O5 | 284.06847499999998 | Flavonoids | 285.1 | 267.1 | + | 9.096583333 | 9.47334E-05 | 0.000116546 | 8.52231E-05 | 9.88342E-05 | 0.000141174 | 0.000136104 | 0.000108317 | 0.000128532 | 0.000152429 | 0.000193294 | 0.000172862 |
| 341 | Calystegine B2 | 127414-85-1 | C10851 | C7H13NO4 | 175.08445900000001 | Alkaloids | 176.1 | 158.1 | + | 2.954033333 | 7.51852E-05 | 0.000109844 | 4.25075E-05 | 7.58457E-05 | 0.000107287 | 5.04371E-05 | 0.000212582 | 0.000123435 | 0.000258355 | 0.000217149 | 0.000237752 |
| 342 | Campesterol | 474-62-4 | C01789 | C28H48O | 400.37051500000001 | Steroids and steroid derivatives | 383.4 | 147.1 | + | 12.1844 | 0.01894516 | 0.021004879 | 0.01562056 | 0.018523533 | 0.021583288 | 0.015950015 | 0.016615259 | 0.018049521 | 0.016224763 | 0.013919997 | 0.01507238 |
| 344 | Cannabinol | 521-35-7 | C07580 | C21H26O2 | 310.19328000000002 | Phenols | 311.2 | 91.1 | + | 11.7538 | 0.000759678 | 0.000539254 | 0.000350423 | 0.000549785 | 0.000646759 | 0.001672065 | 0.000254974 | 0.000857933 | 0.000810359 | 0.000276783 | 0.000543571 |
| 345 | Cantharidin | 56-25-7 | C16778 | C10H12O4 | 196.07355999999999 | Monoterpenoids | 197.1 | 95.1 | + | 6.0265 | 0.00096744 | 0.000967818 | 0.000525599 | 0.000820286 | 0.001318775 | 0.000854919 | 0.000856664 | 0.001010119 | 0.000822831 | 0.000664489 | 0.00074366 |
| 346 | Canthaxanthin | 514-78-3 | C08583 | C40H52O2 | 564.39673000000005 | Prenol lipids | 565.4 | 203.1 | + | 12.82351667 | 0.004498311 | 0.00732834 | 0.002284493 | 0.004703715 | 0.008206866 | 0.003033201 | 0.005205991 | 0.005482019 | 0.002378187 | 0.006087238 | 0.004232713 |
| 347 | Capecitabine | 154361-50-9 | C12650 | C15H22FN3O6 | 359.14926500000001 | Alkaloids | 360.2 | 244.1 | + | 7.871433333 | 3.56915E-06 | 4.47767E-05 | 7.81375E-05 | 4.21611E-05 | 5.49337E-05 | 2.79019E-05 | 1.81487E-05 | 3.36614E-05 | 5.10689E-05 | 4.24681E-05 | 4.67685E-05 |
| 348 | Capsanthin | 465-42-9 | C08584 | C40H56O3 | 584.42294500000003 | Terpene | 585.4 | 549.4 | + | 12.80028333 | 0.000615983 | 0.081287716 | 0.065210497 | 0.049038065 | 0.054330467 | 0.001993914 | 0.070126326 | 0.042150236 | 0.098555255 | 0.074052488 | 0.086303872 |
| 349 | Capsidiol | 37208-05-2 | C09627 | C15H24O2 | 236.17762999999999 | Prenol lipids | 219.2 | 135.1 | + | 9.51615 | 0.000228056 | 0.000220584 | 0.00023817 | 0.000228937 | 0.000699233 | 0.000361991 | 0.000523205 | 0.000528143 | 0.00024827 | 0.000341637 | 0.000294953 |
| 350 | Carbendazim | 10605-21-7 | C10897 | C9H9N3O2 | 191.06947700000001 | Benzimidazoles | 192.1 | 160.1 | + | 4.095283333 | 0.00273585 | 0.002486262 | 0.00240753 | 0.002543214 | 0.004340555 | 0.00308653 | 0.005492514 | 0.004306533 | 0.00237666 | 0.002444953 | 0.002410806 |
| 351 | Carbofuran | 1563-66-2 | C14291 | C12H15NO3 | 221.10519400000001 | Coumarans | 222.1 | 123 | + | 4.025616667 | 2.65319E-05 | 1.59107E-05 | 1.87026E-05 | 2.03817E-05 | 2.37159E-05 | 3.5979E-05 | 6.26699E-05 | 4.07883E-05 | 1.7995E-05 | 3.19269E-05 | 2.49609E-05 |
| 352 | Cardanol (C15:1) | 501-26-8 | C10785 | C21H34O | 302.260965 | Phenols | 303.3 | 107 | + | 12.68811667 | 0.000389775 | 0.006572116 | 0.0044432 | 0.003801697 | 0.006430825 | 0.005171135 | 0.006505328 | 0.006035763 | 0.006549779 | 0.005874454 | 0.006212116 |
| 353 | Carnosic acid | 3650-09-7 | C21818 | C20H28O4 | 332.19875999999999 | Diterpenoids | 331.2 | 287.2 | - | 8.493266667 | 2.01339E-05 | 3.73876E-05 | 3.14027E-05 | 2.96414E-05 | 1.97804E-05 | 2.53914E-05 | 3.72442E-05 | 2.7472E-05 | 2.70464E-05 | 1.71983E-05 | 2.21224E-05 |
| 354 | Carnosol | 5957-80-2 | C09069 | C20H26O4 | 330.18311 | Diterpenoids | 329.2 | 285.2 | - | 12.14696707 | 1.18251E-06 | 1.44753E-05 | 1.58017E-05 | 1.04865E-05 | 4.34535E-05 | 3.0569E-05 | 2.33353E-05 | 3.24526E-05 | 1.1876E-05 | 3.70589E-05 | 2.44675E-05 |
| 356 | Cassiaside B | 119170-51-3 |  | C26H30O14 | 566.16355999999996 | Phenols | 567.2 | 255.1 | + | 13.12446667 | 0.001076095 | 0.000314972 | 0.000238782 | 0.000543283 | 0.000551069 | 0.000288939 | 0.000302862 | 0.000380957 | 0.000546589 | 0.001389441 | 0.000968015 |
| 357 | Cassythicine | 5890-28-8 | C09389 | C19H19NO4 | 325.13140900000002 | Alkaloids | 326.1 | 252.1 | + | 9.903166667 | 3.41803E-05 | 5.11843E-05 | 4.41428E-05 | 4.31691E-05 | 2.62912E-05 | 2.32084E-05 | 1.0546E-05 | 2.00152E-05 | 5.82064E-05 | 2.93151E-05 | 4.37607E-05 |
| 358 | Catalpalactone | 1585-68-8 | C16929 | C15H14O4 | 258.08920999999998 | Sesquiterpenoids | 259.1 | 67.1 | + | 12.83916667 | 0.009222253 | 0.00796113 | 0.00644598 | 0.007876454 | 0.00945454 | 0.007754008 | 0.007269537 | 0.008159362 | 0.007639645 | 0.009374324 | 0.008506984 |
| 359 | Cathinone | 71031-15-7 | C08301 | C9H11NO | 149.08406400000001 | Alkaloids | 150.1 | 117.1 | + | 6.679833333 | 0.000256455 | 0.000199675 | 0.000110434 | 0.000188855 | 0.000162131 | 0.000166837 | 0.000134951 | 0.000154639 | 0.00010047 | 0.000120267 | 0.000110368 |
| 360 | Cedeodarin | 31076-39-8 | C09770 | C16H14O8 | 334.06887 | Flavonoids | 335.1 | 139 | + | 11.73148333 | 0.00038627 | 0.00030189 | 0.000605086 | 0.000431082 | 0.000186906 | 0.000244097 | 0.000546766 | 0.000325923 | 0.000316597 | 0.000199256 | 0.000257926 |
| 361 | Cedrelone;Isomangiferin | 1254-85-9;24699-16-9 | C16979 | C26H30O5;C19H18O11 | 422.20932499999998;422.08491500000002 | Triterpenoids;Xanthones | 423.15 | 405.15 | + | 11.18419167 | 0.000137482 | 0.000184428 | 0.000121231 | 0.000147714 | 0.000169078 | 7.87891E-05 | 0.000136569 | 0.000128146 | 0.000198022 | 6.5715E-05 | 0.000131869 |
| 362 | Cepharanthine | 481-49-2 | C09391 | C37H38N2O6 | 606.27298800000005 | Alkaloids | 607.3 | 576.2 | + | 13.50635 | 1.18251E-06 | 1.85383E-05 | 1.11315E-05 | 1.02841E-05 | 2.16452E-05 | 3.58705E-05 | 1.23455E-06 | 1.95834E-05 | 1.9587E-05 | 2.34836E-05 | 2.15353E-05 |
| 363 | Cevadine | 62-59-9 |  | C32H49NO9 | 591.340734 | Alkaloids | 592.3 | 574.3 | + | 13.27903333 | 0.004025215 | 0.006445722 | 0.005117043 | 0.005195993 | 0.00770171 | 0.006034919 | 0.004606529 | 0.006114386 | 0.005418198 | 0.005261301 | 0.00533975 |
| 364 | Chalconaringenin | 73692-50-9 | C06561 | C15H12O5 | 272.06849999999997 | flavonoids | 272.9 | 152.9 | + | 8.78 | 0.000269391 | 0.000403944 | 0.000352497 | 0.000341944 | 0.000493407 | 0.000352989 | 0.000312469 | 0.000386289 | 0.000350448 | 0.000379202 | 0.000364825 |
| 365 | Chalepensin | 13164-03-9 | C09165 | C16H14O3 | 254.09429499999999 | Coumarins | 255.1 | 69.1 | + | 7.619683333 | 5.39284E-05 | 0.000191886 | 0.000119152 | 0.000121655 | 0.000139791 | 0.000100063 | 0.000182415 | 0.000140756 | 0.000148457 | 0.000136817 | 0.000142637 |
| 366 | Chamazulene | 529-05-5 | C09633 | C14H16 | 184.12520000000001 | lipids | 185.1 | 157.1 | + | 8.741516667 | 0.000314771 | 0.000189942 | 0.000142487 | 0.000215733 | 0.000187341 | 0.000253724 | 0.000158997 | 0.000200021 | 0.000220251 | 0.000285979 | 0.000253115 |
| 367 | Cheilanthifoline | 483-44-3 | C05174 | C19H19NO4 | 325.13140900000002 | Miscellaneous | 326.1 | 268.1 | + | 14.13145 | 1.56336E-05 | 1.61655E-05 | 1.6658E-05 | 1.61524E-05 | 1.42998E-05 | 3.67842E-06 | 1.01094E-05 | 9.36252E-06 | 3.85981E-05 | 1.75297E-05 | 2.80639E-05 |
| 368 | Chelidonine | 476-32-4 | C12242 | C20H19NO5 | 353.12632400000001 | Alkaloids | 354.1 | 275.1 | + | 6.889866667 | 2.47511E-05 | 2.76896E-05 | 3.87788E-05 | 3.04065E-05 | 3.43764E-05 | 3.67617E-05 | 1.23455E-06 | 2.41242E-05 | 0.000100229 | 2.58845E-05 | 6.30568E-05 |
| 369 | Chlorogenic acid | 327-97-9;202650-88-2 | C00852 | C16H18O9 | 354.09508499999998 | Phenylpropanoids | 355.1 | 163 | + | 5.273183333 | 0.00011839 | 0.000140717 | 0.00017192 | 0.000143675 | 9.83347E-05 | 0.000144924 | 9.0921E-05 | 0.000111393 | 0.000266282 | 0.000143856 | 0.000205069 |
| 370 | Chorismate | 617-12-9 | C00251 | C10H10O6 | 226.04774 | Carboxylic acids and derivatives | 225 | 93 | - | 6.69785 | 0.000117215 | 0.000351162 | 0.000108659 | 0.000192345 | 0.000110031 | 0.000301832 | 0.000110065 | 0.000173976 | 0.000556745 | 0.000273877 | 0.000415311 |
| 372 | Chrysophanol;Chrysophanic acid; Chrysophanol | 481-74-3 | C10315 | C15H10O4 | 254.05790999999999 | Anthraquinones | 255.1 | 152.1 | + | 7.456166667 | 5.12346E-05 | 9.52796E-05 | 4.12396E-05 | 6.25846E-05 | 4.265E-05 | 5.23212E-05 | 6.14561E-05 | 5.21424E-05 | 5.66088E-05 | 2.91674E-05 | 4.28881E-05 |
| 373 | Cianidanol | 18829-70-4 | C06562 | C15H14O6 | 290.07900000000001 | flavonoids | 290.9 | 165 | + | 4.5 | 2.13397E-05 | 6.87632E-05 | 3.8281E-05 | 4.27946E-05 | 4.37066E-05 | 2.29502E-05 | 2.87726E-05 | 3.18098E-05 | 3.81551E-05 | 4.27896E-05 | 4.04723E-05 |
| 374 | Cimifugin | 37921-38-3 | C09000 | C16H18O6 | 306.11034000000001 | Flavonoids | 307.1 | 289.1 | + | 4.145616667 | 0.000395214 | 0.000178797 | 0.000163675 | 0.000245896 | 0.000198195 | 0.000204709 | 0.000228887 | 0.000210597 | 0.000146064 | 0.000232243 | 0.000189154 |
| 375 | Cinchonine | 118-10-5 | C06528 | C19H22N2O | 294.17321299999998 | Alkaloids | 295.2 | 79.1 | + | 11.99408333 | 0.003899499 | 0.002040684 | 0.001983483 | 0.002641222 | 0.002929241 | 0.001919433 | 0.001989209 | 0.002279294 | 0.00374723 | 0.002362119 | 0.003054675 |
| 376 | Cinnamaldehyde | 14371-10-9;104-55-2 | C00903 | C9H8O | 132.057515 | Phenylpropanoids | 133.1 | 55 | + | 6.344183333 | 0.000223914 | 0.000183309 | 0.000253961 | 0.000220395 | 0.000183622 | 9.21863E-05 | 0.000195473 | 0.000157094 | 0.000248684 | 0.000208496 | 0.00022859 |
| 377 | Cinnamic acid | 140-10-3 | C00423 | C9H8O2 | 148.05242999999999 | Phenylpropanoids | 149.1 | 131 | + | 2.836566667 | 7.32609E-05 | 0.000166681 | 0.00014981 | 0.000129917 | 0.000274832 | 0.000253053 | 5.05118E-05 | 0.000192799 | 0.0001593 | 0.000112342 | 0.000135821 |
| 378 | Cinnamyl alcohol | 104-54-1 | C02394 | C9H10O | 134.07316499999999 | Phenylpropanoids | 135.1 | 117.1 | + | 10.55668333 | 0.00020415 | 0.000133607 | 0.000315555 | 0.000217771 | 0.000131497 | 0.000129473 | 0.000187441 | 0.00014947 | 0.000287222 | 0.000185366 | 0.000236294 |
| 379 | Cinnamyl cinnamate | 122-69-0 |  | C18H16O2 | 264.11502999999999 | Phenylpropanoids | 265.1 | 117.1 | + | 4.2631 | 0.000117477 | 0.000231426 | 0.000133453 | 0.000160785 | 0.000197198 | 0.000125466 | 0.000159485 | 0.000160716 | 8.05408E-05 | 0.000130274 | 0.000105407 |
| 380 | Cinnzeylanol;Resibufogenin | 62394-04-1;465-39-4 | C17058 | C20H32O7;C24H32O4 | 384.21480500000001;384.23005999999998 | Diterpenoids;Steroids | 385.2 | 367.2 | + | 6.830883333 | 0.000147096 | 0.000158451 | 0.00015208 | 0.000152543 | 0.00014606 | 0.000172791 | 0.00015778 | 0.000158877 | 0.000246905 | 7.93218E-05 | 0.000163113 |
| 381 | cis-2-Methylaconitate | 6061-93-4 | C04225 | C7H8O6 | 188.03209000000001 | Carboxylic acids and derivatives | 189 | 97 | + | 3.8027 | 6.06644E-05 | 0.000103922 | 0.000298183 | 0.000154256 | 0.000137524 | 6.59253E-05 | 3.14811E-05 | 7.83101E-05 | 0.000148139 | 0.000102903 | 0.000125521 |
| 382 | cis-Aconitic acid | 499-12-7;585-84-2 | C00417 | C6H6O6 | 174.01643999999999 | Organic acids and derivatives | 175 | 69 | + | 1.4555 | 0.008424802 | 0.008664236 | 0.006885537 | 0.007991525 | 0.00152783 | 0.005765871 | 0.003126927 | 0.003473543 | 0.001736763 | 0.007604292 | 0.004670528 |
| 383 | cis-Gondoic acid | 5561-99-9 | C16526 | C20H38O2 | 310.28717999999998 | Lipids | 311.3 | 57.1 | + | 13.4027 | 0.013054873 | 0.008292386 | 0.003670149 | 0.008339136 | 0.01238084 | 0.010438538 | 0.014622184 | 0.012480521 | 0.006297635 | 0.009271676 | 0.007784656 |
| 384 | cis-Zeatin | 32771-64-5 | C15545 | C10H13N5O | 219.11199999999999 | phytohormone | 220 | 136 | + | 3.6 | 0.000141343 | 0.000146358 | 8.87338E-05 | 0.000125478 | 5.09206E-05 | 0.000160221 | 9.91844E-05 | 0.000103442 | 0.000112617 | 0.000119474 | 0.000116046 |
| 385 | Citrostadienol | 474-40-8 | C11523 | C30H50O | 426.38616500000001 | Steroids | 427.4 | 409.4 | + | 12.28533333 | 0.000271469 | 0.000591648 | 0.000410524 | 0.000424547 | 0.00014389 | 0.000191447 | 0.000399083 | 0.000244807 | 0.000377358 | 0.00021933 | 0.000298344 |
| 386 | Cleomiscosin A | 76948-72-6 | C09922 | C20H18O8 | 386.10016999999999 | Coumarins | 387.1 | 137.1 | + | 5.74 | 0.003421179 | 0.003685893 | 0.002571001 | 0.003226024 | 0.00222084 | 0.003169805 | 0.002116786 | 0.002502477 | 0.003548604 | 0.002987607 | 0.003268106 |
| 387 | Clivorine | 33979-15-6 | C10282 | C21H27NO7 | 405.17875400000003 | Alkaloids | 406.2 | 346.2 | + | 7.85465 | 7.31496E-05 | 6.15344E-05 | 1.99832E-05 | 5.15557E-05 | 3.11929E-05 | 2.57903E-05 | 2.42819E-05 | 2.70884E-05 | 3.12672E-05 | 7.15164E-05 | 5.13918E-05 |
| 388 | Coclaurine | 486-39-5 | C06161 | C17H19NO3 | 285.13649400000003 | Alkaloids | 286.1 | 107 | + | 9.063016667 | 9.57714E-05 | 4.6852E-05 | 4.73142E-05 | 6.33125E-05 | 4.40389E-05 | 0.000116548 | 9.82398E-05 | 8.62755E-05 | 0.000114137 | 0.000137776 | 0.000125956 |
| 389 | Colneleate | 52761-34-9 | C19827 | C18H30O3 | 294.21949499999999 | Fatty Acyls | 295.2 | 123.1 | + | 11.96606667 | 0.000521818 | 0.00065284 | 0.000548293 | 0.000574317 | 0.000592244 | 0.000620824 | 0.000456822 | 0.00055663 | 0.000535144 | 0.000752581 | 0.000643862 |
| 391 | Coniferylaldehyde | 458-36-6 | C02666 | C10H10O3 | 178.062995 | Phenylpropanoids | 179.1 | 91.1 | + | 6.780533333 | 0.008260377 | 0.012283852 | 0.008969008 | 0.009837746 | 0.013340464 | 0.011273167 | 0.009385366 | 0.011332999 | 0.01149185 | 0.01180552 | 0.011648685 |
| 393 | Coproporphyrin III | 14643-66-4 | C05770 | C36H38N4O8 | 654.26896599999998 | Tetrapyrroles and derivatives | 655.3 | 619.3 | + | 11.84896667 | 0.001716045 | 0.002078937 | 0.001834583 | 0.001876521 | 0.003333105 | 0.003736263 | 0.002294352 | 0.00312124 | 0.00211452 | 0.002833124 | 0.002473822 |
| 394 | Cordycepin | 73-03-0 | C08431 | C10H13N5O3 | 251.10184000000001 | Nucleotide and its derivates | 252.1 | 136.1 | + | 2.34785 | 0.006744277 | 0.008258135 | 0.006998963 | 0.007333791 | 0.011149342 | 0.010562858 | 0.009948126 | 0.010553442 | 0.007770974 | 0.007344449 | 0.007557712 |
| 395 | Corticosterone | 50-22-6 | C02140 | C21H30O4 | 346.21440999999999 | Steroids and steroid derivatives | 347.2 | 329.2 | + | 6.110733333 | 0.004840068 | 0.005632113 | 0.006560079 | 0.00567742 | 0.0123255 | 0.006635053 | 0.006305859 | 0.008422137 | 0.004383987 | 0.00418343 | 0.004283708 |
| 396 | Cortolone-3-glucuronide | 56162-46-0 | C03033 | C27H42O11 | 542.27271499999995 | Organooxygen compounds | 543.3 | 349.2 | + | 12.30211667 | 0.001555348 | 0.001946643 | 0.000873935 | 0.001458642 | 0.000989934 | 0.001367957 | 0.001331638 | 0.001229843 | 0.001258663 | 0.001877104 | 0.001567883 |
| 397 | Costunolide | 553-21-9 | C09382 | C15H20O2 | 232.14633000000001 | Sesquiterpenoids | 233.2 | 161.1 | + | 12.2077303 | 0.000148183 | 0.000132611 | 0.000687464 | 0.000322752 | 0.000539731 | 0.000130198 | 6.2465E-05 | 0.000244131 | 0.000111267 | 0.000217004 | 0.000164136 |
| 398 | Crategolic acid | 4373-41-5 | C16939 | C30H48O4 | 472.35525999999999 | Triterpenoids | 473.4 | 409.3 | + | 12.47787568 | 4.55277E-05 | 0.000124057 | 8.42488E-06 | 5.93364E-05 | 1.59752E-06 | 1.26522E-06 | 0.000148492 | 5.04517E-05 | 9.14609E-05 | 4.42363E-05 | 6.78486E-05 |
| 399 | Crotaline | 315-22-0 | C10350 | C16H23NO6 | 325.15253899999999 | Alkaloids | 326.2 | 120.1 | + | 11.42833333 | 0.003104024 | 0.003230282 | 0.003632312 | 0.003322206 | 1.59752E-06 | 1.26522E-06 | 1.23455E-06 | 1.36576E-06 | 0.003551121 | 0.003666731 | 0.003608926 |
| 400 | Crotonoside | 1818-71-9 | C08432 | C10H13N5O5 | 283.09167000000002 | Alkaloids | 284.1 | 152.1 | + | 2.39745 | 0.15264767 | 0.119423401 | 0.128625352 | 0.133565474 | 0.014841578 | 0.110280263 | 0.019857225 | 0.048326356 | 0.138370807 | 0.121625777 | 0.129998292 |
| 401 | Cucurbitacin A | 6040-19-3 | C08793 | C32H46O9 | 574.31418499999995 | Triterpenoids | 575.3 | 539.3 | + | 11.19445 | 0.00038264 | 7.06852E-05 | 0.000346321 | 0.000266549 | 0.000161182 | 0.000346478 | 0.000249141 | 0.000252267 | 0.000777655 | 0.000142789 | 0.000460222 |
| 403 | Cucurbitacin I;Ganoderenic acid A;Ganoderic acid C1;Ganoderenic acid B;Ganoderic acid Z | 2222-07-3;100665-40-5;95311-97-0;100665-41-6;294674-09-2 | C08800 | C30H42O7 | 514.29305499999998 | Triterpenoids | 515.3 | 479.3 | + | 12.51789 | 0.000332823 | 0.001711856 | 0.000823733 | 0.000956138 | 0.000937155 | 0.000648537 | 0.000463305 | 0.000682999 | 0.001404434 | 0.000472858 | 0.000938646 |
| 404 | Cupressuflavone | 3952-18-9 | C10034 | C30H18O10 | 538.09 | Flavonoids | 539.1 | 521.1 | + | 9.6672 | 0.038041211 | 0.027324795 | 0.032049417 | 0.032471808 | 0.025869527 | 0.013298234 | 0.021396085 | 0.020187949 | 0.035920479 | 0.026579544 | 0.031250012 |
| 406 | Curcumin | 458-37-7 | C10443 | C21H20O6 | 368.12599 | Phenols | 369.1 | 177.1 | + | 5.688233333 | 4.75852E-05 | 0.000108406 | 0.000189294 | 0.000115095 | 0.00047091 | 0.000129546 | 9.16566E-05 | 0.000230704 | 0.000132269 | 0.000143831 | 0.00013805 |
| 407 | Curcumol | 4871-97-0 |  | C15H24O2 | 236.17762999999999 | Sesquiterpenoids | 237.2 | 57.1 | + | 11.14408333 | 0.000216339 | 0.000155506 | 9.35008E-05 | 0.000155115 | 0.000302434 | 0.00020941 | 0.000175929 | 0.000229258 | 0.000152959 | 8.19335E-05 | 0.000117446 |
| 408 | Curzerene | 17910-09-7 |  | C15H20O | 216.15141499999999 | Sesquiterpenoids | 217.2 | 55.1 | + | 2.546233333 | 0.001901418 | 0.001600296 | 0.001138474 | 0.001546729 | 0.002215843 | 0.001463799 | 0.002387244 | 0.002022295 | 0.001704184 | 0.001266962 | 0.001485573 |
| 409 | Cyanidin | 528-58-5 | C05905 | C15H10O6 | 286.04773999999998 | Flavonoids | 287.1 | 137 | + | 5.898142 | 0.021963168 | 0.022707393 | 0.024277111 | 0.022982557 | 0.035222641 | 0.027079286 | 0.031800475 | 0.031367467 | 0.031749086 | 0.023879674 | 0.02781438 |
| 410 | Cyanidin-3-O-rhamnoside chloride | 38533-30-1 |  | C21H21O10 | 433.11347499999999 | Flavonoids | 434.1 | 271.1 | + | 7.3241 | 6.57732E-05 | 2.9339E-05 | 2.05176E-05 | 3.85433E-05 | 1.82744E-05 | 1.4305E-05 | 1.06089E-05 | 1.43961E-05 | 5.09595E-05 | 2.49602E-05 | 3.79599E-05 |
| 411 | Cycleanine | 518-94-5 | C17387 | C38H42N2O6 | 622.30428800000004 | Alkaloids | 623.3 | 578.3 | + | 11.51331667 | 6.97647E-05 | 4.21856E-05 | 1.12648E-06 | 3.76923E-05 | 0.000198018 | 0.000149797 | 1.23455E-06 | 0.00011635 | 0.000151779 | 3.07251E-05 | 9.12521E-05 |
| 412 | Cycloeucalenol | 469-39-6 | C02141 | C30H50O | 426.38616500000001 | Triterpenoids | 427.4 | 83.1 | + | 11.89931667 | 0.000184827 | 0.000169802 | 0.000184055 | 0.000179561 | 0.000278749 | 0.000116624 | 0.000258702 | 0.000218025 | 0.00036553 | 0.000419199 | 0.000392365 |
| 414 | Cynaroside | 5373-11-5 |  | C21H20O11 | 448.10056100000003 | flavonoids | 449 | 286.9 | + | 6.1 | 0.001160598 | 0.003234383 | 0.003348863 | 0.002581281 | 0.005364072 | 0.002565072 | 0.002958525 | 0.003629223 | 0.001201324 | 0.001534104 | 0.001367714 |
| 415 | CYS-GLY;Cysteinylglycine | 19246-18-5 | C01419 | C5H10N2O3S | 178.041214 | Amino acid and derivatives | 179 | 76 | + | 1.38275 | 0.00084035 | 0.001351866 | 0.001125676 | 0.001105964 | 0.000908823 | 0.000658654 | 0.000532895 | 0.000700124 | 0.001517209 | 0.001091217 | 0.001304213 |
| 416 | Cytidine | 65-46-3 | C00475 | C9H13N3O5 | 243.085522 | Nucleotide and its derivates | 244.1 | 112.1 | + | 0.704328709 | 0.000628489 | 0.000712133 | 0.000526026 | 0.000622216 | 0.0011794 | 0.000610367 | 0.000633462 | 0.000807743 | 0.000599298 | 0.000553728 | 0.000576513 |
| 417 | Cytisine | 485-35-8 | C10763 | C11H14N2O | 190.110613 | Alkaloids | 191.1 | 44.1 | + | 1.21823458 | 1.93046E-05 | 2.39325E-05 | 5.49665E-05 | 3.27346E-05 | 5.34357E-05 | 8.81737E-05 | 4.02433E-05 | 6.06176E-05 | 2.64163E-05 | 7.86615E-05 | 5.25389E-05 |
| 420 | D-alpha-Aminobutyric acid | 2623-91-8 | C02261 | C4H9NO2 | 103.063329 | Carboxylic acids and derivatives | 104.1 | 58.1 | + | 0.648333333 | 0.540654932 | 0.684892598 | 0.514875912 | 0.580141147 | 0.678454162 | 0.649470528 | 0.604493706 | 0.644139465 | 0.542477298 | 0.485395938 | 0.513936618 |
| 421 | D-Aspartic acid | 1783-96-6 | C00402 | C4H7NO4 | 133.037509 | Alkaloids | 134 | 74 | + | 0.755483333 | 0.001143208 | 0.000608344 | 0.001165654 | 0.000972402 | 0.001100325 | 0.000898468 | 0.00117911 | 0.001059301 | 0.000495575 | 0.000803564 | 0.000649569 |
| 423 | D-Glucose 6-phosphate;Glucose 6-phosphate | 56-73-5 | C00092 | C6H13O9P | 260.02972199999999 | Carbohydrates | 259 | 97 | - | 0.6463 | 0.000163646 | 0.000105977 | 7.44862E-05 | 0.000114703 | 0.000446128 | 0.000491596 | 0.000199129 | 0.000378951 | 0.00019542 | 0.000258471 | 0.000226946 |
| 424 | D-Glutamine | 5959-95-5 | C00819 | C5H10N2O3 | 146.069143 | Carboxylic acids and derivatives | 147.1 | 84 | + | 0.658483333 | 0.056735084 | 0.055211896 | 0.066858713 | 0.059601898 | 0.147326026 | 0.087059499 | 0.092189308 | 0.108858278 | 0.099472557 | 0.081517331 | 0.090494944 |
| 425 | D-Maltose | 69-79-4 | C00208 | C12H22O11 | 342.11621500000001 | Carbohydrates | 341.1 | 59 | - | 0.764066667 | 0.000325812 | 0.000775198 | 0.000516793 | 0.000539267 | 0.002006295 | 0.001416358 | 0.001119952 | 0.001514202 | 0.000936563 | 0.001270346 | 0.001103455 |
| 427 | D-Pinitol | 10284-63-6 | C03844 | C7H14O6 | 194.07903999999999 | Miscellaneous | 195.1 | 177.1 | + | 0.617181539 | 8.66941E-05 | 9.31568E-05 | 9.33218E-05 | 9.10576E-05 | 0.000211969 | 9.00536E-05 | 9.32662E-05 | 0.000131763 | 0.00016361 | 8.96216E-05 | 0.000126616 |
| 428 | D-Proline | 344-25-2 | C00763 | C5H9NO2 | 115.063329 | Carboxylic acids and derivatives | 116.1 | 70.1 | + | 0.755483333 | 0.085511244 | 0.110738114 | 0.091153827 | 0.095801062 | 0.12822575 | 0.107252023 | 0.0974512 | 0.110976325 | 0.106280669 | 0.099763498 | 0.103022083 |
| 429 | D-Serine | 312-84-5 | C00740 | C3H7NO3 | 105.04259399999999 | Alkaloids | 106 | 60 | + | 0.655383333 | 0.00388549 | 0.004974301 | 0.004703453 | 0.004521081 | 0.006127258 | 0.002786749 | 0.00319362 | 0.004035876 | 0.003828622 | 0.004317443 | 0.004073033 |
| 430 | D-Urobilin | 3947-38-4 | C05795 | C33H40N4O6 | 588.29478600000004 | Tetrapyrroles and derivatives | 589.3 | 553.3 | + | 11.52333333 | 0.00022525 | 0.000574135 | 0.000656154 | 0.000485179 | 0.000617059 | 1.26522E-06 | 0.000355896 | 0.00032474 | 0.000823826 | 0.000470739 | 0.000647283 |
| 431 | D-Xylulose | 551-84-8 | C00310 | C5H10O5 | 150.05282500000001 | Carbohydrates | 133.1 | 43 | + | 1.76245 | 0.001924454 | 0.002002412 | 0.001838803 | 0.00192189 | 0.001574719 | 0.001183006 | 0.001132687 | 0.001296804 | 0.001744439 | 0.001740228 | 0.001742333 |
| 432 | Daidzein | 486-66-8 | C10208 | C15H10O4 | 254.05790999999999 | Flavonoids | 255.1 | 91.1 | + | 8.500016667 | 0.00016635 | 0.000866324 | 9.16806E-05 | 0.000374785 | 0.000855071 | 0.000396316 | 0.000837536 | 0.000696308 | 0.000703591 | 0.000229204 | 0.000466397 |
| 433 | Daidzein-4',7-diglucoside | 53681-67-7 |  | C27H30O14 | 578.16355999999996 | Flavonoids | 579.2 | 399.1 | + | 5.94415 | 0.00015403 | 8.57507E-05 | 0.000120243 | 0.000120008 | 0.000140557 | 7.4581E-05 | 8.52114E-05 | 0.000100116 | 0.000110375 | 0.00023452 | 0.000172447 |
| 434 | Daidzin | 552-66-9 | C10216 | C21H20O9 | 416.11070000000001 | flavonoids | 416.9 | 255 | + | 5.45 | 0.000396262 | 0.000189026 | 0.000272538 | 0.000285942 | 0.00028797 | 0.000496026 | 0.000403421 | 0.000395806 | 7.4988E-05 | 0.000231634 | 0.000153311 |
| 435 | Dalbergin | 482-83-7 | C10414 | C16H12O4 | 268.07355999999999 | Coumarins | 269.1 | 241.1 | + | 9.861833333 | 5.33918E-05 | 5.05042E-05 | 3.75417E-05 | 4.71459E-05 | 4.89972E-05 | 0.000178204 | 3.03443E-05 | 8.58484E-05 | 4.91554E-05 | 7.34554E-05 | 6.13054E-05 |
| 436 | Dalbergioidin | 30368-42-4 | C10415 | C15H12O6 | 288.06339000000003 | Flavonoids | 289.1 | 153 | + | 5.99175 | 0.001033561 | 0.001124484 | 0.000334402 | 0.000830816 | 0.001903941 | 0.00088096 | 0.002214796 | 0.001666565 | 0.001002686 | 0.001082892 | 0.001042789 |
| 437 | Daphnoretin | 2034-69-7 | C09216 | C19H12O7 | 352.05830500000002 | Coumarins | 353.1 | 163 | + | 11.44618333 | 0.024581381 | 0.038368669 | 0.028821741 | 0.030590597 | 0.030967797 | 0.017213319 | 0.024415288 | 0.024198801 | 0.029891423 | 0.042255986 | 0.036073704 |
| 439 | Decursin;5-Geranoxy-7-methoxycoumarin;Decursinol angelate | 5928-25-6;7380-39-4;130848-06-5 | C09258 | C19H20O5;C20H24O4 | 328.13107500000001;328.16746000000001 | Coumarins | 329.1333333 | 175 | + | 13.77691667 | 0.000158172 | 8.47663E-05 | 0.00013551 | 0.00012615 | 0.00012354 | 0.000121598 | 0.000128595 | 0.000124578 | 0.000119355 | 0.000227883 | 0.000173619 |
| 440 | Decursinol | 23458-02-8 | C09259 | C14H14O4 | 246.08921000000001 | Coumarins | 247.1 | 175 | + | 7.9889 | 0.0007034 | 0.000685065 | 0.000507298 | 0.000631921 | 0.00021553 | 0.000101292 | 0.000296614 | 0.000204478 | 0.000227975 | 0.00041844 | 0.000323208 |
| 441 | Deethylatrazine | 6190-65-4 | C06559 | C6H10ClN5 | 187.06247300000001 | Triazines | 188.1 | 146 | + | 3.809966667 | 0.044527509 | 0.043540492 | 0.040818796 | 0.042962266 | 0.042565054 | 0.039396851 | 0.041906457 | 0.041289454 | 0.041185276 | 0.0357564 | 0.038470838 |
| 442 | Deguelin | 522-17-8 | C10417 | C23H22O6 | 394.14164 | Flavonoids | 395.1 | 147 | + | 13.46013333 | 0.000414433 | 0.000591086 | 0.000519147 | 0.000508222 | 0.000482935 | 0.000380328 | 0.000401313 | 0.000421525 | 0.000253895 | 0.000587495 | 0.000420695 |
| 443 | Dehydronuciferine | 7630-74-2 |  | C19H19NO2 | 293.14157899999998 | Alkaloids | 294.1 | 206.1 | + | 5.656083333 | 6.6756E-05 | 0.000147739 | 6.50487E-05 | 9.31814E-05 | 6.06099E-05 | 9.42084E-05 | 0.000286473 | 0.000147097 | 5.01028E-05 | 0.000219783 | 0.000134943 |
| 444 | Delphinidin | 528-53-0 | C05908 | C15H10O7 | 302.04265500000002 | Flavonoids | 303 | 229 | + | 6.858283333 | 0.000566223 | 0.000122521 | 0.000421125 | 0.000369956 | 0.000535891 | 0.000542361 | 0.000296905 | 0.000458386 | 0.000525169 | 0.000546713 | 0.000535941 |
| 445 | Delphinidin-3-O-glucoside;Isoquercitrin | 6906-38-3;50986-17-9;482-35-9 | C12138 | C21H20O12 | 464.09548000000001 | Flavonoids | 465.1 | 303.1 | + | 6.063816667 | 0.000552441 | 0.000970994 | 0.00328575 | 0.001603062 | 0.001151534 | 0.000918118 | 0.000936984 | 0.001002212 | 0.000779742 | 0.000377479 | 0.000578611 |
| 446 | Delta-Nonalactone | 3301-94-8 |  | C9H16O2 | 156.11502999999999 | Miscellaneous | 157.1 | 57.1 | + | 0.529283333 | 0.004500768 | 0.007735732 | 0.005898647 | 0.006045049 | 0.009477409 | 0.00695382 | 0.004991494 | 0.007140908 | 0.005910695 | 0.006270142 | 0.006090418 |
| 447 | Delta-Tocotrienol | 25612-59-3 | C14156 | C27H40O2 | 396.30282999999997 | Phenols | 395.3 | 135 | - | 11.501 | 4.14269E-05 | 1.27649E-06 | 1.43815E-05 | 1.90283E-05 | 1.59752E-06 | 1.26522E-06 | 1.23455E-06 | 1.36576E-06 | 4.16064E-05 | 3.01233E-05 | 3.58649E-05 |
| 448 | Deltaline | 3836-11-9;6836-11-9 | C08679 | C27H41NO8 | 507.28321899999997 | Alkaloids | 508.3 | 476.3 | + | 7.550566667 | 6.64349E-05 | 4.78031E-05 | 2.37482E-05 | 4.59954E-05 | 4.46295E-05 | 2.85221E-05 | 4.39572E-05 | 3.90363E-05 | 3.12077E-05 | 3.26251E-05 | 3.19164E-05 |
| 449 | Demethoxycapillarisin | 61854-36-2 | C17786 | C15H10O6 | 286.04773999999998 | Flavonoids | 287.1 | 269 | + | 6.512016667 | 0.000601948 | 0.000257528 | 0.000276665 | 0.000378713 | 0.000197152 | 0.000253228 | 0.000402283 | 0.000284221 | 0.000399127 | 0.000213967 | 0.000306547 |
| 450 | Demethoxyencecalin | 19013-07-1 |  | C13H14O2 | 202.09938 | Phenols | 203.1 | 119 | + | 11.79861667 | 0.00043736 | 0.000369822 | 0.000457074 | 0.000421419 | 0.000630958 | 0.000219038 | 0.000244468 | 0.000364821 | 0.000424184 | 0.00033304 | 0.000378612 |
| 451 | Denudatine | 26166-37-0 | C08680 | C22H33NO2 | 343.25112899999999 | Alkaloids | 344.3 | 326.2 | + | 1.587366667 | 0.004785653 | 0.006055342 | 0.004351599 | 0.005064198 | 0.007095513 | 0.008377174 | 0.00808361 | 0.007852099 | 0.006995823 | 0.007200008 | 0.007097916 |
| 452 | Deoxycorticosterone | 64-85-7 | C03205 | C21H30O3 | 330.21949499999999 | Steroids and steroid derivatives | 331.2 | 97.1 | + | 9.605566667 | 4.53503E-05 | 5.22716E-05 | 6.30714E-05 | 5.35644E-05 | 0.000113122 | 5.45818E-05 | 7.7534E-05 | 8.1746E-05 | 4.66215E-05 | 2.6802E-05 | 3.67118E-05 |
| 453 | Deoxyelephantopin | 29307-03-7 | C09388 | C19H20O6 | 344.12599 | Sesquiterpenoids | 345.1 | 69 | + | 2.663366667 | 1.49452E-05 | 5.86312E-05 | 4.95138E-05 | 4.10301E-05 | 4.49199E-05 | 5.04527E-05 | 2.35919E-05 | 3.96548E-05 | 2.69594E-05 | 0.000105132 | 6.60458E-05 |
| 455 | Deoxylapachol | 3568-90-9 | C10325 | C15H14O2 | 226.09938 | Quinones | 227.1 | 55.1 | + | 4.934416667 | 0.000218287 | 0.000292759 | 0.00014329 | 0.000218112 | 0.000273806 | 0.000139263 | 0.000203625 | 0.000205565 | 0.000212182 | 0.000324067 | 0.000268124 |
| 456 | Dephospho-CoA | 3633-59-8 | C00882 | C21H35N7O13P2S | 687.14888299999996 | Purine nucleotides | 688.2 | 261.1 | + | 6.849883333 | 0.000111101 | 9.97932E-05 | 0.000107311 | 0.000106068 | 7.98078E-05 | 9.1088E-05 | 0.000106569 | 9.24882E-05 | 0.000128881 | 3.32323E-05 | 8.10565E-05 |
| 457 | Desmethylbellidifolin | 2980-32-7 | C10056 | C13H8O6 | 260.03208999999998 | Xanthones | 261 | 243 | + | 9.969283333 | 9.96518E-05 | 0.000174552 | 0.000112345 | 0.00012885 | 9.33681E-05 | 9.98486E-05 | 2.17223E-05 | 7.16464E-05 | 7.84002E-05 | 0.000166083 | 0.000122242 |
| 458 | Desoxypeganine | 495-59-0 | C10656 | C11H12N2 | 172.10004799999999 | Alkaloids | 173.1 | 144.1 | + | 8.3764 | 0.000157738 | 8.69786E-05 | 8.66756E-05 | 0.000110464 | 0.000442552 | 7.2843E-05 | 0.000173826 | 0.00022974 | 0.00015066 | 0.000454704 | 0.000302682 |
| 459 | Dexamethasone | 50-02-2 | C06945;C15643 | C22H29FO5 | 392.19990300000001 | Steroids and steroid derivatives | 393.2 | 147.1 | + | 12.00001667 | 0.000461925 | 0.000602811 | 0.000276801 | 0.000447179 | 0.002916176 | 0.000510999 | 0.000449707 | 0.001292294 | 0.002500801 | 0.000907548 | 0.001704175 |
| 460 | DG(16:0/16:0/0:0) | 30334-71-5 | C00165 | C35H68O5 | 568.50667499999997 | Glycerolipids | 591.5 | 313.3 | + | 13.42095 | 0.005530233 | 0.011934634 | 0.013027906 | 0.010164258 | 0.012509935 | 0.013537296 | 0.011182397 | 0.012409876 | 0.016241006 | 0.011913717 | 0.014077361 |
| 461 | Dhurrin | 499-20-7 | C05143 | C14H17NO7 | 311.100504 | Alkaloids | 334.1 | 123 | + | 13.32586667 | 0.011141312 | 0.007913609 | 0.008184882 | 0.009079934 | 0.00724603 | 0.006003895 | 0.005946328 | 0.006398751 | 0.009984488 | 0.010547342 | 0.010265915 |
| 462 | Diallyl disulfide | 2179-57-9 | C08369 | C6H10S2 | 146.022392 | Miscellaneous | 147 | 41 | + | 0.678083333 | 0.003381273 | 0.003548229 | 0.003439477 | 0.003456326 | 0.008835268 | 0.008527112 | 0.006976003 | 0.008112794 | 0.007652825 | 0.006710183 | 0.007181504 |
| 463 | Dictamnine | 484-29-7 | C10660 | C12H9NO2 | 199.06332900000001 | Alkaloids | 200.1 | 129.1 | + | 9.897766667 | 0.000301471 | 0.000197357 | 0.000237962 | 0.000245597 | 0.000142256 | 0.000308109 | 0.000335013 | 0.000261793 | 0.00035657 | 0.00026557 | 0.00031107 |
| 465 | Dihydrojasmonic Acid | 3572-64-3 |  | C12H20O3 | 212.141244 | phytohormone | 211 | 59 | - | 9.82 | 1.18251E-06 | 6.16477E-05 | 4.14759E-05 | 3.47687E-05 | 3.07404E-05 | 5.55105E-05 | 3.81424E-05 | 4.14644E-05 | 3.55551E-05 | 3.81566E-05 | 3.68559E-05 |
| 466 | Dihydromethysticin | 19902-91-1 | C09926 | C15H16O5 | 276.09977500000002 | Phenols | 277.1 | 135 | + | 11.96645 | 0.000915216 | 0.002606312 | 0.000734005 | 0.001418511 | 0.000615553 | 0.003022472 | 0.001116942 | 0.001584989 | 0.00077806 | 0.002202612 | 0.001490336 |
| 467 | Dihydromyricetin | 27200-12-0 | C02906 | C15H12O8 | 320.0532 | flavonoids | 320.9 | 302.8 | + | 5.27 | 0.000123873 | 0.007042715 | 0.000106968 | 0.002424519 | 0.001504871 | 0.000306094 | 0.000200366 | 0.000670444 | 0.007129212 | 0.006241945 | 0.006685578 |
| 468 | Dihydrorobinetin | 4382-33-6 |  | C15H12O7 | 304.05830500000002 | Flavonoids | 305.1 | 137 | + | 5.188033333 | 6.18925E-05 | 0.000108685 | 0.000113048 | 9.45416E-05 | 7.30261E-05 | 6.28169E-05 | 0.000118647 | 8.48301E-05 | 0.000182165 | 9.57588E-05 | 0.000138962 |
| 469 | Dihydrotestosterone | 521-18-6 | C03917 | C19H30O2 | 290.22458 | Steroids and steroid derivatives | 291.2 | 273.2 | + | 5.083358333 | 3.99512E-05 | 0.000129757 | 7.86481E-05 | 8.27854E-05 | 0.000123494 | 0.000100513 | 5.36931E-05 | 9.25664E-05 | 6.29726E-05 | 0.000205421 | 0.000134197 |
| 470 | Dihydrozeatin | 23599-75-9 | C02029 | C10H15N5O | 221.12765999999999 | Imidazopyrimidines | 222.1 | 204.1 | + | 4.296666667 | 5.01076E-05 | 2.69494E-05 | 7.60283E-05 | 5.10284E-05 | 9.19196E-05 | 7.53642E-05 | 0.00014266 | 0.000103315 | 0.000118805 | 0.000107023 | 0.000112914 |
| 471 | Dimethylbenzimidazole | 582-60-5 | C03114 | C9H10N2 | 146.08439799999999 | Benzimidazoles | 147.1 | 131.1 | + | 10.5638 | 0.000227036 | 0.000163221 | 0.000224687 | 0.000204981 | 0.00023294 | 8.48987E-05 | 0.000269302 | 0.000195714 | 0.000128628 | 0.000159545 | 0.000144087 |
| 473 | Diosmetin | 520-34-3 | C10038 | C16H12O6 | 300.0634 | flavonoids | 301.3 | 285.8 | + | 9.15 | 0.000138857 | 0.000206496 | 0.000120915 | 0.000155423 | 8.10481E-05 | 3.14717E-05 | 8.82449E-05 | 6.69216E-05 | 0.000139678 | 7.86671E-05 | 0.000109173 |
| 474 | Diosmin | 520-27-4 | C10039 | C28H32O15 | 608.17409999999995 | flavonoids | 609.1 | 463 | + | 6.59 | 0.000164995 | 0.000198718 | 0.000321866 | 0.000228526 | 0.000127019 | 2.0448E-05 | 0.000214339 | 0.000120602 | 0.000267188 | 0.000153995 | 0.000210591 |
| 475 | Diphyllin | 22055-22-7 | C10559 | C21H16O7 | 380.08960500000001 | Lignans | 381.1 | 335.1 | + | 10.08676667 | 3.23712E-05 | 5.80097E-05 | 6.73612E-05 | 5.25807E-05 | 3.03225E-05 | 2.3317E-05 | 2.60896E-05 | 2.65764E-05 | 7.70181E-05 | 4.3666E-05 | 6.03421E-05 |
| 476 | Dipterocarpol | 471-69-2 | C08622 | C30H50O2 | 442.38108 | Triterpenoids | 443.4 | 425.4 | + | 12.05236667 | 0.000269572 | 0.000311235 | 0.000104516 | 0.000228441 | 0.000573049 | 0.000354064 | 0.000230718 | 0.000385944 | 0.000536442 | 0.00035474 | 0.000445591 |
| 477 | DL-Alanine;L-Alanine | 302-72-7;56-41-7 | C01401;C00041 | C3H7NO2 | 89.047679000000002;89.047700000000006 | Alkaloids;Amino acid and derivatives;amino acids | 90.1 | 44 | + | 0.644927778 | 0.003522913 | 0.003184601 | 0.003840262 | 0.003515926 | 0.003554857 | 0.003971694 | 0.00241949 | 0.003315347 | 0.002933984 | 0.003546074 | 0.003240029 |
| 478 | DL-Benzylsuccinic acid |  | C09816 | C11H12O4 | 208.07355999999999 | Phenylpropanoids | 209.1 | 117.1 | + | 4.9512 | 0.000876692 | 0.000782896 | 0.001021244 | 0.000893611 | 0.001070335 | 0.000900966 | 0.001110293 | 0.001027198 | 0.0003555 | 0.000613208 | 0.000484354 |
| 479 | DL-Dihydrozeatin | 14894-18-9 | C02029 | C10H15N5O | 221.1277 | phytohormone | 222 | 136 | + | 3.52 | 7.92361E-05 | 0.000204969 | 6.64988E-05 | 0.000116901 | 0.000257797 | 7.86771E-05 | 0.000112875 | 0.000149783 | 0.000120424 | 0.000134777 | 0.0001276 |
| 480 | DL-Norvaline | 6600-40-4 | C01826 | C5H11NO2 | 117.078979 | Amino acid and derivatives | 118.1 | 72.1 | + | 1.05125 | 0.013807965 | 0.013010408 | 0.019420891 | 0.015413088 | 0.025605889 | 0.011491678 | 0.018150839 | 0.018416135 | 0.017008351 | 0.013317732 | 0.015163042 |
| 481 | Docosahexaenoic acid | 6217-54-5 | C06429 | C22H32O2 | 328.24023 | Fatty Acyls | 329.2 | 93.1 | + | 12.60253333 | 0.000340287 | 0.000392894 | 0.000307629 | 0.000346937 | 0.000773086 | 0.000527344 | 0.000914411 | 0.00073828 | 0.001812103 | 0.000962492 | 0.001387297 |
| 482 | Dopamine glucuronide | 38632-24-5 | C03033 | C14H19NO8 | 329.11106899999999 | Organooxygen compounds | 330.1 | 154.1 | + | 5.20295 | 4.96276E-05 | 3.37574E-05 | 2.99783E-05 | 3.77877E-05 | 3.23019E-05 | 9.10121E-05 | 1.23455E-06 | 4.15162E-05 | 4.86367E-05 | 0.000109517 | 7.90769E-05 |
| 483 | Dulcoside A | 64432-06-0 |  | C38H60O17 | 788.38305500000001 | Diterpenoids | 811.4 | 649.3 | + | 7.535766667 | 0.000185062 | 7.38598E-05 | 0.000165526 | 0.000141483 | 0.000125668 | 0.000126773 | 0.000166108 | 0.000139516 | 0.000281784 | 8.50839E-05 | 0.000183434 |
| 484 | dUMP | 964-26-1 | C00365 | C9H13N2O8P | 308.040955 | Pyrimidine nucleotides | 309 | 113 | + | 0.760366667 | 0.000382954 | 0.000387508 | 0.00050305 | 0.000424504 | 0.000436123 | 0.00056838 | 0.000480716 | 0.000495073 | 0.000228718 | 0.000242112 | 0.000235415 |
| 485 | Eicosapentaenoic acid | 10417-94-4 | C06428 | C20H30O2 | 302.22458 | Fatty Acyls | 303.2 | 285.2 | + | 6.86445 | 0.000238477 | 0.000182601 | 0.000284221 | 0.0002351 | 0.000229127 | 0.000213494 | 0.000155622 | 0.000199415 | 0.000173746 | 0.000171671 | 0.000172708 |
| 486 | Embelin | 550-24-3 | C10342 | C17H26O4 | 294.18311 | Quinones | 293.2 | 96 | - | 12.90255 | 9.80324E-06 | 2.97988E-05 | 4.89308E-05 | 2.95109E-05 | 0.000150439 | 6.47403E-05 | 6.21045E-05 | 9.24279E-05 | 0.000204599 | 8.59457E-05 | 0.000145273 |
| 487 | Encecalin | 20628-09-5 | C09005 | C14H16O3 | 232.10994500000001 | Phenols | 233.1 | 177.1 | + | 10.56445 | 0.000131292 | 0.000354886 | 0.000182287 | 0.000222822 | 0.000101409 | 0.000225616 | 0.00012231 | 0.000149778 | 0.000214047 | 0.000192309 | 0.000203178 |
| 488 | Enoxacin | 74011-58-8 | C06979 | C15H17FN4O3 | 320.128469 | Diazanaphthalenes | 321.1 | 303.1 | + | 10.84053333 | 9.33351E-05 | 0.000103752 | 0.000128521 | 0.000108536 | 0.000262723 | 0.000141716 | 0.000120379 | 0.000174939 | 9.99436E-05 | 9.48713E-05 | 9.74074E-05 |
| 489 | ent-16beta,17-dihydroxy-9(11)-kauren-19-oic acid | 55483-24-4 |  | C20H30O4 | 334.21440999999999 | Diterpenoids | 335.2 | 271.2 | + | 7.149766667 | 0.00026986 | 8.31755E-05 | 6.56109E-05 | 0.000139549 | 0.00027034 | 6.5713E-05 | 9.94523E-05 | 0.000145169 | 0.000168401 | 0.000104202 | 0.000136301 |
| 491 | Enterolactone | 78473-71-9 | C18165 | C18H18O4 | 298.12051000000002 | Lignans | 299.1 | 107 | + | 12.06515 | 0.000649332 | 0.000625331 | 0.000357686 | 0.000544116 | 0.000677226 | 0.000527458 | 0.000282344 | 0.000495676 | 0.000436312 | 0.000514402 | 0.000475357 |
| 492 | Epiandrosterone | 481-29-8 | C07635 | C19H30O2 | 290.22458 | Steroids and steroid derivatives | 291.2 | 273.2 | + | 5.165133333 | 0.000150413 | 3.37574E-05 | 7.86481E-05 | 8.76063E-05 | 0.000196251 | 0.000100513 | 5.36931E-05 | 0.000116819 | 7.87768E-05 | 0.000205421 | 0.000142099 |
| 493 | Epicatechin;(+)-Epicatechin | 490-46-0;35323-91-2 | C09727;C09728 | C15H14O6 | 290.07904000000002 | Flavonoids | 291.1 | 139 | + | 5.07785 | 6.82585E-05 | 3.88874E-05 | 3.80878E-05 | 4.84113E-05 | 0.000138696 | 4.3109E-05 | 8.29116E-06 | 6.33653E-05 | 9.97486E-05 | 0.000108322 | 0.000104035 |
| 494 | Epifriedelanol | 16844-71-6 | C17123 | C30H52O | 428.401815 | Triterpenoids | 429.4 | 411.4 | + | 12.28533333 | 0.002626533 | 0.003466544 | 0.001582847 | 0.002558641 | 0.002650298 | 0.002787112 | 0.001539121 | 0.00232551 | 0.003735021 | 0.003241934 | 0.003488477 |
| 495 | Epigallate catechin gallate (EGCG) | 989-51-5 | C09731 | C22H18O11 | 458.08491500000002 | Flavonoids | 459.1 | 139 | + | 5.14 | 0.00191613 | 5.1848E-05 | 1.53534E-05 | 0.00066111 | 9.68474E-05 | 4.32411E-05 | 1.23455E-06 | 4.71077E-05 | 0.000115359 | 0.00019539 | 0.000155374 |
| 496 | Epitulipinolide | 24164-13-4 | C09566 | C17H22O4 | 290.15181000000001 | Sesquiterpenoids | 291.2 | 231.1 | + | 5.068683333 | 0.000599545 | 0.000588187 | 0.000581716 | 0.000589816 | 0.000542247 | 0.000652892 | 0.000739835 | 0.000644991 | 0.000323221 | 0.000525937 | 0.000424579 |
| 498 | Ergocristine | 511-08-0 | C09164 | C35H39N5O5 | 609.29512 | Alkaloids | 610.3 | 251.1 | + | 9.180483333 | 2.24665E-05 | 8.25278E-05 | 2.86667E-05 | 4.45537E-05 | 4.244E-05 | 9.02383E-05 | 1.21143E-05 | 4.82642E-05 | 1.36623E-05 | 3.76923E-05 | 2.56773E-05 |
| 499 | Ergothioneine | 58511-63-0;497-30-3 | C05570 | C9H15N3O2S | 229.08849799999999 | Carboxylic acids and derivatives | 230.1 | 127 | + | 0.520516667 | 0.000159808 | 0.000291802 | 0.000158135 | 0.000203248 | 0.000284481 | 0.000298486 | 0.000227909 | 0.000270292 | 0.000174243 | 0.000120665 | 0.000147454 |
| 500 | Eriocitrin | 13463-28-0 | C09732 | C27H32O15 | 596.17409999999995 | flavonoids | 595 | 458.9 | - | 5.79 | 4.35409E-06 | 4.19406E-05 | 1.12648E-06 | 1.58071E-05 | 2.59039E-05 | 1.26522E-06 | 4.45053E-06 | 1.05399E-05 | 1.58124E-05 | 5.02605E-05 | 3.30364E-05 |
| 501 | Eriodictyol | 552-58-9 | C05631 | C15H12O6 | 288.0634 | flavonoids | 288.9 | 163 | + | 7.9 | 0.000228712 | 0.000450703 | 0.000332623 | 0.000337346 | 0.000316737 | 0.000380406 | 0.000234869 | 0.00031067 | 0.000496659 | 0.000301123 | 0.000398891 |
| 502 | Erucic acid | 112-86-7 | C08316 | C22H42O2 | 338.31848000000002 | Fatty Acyls | 339.3 | 321.3 | + | 10.4583 | 0.000136591 | 3.14545E-05 | 0.000231531 | 0.000133192 | 0.000203193 | 7.46979E-05 | 6.08187E-05 | 0.000112903 | 6.47005E-05 | 9.72122E-05 | 8.09563E-05 |
| 503 | Esculin | 531-75-9 | C09264 | C15H16O9 | 340.07943499999999 | Coumarins | 341.1 | 179 | + | 4.230816667 | 0.002669134 | 0.003961197 | 0.003581805 | 0.003404046 | 0.004409497 | 0.003081603 | 0.002942617 | 0.003477905 | 0.003269759 | 0.00318923 | 0.003229495 |
| 504 | Estradiol-17beta 3-sulfate | 4999-79-5 | C08357 | C18H24O5S | 352.13444600000003 | Sterol lipids | 351.1 | 271.2 | - | 11.89345 | 8.75512E-05 | 5.56384E-05 | 1.28088E-05 | 5.19995E-05 | 2.04832E-05 | 3.65936E-05 | 2.22653E-05 | 2.64474E-05 | 1.60049E-05 | 6.14604E-05 | 3.87326E-05 |
| 505 | Estriol 3-sulfate 16-glucuronide | 4661-65-8 | C03033 | C24H32O12S | 544.16145100000006 | Organooxygen compounds | 545.2 | 351.1 | + | 12.1175 | 0.000376016 | 0.000543644 | 0.000600048 | 0.000506569 | 0.000388739 | 0.000332869 | 0.000227299 | 0.000316302 | 0.00058451 | 0.000361071 | 0.000472791 |
| 506 | Ethyl 3,4,5-trimethoxybenzoate | 6178-44-5 |  | C12H16O5 | 240.09977499999999 | Phenols | 241.1 | 195.1 | + | 0.529283333 | 4.22257E-05 | 8.21321E-05 | 6.25746E-05 | 6.23108E-05 | 9.5499E-05 | 9.99833E-05 | 0.000103929 | 9.98038E-05 | 7.12879E-05 | 8.86323E-05 | 7.99601E-05 |
| 507 | Ethyl acrylate | 140-88-5 | C19238 | C5H8O2 | 100.05243 | Carboxylic acids and derivatives | 101.1 | 55 | + | 1.73615 | 0.001544866 | 0.00174422 | 0.001721582 | 0.001670223 | 0.004005705 | 0.003709831 | 0.002869672 | 0.003528403 | 0.002487497 | 0.002607176 | 0.002547337 |
| 508 | Ethyl caproate | 123-66-0 |  | C8H16O2 | 144.11502999999999 | Miscellaneous | 145.1 | 57.1 | + | 0.7377 | 0.003738727 | 0.004723049 | 0.004462185 | 0.004307987 | 0.003340218 | 0.004096566 | 0.003305904 | 0.003580896 | 0.002280358 | 0.000606808 | 0.001443583 |
| 509 | Ethyl cinnamate | 103-36-6 | C06359 | C11H12O2 | 176.08373 | Phenylpropanoids | 177.1 | 131 | + | 4.8505 | 0.000159446 | 0.000435859 | 0.000210775 | 0.000268694 | 0.000543539 | 0.000352793 | 0.000365035 | 0.000420456 | 0.000306995 | 0.000196421 | 0.000251708 |
| 510 | Ethyl gallate | 831-61-8 |  | C9H10O5 | 198.05282500000001 | Phenols | 199.1 | 153 | + | 8.862616667 | 4.47267E-05 | 0.000249552 | 0.000153408 | 0.000149229 | 0.000357562 | 0.000215763 | 8.67902E-05 | 0.000220038 | 0.0001303 | 0.000235847 | 0.000183073 |
| 511 | Ethyl isovalerate | 108-64-5 | C12290 | C7H14O2 | 130.09938 | Miscellaneous | 131.1 | 57.1 | + | 1.190583333 | 0.001481585 | 0.002474023 | 0.001814141 | 0.00192325 | 0.001933058 | 0.00224983 | 0.00149339 | 0.001892093 | 0.002290103 | 0.002098642 | 0.002194373 |
| 512 | Ethyl trans-p-methoxycinnamate | 1929-30-2 | C10476 | C12H14O3 | 206.09429499999999 | Cinnamic acids and derivatives | 207.1 | 133.1 | + | 4.967983333 | 0.005697491 | 0.005794863 | 0.00524337 | 0.005578575 | 0.009994156 | 0.006105691 | 0.005453422 | 0.007184423 | 0.00575032 | 0.005082719 | 0.005416519 |
| 513 | Etiocholanolone | 53-42-9 | C04373 | C19H30O2 | 290.22458 | Steroids and steroid derivatives | 291.2 | 255.2 | + | 5.074283333 | 2.28118E-05 | 0.000197029 | 3.82289E-05 | 8.60233E-05 | 6.4729E-05 | 2.55366E-05 | 3.26999E-05 | 4.09885E-05 | 0.000108244 | 0.000108988 | 0.000108616 |
| 514 | Etoposide | 33419-42-0 | C01576 | C29H32O13 | 588.18429500000002 | Lignans | 611.2 | 405.1 | + | 12.79708333 | 0.001617795 | 0.001596501 | 0.000656878 | 0.001290391 | 0.000809561 | 0.000737418 | 0.00115011 | 0.000899029 | 0.002161614 | 0.000468679 | 0.001315147 |
| 515 | Eupatolide | 6750-25-0 | C09440 | C15H20O3 | 248.141245 | Sesquiterpenoids | 249.1 | 231.1 | + | 7.175316667 | 0.00012566 | 5.1204E-05 | 0.000198868 | 0.000125244 | 0.000123899 | 0.000159209 | 6.5825E-05 | 0.000116311 | 4.37862E-05 | 0.0001373 | 9.05431E-05 |
| 516 | Eurycomalactone;Ingenol | 23062-24-0;30220-46-3 | C08759;C09112 | C19H24O6;C20H28O5 | 348.15728999999999;348.19367499999998 | Diterpenoids | 349.2 | 331.2 | + | 5.79035 | 8.13021E-05 | 0.000311315 | 0.000185739 | 0.000192785 | 8.75448E-05 | 8.4272E-05 | 0.000266648 | 0.000146155 | 0.000471335 | 0.000279853 | 0.000375594 |
| 517 | Exemestane | 107868-30-4 | C08162 | C20H24O2 | 296.17763000000002 | Steroids and steroid derivatives | 297.2 | 121.1 | + | 11.61401667 | 0.004624393 | 0.002342172 | 0.000515192 | 0.002493919 | 0.000782266 | 0.001975762 | 0.001549532 | 0.001435853 | 0.001252541 | 0.000428848 | 0.000840694 |
| 518 | Farrerol | 24211-30-1 | C09734 | C17H16O5 | 300.09977500000002 | Flavonoids | 301.1 | 105 | + | 8.458833333 | 0.000101091 | 0.000100105 | 0.000138043 | 0.00011308 | 0.000379156 | 0.000153609 | 0.000150945 | 0.000227903 | 0.000262534 | 7.44446E-05 | 0.000168489 |
| 519 | Ferruginol | 514-62-5 | C09092 | C20H30O | 286.22966500000001 | Diterpenoids | 287.2 | 271.2 | + | 7.586116667 | 2.92158E-05 | 4.54894E-05 | 4.0077E-05 | 3.82607E-05 | 6.8106E-05 | 0.000134541 | 2.85444E-05 | 7.70639E-05 | 4.02718E-05 | 7.50901E-05 | 5.76809E-05 |
| 520 | Ferulic acid; Trans-Ferulic acid;trans-Ferulic acid | 1135-24-6;537-98-4 | C01494 | C10H10O4 | 194.05790999999999 | Phenylpropanoids | 195.1 | 117 | + | 4.85895 | 0.000641422 | 0.000549666 | 0.000662376 | 0.000617821 | 0.001522018 | 0.00087109 | 0.001176269 | 0.001189792 | 0.000831532 | 0.001325588 | 0.00107856 |
| 521 | Fingolimod hydrochloride | 162359-56-0 |  | C19H33NO2 | 307.25112899999999 | Miscellaneous | 308.3 | 105.1 | + | 13.10768333 | 0.001081267 | 0.001283556 | 0.001131361 | 0.001165395 | 0.00233159 | 0.001297456 | 0.002004922 | 0.001877989 | 0.001752185 | 0.001886809 | 0.001819497 |
| 522 | Fisetin | 528-48-3 | C01378 | C15H12O6 | 288.0634 | flavonoids | 286.9 | 241 | + | 7.03 | 0.000306925 | 0.000510146 | 0.000456416 | 0.000424496 | 0.000853338 | 0.000391575 | 0.000346439 | 0.000530451 | 0.00071482 | 0.000366238 | 0.000540529 |
| 523 | Flavin adenine dinucleotide (FAD) | 146-14-5 | C00016 | C27H33N9O15P2 | 785.15714000000003 | Nucleotide and its derivates | 786.2 | 348.1 | + | 4.0818 | 0.003814879 | 0.002577749 | 0.002027185 | 0.002806604 | 0.003717896 | 0.002688918 | 0.002975697 | 0.003127504 | 0.004615344 | 0.002570908 | 0.003593126 |
| 524 | Flavone | 525-82-6 | C15608;C10043 | C15H10O2 | 222.06808000000001 | Flavonoids | 223.1 | 77 | + | 4.128633333 | 0.000225473 | 9.87196E-05 | 0.00011369 | 0.000145961 | 0.0003663 | 0.000259696 | 0.000254638 | 0.000293545 | 0.000269747 | 0.000313686 | 0.000291717 |
| 525 | Formononetin | 485-72-3 | C00858 | C16H12O4 | 268.07355999999999 | Flavonoids | 269.1 | 197.1 | + | 9.9393 | 0.00010729 | 0.000191953 | 0.000106413 | 0.000135219 | 0.000140636 | 0.00012607 | 0.000181772 | 0.000149493 | 0.000132074 | 0.000123291 | 0.000127683 |
| 526 | Formylanthranilic acid | 3342-77-6 | C05653 | C8H7NO3 | 165.04259400000001 | Benzene and substituted derivatives | 166 | 120 | + | 5.0742 | 0.000940975 | 0.001249989 | 0.000655864 | 0.000948943 | 0.001141337 | 0.000590365 | 0.00100997 | 0.000913891 | 0.000823572 | 0.000719557 | 0.000771565 |
| 527 | Fraxetin | 574-84-5 | C09265 | C10H8O5 | 208.03717499999999 | Coumarins | 209 | 82 | + | 5.255583333 | 8.2266E-05 | 0.000108803 | 6.54871E-05 | 8.55186E-05 | 9.85998E-05 | 0.00013941 | 7.93282E-06 | 8.19808E-05 | 3.54032E-05 | 8.53155E-05 | 6.03593E-05 |
| 528 | Fucosterol | 17605-67-3 | C08817 | C29H48O | 412.37051500000001 | Steroids | 413.4 | 353.3 | + | 12.07523333 | 9.49398E-05 | 0.000184953 | 1.12648E-06 | 9.3673E-05 | 0.000181071 | 8.92547E-05 | 6.69949E-05 | 0.00011244 | 4.50013E-05 | 0.000199803 | 0.000122402 |
| 529 | Fucoxanthin | 3351-86-8 | C08596 | C42H58O6 | 658.42334000000005 | Miscellaneous | 659.4 | 109.1 | + | 12.49073333 | 0.00036579 | 0.000288269 | 0.000307751 | 0.000320603 | 0.000890002 | 0.000835342 | 0.001270507 | 0.000998617 | 0.000775624 | 0.000358386 | 0.000567005 |
| 530 | Fulvine | 6029-87-4 | C10304 | C16H23NO5 | 309.157624 | Alkaloids | 310.2 | 292.2 | + | 7.9889 | 4.85424E-05 | 3.41689E-05 | 4.1655E-05 | 4.14554E-05 | 4.78441E-05 | 6.23278E-05 | 3.782E-05 | 4.93306E-05 | 3.59788E-05 | 6.63619E-05 | 5.11703E-05 |
| 531 | Furanodiene | 19912-61-9 | C16959 | C15H20O | 216.15141499999999 | Sesquiterpenoids | 217.2 | 201.1 | + | 10.01963333 | 2.60182E-05 | 5.16225E-05 | 6.97956E-05 | 4.91455E-05 | 7.68637E-05 | 5.45732E-05 | 6.23024E-05 | 6.45798E-05 | 3.65023E-05 | 1.52123E-05 | 2.58573E-05 |
| 532 | Furfuryl acetate | 623-17-6 |  | C7H8O3 | 140.04734500000001 | Miscellaneous | 141.1 | 81 | + | 5.471466667 | 0.000448787 | 0.000323671 | 0.000466567 | 0.000413008 | 0.000228403 | 0.000216879 | 0.000178877 | 0.000208053 | 0.000418792 | 0.000211518 | 0.000315155 |
| 533 | Fustin | 20725-03-5 | C01378 | C15H12O6 | 288.06339000000003 | Flavonoids | 289.1 | 121 | + | 7.587616667 | 0.003255967 | 0.003905021 | 0.004276721 | 0.00381257 | 0.004963429 | 0.002875376 | 0.002063799 | 0.003300868 | 0.003924991 | 0.002764668 | 0.003344829 |
| 534 | g-Strophanthin | 630-60-4 | C01443 | C29H44O12 | 584.28327999999999 | Steroids and steroid derivatives | 585.3 | 355.2 | + | 12.78815 | 0.000730137 | 0.220952422 | 0.182775631 | 0.134819397 | 0.294359253 | 0.153436166 | 0.148458466 | 0.198751295 | 0.191910483 | 0.18729049 | 0.189600486 |
| 535 | Galactinol | 3687-64-7 | C01235 | C12H22O11 | 342.11621500000001 | Organooxygen compounds | 343.1 | 181.1 | + | 2.789716667 | 0.001288674 | 0.017702034 | 0.01546567 | 0.011485459 | 0.018554768 | 0.001374553 | 0.018135725 | 0.012688349 | 0.001290471 | 0.001112587 | 0.001201529 |
| 537 | Galantamine | 357-70-0 | C08526 | C17H21NO3 | 287.15214400000002 | Alkaloids | 288.2 | 213.1 | + | 6.526 | 5.11502E-05 | 9.02153E-05 | 0.000129214 | 9.01933E-05 | 0.000116791 | 0.000114167 | 0.000256459 | 0.000162473 | 7.83921E-05 | 0.000117804 | 9.80979E-05 |
| 538 | Galanthaminone | 510-77-0 | C08534 | C17H19NO3 | 285.13649400000003 | Alkaloids | 286.1 | 242.1 | + | 6.9108 | 2.21126E-05 | 5.10164E-06 | 1.12648E-06 | 9.4469E-06 | 3.99119E-05 | 5.91552E-05 | 4.64933E-05 | 4.85201E-05 | 5.30167E-05 | 1.01912E-05 | 3.16039E-05 |
| 539 | Gallocatechin | 970-73-0 | C12127 | C15H14O7 | 306.07400000000001 | flavonoids | 306.9 | 180.9 | + | 3.36 | 4.93059E-05 | 6.90791E-05 | 3.25551E-05 | 5.03133E-05 | 0.000123279 | 4.12208E-05 | 4.17216E-05 | 6.87404E-05 | 7.33685E-05 | 5.12785E-05 | 6.23235E-05 |
| 541 | gamma-Hydroxy-3-pyridinebutanoate | 15569-97-8 | C19579 | C9H11NO3 | 181.073894 | Pyridines and derivatives | 182.1 | 164.1 | + | 4.3638 | 3.30933E-05 | 4.64313E-05 | 0.000105568 | 6.16977E-05 | 0.000428946 | 0.00034783 | 0.000362968 | 0.000379915 | 0.000128596 | 0.000167754 | 0.000148175 |
| 542 | Ganoderal A | 104700-98-3 |  | C30H44O2 | 436.33413000000002 | Triterpenoids | 437.3 | 419.3 | + | 8.0896 | 8.2208E-05 | 7.08247E-05 | 5.07058E-05 | 6.79128E-05 | 7.12631E-05 | 8.41191E-05 | 7.14427E-05 | 7.56083E-05 | 8.24449E-05 | 7.25653E-05 | 7.75051E-05 |
| 543 | Ganoderenic acid E | 110241-23-1 |  | C30H40O8 | 528.27232000000004 | Triterpenoids | 529.3 | 493.3 | + | 12.78881667 | 0.001223658 | 0.000838877 | 0.00089822 | 0.000986918 | 0.000529926 | 0.000904061 | 0.000505779 | 0.000646589 | 0.000690614 | 0.000965743 | 0.000828178 |
| 544 | Ganoderic acid A;Ganoderenic acid C;Ganoderic acid B | 81907-62-2;100665-42-7;81907-61-1 | | C30H44O7 | 516.30870500000003 | Triterpenoids | 517.3 | 481.3 | + | 12.38601667 | 0.000824025 | 0.001414228 | 0.000787915 | 0.001008723 | 0.000721364 | 0.000804355 | 0.000691155 | 0.000738958 | 0.000724373 | 0.000396015 | 0.000560194 |
| 545 | Ganoderic acid D2 | 97653-94-6 |  | C30H42O8 | 530.28796999999997 | Triterpenoids | 531.3 | 467.3 | + | 12.59793333 | 8.64255E-05 | 5.52592E-05 | 5.63499E-05 | 6.60115E-05 | 7.24751E-05 | 1.26522E-06 | 8.24543E-05 | 5.20649E-05 | 0.000326273 | 6.18497E-05 | 0.000194061 |
| 546 | Ganoderic acid F | 98665-15-7 |  | C32H42O9 | 570.28288499999996 | Triterpenoids | 571.3 | 553.3 | + | 12.97341667 | 0.006271439 | 0.051052962 | 0.039655334 | 0.032326579 | 0.067792008 | 0.07264391 | 0.066117716 | 0.068851211 | 0.074014102 | 0.036279636 | 0.055146869 |
| 547 | Ganoderic acid H | 98665-19-1 |  | C32H44O9 | 572.29853500000002 | Triterpenoids | 573.3 | 555.3 | + | 11.73148333 | 0.007784947 | 0.009259846 | 0.005304821 | 0.007449871 | 0.008291717 | 0.007617468 | 0.007488017 | 0.007799067 | 0.009146538 | 0.006609041 | 0.00787779 |
| 548 | Ganoderic acid L | 102607-24-9 |  | C30H46O8 | 534.31926999999996 | Alkaloids | 535.3 | 499.3 | + | 5.823916667 | 0.000286269 | 0.000187501 | 9.22909E-05 | 0.000188687 | 0.000189967 | 9.35419E-05 | 7.85725E-05 | 0.000120694 | 0.000170524 | 0.000157376 | 0.00016395 |
| 549 | Ganoderic acid N | 110241-19-5 |  | C30H42O8 | 530.28796999999997 | Triterpenoids | 531.3 | 495.3 | + | 12.59793333 | 0.002040809 | 0.001546074 | 0.000383053 | 0.001323312 | 0.002366257 | 0.000304917 | 0.001598741 | 0.001423305 | 0.000443093 | 0.002018923 | 0.001231008 |
| 550 | Ganoderol A | 104700-97-2 |  | C30H46O2 | 438.34978000000001 | Triterpenoids | 439.4 | 421.3 | + | 11.31191667 | 1.18251E-06 | 0.000217046 | 1.12648E-06 | 7.31183E-05 | 0.000181857 | 1.26522E-06 | 0.000215141 | 0.000132754 | 0.000139632 | 0.000383734 | 0.000261683 |
| 552 | Gastrodin | 62499-27-8 | C16964 | C13H18O7 | 286.105255 | Phenols | 287.1 | 107 | + | 2.53400463 | 7.05588E-05 | 0.000141031 | 7.51705E-05 | 9.55868E-05 | 0.000118357 | 0.000110739 | 5.89927E-05 | 9.60294E-05 | 0.000192251 | 8.00243E-05 | 0.000136138 |
| 553 | Gemcitabine | 95058-81-4 | C07650 | C9H11F2N3O4 | 263.07176299999998 | Pyrimidine nucleosides | 264.1 | 112.1 | + | 0.745466667 | 0.000757125 | 0.000601942 | 0.00062658 | 0.000661883 | 0.00081655 | 0.000764878 | 0.000707367 | 0.000762932 | 0.000929393 | 0.001005318 | 0.000967355 |
| 554 | Genipin | 6902-77-8 | C09780 | C11H14O5 | 226.084125 | Iridoids | 227.1 | 149.1 | + | 4.8505 | 0.000804365 | 0.001169853 | 0.001303303 | 0.001092507 | 0.001179969 | 0.00076803 | 0.000643405 | 0.000863801 | 0.000727025 | 0.000631268 | 0.000679147 |
| 555 | Genipin-1-O-gentiobioside | 29307-60-6 | C16965 | C23H34O15 | 550.18977500000005 | Iridoids | 551.2 | 515.2 | + | 4.665606648 | 4.02595E-05 | 4.94415E-05 | 8.69149E-05 | 5.8872E-05 | 0.000126267 | 6.78571E-05 | 3.16205E-05 | 7.52483E-05 | 6.07618E-05 | 3.35338E-05 | 4.71478E-05 |
| 556 | Geniposide | 24512-63-8 | C09781 | C17H24O10 | 388.13695000000001 | Iridoids | 389.1 | 209.1 | + | 5.142134145 | 0.000252196 | 0.000621097 | 0.000231516 | 0.00036827 | 0.000360652 | 0.000241763 | 0.000433327 | 0.000345248 | 7.07894E-05 | 0.000318307 | 0.000194548 |
| 557 | Geniposidic acid;Geniposidic acid | 27741-01-1 | C11673 | C16H22O10 | 374.12130000000002 | Iridoids;Terpene | 375.1 | 339.1 | + | 3.696899986 | 0.000194932 | 0.000194264 | 0.00023482 | 0.000208005 | 0.000318302 | 0.000200217 | 0.000246462 | 0.000254993 | 0.000205937 | 0.000176437 | 0.000191187 |
| 558 | Genistein | 446-72-0 | C06563 | C15H10O5 | 270.05279999999999 | flavonoids | 271.2 | 242.8 | + | 8.93 | 0.000136434 | 0.000198404 | 0.000293598 | 0.000209479 | 0.000250874 | 0.000244107 | 0.00028785 | 0.000260944 | 0.000478963 | 0.000510583 | 0.000494773 |
| 559 | Genistin | 529-59-9 |  | 21H20O10 | 432.10564699999998 | flavonoids | 433.3 | 270.8 | + | 6.62 | 0.000160686 | 0.000218978 | 0.000159438 | 0.0001797 | 0.001291116 | 0.000933183 | 0.000233305 | 0.000819201 | 0.000251054 | 0.000594564 | 0.000422809 |
| 560 | Gentioflavin | 18058-50-9 | C09962 | C10H11NO3 | 193.073894 | Alkaloids | 194.1 | 148.1 | + | 4.934416667 | 0.000104216 | 0.000312142 | 0.000146629 | 0.000187663 | 0.000142434 | 0.000244114 | 0.00018365 | 0.000190066 | 0.000198394 | 6.15327E-05 | 0.000129963 |
| 561 | Gentisein;1,3,5-Trihydroxyxanthone | 529-49-7;6732-85-0 | C10065;C10094 | C13H8O5 | 244.03717499999999 | Xanthones | 245 | 135 | + | 8.72735 | 8.19615E-05 | 6.67936E-05 | 5.1933E-05 | 6.68961E-05 | 4.15913E-05 | 4.54048E-05 | 6.2558E-05 | 4.98514E-05 | 8.67053E-05 | 9.00562E-05 | 8.83807E-05 |
| 563 | Germanicol | 465-02-1 | C19833 | C30H50O | 426.38616500000001 | Triterpenoids | 427.4 | 409.4 | + | 12.63101667 | 0.000535214 | 0.0011432 | 0.000230994 | 0.00063647 | 0.000465122 | 0.000389808 | 0.000274165 | 0.000376365 | 0.000408581 | 0.000261074 | 0.000334827 |
| 564 | Gibberellin A1 | 545-97-1 | C00859 | C19H24O6 | 348.15730000000002 | phytohormone | 347 | 259 | - | 6.12 | 7.66626E-05 | 0.000108344 | 5.99324E-05 | 8.16465E-05 | 6.16275E-05 | 9.05529E-05 | 2.88583E-05 | 6.03462E-05 | 0.00013161 | 8.66183E-05 | 0.000109114 |
| 565 | Gibberellin A3 | 77-06-5 | C01699 | C19H22O6 | 346.14159999999998 | phytohormone | 345 | 143 | - | 6.04 | 6.68798E-05 | 5.63614E-05 | 1.12648E-06 | 4.14559E-05 | 0.000140603 | 0.000103992 | 4.83534E-05 | 9.76496E-05 | 5.30063E-05 | 0.000124837 | 8.89218E-05 |
| 566 | Gibberellin A4 | 468-44-0 | C11864 | C19H24O5 | 332.16239999999999 | phytohormone | 331 | 243 | - | 9.98 | 3.794E-05 | 8.17922E-05 | 6.32827E-05 | 6.1005E-05 | 7.81732E-05 | 3.36818E-05 | 4.37559E-05 | 5.18703E-05 | 5.38863E-05 | 3.97991E-05 | 4.68427E-05 |
| 567 | Gibberellin A7 | 510-75-8 | C11867 | C19H22O5 | 330.14670000000001 | phytohormone | 329 | 223 | - | 9.85 | 7.42246E-05 | 3.93262E-05 | 5.78705E-05 | 5.71404E-05 | 4.6672E-05 | 2.92319E-05 | 5.94933E-05 | 4.51324E-05 | 2.15866E-05 | 4.73687E-05 | 3.44776E-05 |
| 568 | Ginkgolic acid C15:1 | 22910-60-7 | C10794 | C22H34O3 | 346.25079499999998 | Phenols | 345.2 | 301.3 | - | 7.57765 | 5.78999E-05 | 2.27384E-05 | 7.98518E-05 | 5.34967E-05 | 9.57446E-05 | 3.49073E-05 | 8.90242E-05 | 7.32254E-05 | 2.28959E-05 | 4.64221E-05 | 3.4659E-05 |
| 570 | Ginsenoside F2 | 62025-49-4 |  | C42H72O13 | 784.49729500000001 | Triterpenoids | 785.5 | 605.4 | + | 12.14981667 | 0.000562833 | 0.000630803 | 0.00037046 | 0.000521365 | 0.000835071 | 0.000852194 | 1.23455E-06 | 0.000562833 | 0.001120228 | 0.000545626 | 0.000832927 |
| 571 | Ginsenoside F3 | 62025-50-7 |  | C41H70O13 | 770.48164499999996 | Triterpenoids | 771.5 | 735.5 | + | 12.3224 | 0.000996596 | 0.001089152 | 0.001569514 | 0.001218421 | 0.001299087 | 0.000665868 | 0.000601596 | 0.000855517 | 0.00181326 | 0.001127214 | 0.001470237 |
| 572 | Ginsenoside Rf | 52286-58-5 | C08945 | C42H72O14 | 800.49221 | Triterpenoids | 801.5 | 765.5 | + | 9.014578 | 0.000101105 | 0.000140499 | 8.56859E-05 | 0.000109097 | 9.90868E-05 | 0.000154765 | 0.000195 | 0.000149617 | 0.000102441 | 0.000470295 | 0.000286368 |
| 573 | Ginsenoside Rg1 | 22427-39-0 | C08946 | C42H72O14 | 800.49221 | Triterpenoids | 801.5 | 621.4 | + | 7.90011087 | 0.000103236 | 2.02701E-05 | 6.62655E-05 | 6.32571E-05 | 6.00262E-05 | 8.23364E-05 | 0.000135464 | 9.26089E-05 | 3.70547E-05 | 0.00015771 | 9.73824E-05 |
| 574 | Glabridin | 59870-68-7 | C10421 | C20H20O4 | 324.13619999999997 | flavonoids | 325.4 | 188.9 | + | 11.58 | 0.003984947 | 0.004768812 | 0.003756893 | 0.004170217 | 0.005515368 | 0.004949396 | 0.003873397 | 0.004779387 | 0.00411319 | 0.002279063 | 0.003196127 |
| 575 | Glucocheirolin | 15592-36-6 | C08405 | C11H21NO11S3 | 439.02767699999998 | Miscellaneous | 440 | 165 | + | 8.72735 | 7.04228E-05 | 0.000102083 | 6.70705E-05 | 7.98588E-05 | 9.34538E-05 | 8.8408E-05 | 1.59797E-05 | 6.59472E-05 | 2.67042E-05 | 0.000103197 | 6.49509E-05 |
| 576 | Glucofrangulin B | 14062-59-0 | C16803 | C26H28O14 | 564.14791000000002 | Anthraquinones | 565.2 | 385.1 | + | 5.68965 | 0.000179465 | 0.000115468 | 0.000347863 | 0.000214265 | 0.00022318 | 0.000177495 | 0.000181589 | 0.000194088 | 0.000159505 | 0.000156897 | 0.000158201 |
| 577 | Glucoiberin | 554-88-1 | C08411 | C11H21NO10S3 | 423.03276199999999 | Organooxygen compounds | 424 | 149 | + | 10.46526667 | 7.49564E-06 | 6.38993E-05 | 2.16812E-05 | 3.10254E-05 | 0.0001753 | 3.25183E-05 | 3.10113E-05 | 7.96099E-05 | 8.78656E-05 | 9.07141E-05 | 8.92898E-05 |
| 578 | Glucolimnanthin | 111810-95-8 | C08414 | C15H21NO10S2 | 439.06069100000002 | Miscellaneous | 440.1 | 165 | + | 8.72735 | 7.04228E-05 | 0.000102083 | 6.70705E-05 | 7.98588E-05 | 9.34538E-05 | 8.8408E-05 | 1.59797E-05 | 6.59472E-05 | 2.67042E-05 | 0.000103197 | 6.49509E-05 |
| 579 | Gluconapin | 19041-09-9 | C08415 | C11H19NO9S2 | 373.05012599999998 | Carbohydrates | 374.1 | 99 | + | 5.656083333 | 5.00412E-05 | 0.000876303 | 5.77049E-05 | 0.000328016 | 0.000217211 | 0.000184468 | 0.000600622 | 0.0003341 | 0.000129062 | 0.000566807 | 0.000347934 |
| 581 | Glucoraphanin | 21414-41-5 | C08419 | C12H23NO10S3 | 437.04841199999998 | Miscellaneous | 438.1 | 163 | + | 8.72735 | 7.63332E-06 | 0.000127015 | 7.01518E-06 | 4.72211E-05 | 6.22025E-05 | 1.71827E-05 | 1.23455E-06 | 2.68732E-05 | 4.23194E-05 | 5.50407E-05 | 4.86801E-05 |
| 582 | Glucosamine | 3416-24-8 | C00329 | C6H13NO5 | 179.079374 | Carbohydrates | 180.1 | 162.1 | + | 2.6016 | 0.004144787 | 0.00463801 | 0.003865097 | 0.004215965 | 0.005896713 | 0.005311015 | 0.002768091 | 0.004658607 | 0.005421232 | 0.00545326 | 0.005437246 |
| 583 | Glucose 1-phosphate | 59-56-3 | C00103 | C6H13O9P | 260.02972199999999 | Organooxygen compounds | 283 | 121 | + | 12.9983 | 0.028647181 | 0.025205813 | 0.020697398 | 0.024850131 | 0.034191047 | 0.027127751 | 0.024000507 | 0.028439769 | 0.028447097 | 0.027105725 | 0.027776411 |
| 584 | Glutathione | 70-18-8 | C00051 | C10H17N3O6S | 307.08380799999998 | Amino acid and derivatives | 308.1 | 76 | + | 2.021866667 | 0.000392614 | 0.000206813 | 0.000156725 | 0.000252051 | 0.000155692 | 0.000247338 | 0.000176835 | 0.000193289 | 0.000561035 | 0.000157397 | 0.000359216 |
| 585 | Glutathione oxidized | 27025-41-8 | C00127 | C20H32N6O12S2 | 612.15196600000002 | Amino acid and derivatives | 613.2 | 355.1 | + | 2.517683333 | 0.000182342 | 0.000126271 | 0.000130007 | 0.000146207 | 0.00039966 | 0.000272476 | 0.000197626 | 0.000289921 | 0.000251957 | 0.000120072 | 0.000186015 |
| 586 | Glyceric acid | 473-81-4 | C00258 | C3H6O4 | 106.02661000000001 | Organooxygen compounds | 107 | 89 | + | 0.554083333 | 0.000828601 | 0.000864424 | 0.000621833 | 0.000771619 | 0.000938983 | 0.000483408 | 0.000765822 | 0.000729404 | 0.000474312 | 0.000821867 | 0.00064809 |
| 587 | Glycerophosphocholine | 28319-77-9 | C00670 | C8H20NO6P | 257.10282599999999 | Cholines | 258.1 | 104.1 | + | 1.473683333 | 0.000242022 | 0.000665825 | 0.000591989 | 0.000499945 | 0.000742466 | 0.000396776 | 0.000567586 | 0.000568943 | 0.000570212 | 0.000812208 | 0.00069121 |
| 588 | Glycinol | 69393-95-9 | C01263 | C15H12O5 | 272.06847499999998 | Flavonoids | 273.1 | 95 | + | 6.545583333 | 0.000146516 | 0.000298986 | 0.0001367 | 0.000194067 | 0.000226902 | 0.000172043 | 0.000170509 | 0.000189818 | 0.000222926 | 0.000129105 | 0.000176016 |
| 589 | Glycitein | 40957-83-3 | C14536 | C16H12O5 | 284.06849999999997 | flavonoids | 285.3 | 269.8 | + | 7.91 | 6.05588E-05 | 5.6769E-05 | 0.000119877 | 7.90684E-05 | 0.000148462 | 4.45265E-05 | 6.71726E-05 | 8.67204E-05 | 0.000105541 | 9.31862E-05 | 9.93634E-05 |
| 590 | Glycitin | 40246-10-4 | C16195 | C22H22O10 | 446.12130000000002 | flavonoids | 447.4 | 284.9 | + | 5.57 | 0.000192502 | 0.000302219 | 0.000100197 | 0.000198306 | 0.000230367 | 0.00014105 | 0.000136278 | 0.000169232 | 0.000199707 | 0.000176934 | 0.00018832 |
| 591 | Glycyrrhetic acid 3-O-mono-beta-D-glucuronide | 34096-83-8 |  | C36H54O10 | 646.37170000000003 | Triterpenoids | 647.4 | 453.3 | + | 14.02723333 | 0.000131351 | 1.27649E-06 | 9.03E-05 | 7.43092E-05 | 0.000268485 | 0.000159133 | 1.23455E-06 | 0.000142951 | 8.85743E-05 | 8.18409E-05 | 8.52076E-05 |
| 592 | Glycyrrhetinic acid | 471-53-4 |  | C30H46O4 | 470.33960999999999 | Triterpenoids | 471.3 | 189.2 | + | 9.227716667 | 6.20453E-05 | 8.71815E-05 | 4.66841E-05 | 6.53036E-05 | 0.000118258 | 6.28905E-05 | 5.33399E-05 | 7.81629E-05 | 6.57111E-05 | 2.71925E-05 | 4.64518E-05 |
| 593 | Glycyrrhetinic acid;Eburicoic acid | 1449-05-4;471-53-4;560-66-7 | C02283;C14495 | C30H46O4;C31H50O3 | 470.33960999999999;470.37599499999999 | Triterpenoids | 471.35 | 407.35 | + | 10.57346667 | 6.60387E-05 | 1.97811E-05 | 9.1155E-06 | 3.16451E-05 | 2.15676E-05 | 8.57307E-05 | 4.10491E-05 | 4.94491E-05 | 2.58382E-05 | 5.52502E-05 | 4.05442E-05 |
| 594 | Goshonoside F1 | 90851-24-4 |  | C26H44O8 | 484.30362000000002 | Diterpenoids | 485.3 | 449.3 | + | 12.30211667 | 0.000305754 | 0.000200504 | 0.00069484 | 0.000400366 | 8.48534E-05 | 0.000124693 | 0.000191415 | 0.000133654 | 0.000141588 | 0.000294354 | 0.000217971 |
| 595 | Gramine | 87-52-5 | C08304 | C11H14N2 | 174.11569800000001 | Alkaloids | 175.1 | 130.1 | + | 8.72735 | 9.75103E-05 | 0.000156067 | 0.00011502 | 0.000122866 | 0.000263085 | 0.000111328 | 0.000107355 | 0.000160589 | 0.00013498 | 0.000154995 | 0.000144988 |
| 596 | Grandifloric acid;Steviol | 22338-69-8;471-80-7 | C17956;C20212 | C20H30O3 | 318.21949499999999 | Diterpenoids | 319.2 | 255.2 | + | 6.47845 | 0.000630771 | 0.000230449 | 0.000452948 | 0.000438056 | 0.000316745 | 0.000289307 | 0.000363572 | 0.000323208 | 0.000151497 | 0.00021826 | 0.000184878 |
| 597 | Grossamide | 80510-06-1 |  | C36H36N2O8 | 624.24716799999999 | Alkaloids | 625.3 | 121.1 | + | 13.08188333 | 0.002010866 | 0.001595682 | 0.000757869 | 0.001454806 | 0.002824451 | 0.002038822 | 0.002264883 | 0.002376052 | 0.003018681 | 0.001575892 | 0.002297287 |
| 598 | Guaiacin | 36531-08-5 |  | C20H24O4 | 328.16746000000001 | Lignans | 329.2 | 297.1 | + | 7.535766667 | 4.90767E-05 | 3.05019E-05 | 7.80549E-05 | 5.25445E-05 | 6.86832E-05 | 4.32706E-05 | 2.55429E-05 | 4.58322E-05 | 3.72827E-05 | 5.7096E-05 | 4.71893E-05 |
| 599 | Guaiacol | 90-05-1 | C15572;C01502 | C7H8O2 | 124.05243 | Phenols | 125.1 | 65 | + | 5.622516667 | 0.000307134 | 0.000219989 | 0.000258104 | 0.000261742 | 0.000375401 | 0.000355024 | 0.000462007 | 0.000397477 | 0.000253166 | 0.000430882 | 0.000342024 |
| 600 | Guaiazulene | 489-84-9 | C09675 | C15H18 | 198.14085 | Sesquiterpenoids | 199.1 | 183.1 | + | 8.744133333 | 8.25233E-05 | 0.000162604 | 0.000150804 | 0.000131977 | 0.000104025 | 0.000139103 | 0.000119209 | 0.000120779 | 9.34052E-05 | 9.86795E-05 | 9.60423E-05 |
| 601 | Guanidineacetic acid | 352-97-6 | C00581 | C3H7N3O2 | 117.053827 | Amino acid and derivatives | 118.1 | 43 | + | 1.107916667 | 0.000193248 | 0.000439555 | 0.000335154 | 0.000322652 | 0.00033241 | 0.000234193 | 0.000314889 | 0.00029383 | 0.000212825 | 0.000343114 | 0.00027797 |
| 602 | Guanosine | 118-00-3 | C00387 | C10H13N5O5 | 283.09167000000002 | Nucleotide and its derivates | 284.1 | 152.1 | + | 2.65195 | 0.001223988 | 0.001537348 | 0.000737596 | 0.001166311 | 0.014841578 | 0.001364931 | 0.001438901 | 0.005881803 | 0.138370807 | 0.121625777 | 0.129998292 |
| 603 | Guanosine 3',5'-cyclic monophosphate | 7665-99-8 | C00942 | C10H12N5O7P | 345.047437 | Nucleotide and its derivates | 346.1 | 152.1 | + | 2.25995 | 0.001258093 | 0.001372406 | 0.001124473 | 0.001251657 | 0.000996473 | 0.000788945 | 0.000841586 | 0.000875668 | 0.002310514 | 0.001334875 | 0.001822695 |
| 604 | Guggulsterone E&Z | 95975-55-6 |  | C22H30O3 | 342.21949499999999 | Steroids | 343.2 | 325.2 | + | 11.07695 | 0.000162898 | 5.29806E-05 | 0.000152368 | 0.000122749 | 9.51714E-05 | 8.48425E-05 | 0.000149279 | 0.000109764 | 9.24884E-05 | 0.000140895 | 0.000116692 |
| 605 | Hamamelitannin | 469-32-9 |  | C20H20O14 | 484.08530999999999 | Phenols | 485.1 | 153 | + | 12.28533333 | 0.00014407 | 0.000217783 | 0.000209521 | 0.000190458 | 0.00014804 | 0.000164427 | 0.000157412 | 0.000156626 | 0.000281188 | 0.000219211 | 0.000250199 |
| 606 | Harmaline | 304-21-2 | C06536 | C13H14N2O | 214.110613 | Alkaloids | 215.1 | 172.1 | + | 10.00223333 | 0.000217893 | 0.000365549 | 0.000289631 | 0.000291025 | 0.000418522 | 0.000280836 | 0.000236212 | 0.000311857 | 0.000265181 | 0.000295373 | 0.000280277 |
| 607 | Harman | 486-84-0 | C09209 | C12H10N2 | 182.08439799999999 | Alkaloids | 183.1 | 140 | + | 8.5971 | 3.93342E-05 | 5.04855E-05 | 0.000105338 | 6.50526E-05 | 3.93378E-05 | 5.80957E-05 | 7.51254E-05 | 5.75196E-05 | 7.77687E-05 | 4.0673E-05 | 5.92209E-05 |
| 608 | Harpagoside | 19210-12-9 | C09783 | C24H30O11 | 494.17881499999999 | Iridoids | 495.2 | 167.1 | + | 6.586816667 | 4.39546E-05 | 4.102E-05 | 5.94717E-05 | 4.81488E-05 | 7.75285E-05 | 2.75078E-05 | 1.93883E-05 | 4.14748E-05 | 7.8093E-05 | 0.000100118 | 8.91054E-05 |
| 609 | Hesperetin | 520-33-2 | C01709 | C16H14O6 | 302.07900000000001 | flavonoids | 303.3 | 178.9 | + | 9.2 | 0.000158066 | 9.51004E-05 | 8.77346E-05 | 0.000113634 | 0.000126881 | 9.74077E-05 | 9.50897E-05 | 0.000106459 | 4.89433E-05 | 9.01297E-05 | 6.95365E-05 |
| 610 | Hesperetin 7-O-glucoside | 31712-49-9 | C16422 | C22H24O11 | 464.131865 | Flavonoids | 465.1 | 285.1 | + | 6.067633333 | 0.000135248 | 0.00021123 | 0.00022592 | 0.000190799 | 0.000231203 | 0.000248853 | 0.000338475 | 0.000272844 | 0.000352338 | 0.00015974 | 0.000256039 |
| 611 | Hesperidin | 520-26-3 | C09755 | C28H34O15 | 610.18979999999999 | flavonoids | 609 | 300.8 | - | 6.65 | 0.00554389 | 0.004677513 | 0.005395751 | 0.005205718 | 0.000198198 | 2.76081E-05 | 4.67935E-05 | 9.08664E-05 | 0.002554677 | 0.002189 | 0.002371838 |
| 612 | Hinokiflavone | 19202-36-9 | C10057 | C30H18O10 | 538.09 | Flavonoids | 539.1 | 521.1 | + | 11.93288333 | 0.001972201 | 0.001476053 | 0.001305562 | 0.001584605 | 0.00098144 | 0.000802358 | 0.001017835 | 0.000933878 | 0.002378322 | 0.002233833 | 0.002306078 |
| 613 | Homobaldrinal | 67910-07-0 | C16812 | C15H16O4 | 260.10485999999997 | Miscellaneous | 261.1 | 159 | + | 10.00285 | 0.003570441 | 0.003200859 | 0.003711155 | 0.003494151 | 0.000124081 | 0.000246914 | 0.000144164 | 0.00017172 | 0.001262263 | 0.001412335 | 0.001337299 |
| 614 | Homodihydrocapsaicin I | 20279-06-5 |  | C19H31NO3 | 321.23039399999999 | Miscellaneous | 322.2 | 137.1 | + | 13.30908333 | 0.000227712 | 0.000307407 | 7.09933E-05 | 0.000202037 | 0.000199554 | 0.000183445 | 0.000214781 | 0.00019926 | 0.000257388 | 0.00013141 | 0.000194399 |
| 615 | Homoeriodictyol | 446-71-9 | C09756 | C16H14O6 | 302.07904000000002 | Flavonoids | 303.1 | 153 | + | 6.830883333 | 0.017729611 | 0.012999636 | 0.020200828 | 0.016976692 | 0.000274005 | 0.000300126 | 0.000186482 | 0.000253538 | 0.006108909 | 0.007816211 | 0.00696256 |
| 616 | Homoferreirin | 482-01-9 | C10457 | C17H16O6 | 316.09469000000001 | Flavonoids | 317.1 | 153 | + | 8.0896 | 7.01748E-05 | 7.0026E-05 | 5.99241E-05 | 6.67083E-05 | 9.39058E-05 | 6.69432E-05 | 4.37717E-05 | 6.82069E-05 | 8.27258E-05 | 6.18185E-05 | 7.22721E-05 |
| 617 | Homoorientin | 4261-42-1 | C01821 | C21H20O11 | 448.10059999999999 | flavonoids | 449 | 430.9 | + | 5.43 | 0.000599779 | 0.000185209 | 0.000374142 | 0.000386377 | 9.22776E-05 | 7.93105E-05 | 0.000327491 | 0.00016636 | 0.000340968 | 0.000101929 | 0.000221448 |
| 618 | Homopterocarpin | 606-91-7 |  | C17H16O4 | 284.10485999999997 | Flavonoids | 285.1 | 107 | + | 9.074433333 | 0.000211082 | 0.000369772 | 0.000279225 | 0.000286693 | 0.000191424 | 0.000492298 | 0.000227107 | 0.000303609 | 0.000222999 | 0.00034819 | 0.000285594 |
| 619 | Hordenine | 539-15-1 | C06199 | C10H15NO | 165.115364 | Alkaloids | 166.1 | 121.1 | + | 3.03795 | 7.23665E-05 | 0.000101104 | 6.35078E-05 | 7.89926E-05 | 0.000211547 | 0.0001575 | 0.000139787 | 0.000169611 | 6.15737E-05 | 9.74289E-05 | 7.95013E-05 |
| 620 | Huperzine B | 103548-82-9 | C09866 | C16H20N2O | 256.15756299999998 | Alkaloids | 257.2 | 198.1 | + | 3.829592465 | 6.56761E-05 | 3.1237E-05 | 9.53628E-05 | 6.40919E-05 | 4.44375E-05 | 4.25273E-05 | 3.4887E-05 | 4.06173E-05 | 4.91529E-05 | 1.64268E-05 | 3.27898E-05 |
| 621 | Hydrangenol | 480-47-7 | C10262 | C15H12O4 | 256.07355999999999 | Flavonoids | 257.1 | 105 | + | 11.31191667 | 0.015256295 | 0.009562889 | 0.014145182 | 0.012988122 | 0.021126734 | 0.013824265 | 0.017302788 | 0.017417929 | 0.015894049 | 0.01844445 | 0.017169249 |
| 622 | Hydroxysafflor yellow A | 78281-02-4 |  | C27H32O16 | 612.16904 | Chalcones | 613.2 | 595.2 | + | 12.58741667 | 0.014167117 | 0.135815977 | 0.098608567 | 0.082863887 | 0.161721289 | 0.03788697 | 0.079545223 | 0.09305116 | 0.18107739 | 0.163919403 | 0.172498396 |
| 623 | Hygromycin B | 31282-04-9 | C01925 | C20H37N3O13 | 527.23264200000006 | Organooxygen compounds | 528.2 | 177.1 | + | 4.479333333 | 0.000483584 | 0.00019663 | 0.000285313 | 0.000321842 | 0.000352252 | 9.09158E-05 | 0.00031133 | 0.000251499 | 0.000420349 | 0.000252408 | 0.000336379 |
| 624 | Hypotaurine | 300-84-5 | C00519 | C2H7NO2S | 109.01975 | Alkaloids | 110 | 65 | + | 2.24245 | 0.000107045 | 0.000359671 | 0.00015197 | 0.000206228 | 0.000176481 | 0.000136627 | 0.000259825 | 0.000190978 | 0.000224631 | 0.000145092 | 0.000184861 |
| 625 | Imidacloprid | 138261-41-3 | C11110 | C9H10ClN5O2 | 255.05230299999999 | Organonitrogen compounds | 254 | 46 | - | 0.66345 | 5.13397E-06 | 1.10391E-05 | 6.57187E-05 | 2.72972E-05 | 1.6676E-05 | 0.000101914 | 1.14802E-05 | 4.33568E-05 | 4.02555E-05 | 2.13217E-05 | 3.07886E-05 |
| 626 | Indaconitine | 4491-19-4 | C08691 | C34H47NO10 | 629.31999900000005 | Alkaloids | 630.3 | 105 | + | 12.00276667 | 0.001322567 | 0.000708305 | 0.001330638 | 0.001120504 | 0.001758926 | 0.001386019 | 0.001520412 | 0.001555119 | 0.001208192 | 0.001266785 | 0.001237489 |
| 627 | Indole | 120-72-9 | C00463 | C8H7N | 117.057849 | Alkaloids | 118.1 | 91.1 | + | 3.81955 | 0.047061243 | 0.00177196 | 0.003317975 | 0.017383726 | 0.061499632 | 0.055700759 | 0.043524062 | 0.053574818 | 0.045166576 | 0.055073483 | 0.050120029 |
| 628 | Indole-3-acetic acid | 87-51-4 | C00954 | C10H9NO2 | 175.0633 | phytohormone | 176 | 130 | + | 7.14 | 0.001312571 | 0.000966392 | 0.000730404 | 0.001003122 | 0.000897038 | 0.000814216 | 0.000705364 | 0.000805539 | 0.000991488 | 0.001041027 | 0.001016257 |
| 629 | Indole-3-carboxaldehyde | 487-89-8 | C08493 | C9H7NO | 145.05279999999999 | phytohormone | 146 | 118 | + | 6.73 | 0.044068421 | 0.057134136 | 0.04445381 | 0.048552123 | 0.063460197 | 0.049990901 | 0.047758913 | 0.05373667 | 0.064957025 | 0.058040392 | 0.061498708 |
| 630 | Indolepyruvate | 392-12-1 | C00331 | C11H9NO3 | 203.058244 | Indoles and derivatives | 202.1 | 128 | - | 3.028433333 | 1.18251E-06 | 3.15876E-05 | 5.37599E-06 | 1.27154E-05 | 3.45795E-06 | 8.64881E-06 | 2.81072E-05 | 1.34047E-05 | 5.95081E-05 | 1.08743E-05 | 3.51912E-05 |
| 631 | Inosine 5'-monophosphate;Inosinic acid | 131-99-7 | C00130 | C10H13N4O8P | 348.04710299999999 | Nucleotide and its derivates | 347 | 79 | - | 1.5555 | 2.23456E-05 | 0.00011396 | 8.20436E-06 | 4.81701E-05 | 0.000115858 | 5.09137E-05 | 3.83177E-05 | 6.8363E-05 | 5.06035E-05 | 6.34282E-05 | 5.70158E-05 |
| 632 | Inositol | 643-10-7;87-89-8 | C00137 | C6H12O6 | 180.06339 | Organooxygen compounds | 181.1 | 163.1 | + | 9.935716667 | 0.008699168 | 0.00929341 | 0.007287336 | 0.008426638 | 0.01565158 | 0.010175485 | 0.010850678 | 0.012225914 | 0.010846186 | 0.008703376 | 0.009774781 |
| 633 | Ipecoside | 15401-60-2 | C09464 | C27H35NO12 | 565.21592899999996 | Iridoids | 566.2 | 548.2 | + | 12.69713333 | 0.005675736 | 0.002966096 | 0.002562544 | 0.003734792 | 0.004511683 | 0.001940215 | 0.001760491 | 0.002737463 | 0.003582432 | 0.002491844 | 0.003037138 |
| 634 | Irisflorentin | 41743-73-1 | C17958 | C20H18O8 | 386.10016999999999 | Flavonoids | 387.1 | 167.1 | + | 8.643433333 | 0.000134244 | 2.64317E-05 | 0.000160587 | 0.000107088 | 0.000169581 | 0.00015474 | 7.1206E-05 | 0.000131842 | 0.000100273 | 8.57199E-05 | 9.29966E-05 |
| 635 | Isatidine | 15503-86-3 | C10333 | C18H25NO7 | 367.16310399999998 | Alkaloids | 368.2 | 350.2 | + | 6.730183333 | 8.71015E-05 | 8.61844E-05 | 6.09425E-05 | 7.80761E-05 | 5.72312E-05 | 0.000185108 | 0.000139182 | 0.000127174 | 0.000162275 | 0.000122738 | 0.000142507 |
| 636 | Isoarnebin I;Dimethylacrylshikonin | 5162-01-6;24502-79-2 | C10293;C17415 | C21H22O6 | 370.14164 | Quinones | 371.1 | 55.1 | + | 7.770733333 | 6.16337E-05 | 2.98878E-05 | 5.16475E-05 | 4.7723E-05 | 7.09896E-05 | 4.94783E-05 | 6.91204E-06 | 4.246E-05 | 4.14442E-05 | 5.43451E-05 | 4.78947E-05 |
| 637 | Isobergapten | 482-48-4 | C18082 | C12H8O4 | 216.04226 | Coumarins | 217 | 202 | + | 9.937166667 | 0.003781523 | 0.004147487 | 0.004934385 | 0.004287798 | 0.000384737 | 0.000235627 | 0.000125711 | 0.000248691 | 0.001838302 | 0.001684419 | 0.00176136 |
| 638 | Isochlorogenic acid B;(1S,3R,4R,5R)-3,4-bis[[(E)-3-(3,4-dihydroxyphenyl)prop-2-enoyl]oxy]-1,5-dihydroxycyclohexane-1-carboxylic acid;3,4-Di-O-caffeoylquinic acid | 14534-61-3 | C10468 | C25H24O12 | 516.12678000000005 | Phenylpropanoids | 517.1 | 121 | + | 6.617503667 | 0.000343836 | 0.000267741 | 0.000522888 | 0.000378155 | 0.000116688 | 0.000349006 | 0.000142952 | 0.000202882 | 0.000457951 | 8.73128E-05 | 0.000272632 |
| 639 | Isodiospyrin | 20175-84-2 | C10358 | C22H14O6 | 374.07904000000002 | Quinones | 375.1 | 357.1 | + | 11.27835 | 0.000142669 | 0.001133521 | 0.00100733 | 0.000761173 | 0.00092186 | 0.00080961 | 0.000177195 | 0.000636221 | 0.001005996 | 0.000573682 | 0.000789839 |
| 640 | Isofraxidin | 486-21-5 | C17480 | C11H10O5 | 222.05282500000001 | Phenylpropanoids | 223.1 | 205.1 | + | 4.249083333 | 8.47451E-05 | 0.00011014 | 8.81598E-05 | 9.43483E-05 | 0.000276457 | 0.00011413 | 5.74067E-05 | 0.000149331 | 8.32459E-05 | 9.4347E-05 | 8.87965E-05 |
| 641 | Isogentisin | 491-64-5 | C10070 | C14H10O5 | 258.05282499999998 | Xanthones | 259.1 | 241.1 | + | 7.75395 | 4.30985E-05 | 0.000140758 | 0.000140194 | 0.000108017 | 0.000176822 | 0.000209719 | 9.70071E-05 | 0.000161183 | 7.45012E-05 | 4.35608E-05 | 5.9031E-05 |
| 642 | Isoleucine | 443-79-8 | C16434;C06418 | C6H13NO2 | 131.094629 | Amino acid and derivatives | 132.1 | 86.1 | + | 1.796016667 | 0.011647802 | 0.008250086 | 0.0072977 | 0.009065196 | 0.003270213 | 0.003943126 | 0.006829968 | 0.004681103 | 0.008090435 | 0.0081886 | 0.008139517 |
| 643 | isoliquiritigenin | 961-29-5 | C08650 | C15H12O4 | 256.0736 | flavonoids | 256.9 | 211 | + | 9.73 | 9.11609E-05 | 0.000175537 | 6.10186E-05 | 0.000109239 | 0.000171271 | 6.643E-05 | 0.000154817 | 0.000130839 | 7.8949E-05 | 5.41916E-05 | 6.65703E-05 |
| 644 | Isoliquiritin; Isoliquiritoside | 5041-81-6 | C16978 | C21H22O9 | 418.12638500000003 | Chalcones | 419.1 | 239.1 | + | 6.9421 | 8.11882E-05 | 0.000107557 | 0.000107808 | 9.88508E-05 | 5.78418E-05 | 8.8945E-05 | 9.18009E-05 | 7.95292E-05 | 5.95242E-05 | 0.000143683 | 0.000101603 |
| 646 | Isoquercitrin | 482-35-9 | C05623 | C21H20O12 | 464.09550000000002 | flavonoids | 465.4 | 302.8 | + | 6.06 | 0.000552441 | 0.000970994 | 0.00328575 | 0.001603062 | 0.001151534 | 0.000918118 | 0.000936984 | 0.001002212 | 0.000779742 | 0.000377479 | 0.000578611 |
| 647 | Isorhamnetin | 480-19-3 | C10084 | C16H12O7 | 316.05830500000002 | Flavonoids | 317.1 | 153 | + | 9.23 | 5.79327E-05 | 0.000103323 | 0.000119542 | 9.35992E-05 | 8.36958E-05 | 0.000212415 | 5.01323E-05 | 0.000115414 | 5.063E-05 | 8.98466E-05 | 7.02383E-05 |
| 648 | Isorubrofusarin 10-gentiobioside;Rubrofusarin-6-O-beta-D-gentiobioside;Cassiaside C | 200127-93-1;24577-90-0;119170-52-4 | | C27H32O15 | 596.174125 | Phenols | 597.2 | 255.1 | + | 12.83916667 | 0.001024049 | 0.001141646 | 0.001794194 | 0.001319963 | 0.002014417 | 0.001628365 | 0.001232691 | 0.001625158 | 0.002800685 | 0.002217202 | 0.002508944 |
| 649 | Isotetrandrine | 477-57-6 | C17060 | C38H42N2O6 | 622.30428800000004 | Alkaloids | 623.3 | 578.3 | + | 12.18463333 | 6.97647E-05 | 4.21856E-05 | 1.12648E-06 | 3.76923E-05 | 0.000198018 | 0.000149797 | 0.000128728 | 0.000158848 | 0.000151779 | 3.07251E-05 | 9.12521E-05 |
| 650 | Isovitexin | 38953-85-4 | C01714 | C21H20O10 | 432.10565000000003 | Flavonoids | 433.1 | 415.1 | + | 5.854966667 | 0.00017377 | 0.000149406 | 0.00011421 | 0.000145795 | 0.000595441 | 0.000172762 | 0.000510653 | 0.000426285 | 0.000171824 | 0.000475991 | 0.000323907 |
| 651 | Isoxanthopterin | 529-69-1 | C03975 | C6H5N5O2 | 179.04432499999999 | Pteridines and derivatives | 180.1 | 135 | + | 3.256133333 | 0.000949194 | 0.001085246 | 0.001010231 | 0.00101489 | 0.001387601 | 0.000960535 | 0.00095277 | 0.001100302 | 0.000873742 | 0.001045646 | 0.000959694 |
| 652 | Itaconic acid | 97-65-4 | C00490 | C5H6O4 | 130.02661000000001 | Fatty Acyls | 131 | 85 | + | 0.520516667 | 0.00984759 | 0.006089957 | 0.009716138 | 0.008551228 | 0.013501823 | 0.009533614 | 0.009850088 | 0.010961842 | 0.009183249 | 0.009771182 | 0.009477216 |
| 653 | Jasmonic acid | 6894-38-8 | C08491 | C12H18O3 | 210.125595 | Fatty Acyls | 211.1 | 151.1 | + | 6.729016667 | 0.000175929 | 0.000114823 | 0.000290367 | 0.000193706 | 0.000118302 | 0.000101935 | 0.000144896 | 0.000121711 | 9.72272E-05 | 0.000121254 | 0.00010924 |
| 654 | Jervine | 469-59-0 | C10811 | C27H39NO3 | 425.29299400000002 | Alkaloids | 426.3 | 408.3 | + | 10.52311667 | 0.000121137 | 0.000184428 | 6.58457E-05 | 0.000123803 | 5.19675E-05 | 1.60859E-05 | 8.57818E-05 | 5.12784E-05 | 8.47385E-05 | 0.000109002 | 9.68701E-05 |
| 655 | Juglone | 481-39-0 | C03840 | C10H6O3 | 174.03169500000001 | Quinones | 175 | 147 | + | 8.741533333 | 0.000244484 | 0.00010403 | 0.00013759 | 0.000162034 | 0.000214398 | 0.000120024 | 0.000193808 | 0.000176077 | 0.000193383 | 0.000172867 | 0.000183125 |
| 656 | Kadsurin A | 99340-07-5 | C10640 | C21H24O6 | 372.15728999999999 | Lignans | 373.2 | 135 | + | 11.39106667 | 0.000190059 | 0.00021108 | 9.77584E-05 | 0.000166299 | 0.000140429 | 0.000464605 | 0.000191226 | 0.00026542 | 0.000274746 | 0.000193406 | 0.000234076 |
| 657 | Kaempferitrin | 482-38-2 | C16981 | C27H30O14 | 578.16359999999997 | flavonoids | 577.5 | 431.7 | - | 6.09 | 0.004165335 | 0.003265739 | 0.003747678 | 0.003726251 | 0.004619703 | 0.004260582 | 0.005295994 | 0.004725426 | 0.004063342 | 0.004871721 | 0.004467531 |
| 658 | Kaempferol | 520-18-3 | C05903 | C15H10O6 | 286.04773999999998 | Flavonoids | 287.1 | 153 | + | 9.390023745 | 7.19444E-05 | 6.09793E-05 | 5.73188E-05 | 6.34141E-05 | 0.000103387 | 5.73332E-05 | 0.000156984 | 0.000105902 | 5.55949E-05 | 6.99899E-05 | 6.27924E-05 |
| 659 | Karanjin | 521-88-0 |  | C18H12O4 | 292.07355999999999 | Flavonoids | 293.1 | 105 | + | 11.6673 | 0.009750742 | 0.011142258 | 0.010794736 | 0.010562579 | 0.012472972 | 0.010541754 | 0.01145687 | 0.011490532 | 0.013594982 | 0.0081037 | 0.010849341 |
| 660 | Kaurenoic acid | 6730-83-2 | C11874 | C20H30O2 | 302.22458 | Diterpenoids | 303.2 | 257.2 | + | 5.902316667 | 0.0006546 | 0.00060277 | 0.000521953 | 0.000593107 | 0.001063631 | 0.000575461 | 0.00064918 | 0.000762757 | 0.000868648 | 0.000483186 | 0.000675917 |
| 661 | Kazinol A | 99624-28-9 | C09760 | C25H30O4 | 394.21440999999999 | Flavonoids | 395.2 | 123 | + | 13.25873333 | 0.000254781 | 0.000227987 | 0.000457667 | 0.000313478 | 0.000230825 | 0.000382077 | 0.000436591 | 0.000349831 | 0.000200804 | 0.000233932 | 0.000217368 |
| 662 | Ketopantoic acid | 470-30-4 | C00966 | C6H10O4 | 146.05790999999999 |  | 147.1 | 73.1 | + | 0.698866667 | 0.02710352 | 0.019007737 | 0.030739385 | 0.025616881 | 0.050035288 | 0.025412205 | 0.031861481 | 0.035769658 | 0.036139453 | 0.035334725 | 0.035737089 |
| 663 | Kirenol | 52659-56-0 |  | C20H34O4 | 338.24570999999997 | Diterpenoids | 321.2 | 81.1 | + | 13.15803333 | 0.012446861 | 0.011705926 | 0.002601307 | 0.008918031 | 0.032518013 | 0.052989959 | 0.024647463 | 0.036718478 | 0.049584111 | 0.046351191 | 0.047967651 |
| 664 | Knightinol | 77053-06-6 | C10859 | C17H23NO3 | 289.16779400000001 | Alkaloids | 290.2 | 230.2 | + | 5.958183333 | 0.000120336 | 0.000128157 | 1.78351E-05 | 8.8776E-05 | 8.65446E-05 | 4.45725E-05 | 3.84488E-05 | 5.6522E-05 | 9.06212E-05 | 0.000250427 | 0.000170524 |
| 665 | Koaburaside monomethyl ether | 41514-64-1 |  | C15H22O9 | 346.12638500000003 | Phenols | 347.1 | 185.1 | + | 11.64808333 | 0.000198433 | 0.000342617 | 0.00032342 | 0.000288157 | 0.000946645 | 0.000200975 | 0.000570687 | 0.000572769 | 0.00049745 | 0.000467575 | 0.000482513 |
| 666 | Koenigicine | 24123-92-0 |  | C20H21NO3 | 323.15214400000002 | Alkaloids | 324.2 | 266.1 | + | 6.5288 | 3.85782E-05 | 0.000129497 | 2.47687E-05 | 6.42812E-05 | 0.000157156 | 0.00015185 | 0.000156425 | 0.000155144 | 0.000164754 | 3.06511E-05 | 9.77023E-05 |
| 667 | Kojibiose | 2140-29-6 | C19632 | C12H22O11 | 342.11621500000001 | Fatty Acyls | 325.1 | 85 | + | 0.721916667 | 0.0033474 | 0.00452438 | 0.00243942 | 0.003437066 | 0.003404909 | 0.002158419 | 0.002125322 | 0.002562883 | 0.001518395 | 0.004255786 | 0.00288709 |
| 668 | Kuwanon H | 76472-87-2 | C10100 | C45H44O11 | 760.288365 | Flavonoids | 761.3 | 743.3 | + | 12.03583333 | 0.00054327 | 0.000871487 | 0.001017002 | 0.000810586 | 0.00081582 | 0.000711706 | 0.000406836 | 0.000644787 | 0.000894275 | 0.000506084 | 0.00070018 |
| 671 | L-Arginine | 74-79-3 | C00062 | C6H14N4O2 | 174.11167599999999;174.11170000000001 | Amino acid and derivatives;amino acids | 175.1 | 70.05 | + | 0.6 | 0.012550027 | 0.012990712 | 0.011526576 | 0.012355772 | 0.017335461 | 0.01297985 | 0.01354847 | 0.01462126 | 0.01418594 | 0.015379915 | 0.014782927 |
| 672 | L-Asparagine | 70-47-3 | C00152 | C4H8N2O3 | 132.053493;132.05350000000001 | Amino acid and derivatives;amino acids | 133.1 | 74 | + | 0.64 | 0.000299217 | 0.00026287 | 0.000345902 | 0.000302663 | 0.000707669 | 0.000436107 | 0.000539175 | 0.000560983 | 0.000308057 | 0.000617082 | 0.00046257 |
| 673 | L-Aspartic acid | 56-84-8 | C00049 | C4H7NO4 | 133.037509;133.03749999999999 | Amino acid and derivatives;amino acids | 134 | 74 | + | 0.63 | 0.000364877 | 0.000608344 | 0.001165654 | 0.000712959 | 0.001100325 | 0.00075857 | 0.00117911 | 0.001012668 | 0.000257499 | 0.000803564 | 0.000530531 |
| 674 | L-Citruline;L-Citrulline | 372-75-8 | C00327 | C6H13N3O3 | 175.09569200000001;175.09569999999999 | Amino acid and derivatives;amino acids | 176.1 | 70.1 | + | 0.66 | 0.006491349 | 0.001090369 | 0.006485486 | 0.004689068 | 0.012435312 | 0.007612428 | 0.007629376 | 0.009225705 | 0.007811458 | 0.008266146 | 0.008038802 |
| 675 | L-Cysteine | 52-90-4 | C00097 | C3H7NO2S | 121.01975 | Amino acid and derivatives | 122 | 59 | + | 4.4615 | 5.06417E-05 | 4.55092E-05 | 2.0427E-05 | 3.88593E-05 | 6.46709E-05 | 2.9967E-05 | 5.16695E-05 | 4.87691E-05 | 4.85856E-05 | 6.74329E-05 | 5.80093E-05 |
| 676 | L-Glutamic acid | 56-86-0 | C00025 | C5H9NO4 | 147.05315899999999;147.0532 | Amino acid and derivatives;amino acids | 148.1 | 84 | + | 0.66 | 0.0387896 | 0.050308018 | 0.045168687 | 0.044755435 | 0.057786391 | 0.040584292 | 0.046005846 | 0.04812551 | 0.039081165 | 0.041422044 | 0.040251605 |
| 677 | L-Gulose | 6027-89-0 | C15923 | C6H12O6 | 180.06339 | Carbohydrates | 203.1 | 81.1 | + | 8.711066667 | 0.000134067 | 0.000372282 | 0.000188785 | 0.000231711 | 0.000449626 | 0.000227359 | 0.000149142 | 0.000275375 | 0.000122009 | 0.000165157 | 0.000143583 |
| 678 | L-Histidine | 71-00-1 | C00135 | C6H9N3O2 | 155.06947700000001;155.06950000000001 | Amino acid and derivatives;amino acids | 156.1 | 110.1 | + | 0.59 | 0.003130582 | 0.002670032 | 0.001294492 | 0.002365035 | 0.002941754 | 0.00413419 | 0.002850162 | 0.003308702 | 0.002025635 | 0.003845521 | 0.002935578 |
| 679 | L-Homoglutamic acid | 542-32-5;1118-90-7 | C00956 | C6H11NO4 | 161.06880899999999 | Amino acid and derivatives | 162.1 | 98.1 | + | 0.728116667 | 0.00018428 | 0.000411017 | 0.000178435 | 0.000257911 | 0.000303914 | 0.000218627 | 0.000260175 | 0.000260905 | 0.000318174 | 0.000201233 | 0.000259704 |
| 680 | L-Homoserine | 1927-25-9;672-15-1;6027-21-0 | C00263 | C4H9NO3 | 119.058244 | Amino acid and derivatives | 120.1 | 56 | + | 0.671183333 | 0.012091 | 0.019395135 | 0.022472573 | 0.017986236 | 0.011348621 | 0.01263711 | 0.007699714 | 0.010561815 | 0.016308201 | 0.013619137 | 0.014963669 |
| 681 | L-Isoleucine;L-Leucine | 73-32-5;61-90-5 | C00407;C00123 | C6H13NO2 | 131.094629 | Amino acid and derivatives | 132.1 | 86.1 | + | 1.779341667 | 0.008857563 | 0.009294434 | 0.0072977 | 0.008483232 | 0.003270213 | 0.003943126 | 0.006829968 | 0.004681103 | 0.008090435 | 0.0081886 | 0.008139517 |
| 682 | L-Kynurenine | 343-65-7;2922-83-0 | C00328;C01718 | C10H12N2O3 | 208.08479299999999 | Amino acid and derivatives | 209.1 | 94.1 | + | 1.980633333 | 5.30607E-05 | 8.72847E-05 | 1.7731E-05 | 5.26921E-05 | 4.11909E-05 | 0.000106412 | 1.01722E-05 | 5.25917E-05 | 4.29366E-05 | 0.000105298 | 7.41174E-05 |
| 683 | L-Lysine;L-Glutamine | 56-87-1;56-85-9 | C00047;C00064 | C6H14N2O2;C5H10N2O3 | 146.10552799999999;146.06909999999999;146.10550000000001 | Amino acid and derivatives;amino acids | 147.1 | 84.06666667 | + | 0.64 | 0.056735084 | 0.055211896 | 0.066858713 | 0.059601898 | 0.147326026 | 0.087059499 | 0.092189308 | 0.108858278 | 0.099472557 | 0.081517331 | 0.090494944 |
| 684 | L-Malic acid | 97-67-6 | C00149 | C4H6O5 | 134.021525 | Hydroxy acids and derivatives | 157 | 110.1 | + | 1.37645 | 4.30783E-05 | 2.52459E-05 | 3.66644E-05 | 3.49962E-05 | 7.37537E-05 | 2.80071E-05 | 3.27693E-05 | 4.48434E-05 | 3.26579E-05 | 4.23115E-05 | 3.74847E-05 |
| 686 | L-Nicotine | 54-11-5 | C00745 | C10H14N2 | 162.11569800000001 | Alkaloids | 163.1 | 130.1 | + | 2.294983333 | 0.00040855 | 0.000383275 | 0.000489079 | 0.000426968 | 0.000375024 | 0.000271946 | 0.000252461 | 0.00029981 | 0.000520358 | 0.000379458 | 0.000449908 |
| 687 | L-Norleucine | 327-57-1 | C01933 | C6H13NO2 | 131.094629 | Amino acid and derivatives | 132.1 | 86.1 | + | 1.8128 | 0.011647802 | 0.008250086 | 0.0072977 | 0.009065196 | 0.003270213 | 0.003943126 | 0.006829968 | 0.004681103 | 0.008090435 | 0.0081886 | 0.008139517 |
| 689 | L-Phenylalanine;D-(+)-Phenylalanine;DL-Phenylalanine | 63-91-2;673-06-3;150-30-1 | C00079;C02265;C02057 | C9H11NO2 | 165.078979;165.07900000000001 | Amino acid and derivatives;Phenols;amino acids | 166.1 | 120.1 | + | 2.819775 | 0.522356509 | 0.515505904 | 0.481029511 | 0.506297308 | 0.473249532 | 0.444514936 | 0.372256318 | 0.430006929 | 0.536621717 | 0.446101475 | 0.491361596 |
| 690 | L-Pipecolic acid | 535-75-1;3105-95-1 | C00408 | C6H11NO2 | 129.078979 | Amino acid and derivatives | 130.1 | 84.1 | + | 0.68835 | 0.037522944 | 0.035692494 | 0.040331209 | 0.037848883 | 0.087631726 | 0.055948419 | 0.047562339 | 0.063714161 | 0.056374605 | 0.068405252 | 0.062389929 |
| 691 | L-Quebrachitol | 642-38-6 | C08257 | C7H14O6 | 194.07903999999999 | Miscellaneous | 195.1 | 177.1 | + | 4.591333333 | 2.75073E-05 | 6.95365E-05 | 0.000202074 | 9.9706E-05 | 6.50465E-05 | 7.94788E-05 | 7.49083E-05 | 7.31445E-05 | 7.43008E-05 | 8.19763E-05 | 7.81385E-05 |
| 692 | L-Serine | 56-45-1 | C00065 | C3H7NO3 | 105.04259399999999;105.04259999999999 | Amino acid and derivatives;amino acids | 106 | 60 | + | 0.62 | 0.00388549 | 0.004974301 | 0.004703453 | 0.004521081 | 0.006127258 | 0.002786749 | 0.00319362 | 0.004035876 | 0.003828622 | 0.004317443 | 0.004073033 |
| 693 | L-Theanine | 3081-61-6 | C01047 | C7H14N2O3 | 174.10044300000001 | Amino acid and derivatives | 175.1 | 84 | + | 0.848766667 | 0.000258147 | 0.000168865 | 0.000431904 | 0.000286305 | 0.00022136 | 0.000584046 | 0.000178166 | 0.000327857 | 0.000234902 | 0.000326773 | 0.000280837 |
| 694 | L-Threonine | 72-19-5 | C00188 | C4H9NO3 | 119.058244;119.0582 | Amino acid and derivatives;amino acids | 120.1 | 56.1 | + | 0.64 | 0.019505179 | 0.024691777 | 0.022472573 | 0.022223176 | 0.011348621 | 0.01263711 | 0.007699714 | 0.010561815 | 0.016308201 | 0.013619137 | 0.014963669 |
| 696 | L-Valine | 72-18-4 | C00183 | C5H11NO2 | 117.078979;117.07899999999999 | Amino acid and derivatives;amino acids | 118.1 | 72.1 | + | 0.93 | 0.004725645 | 0.00438661 | 0.019420891 | 0.009511049 | 0.025605889 | 0.006119013 | 0.018150839 | 0.016625247 | 0.005268196 | 0.004954685 | 0.005111441 |
| 698 | Lactaroviolin | 85-33-6 | C09696 | C15H14O | 210.104465 | Prenol lipids | 211.1 | 193.1 | + | 10.50633333 | 6.1486E-05 | 7.29216E-05 | 2.19364E-05 | 5.21147E-05 | 0.000152099 | 7.22217E-05 | 4.68627E-05 | 9.03943E-05 | 0.000114194 | 0.000180694 | 0.000147444 |
| 699 | Lactulose | 4618-18-2 | C07064 | C12H22O11 | 342.11621500000001 | Organooxygen compounds | 365.1 | 203.1 | + | 0.772266667 | 0.005102065 | 0.005008031 | 0.005061938 | 0.005057345 | 0.017284764 | 0.013223925 | 0.011440045 | 0.013982911 | 0.012389093 | 0.008000565 | 0.010194829 |
| 700 | Lactupicrin | 65725-11-3 | C09490 | C23H22O7 | 410.13655499999999 | Sesquiterpenoids | 411.1 | 259.1 | + | 13.79578333 | 6.88038E-05 | 3.10893E-05 | 2.68146E-05 | 4.22359E-05 | 3.98149E-05 | 2.25971E-05 | 3.46615E-05 | 3.23578E-05 | 5.61786E-05 | 4.94175E-05 | 5.2798E-05 |
| 701 | Lancerin | 81991-99-3 | C10075 | C19H18O10 | 406.09 | Xanthones | 407.1 | 389.1 | + | 11.47373333 | 0.00017582 | 0.00011573 | 0.000293289 | 0.000194946 | 0.00012707 | 0.000209457 | 9.69143E-05 | 0.00014448 | 7.57598E-05 | 0.000365832 | 0.000220796 |
| 702 | Lannaconitine | 32854-75-4 | C08694 | C32H44N2O8 | 584.30976799999996 | Alkaloids | 585.3 | 162.1 | + | 6.6628 | 6.08569E-06 | 1.99826E-05 | 2.90029E-05 | 1.8357E-05 | 1.59752E-06 | 5.32094E-05 | 1.25526E-05 | 2.24532E-05 | 4.81468E-05 | 5.26611E-05 | 5.04039E-05 |
| 703 | Lansiumarin A | 205115-73-7 |  | C21H20O5 | 352.13107500000001 | Coumarins | 353.1 | 69 | + | 11.64758333 | 0.010675815 | 0.005759455 | 0.016785095 | 0.011073455 | 0.003065149 | 0.012228576 | 0.003946496 | 0.006413407 | 0.004697586 | 0.005003564 | 0.004850575 |
| 704 | Lariciresinol | 27003-73-2 | C10646 | C20H24O6 | 360.15728999999999 | Lignans | 359.2 | 329.1 | - | 5.88315 | 0.000262621 | 0.000155358 | 0.000143797 | 0.000187259 | 0.000271695 | 1.26522E-06 | 0.000151048 | 0.000141336 | 0.000253361 | 0.000211849 | 0.000232605 |
| 705 | Lathyrol | 34420-19-4 | C09125 | C20H30O4 | 334.21440999999999 | Diterpenoids | 335.2 | 317.2 | + | 7.149766667 | 8.08505E-05 | 0.000122029 | 8.79691E-05 | 9.69496E-05 | 0.000171914 | 0.000116009 | 8.80692E-05 | 0.000125331 | 9.59395E-05 | 9.55232E-05 | 9.57314E-05 |
| 706 | Latifoline | 6029-86-3 | C10344 | C20H27NO7 | 393.17875400000003 | Alkaloids | 394.2 | 55.1 | + | 8.559516667 | 0.00033047 | 0.000282772 | 3.39444E-05 | 0.000215729 | 2.50858E-05 | 1.26522E-06 | 0.000320467 | 0.000115606 | 0.000322586 | 0.000277714 | 0.00030015 |
| 707 | Lecanoric acid | 480-56-8 | C02868 | C16H14O7 | 318.07395500000001 | Phenols | 317.1 | 167 | - | 0.657066667 | 2.3943E-05 | 8.75096E-05 | 2.68151E-05 | 4.60893E-05 | 5.65916E-05 | 0.000121234 | 7.22898E-05 | 8.33718E-05 | 7.45459E-05 | 5.46383E-05 | 6.45921E-05 |
| 708 | Leiocarposide | 71953-77-0 | C10805 | C27H34O16 | 614.18469000000005 | Phenols | 615.2 | 435.1 | + | 12.29308333 | 0.000863313 | 0.001585408 | 0.000690638 | 0.001046453 | 0.00058964 | 0.000797755 | 0.00071052 | 0.000699305 | 0.001044137 | 0.000675727 | 0.000859932 |
| 709 | Leucodopachrome | 18766-67-1 | C05604 | C9H9NO4 | 195.05315899999999 | Indoles and derivatives | 196.1 | 150.1 | + | 4.816933333 | 0.00012707 | 0.000145484 | 0.000110044 | 0.000127533 | 8.04981E-05 | 0.00010702 | 5.98905E-05 | 8.24695E-05 | 5.02859E-05 | 0.000225865 | 0.000138076 |
| 710 | Leukoaminochrome | 29539-03-5 | C17756 | C8H9NO2 | 151.06332900000001 | Indoles and derivatives | 152.1 | 80 | + | 2.416983333 | 0.011343602 | 0.016155635 | 0.011131273 | 0.012876837 | 0.014670033 | 0.013713664 | 0.011489996 | 0.013291231 | 0.012736499 | 0.010584092 | 0.011660296 |
| 711 | Leukotriene A4 | 72059-45-1 | C00909 | C20H30O3 | 318.21949499999999 | Fatty Acyls | 319.2 | 301.2 | + | 5.565183333 | 0.000132632 | 8.98669E-05 | 0.000207082 | 0.000143194 | 0.000264337 | 5.13183E-05 | 0.000211671 | 0.000175775 | 0.000121207 | 0.000109333 | 0.00011527 |
| 712 | Levodopa | 59-92-7 | C00355 | C9H11NO4 | 197.06880899999999 | Amino acid and derivatives | 198.1 | 152.1 | + | 9.867066667 | 1.45914E-05 | 1.32064E-05 | 3.31303E-05 | 2.03093E-05 | 4.93151E-05 | 2.36829E-05 | 5.52343E-05 | 4.27441E-05 | 2.1138E-05 | 2.79642E-05 | 2.45511E-05 |
| 713 | Ligustilide | 4431-01-0 | C16987 | C12H14O2 | 190.09938 | Miscellaneous | 191.1 | 67.1 | + | 2.803 | 0.000220796 | 0.000251711 | 0.000190282 | 0.00022093 | 0.000288421 | 0.000270929 | 0.000277102 | 0.000278817 | 0.000245563 | 0.000226588 | 0.000236076 |
| 714 | Limonexic acid | 99026-99-0 |  | C26H30O10 | 502.18389999999999 | Triterpenoids | 503.2 | 485.2 | + | 12.55385 | 0.003324835 | 0.006663443 | 0.00651936 | 0.005502546 | 0.011343616 | 0.007449236 | 0.009700763 | 0.009497872 | 0.005461334 | 0.00312407 | 0.004292702 |
| 715 | Linamarin | 554-35-8 | C01594 | C10H17NO6 | 247.10558900000001 | Organooxygen compounds | 248.1 | 68 | + | 0.705133333 | 0.000140691 | 0.000103908 | 0.000217516 | 0.000154038 | 0.000101379 | 0.000105684 | 0.000108003 | 0.000105022 | 0.000210611 | 0.000197918 | 0.000204264 |
| 716 | Linarin | 480-36-4 |  | C28H32O14 | 592.17921000000001 | Flavonoids | 593.2 | 285.1 | + | 7.649633333 | 0.000159688 | 5.28943E-05 | 0.000139423 | 0.000117335 | 6.64636E-05 | 2.1001E-05 | 7.96567E-05 | 5.57071E-05 | 7.27426E-05 | 6.73317E-05 | 7.00372E-05 |
| 718 | Linderane | 13476-25-0 | C09495 | C15H16O4 | 260.10485999999997 | Sesquiterpenoids | 261.1 | 243.1 | + | 10.01963333 | 3.93776E-05 | 4.00757E-05 | 0.000112345 | 6.39326E-05 | 4.20277E-05 | 4.11524E-05 | 2.17223E-05 | 3.49675E-05 | 5.0196E-05 | 3.51037E-05 | 4.26499E-05 |
| 719 | Linolenic acid | 463-40-1 | C06427 | C18H30O2 | 278.22458 | Lipids | 277.2 | 59 | - | 12.16906198 | 0.000208375 | 0.000199311 | 0.000220376 | 0.000209354 | 0.001591005 | 0.000336949 | 0.000179795 | 0.000702583 | 0.000550169 | 0.000165798 | 0.000357984 |
| 720 | Lipiferolide | 41059-80-7 | C09497 | C17H22O5 | 306.146725 | Sesquiterpenoids | 307.2 | 247.1 | + | 5.35675 | 0.000465833 | 0.001087364 | 0.000465749 | 0.000672982 | 0.001245956 | 0.000942208 | 0.001269128 | 0.001152431 | 0.00066023 | 0.001043991 | 0.000852111 |
| 721 | Lipoic acid | 1077-28-7;62-46-4 | C00725 | C8H14O2S2 | 206.043522 | Organic acids and derivatives | 205 | 65 | - | 4.9015 | 0.000153405 | 0.000348 | 1.12648E-06 | 0.00016751 | 0.000917751 | 0.000587742 | 1.23455E-06 | 0.000502242 | 0.000347124 | 0.000483918 | 0.000415521 |
| 722 | Liquiritigenin | 578-86-9 | C09762 | C15H12O4 | 256.07355999999999 | Flavonoids | 257.1 | 121 | + | 9.650416667 | 0.000165009 | 4.6317E-05 | 0.000123088 | 0.000111471 | 0.000123965 | 0.000108249 | 7.20875E-05 | 0.000101434 | 8.23719E-05 | 0.000106531 | 9.44513E-05 |
| 723 | Liquiritin | 551-15-5 | C16989 | C21H22O9 | 418.12638500000003 | Flavonoids | 419.1 | 239.1 | + | 5.730733333 | 0.000523772 | 0.000683597 | 0.000225698 | 0.000477689 | 0.000716528 | 0.000454834 | 0.000264635 | 0.000478666 | 0.00040975 | 0.000607205 | 0.000508477 |
| 724 | Liriodendrin | 573-44-4 |  | C34H46O18 | 742.26841999999999 | Lignans | 743.3 | 563.2 | + | 12.13428333 | 0.001963524 | 0.002559789 | 0.006153222 | 0.003558845 | 0.00345729 | 0.001620229 | 0.002094472 | 0.002390663 | 0.003485687 | 0.005325778 | 0.004405732 |
| 725 | Lithocholic acid | 434-13-9 | C03990 | C24H40O3 | 376.29774500000002 | Steroids | 375.3 | 45 | - | 3.137633333 | 4.62854E-05 | 2.38418E-05 | 3.91638E-05 | 3.64303E-05 | 3.26429E-05 | 4.44547E-05 | 2.58716E-05 | 3.43231E-05 | 2.74721E-05 | 3.29555E-05 | 3.02138E-05 |
| 726 | Loganin | 18524-94-2 | C01433 | C17H26O10 | 390.15260000000001 | Terpene | 373.1 | 179.1 | + | 5.09568164 | 0.000375666 | 0.000457936 | 0.000504404 | 0.000446002 | 0.000543712 | 0.000378639 | 0.00029924 | 0.000407197 | 0.000203636 | 0.000235798 | 0.000219717 |
| 727 | Lonicerin | 25694-72-8 | C12630 | C27H30O15 | 594.15847499999995 | Flavonoids | 595.2 | 269 | + | 6.084166667 | 0.000126253 | 0.000180991 | 5.23284E-05 | 0.000119857 | 7.54524E-05 | 7.54172E-05 | 0.000102108 | 8.4326E-05 | 0.000205376 | 0.000116549 | 0.000160962 |
| 728 | Lubiprostone | 136790-76-6;333963-40-9 | C13707 | C20H32F2O5 | 390.22178100000002 | Fatty Acyls | 391.2 | 149 | + | 12.74898333 | 0.0121159 | 0.00369359 | 0.076198815 | 0.030669435 | 0.08103431 | 0.007472864 | 0.104342922 | 0.064283365 | 0.102145841 | 0.080900897 | 0.091523369 |
| 729 | Lucidadiol | 252351-95-4 |  | C30H48O3 | 456.360345 | Triterpenoids | 457.4 | 421.3 | + | 12.64203333 | 0.0001138 | 7.10234E-05 | 0.000202385 | 0.00012907 | 5.73271E-05 | 2.67731E-05 | 0.000167617 | 8.39058E-05 | 6.10247E-05 | 0.000118474 | 8.97493E-05 |
| 730 | Lucidin | 478-08-0 | C10369 | C15H10O5 | 270.05282499999998 | Anthraquinones | 271.1 | 253.1 | + | 8.894683333 | 2.29721E-05 | 7.92542E-05 | 3.35403E-05 | 4.52555E-05 | 0.000121093 | 7.20338E-05 | 0.00017257 | 0.000121899 | 0.000171687 | 0.000138286 | 0.000154987 |
| 731 | Lumichrome | 1086-80-2 | C01727 | C12H10N4O2 | 242.080376 | Alkaloids | 243.1 | 198.1 | + | 6.696616667 | 0.002272723 | 0.002119265 | 0.002082433 | 0.00215814 | 0.001927071 | 0.001208714 | 0.001322665 | 0.00148615 | 0.001699764 | 0.001609722 | 0.001654743 |
| 732 | Lupanine | 550-90-3 | C10772 | C15H24N2O | 248.188863 | Alkaloids | 249.2 | 136.1 | + | 2.958883333 | 0.001572905 | 0.002058918 | 0.00224121 | 0.001957678 | 0.000497054 | 0.00047374 | 0.000517228 | 0.000496007 | 0.002974913 | 0.002880193 | 0.002927553 |
| 733 | Lupeol | 545-47-1 | C08628 | C30H50O | 426.38616500000001 | Triterpenoids | 427.4 | 109.1 | + | 11.90331667 | 0.000615531 | 0.000363851 | 0.000389495 | 0.000456293 | 0.000552217 | 0.00035757 | 0.000614472 | 0.000508087 | 0.000358911 | 0.000214771 | 0.000286841 |
| 734 | Lusianthridin | 87530-30-1 | C10257 | C15H14O3 | 242.09429499999999 | Miscellaneous | 243.1 | 183.1 | + | 9.986066667 | 0.000131584 | 5.06716E-05 | 0.000168171 | 0.000116809 | 6.41698E-05 | 0.000121453 | 0.000102629 | 9.60838E-05 | 8.88189E-05 | 0.000100304 | 9.45614E-05 |
| 735 | Lutein | 127-40-2 | C08601 | C40H56O2 | 568.42803000000004 | Prenol lipids | 569.4 | 533.4 | + | 13.1366 | 8.1663E-05 | 0.000391218 | 0.000107775 | 0.000193552 | 0.000149002 | 0.000169655 | 0.000173661 | 0.000164106 | 0.000278914 | 0.000142905 | 0.00021091 |
| 737 | Luteolin-6-C-glucoside;Orientin | 4261-42-1;28608-75-5 | C01821;C10114 | C21H20O11 | 448.10056500000002 | Flavonoids | 449.1 | 431.1 | + | 5.555133333 | 0.000599779 | 0.000576529 | 0.000282499 | 0.000486269 | 9.22776E-05 | 0.000665754 | 0.001047812 | 0.000601948 | 0.000111976 | 0.000109381 | 0.000110678 |
| 738 | Luvangetin | 483-92-1 | C09273 | C15H14O4 | 258.08920999999998 | Coumarins | 259.1 | 203 | + | 4.480533333 | 0.000116959 | 7.95226E-05 | 5.27673E-05 | 8.30829E-05 | 6.15381E-05 | 6.56042E-05 | 8.15043E-05 | 6.95489E-05 | 0.000197413 | 7.31991E-05 | 0.000135306 |
| 739 | Lycopene | 502-65-8 | C05432 | C40H56 | 536.43820000000005 | Miscellaneous | 536.4 | 69.1 | + | 11.18425 | 8.78602E-05 | 0.000147356 | 0.000238142 | 0.000157786 | 1.59752E-06 | 4.96087E-05 | 0.000159969 | 7.03917E-05 | 0.000107269 | 0.000141369 | 0.000124319 |
| 740 | Lycorine | 476-28-8 | C08532 | C16H17NO4 | 287.11575900000003 | Alkaloids | 288.1 | 147 | + | 7.585416667 | 0.000127313 | 0.000106326 | 0.00010943 | 0.000114356 | 0.00014777 | 7.34867E-05 | 4.32695E-05 | 8.81755E-05 | 0.000132489 | 5.99172E-05 | 9.62032E-05 |
| 741 | Macamide B | 74058-71-2 |  | C23H39NO | 345.30316399999998 | Alkaloids | 346.3 | 91.1 | + | 8.8496 | 0.001038316 | 0.000684095 | 0.000612315 | 0.000778242 | 0.00067459 | 0.00046108 | 0.000345366 | 0.000493679 | 0.000979426 | 0.000557964 | 0.000768695 |
| 742 | Maclurin | 519-34-6 | C09951 | C13H10O6 | 262.04773999999998 | Xanthones | 263.1 | 137 | + | 8.72735 | 0.00028867 | 0.000148447 | 0.000101037 | 0.000179385 | 0.000143555 | 0.000112553 | 0.000147931 | 0.00013468 | 0.000207717 | 0.000457716 | 0.000332717 |
| 743 | Maculosidin | 522-19-0 | C10716 | C14H13NO4 | 259.08445899999998 | Alkaloids | 260.1 | 228.1 | + | 8.82805 | 0.001565156 | 1.52459E-05 | 3.64686E-05 | 0.000538957 | 9.8359E-06 | 2.3031E-05 | 0.001369903 | 0.00046759 | 0.001292425 | 0.001304943 | 0.001298684 |
| 744 | Magnoflorine | 2141-09-5 | C09581 | C20H23NO4 | 341.16270900000001 | Alkaloids | 342.2 | 269.1 | + | 6.545583333 | 3.87068E-05 | 4.84372E-05 | 3.72435E-05 | 4.14625E-05 | 5.17802E-05 | 2.01887E-05 | 3.33075E-05 | 3.50921E-05 | 4.29545E-05 | 0.000182577 | 0.000112766 |
| 746 | Mahanine | 28360-49-8 |  | C23H25NO2 | 347.18852900000002 | Alkaloids | 348.2 | 222.1 | + | 2.1164 | 9.50314E-06 | 3.58129E-05 | 2.33734E-06 | 1.58844E-05 | 6.64823E-06 | 3.10537E-05 | 7.56533E-06 | 1.50891E-05 | 2.10939E-05 | 3.06671E-05 | 2.58805E-05 |
| 747 | Maltol | 118-71-8 | C11918 | C6H6O3 | 126.031695 | Flavonoids | 127 | 43 | + | 0.8987 | 0.001014736 | 0.002304304 | 0.0011538 | 0.001490947 | 0.003202131 | 0.001255181 | 0.001776229 | 0.002077847 | 0.001851079 | 0.002369386 | 0.002110232 |
| 748 | Maltotriose | 1109-28-0 | C01835 | C18H32O16 | 504.16904 | Organooxygen compounds | 527.2 | 365.1 | + | 1.226433333 | 0.001036713 | 0.001113331 | 0.001202888 | 0.001117644 | 0.003989687 | 0.003581774 | 0.003182533 | 0.003584665 | 0.001742612 | 0.002045845 | 0.001894228 |
| 749 | Malvidin | 643-84-5 | C08716 | C17H14O7 | 330.07395500000001 | Flavonoids | 331.1 | 315 | + | 5.413333333 | 1.18251E-06 | 1.58848E-05 | 1.12648E-06 | 6.0646E-06 | 5.95728E-05 | 7.87502E-05 | 7.81654E-06 | 4.87132E-05 | 2.49877E-05 | 0.000111019 | 6.80034E-05 |
| 750 | Malvidin 3-O-glucoside (Oenin);Malvidin 3-glucoside | 18470-06-9 | C12140 | C23H24O12 | 492.12678 | Flavonoids | 491.1 | 329.1 | - | 7.2965 | 0.000206315 | 6.80778E-05 | 0.000262813 | 0.000179069 | 0.000304813 | 0.000260279 | 2.70688E-05 | 0.000197387 | 2.53218E-05 | 9.61645E-05 | 6.07432E-05 |
| 753 | Mangiferin | 4773-96-0 | C10077 | C19H18O11 | 422.08491500000002 | Xanthones | 423.1 | 273 | + | 5.143064467 | 0.000140817 | 6.9388E-05 | 2.25953E-05 | 7.76002E-05 | 2.0878E-05 | 6.00916E-05 | 0.000263481 | 0.000114817 | 9.30048E-05 | 3.2637E-05 | 6.28209E-05 |
| 755 | Mannose 6-phosphate |  | C00275 | C6H13O9P | 260.02972199999999 | Organooxygen compounds | 283 | 185 | + | 13.02778333 | 0.001672952 | 0.002209913 | 0.001676824 | 0.001853229 | 0.002518716 | 0.001937209 | 0.001188289 | 0.001881405 | 0.001405362 | 0.002171082 | 0.001788222 |
| 758 | Melatonin | 8041-44-9;73-31-4 | C01598 | C13H16N2O2 | 232.12117799999999 | Alkaloids | 233.1 | 174.1 | + | 10.42243333 | 6.39978E-05 | 6.99518E-05 | 0.000112308 | 8.20857E-05 | 8.76333E-06 | 3.61163E-05 | 0.00013248 | 5.91198E-05 | 6.24111E-05 | 3.33302E-05 | 4.78707E-05 |
| 759 | Mellein | 480-33-1 |  | C10H10O3 | 178.062995 | Coumarins | 179.1 | 161.1 | + | 6.445383333 | 0.000296383 | 0.000386695 | 0.00015708 | 0.000280052 | 0.000489537 | 0.000382924 | 0.000436858 | 0.00043644 | 0.000253891 | 0.0002085 | 0.000231195 |
| 760 | Meloside A | 60767-80-8 |  | C27H30O15 | 594.15847499999995 | Flavonoids | 595.2 | 415.1 | + | 4.864158333 | 8.79423E-05 | 4.06588E-05 | 8.77679E-05 | 7.2123E-05 | 0.000344933 | 0.000171514 | 5.76405E-05 | 0.000191363 | 0.000144601 | 0.000125919 | 0.00013526 |
| 761 | Mesaconitine | 2752-64-9 | C08698 | C33H45NO11 | 631.29926399999999 | Alkaloids | 632.3 | 105 | + | 12.92308333 | 0.000211586 | 0.000166442 | 0.00021191 | 0.000196646 | 0.000381872 | 0.000252134 | 0.000254277 | 0.000296094 | 0.000293678 | 0.000123343 | 0.00020851 |
| 762 | Metanephrine | 5001-33-2 | C05588 | C10H15NO3 | 197.10519400000001 | Phenols | 198.1 | 180.1 | + | 2.131683333 | 3.60454E-05 | 0.000157179 | 4.80514E-05 | 8.04252E-05 | 0.000132503 | 0.000182206 | 0.000132902 | 0.000149204 | 4.78406E-05 | 6.31542E-05 | 5.54974E-05 |
| 763 | Methotrexate | 59-05-2 | C01937 | C20H22N8O5 | 454.17131699999999 | Carboxylic acids and derivatives | 453.2 | 367.1 | - | 4.10825 | 0.000104051 | 3.28692E-05 | 0.000123531 | 8.68171E-05 | 9.40041E-05 | 9.80962E-05 | 9.93882E-05 | 9.71629E-05 | 8.50118E-05 | 0.00013356 | 0.000109286 |
| 765 | Methyl benzoate | 93-58-3 | C20645 | C8H8O2 | 136.05242999999999 | Phenols | 137.1 | 105 | + | 10.72676667 | 0.00026546 | 0.000208611 | 0.000139848 | 0.00020464 | 7.7966E-05 | 0.000125489 | 8.26918E-05 | 9.53822E-05 | 0.00018708 | 0.000108816 | 0.000147948 |
| 766 | Methyl caffeate acid | 3843-74-1 | C10477 | C10H10O4 | 194.05790999999999 | Phenylpropanoids | 195.1 | 121 | + | 4.59625 | 0.000314584 | 0.000349161 | 0.000270821 | 0.000311522 | 0.000902503 | 0.000325282 | 0.000245347 | 0.000491044 | 0.000285119 | 0.000367542 | 0.000326331 |
| 767 | Methyl cinnamate | 103-26-4 | C06358 | C10H10O2 | 162.06808000000001 | Phenylpropanoids | 163.1 | 103.1 | + | 4.90085 | 0.004534257 | 0.005259467 | 0.002992273 | 0.004261999 | 0.006311259 | 0.005342529 | 0.005902482 | 0.00585209 | 0.00472839 | 0.005188851 | 0.00495862 |
| 768 | Methyl gallate | 99-24-1 |  | C8H8O5 | 184.03717499999999 | Phenols | 185 | 125 | + | 4.4436 | 0.000110463 | 4.58862E-05 | 8.94857E-05 | 8.1945E-05 | 0.000104483 | 6.10043E-05 | 7.86041E-05 | 8.13638E-05 | 0.00012985 | 8.13395E-05 | 0.000105595 |
| 769 | Methyl hesperidin | 11013-97-1 | C17393 | C29H36O15 | 624.20542499999999 | Flavonoids | 625.2 | 317.1 | + | 12.9902 | 0.001084055 | 0.000873192 | 0.001021003 | 0.00099275 | 0.001223808 | 0.00142296 | 0.000626309 | 0.001091025 | 0.001608292 | 0.001185109 | 0.0013967 |
| 770 | Methyl jasmonate | 39924-52-2 | C11512 | C13H20O3 | 224.1412 | phytohormone | 225 | 151 | + | 11.3 | 0.000253668 | 0.000779257 | 0.000148036 | 0.000393654 | 0.00072067 | 0.00020402 | 0.000367488 | 0.000430726 | 0.000291288 | 0.000394709 | 0.000342998 |
| 771 | Methyl linoleate | 112-63-0 |  | C19H34O2 | 294.25587999999999 | Miscellaneous | 295.3 | 55.1 | + | 12.00001667 | 0.00223318 | 0.00137761 | 0.00164467 | 0.00175182 | 0.001534438 | 0.00125306 | 0.001317043 | 0.001368181 | 0.001661886 | 0.001460414 | 0.00156115 |
| 772 | Methyl reserpate | 2901-66-8 |  | C23H30N2O5 | 414.21547299999997 | Alkaloids | 415.2 | 174.1 | + | 7.21325 | 3.62875E-05 | 3.89082E-05 | 0.000209385 | 9.48601E-05 | 0.000207617 | 0.000183673 | 0.00012186 | 0.00017105 | 0.000202478 | 0.000128745 | 0.000165611 |
| 773 | Methyl rosmarinate | 99353-00-1 |  | C19H18O8 | 374.10016999999999 | Phenylpropanoids | 375.1 | 121 | + | 6.595933333 | 0.000120195 | 0.000162209 | 0.000241438 | 0.000174614 | 0.000211863 | 0.000199931 | 0.000361739 | 0.000257844 | 0.00023797 | 0.000244104 | 0.000241037 |
| 774 | Methyl tanshinonate | 18887-19-9 |  | C20H18O5 | 338.11542500000002 | Diterpenoids | 339.1 | 171.1 | + | 11.4294 | 0.000195587 | 0.000263561 | 0.000378282 | 0.000279143 | 0.000399001 | 0.000489799 | 0.00044301 | 0.000443937 | 0.000603775 | 0.000165613 | 0.000384694 |
| 775 | Methylecgonine | 7143-09-1 | C12448 | C10H17NO3 | 199.12084400000001 | Alkaloids | 200.1 | 182.1 | + | 3.13865 | 3.17996E-05 | 5.16431E-05 | 3.61475E-05 | 3.98634E-05 | 4.87483E-05 | 0.000109821 | 4.0548E-05 | 6.63725E-05 | 0.000107556 | 7.14695E-05 | 8.95126E-05 |
| 776 | Methylimidazole acetaldehyde | 19639-03-3 | C05827 | C6H8N2O | 124.06366300000001 | Azoles | 125.1 | 107.1 | + | 0.71115 | 3.22449E-05 | 1.47583E-05 | 9.00116E-05 | 4.56716E-05 | 0.000112093 | 5.09581E-05 | 2.47852E-05 | 6.26119E-05 | 0.000136436 | 4.91146E-05 | 9.27755E-05 |
| 777 | Methylisopelletierine | 18747-42-7 | C06184 | C9H17NO | 155.13101399999999 | Alkaloids | 156.1 | 98.1 | + | 4.11205 | 3.5667E-05 | 4.7356E-05 | 3.14503E-05 | 3.81577E-05 | 4.75546E-05 | 2.57031E-05 | 4.02525E-05 | 3.78367E-05 | 3.62988E-05 | 3.33082E-05 | 3.48035E-05 |
| 778 | Mevastatin | 73573-88-3 | C13963 | C23H34O5 | 390.24062500000002 | Diterpenoids | 391.2 | 185.1 | + | 12.06715 | 0.001062716 | 0.000786879 | 0.001170568 | 0.001006721 | 0.001570165 | 0.00039416 | 0.001100871 | 0.001021732 | 0.001445277 | 0.000548377 | 0.000996827 |
| 779 | Micromelin | 15085-71-9 | C09277 | C15H12O6 | 288.06339000000003 | Coumarins | 289.1 | 203 | + | 5.1253 | 9.42837E-05 | 5.19717E-05 | 4.1157E-05 | 6.24708E-05 | 0.000222806 | 8.62073E-05 | 4.15915E-05 | 0.000116868 | 7.09133E-05 | 0.000120711 | 9.58122E-05 |
| 780 | Miltirone | 27210-57-7 | C13715 | C19H22O2 | 282.16198000000003 | Diterpenoids | 283.2 | 55.1 | + | 12.97693333 | 0.189247299 | 0.181016673 | 0.133059664 | 0.167774545 | 0.188300225 | 0.14390831 | 0.165538924 | 0.16591582 | 0.160670312 | 0.174880233 | 0.167775273 |
| 781 | Mitraphylline | 509-80-8 | C09227 | C21H24N2O4 | 368.173608 | Alkaloids | 369.2 | 55.1 | + | 13.02376667 | 0.000416099 | 0.000353423 | 0.000196932 | 0.000322151 | 0.000512375 | 0.000541547 | 0.000540949 | 0.000531624 | 0.00048754 | 0.000515677 | 0.000501609 |
| 782 | Momordicoside G | 81371-54-2 |  | C37H60O8 | 632.42881999999997 | Triterpenoids | 633.4 | 471.4 | + | 11.47975 | 0.000207443 | 0.000303224 | 0.000279433 | 0.000263366 | 0.000213777 | 0.000507213 | 0.000255083 | 0.000325358 | 0.000503922 | 0.000172118 | 0.00033802 |
| 783 | Moracin C | 69120-06-5 |  | C19H18O4 | 310.12051000000002 | Phenols | 311.1 | 55.1 | + | 13.98666667 | 0.008221224 | 0.020142705 | 0.017605453 | 0.015323127 | 0.019030034 | 0.005268444 | 0.009257428 | 0.011185302 | 0.008610715 | 0.011641794 | 0.010126254 |
| 784 | Moracin O | 123702-97-6 |  | C19H18O5 | 326.11542500000002 | Phenols | 327.1 | 239.1 | + | 9.5204 | 7.33787E-05 | 3.11795E-05 | 6.80253E-05 | 5.75278E-05 | 7.58389E-05 | 4.55457E-05 | 3.25994E-05 | 5.1328E-05 | 4.95562E-05 | 7.3312E-05 | 6.14341E-05 |
| 785 | Morin | 480-16-0 | C10105 | C15H10O7 | 302.04265500000002 | Flavonoids | 303 | 153 | + | 7.711908 | 5.03586E-05 | 8.51379E-05 | 6.84144E-05 | 6.79703E-05 | 0.000138458 | 7.68564E-05 | 0.000156777 | 0.00012403 | 0.00011792 | 9.73616E-05 | 0.000107641 |
| 786 | Morroniside | 25406-64-8 | C17000 | C17H26O11 | 406.147515 | Iridoids | 407.2 | 227.1 | + | 4.48674965 | 0.000188046 | 0.000276718 | 0.000201005 | 0.000221923 | 0.00022742 | 0.00019032 | 0.000213349 | 0.000210363 | 0.000270302 | 0.000212919 | 0.000241611 |
| 787 | Morusin | 62596-29-6 | C10106 | C25H24O6 | 420.15728999999999 | Flavonoids | 421.2 | 403.2 | + | 4.90085 | 0.000527523 | 0.000601269 | 0.000379542 | 0.000502778 | 0.000849292 | 0.000323681 | 0.000553295 | 0.000575423 | 0.000447633 | 0.000358589 | 0.000403111 |
| 788 | Moschamine | 68573-23-9 |  | C20H20N2O4 | 352.14230800000001 | Alkaloids | 353.1 | 160.1 | + | 11.11051667 | 0.000681211 | 0.00064509 | 0.000492214 | 0.000606172 | 0.000908979 | 0.000331474 | 0.000705063 | 0.000648506 | 0.001290823 | 0.000639117 | 0.00096497 |
| 789 | Mulberrofuran A | 68978-04-1 | C08846 | C25H28O4 | 392.19875999999999 | Phenols | 393.2 | 55.1 | + | 13.22516667 | 4.1192E-05 | 7.70358E-05 | 0.000114095 | 7.74408E-05 | 0.000124088 | 7.41578E-05 | 9.6625E-05 | 9.82903E-05 | 0.000106189 | 4.44918E-05 | 7.53404E-05 |
| 790 | Mulberrofuran C | 77996-04-4 | C08928 | C34H28O9 | 580.17333499999995 | Phenols | 581.2 | 137 | + | 12.8056 | 0.033449203 | 0.034912218 | 0.024733956 | 0.031031792 | 0.066414826 | 0.051854722 | 0.041420356 | 0.053229968 | 0.045299291 | 0.035507778 | 0.040403535 |
| 791 | Mulberrofuran Q | 101383-35-1 |  | C34H24O10 | 592.13694999999996 | Phenols | 593.1 | 575.1 | + | 13.27551667 | 0.048394652 | 0.049379767 | 0.043166406 | 0.046980275 | 0.054112748 | 0.05299628 | 0.048552723 | 0.05188725 | 0.056554437 | 0.044867776 | 0.050711107 |
| 792 | Muramic acid | 1114-41-6 | C06470 | C9H17NO7 | 251.100504 | Organooxygen compounds | 252.1 | 72 | + | 2.782966667 | 3.11523E-05 | 5.03785E-05 | 6.20085E-05 | 4.78464E-05 | 3.59029E-05 | 0.000106858 | 8.80313E-05 | 7.69308E-05 | 4.00222E-05 | 9.84854E-05 | 6.92538E-05 |
| 793 | Myricetin | 529-44-2 | C10107 | C15H10O8 | 318.0376 | flavonoids | 318.9 | 272.9 | + | 7.05 | 0.000228701 | 0.000227064 | 9.76308E-05 | 0.000184465 | 0.000122567 | 0.000127244 | 9.88346E-05 | 0.000116215 | 9.71425E-05 | 6.67813E-05 | 8.19619E-05 |
| 794 | Myricitrin;Myricetin 3-O-rhamnoside (Myricitrin) | 17912-87-7 | C10108 | C21H20O12 | 464.09548000000001 | Flavonoids | 465.1 | 319 | + | 6.844666667 | 4.5998E-05 | 5.91187E-05 | 0.000121692 | 7.56028E-05 | 9.60304E-05 | 0.000137526 | 6.56113E-05 | 9.97226E-05 | 4.81317E-05 | 0.000199633 | 0.000123882 |
| 795 | Myristoleic acid | 544-64-9 | C08322 | C14H26O2 | 226.19327999999999 | Lipids | 227.2 | 69.1 | + | 4.959966667 | 0.000325863 | 0.000337614 | 0.000482174 | 0.000381884 | 0.000391726 | 0.000159766 | 0.000257779 | 0.000269757 | 0.000400517 | 0.000255592 | 0.000328055 |
| 796 | N-((-)-jasmonoyl)-S-isoleucine | 120330-92-9 | C18699 | C18H29NO4 | 323.20965799999999 | phytohormone | 322 | 130 | - | 10.41 | 0.003113352 | 0.0034057 | 0.003074892 | 0.003197981 | 0.000403204 | 0.000314397 | 0.000330412 | 0.000349338 | 0.001327349 | 0.001220767 | 0.001274058 |
| 797 | N-(p-Hydroxyphenethyl)actinidine | 15794-92-0 | C09984 | C18H22NO | 268.17013900000001 | Phenols | 269.2 | 121.1 | + | 9.868583333 | 0.000317858 | 0.000188463 | 0.000290342 | 0.000265554 | 0.000389335 | 0.000359019 | 0.00025668 | 0.000335011 | 0.000365939 | 0.000224528 | 0.000295233 |
| 798 | N-Acetyl-D-glucosamine 6-phosphate | 1746-32-3 | C00357 | C8H16NO9P | 301.05627099999998 | Organooxygen compounds | 302.1 | 99 | + | 5.500716667 | 0.000598786 | 0.000784056 | 0.000824842 | 0.000735895 | 0.001779636 | 0.000802925 | 0.000966982 | 0.001183181 | 0.001073965 | 0.000712612 | 0.000893289 |
| 799 | N-Acetyl-L-glutamate 5-semialdehyde | 13074-21-0 | C01250 | C7H11NO4 | 173.06880899999999 | Carboxylic acids and derivatives | 174.1 | 86.1 | + | 1.51005 | 0.000354673 | 0.000741807 | 0.000683893 | 0.000593458 | 0.000599736 | 0.000417956 | 0.000295062 | 0.000437585 | 0.00067427 | 0.000254734 | 0.000464502 |
| 800 | N-Acetyl-L-glutamic acid | 1188-37-0 | C00624 | C7H11NO5 | 189.06372400000001 | Amino acid and derivatives | 190.1 | 84 | + | 3.74675 | 3.07647E-05 | 3.77766E-05 | 4.92833E-05 | 3.92749E-05 | 4.3874E-05 | 2.38283E-05 | 2.27872E-05 | 3.01632E-05 | 2.7452E-05 | 3.8741E-05 | 3.30965E-05 |
| 802 | N-Acetyl-l-leucine | 1188-21-2 | C02710 | C8H15NO3 | 173.10519400000001 | Amino acid and derivatives | 174.1 | 86.1 | + | 5.376733333 | 0.0002214 | 0.000389073 | 0.000364171 | 0.000324881 | 0.000478851 | 0.000193381 | 0.000283615 | 0.000318616 | 0.000638985 | 0.000227567 | 0.000433276 |
| 804 | N-Feruloyl putrescine | 501-13-3 | C10497 | C14H20N2O3 | 264.14739300000002 | Phenolamides | 265.2 | 72.1 | + | 4.331733333 | 0.009019486 | 0.009840955 | 0.007852323 | 0.008904255 | 0.007061051 | 0.005769918 | 0.00746986 | 0.006766943 | 0.008664889 | 0.006995881 | 0.007830385 |
| 805 | N-formylmethionine | 4289-98-9 | C03145 | C6H11NO3S | 177.045965 | Amino acid and derivatives | 178.1 | 61 | + | 0.673566667 | 0.000225345 | 0.000280818 | 0.000179132 | 0.000228432 | 0.00024886 | 0.000395624 | 0.000370159 | 0.000338214 | 0.000303294 | 0.000163469 | 0.000233381 |
| 806 | N-hydroxy tryptamine |  | C17203 | C10H12N2O | 176.09496300000001 | Tryptamine derivatives | 177.1 | 144.1 | + | 2.618383333 | 0.002233797 | 0.001746578 | 0.001133957 | 0.001704777 | 0.006148284 | 0.004850187 | 0.004958676 | 0.005319049 | 0.002087624 | 0.002768285 | 0.002427955 |
| 807 | N,N'-diacetylchitobiose | 35061-50-8 | C01674 | C16H28N2O11 | 424.16931299999999 | Organooxygen compounds | 425.2 | 204.1 | + | 0.7655 | 0.000103974 | 7.59515E-05 | 0.000117473 | 9.91329E-05 | 5.22991E-05 | 7.57058E-05 | 2.01975E-05 | 4.94008E-05 | 9.13908E-05 | 0.000174889 | 0.00013314 |
| 808 | N'-Formylkynurenine | 1022-31-7 | C02700 | C11H12N2O4 | 236.07970800000001 | Amino acid and derivatives | 237.1 | 163.1 | + | 9.5123 | 6.84732E-05 | 0.000144812 | 0.000135769 | 0.000116351 | 0.000189004 | 5.88643E-05 | 0.000175435 | 0.000141101 | 5.43156E-05 | 0.000116494 | 8.54045E-05 |
| 809 | N1-(alpha-D-ribosyl)-5,6-dimethyl-benzimidazole | 132-13-8 | C05775 | C14H18N2O4 | 278.12665800000002 | Benzimidazole ribonucleosides and ribonucleotides | 279.1 | 147.1 | + | 11.88253333 | 0.000416032 | 0.000732936 | 0.000433492 | 0.000527487 | 0.000269624 | 0.000430315 | 0.000307013 | 0.000335651 | 0.000800511 | 0.000302826 | 0.000551669 |
| 810 | N1-Methyl-2-pyridone-5-carboxamide | 701-44-0 | C05842 | C7H8N2O2 | 152.05857800000001 | Pyridines and derivatives | 153.1 | 108 | + | 2.627833333 | 0.000740718 | 0.000636979 | 0.000465969 | 0.000614555 | 0.001092695 | 0.00058191 | 0.000880121 | 0.000851575 | 0.000991309 | 0.00065921 | 0.000825259 |
| 811 | N1-Methyl-4-pyridone-3-carboxamide | 769-49-3 | C05843 | C7H8N2O2 | 152.05857800000001 | Pyridines and derivatives | 153.1 | 136 | + | 2.655733333 | 0.003780539 | 0.003950752 | 0.004062966 | 0.003931419 | 0.005372698 | 0.004263625 | 0.004024834 | 0.004553719 | 0.004522707 | 0.004093037 | 0.004307872 |
| 812 | N2-gamma-Glutamylglutamine | 1466-50-8 | C05283 | C10H17N3O6 | 275.11173700000001 | Carboxylic acids and derivatives | 276.1 | 213.1 | + | 2.416983333 | 3.65594E-06 | 5.28942E-05 | 7.19825E-06 | 2.12495E-05 | 3.34586E-05 | 4.03761E-06 | 3.86093E-05 | 2.53685E-05 | 2.82529E-05 | 6.37148E-05 | 4.59839E-05 |
| 813 | N6-(delta 2-Isopentenyl)-adenine | 2365-40-4 | C04083 | C10H13N5 | 203.11709999999999 | phytohormone | 204 | 136 | + | 5.3 | 6.8102E-05 | 0.0001287 | 4.35914E-05 | 8.01313E-05 | 0.000454552 | 0.000130721 | 9.05687E-05 | 0.00022528 | 9.58689E-05 | 8.01158E-05 | 8.79923E-05 |
| 814 | N6-isopentenyladenosine | 7724-76-7 |  | C15H21N5O4 | 335.15935400000001 | phytohormone | 336 | 204 | + | 6.1 | 0.005923421 | 0.005914904 | 0.007667064 | 0.006501796 | 0.009925693 | 0.008339598 | 0.00735389 | 0.008539727 | 0.007186458 | 0.007010053 | 0.007098256 |
| 815 | Nandrolone | 434-22-0 | C07254 | C18H26O2 | 274.19328000000002 | Steroids and steroid derivatives | 275.2 | 257.2 | + | 10.58411667 | 0.000298722 | 0.000733977 | 0.000267559 | 0.000433419 | 0.000361153 | 0.001381784 | 0.00032012 | 0.000687686 | 0.000517441 | 0.000255246 | 0.000386343 |
| 816 | Napellonine | 509-24-0 | C08707 | C22H31NO3 | 357.23039399999999 | Alkaloids | 358.2 | 340.2 | + | 8.475616667 | 8.05742E-05 | 0.000102117 | 0.000115592 | 9.9428E-05 | 9.3335E-05 | 0.000100196 | 8.74128E-05 | 9.36479E-05 | 0.000109364 | 0.000149098 | 0.000129231 |
| 817 | Narciclasine | 29477-83-6 | C08533 | C14H13NO7 | 307.06920400000001 | Alkaloids | 308.1 | 216 | + | 4.116816667 | 0.000101346 | 0.000151974 | 7.04104E-05 | 0.00010791 | 5.33606E-05 | 6.5304E-05 | 8.33976E-05 | 6.73541E-05 | 6.21075E-05 | 6.42682E-05 | 6.31879E-05 |
| 818 | Naringenin | 480-41-1 | C00509 | C15H12O5 | 272.06849999999997 | flavonoids | 273.3 | 152.8 | + | 8.86 | 0.000252202 | 0.000318444 | 0.000332839 | 0.000301161 | 0.000563778 | 0.000200546 | 0.000150331 | 0.000304885 | 0.000263886 | 0.000212 | 0.000237943 |
| 819 | Naringenin chalcone | 25515-46-2 | C06561 | C15H12O5 | 272.06847499999998 | Flavonoids | 273.1 | 153 | + | 6.54065 | 0.009773284 | 0.011121902 | 0.005313193 | 0.008736126 | 0.003949668 | 0.001583327 | 0.001516959 | 0.002349985 | 0.00465206 | 0.002302957 | 0.003477509 |
| 823 | Nemorensine | 50906-96-2 | C10353 | C18H27NO5 | 337.18892399999999 | Alkaloids | 338.2 | 320.2 | + | 8.324566667 | 7.8714E-05 | 0.000141575 | 0.000163803 | 0.000128031 | 0.000112193 | 0.000216725 | 0.000122864 | 0.000150594 | 7.96025E-05 | 0.000161886 | 0.000120744 |
| 824 | Neocnidilide | 4567-33-3 | C17002 | C12H18O2 | 194.13068000000001 | Lactones | 195.1 | 79.1 | + | 6.8141 | 0.000380571 | 0.000355513 | 0.000333986 | 0.00035669 | 0.000785887 | 0.00029667 | 0.000321856 | 0.000468138 | 0.000353137 | 0.000305319 | 0.000329228 |
| 825 | Neogrifolin;Grifolin;Ugaxanthone | 23665-96-5;6903-07-7;13179-11-8 | | C22H32O2;C18H16O6 | 328.24023;328.09469000000001 | Phenols;Xanthones | 329.1666667 | 55.1 | + | 13.79578333 | 4.26925E-05 | 5.45474E-05 | 8.35383E-05 | 6.02594E-05 | 0.000186792 | 9.97226E-05 | 8.424E-05 | 0.000123585 | 9.12766E-05 | 6.24478E-05 | 7.68622E-05 |
| 826 | Neohesperidin | 13241-33-3 | C09806 | C28H34O15 | 610.18979999999999 | flavonoids | 609 | 300.8 | - | 6.83 | 0.00554389 | 0.004677513 | 0.005395751 | 0.005205718 | 0.000198198 | 2.76081E-05 | 0.000133779 | 0.000119861 | 0.002554677 | 0.002189 | 0.002371838 |
| 827 | Neolitsine | 2466-42-4 |  | C19H17NO4 | 323.11575900000003 | Alkaloids | 324.1 | 250.1 | + | 5.035116667 | 8.88789E-05 | 0.000172444 | 8.25185E-05 | 0.000114614 | 0.000140117 | 2.56423E-05 | 0.000116441 | 9.40667E-05 | 5.19325E-05 | 0.000131031 | 9.1482E-05 |
| 828 | Neosperidin dihydrochalcone | 20702-77-6 |  | C28H36O15 | 612.20542499999999 | Chalcones | 613.2 | 305.1 | + | 12.45538333 | 0.002508366 | 0.018658504 | 0.013871703 | 0.011679524 | 0.019839198 | 0.016909089 | 0.008652907 | 0.015133731 | 0.017093293 | 0.009119451 | 0.013106372 |
| 829 | Nerylacetate | 141-12-8 |  | C12H20O2 | 196.14633000000001 | Monoterpenoids | 219.1 | 81.1 | + | 8.374916667 | 0.00040384 | 0.000389052 | 0.000799718 | 0.00053087 | 0.000318742 | 0.000397488 | 0.000237172 | 0.000317801 | 0.000816809 | 0.000580061 | 0.000698435 |
| 830 | NG,NG-Dimethylarginine dihydrochloride | 220805-22-1;30315-93-6 | C03626 | C8H18N4O2 | 202.142976 | Carboxylic acids and derivatives | 203.2 | 70.1 | + | 0.7442 | 0.001228619 | 0.001681352 | 0.001442427 | 0.001450799 | 0.002978126 | 0.002329553 | 0.002841001 | 0.002716227 | 0.00215208 | 0.001458923 | 0.001805501 |
| 831 | Nicotinamide | 98-92-0 | C00153 | C6H6N2O | 122.048013 | Alkaloids | 123.1 | 80.1 | + | 1.01434493 | 0.011133071 | 0.018010219 | 0.011225392 | 0.013456227 | 0.014788701 | 0.011465123 | 0.007323043 | 0.011192289 | 0.011230882 | 0.011711281 | 0.011471081 |
| 832 | Nicotinate ribonucleoside | | C05841 | C11H13NO6 | 255.07428899999999 | Nicotinic acid derivatives | 256.1 | 124 | + | 4.546666667 | 9.97783E-05 | 8.74216E-05 | 0.000155792 | 0.000114331 | 6.86423E-05 | 0.000107138 | 0.000119054 | 9.82781E-05 | 0.000155045 | 8.72816E-05 | 0.000121163 |
| 833 | Nicotinic acid | 59-67-6 | C00253 | C6H5NO2 | 123.03202899999999 | Nicotinic acid derivatives | 124 | 78 | + | 0.73194025 | 0.003442694 | 0.003385447 | 0.003684687 | 0.003504276 | 0.007350231 | 0.005339525 | 0.004887808 | 0.005859188 | 0.003256064 | 0.003677344 | 0.003466704 |
| 834 | Nicotinic acid adenine dinucleotide;NAD | 53-84-9 | C00003 | C21H27N7O14P2 | 663.10912699999994 | Nucleotide and its derivates | 664.1 | 136.1 | + | 0.753433333 | 0.000425061 | 0.000552813 | 0.000591341 | 0.000523072 | 0.000723216 | 0.000632938 | 0.000619344 | 0.000658499 | 0.000562813 | 0.000714414 | 0.000638613 |
| 835 | Nicotinic acid mononucleotide | 321-02-8 | C01185 | C11H14NO9P | 335.04062099999999 | Organooxygen compounds | 358 | 121 | + | 5.656083333 | 5.0148E-05 | 7.07364E-05 | 8.3072E-05 | 6.79855E-05 | 0.000121086 | 5.70115E-05 | 8.28355E-05 | 8.69775E-05 | 0.000162126 | 4.02449E-05 | 0.000101185 |
| 836 | Nicotinurate | 583-08-4 | C05380 | C8H8N2O3 | 180.053493 | Carboxylic acids and derivatives | 181.1 | 135.1 | + | 3.274033333 | 0.000405556 | 0.000410118 | 0.000328832 | 0.000381502 | 0.000445567 | 0.000328656 | 0.000388079 | 0.000387434 | 0.000130326 | 0.000360771 | 0.000245549 |
| 837 | Niloticin | 115404-57-4 |  | C30H48O3 | 456.360345 | Triterpenoids | 457.4 | 379.2 | + | 9.372466667 | 5.82737E-06 | 9.65963E-06 | 3.61263E-05 | 1.72044E-05 | 3.95358E-05 | 3.75452E-05 | 2.03868E-05 | 3.24892E-05 | 3.58357E-05 | 8.95774E-06 | 2.23967E-05 |
| 838 | Nobiletin | 478-01-3 | C10112 | C21H22O8 | 402.13146999999998 | Flavonoids | 403.1 | 373.1 | + | 11.03 | 0.031672232 | 0.02886204 | 0.039410163 | 0.033314812 | 0.001295352 | 0.000840962 | 1.23455E-06 | 0.000712516 | 0.014735982 | 0.013653674 | 0.014194828 |
| 839 | Norathyriol | 3542-72-1 | C10086 | C13H8O6 | 260.03208999999998 | Xanthones | 261 | 243 | + | 10.00285 | 9.96518E-05 | 4.00757E-05 | 0.000112345 | 8.4024E-05 | 9.33681E-05 | 4.11524E-05 | 2.17223E-05 | 5.2081E-05 | 7.84002E-05 | 3.51037E-05 | 5.67519E-05 |
| 840 | Norbixin | 542-40-5 | C08608 | C24H28O4 | 380.19875999999999 | Prenol lipids | 381.2 | 345.2 | + | 9.83605 | 4.94571E-05 | 3.07964E-05 | 9.40926E-05 | 5.81154E-05 | 7.97259E-05 | 5.03761E-05 | 6.0643E-05 | 6.35817E-05 | 0.000116403 | 3.86405E-05 | 7.75215E-05 |
| 841 | Norchelerythrine | 6900-99-8 | C12226 | C20H15NO4 | 333.10010899999997 | Alkaloids | 334.1 | 304.1 | + | 7.468633333 | 1.61913E-05 | 1.06001E-05 | 4.14615E-05 | 2.2751E-05 | 3.70035E-05 | 1.74095E-05 | 1.35689E-05 | 2.26606E-05 | 2.97032E-05 | 5.14147E-05 | 4.0559E-05 |
| 842 | Nordihydrocapsaicin | 28789-35-7 | C20216 | C17H27NO3 | 293.199094 | Alkaloids | 294.2 | 137.1 | + | 11.86575 | 0.000321658 | 0.000391635 | 0.000257906 | 0.000323733 | 1.59752E-06 | 0.000146601 | 0.000294569 | 0.000147589 | 0.000188559 | 0.000474162 | 0.000331361 |
| 843 | Norepinephrine | 51-41-2 | C00547 | C8H11NO3 | 169.073894 | Alkaloids | 170.1 | 152.1 | + | 1.846366667 | 9.02854E-05 | 6.92998E-05 | 6.15428E-05 | 7.37093E-05 | 6.86015E-05 | 7.361E-05 | 4.85473E-05 | 6.35863E-05 | 5.29199E-05 | 7.79021E-05 | 6.5411E-05 |
| 844 | Norwogonin | 4443-09-8 | C10113 | C15H10O5 | 270.05282499999998 | Flavonoids | 271.1 | 145 | + | 11.83218333 | 0.001354334 | 0.000611796 | 0.000606428 | 0.000857519 | 0.000912332 | 0.00037322 | 0.000905334 | 0.000730295 | 0.000761455 | 0.001047665 | 0.00090456 |
| 845 | Notopterol | 88206-46-6 | C17499 | C21H22O5 | 354.146725 | Coumarins | 355.2 | 55.1 | + | 11.94387 | 0.000204536 | 0.000342893 | 0.000120393 | 0.000222608 | 0.000713828 | 0.000357319 | 0.000268114 | 0.000446421 | 0.00041071 | 0.000196684 | 0.000303697 |
| 846 | O-Acetylethanolamine | 1854-30-4 |  | C4H9NO2 | 103.063329 | Alkaloids | 104.1 | 44 | + | 0.671566667 | 0.175321122 | 0.170579685 | 0.217833979 | 0.187911595 | 0.249590543 | 0.258891345 | 0.153854442 | 0.220778777 | 0.231273307 | 0.165519375 | 0.198396341 |
| 847 | O-Succinyl-L-homoserine | 1492-23-5 | C01118 | C8H13NO6 | 219.07428899999999 |  | 220.1 | 102.1 | + | 2.280366667 | 8.11516E-05 | 3.76639E-05 | 3.40784E-05 | 5.09647E-05 | 0.000122657 | 8.51166E-05 | 2.66997E-05 | 7.81579E-05 | 0.000146297 | 0.000108992 | 0.000127644 |
| 848 | Octadecanamide | 124-26-5 | C13846 | C18H37NO | 283.28751399999999 | Fatty Acyls | 284.3 | 43.1 | + | 13.02093333 | 0.007949192 | 0.006279647 | 0.004745678 | 0.006324839 | 0.006114394 | 0.005919546 | 0.005651398 | 0.005895113 | 0.004907514 | 0.006650319 | 0.005778917 |
| 849 | Octadecyl p-coumarate | 72943-88-5 |  | C27H44O3 | 416.32904500000001 | Phenylpropanoids | 417.3 | 105 | + | 12.06715 | 0.000818692 | 0.000530325 | 0.00054919 | 0.000632736 | 0.00090765 | 0.00037938 | 0.000988921 | 0.00075865 | 0.001603681 | 0.000853431 | 0.001228556 |
| 850 | Octyl Gallate | 1034-01-1 |  | C15H22O5 | 282.146725 | Phenols | 283.2 | 153 | + | 12.97341667 | 0.002856875 | 0.004032883 | 0.002268592 | 0.003052784 | 0.00241172 | 0.002433608 | 0.002635294 | 0.002493541 | 0.002631151 | 0.003555066 | 0.003093109 |
| 851 | Officinalisinin I | 57944-18-0 | C08904 | C45H76O19 | 920.49808499999995 | Steroids and steroid derivatives | 921.5 | 741.4 | + | 14.01396667 | 1.18251E-06 | 7.66154E-05 | 5.7798E-05 | 4.51987E-05 | 7.31051E-05 | 5.68118E-05 | 8.24903E-05 | 7.08024E-05 | 0.000106612 | 6.88514E-05 | 8.77317E-05 |
| 852 | Okanin | 484-76-4 | C08724 | C15H12O6 | 288.06339000000003 | Chalcones | 289.1 | 135 | + | 5.99175 | 8.61828E-05 | 0.000199648 | 0.000266347 | 0.000184059 | 0.000313219 | 0.000327117 | 0.000140663 | 0.000260333 | 0.000365953 | 0.000234342 | 0.000300148 |
| 853 | Oleic acid;Vaccenic acid;Petroselinic acid | 112-80-1;693-72-1;593-39-5 | C00712;C08367;C08363 | C18H34O2 | 282.25587999999999 | Fatty Acyls | 283.3 | 57.1 | + | 13.0279 | 0.141745067 | 0.123268665 | 0.096796912 | 0.120603548 | 0.157739244 | 0.129992655 | 0.094739462 | 0.127490454 | 0.150802566 | 0.10596741 | 0.128384988 |
| 854 | Oleocanthal | 289030-99-5 |  | C17H20O5 | 304.13107500000001 | Phenols | 305.1 | 121.1 | + | 12.4028 | 0.000771061 | 0.001113663 | 0.000801485 | 0.000895403 | 0.000941782 | 0.001095929 | 0.001361342 | 0.001133018 | 0.000759339 | 0.000601871 | 0.000680605 |
| 855 | Oleuropein | 32619-42-4 | C09794 | C25H32O13 | 540.18429500000002 | Iridoids | 541.2 | 137.1 | + | 7.40245675 | 6.39092E-05 | 0.000208063 | 0.000135011 | 0.000135661 | 0.000171252 | 0.000103755 | 9.05451E-05 | 0.000121851 | 0.00013375 | 0.00011003 | 0.00012189 |
| 856 | Oleuroside | 116383-31-4 |  | C25H32O13 | 540.18429500000002 | Iridoids | 541.2 | 137.1 | + | 12.730075 | 0.0004335 | 0.000934069 | 0.000549239 | 0.000638936 | 0.001035922 | 0.001809736 | 0.00178211 | 0.001542589 | 0.001202229 | 0.001869791 | 0.00153601 |
| 857 | Ononin;Formononetin 7-O-glucoside (Ononin) | 486-62-4 | C10509 | C22H22O9 | 430.12638500000003 | Flavonoids | 431.1 | 269.1 | + | 7.3 | 7.57524E-05 | 0.000269122 | 4.33067E-05 | 0.000129394 | 0.000146585 | 9.53472E-05 | 3.34157E-05 | 9.17825E-05 | 0.000146369 | 7.37172E-05 | 0.000110043 |
| 858 | Orobol (5,7,3',4'-tetrahydroxyisoflavone) | 480-23-9 | C10510 | C15H10O6 | 286.04773999999998 | Flavonoids | 287.1 | 269 | + | 7.904983333 | 0.000381774 | 0.000450671 | 0.000169498 | 0.000333981 | 0.000859066 | 0.000571192 | 0.000653447 | 0.000694568 | 0.000423736 | 0.000836899 | 0.000630318 |
| 860 | Oxoglaucine | 5574-24-3 |  | C20H17NO5 | 351.11067400000002 | Alkaloids | 352.1 | 188.1 | + | 13.82935 | 6.01818E-05 | 0.000149703 | 7.73836E-05 | 9.57561E-05 | 0.000107256 | 9.68209E-05 | 6.35229E-05 | 8.91999E-05 | 3.10314E-05 | 7.117E-05 | 5.11007E-05 |
| 862 | Oxymatrine | 16837-52-8 | C10749 | C15H24N2O2 | 264.18377800000002 | Alkaloids | 265.2 | 166.1 | + | 8.374916667 | 3.51683E-05 | 5.24984E-05 | 1.72829E-05 | 3.49832E-05 | 4.67288E-05 | 7.40269E-05 | 3.73641E-05 | 5.27066E-05 | 3.08225E-05 | 5.2797E-05 | 4.18098E-05 |
| 863 | Oxymorphone | 76-41-5 | C08019 | C17H19NO4 | 301.13140900000002 | Phenanthrenes and derivatives | 302.1 | 284.1 | + | 11.18418333 | 0.006354545 | 0.00851559 | 0.008519087 | 0.007796407 | 0.01302116 | 0.00904012 | 0.007135899 | 0.009732393 | 0.011568708 | 0.010413927 | 0.010991317 |
| 864 | Oxypeucedanin | 737-52-0;26091-73-6 | C09282 | C16H14O5 | 286.08412499999997 | Coumarins | 287.1 | 203 | + | 8.123166667 | 9.89372E-05 | 0.000120152 | 7.34233E-05 | 9.75042E-05 | 0.000123151 | 8.09121E-05 | 0.000137115 | 0.000113726 | 5.71395E-05 | 0.000113983 | 8.55612E-05 |
| 865 | p-Cymene | 99-87-6 | C06575 | C10H14 | 134.10955000000001 | Monoterpenoids | 135.1 | 93.1 | + | 11.62068333 | 0.000870754 | 0.000214229 | 0.001411792 | 0.000832258 | 0.001024262 | 0.00076698 | 0.001373901 | 0.001055048 | 0.001181637 | 0.001165683 | 0.00117366 |
| 866 | p-Hydroxymandelic acid | 1198-84-1 | C11527 | C8H8O4 | 168.04226 | Phenols | 169 | 123 | + | 3.356833333 | 0.000836481 | 0.001127182 | 0.000706948 | 0.000890204 | 0.000239804 | 0.000945858 | 0.000877304 | 0.000687656 | 0.000885205 | 0.000991212 | 0.000938208 |
| 867 | p-Hydroxyphenylacetylglycine | | C05596 | C10H11NO4 | 209.06880899999999 | Carboxylic acids and derivatives | 210.1 | 107 | + | 8.173516667 | 9.75144E-05 | 0.000120319 | 0.000104765 | 0.000107533 | 0.00026849 | 0.000148037 | 0.000141905 | 0.000186144 | 0.00023559 | 0.00015167 | 0.00019363 |
| 868 | p-Octopamine | 104-14-3 | C04227 | C8H11NO2 | 153.078979 | Phenols | 154.1 | 91.1 | + | 3.835766667 | 0.15826413 | 0.182135177 | 0.139603781 | 0.160001029 | 0.205854805 | 0.155727184 | 0.156907952 | 0.17282998 | 0.178326324 | 0.176732889 | 0.177529606 |
| 869 | p-Toluenesulfonic acid | 3233-58-7;104-15-4 | C06677 | C7H8O4S | 188.014331 | Organic acids | 189 | 91.1 | + | 11.24478333 | 0.000496795 | 0.000277784 | 0.000640772 | 0.000471784 | 0.000575312 | 0.000501117 | 0.000438365 | 0.000504932 | 0.00051164 | 0.000673145 | 0.000592392 |
| 870 | Paeonolide | 72520-92-4 | C10715 | C20H28O12 | 460.15807999999998 | Phenols | 461.2 | 167.1 | + | 12.06715 | 0.001085543 | 0.000695996 | 0.000847922 | 0.000876487 | 0.001406483 | 0.001162142 | 0.000994205 | 0.00118761 | 0.001920918 | 0.000681352 | 0.001301135 |
| 872 | Palmitic acid | 57-10-3 | C00249 | C16H32O2 | 256.24023 | Lipids | 257.2 | 57.1 | + | 12.91665 | 0.018958706 | 0.021107143 | 0.010405564 | 0.016823804 | 0.020886159 | 0.013948389 | 0.01048324 | 0.015105929 | 0.017860911 | 0.02213682 | 0.019998865 |
| 873 | Palmitoylethanolamide | 544-31-0 | C16512 | C18H37NO2 | 299.28242899999998 | Carboximidic acids and derivatives | 300.3 | 62.1 | + | 12.61295 | 0.000501904 | 1.27649E-06 | 9.65979E-05 | 0.000199926 | 0.000186583 | 0.000145804 | 0.000203199 | 0.000178528 | 0.000124397 | 0.000118724 | 0.00012156 |
| 874 | Panaxynol | 81203-57-8 | C17447 | C17H24O | 244.182715 | Miscellaneous | 245.2 | 57 | + | 12.56613333 | 0.000888861 | 0.000804144 | 0.000460869 | 0.000717958 | 0.00062743 | 0.000526649 | 0.000436755 | 0.000530278 | 0.000683273 | 0.000358926 | 0.000521099 |
| 875 | Pectolinarin | 28978-02-1 |  | C29H34O15 | 622.18977500000005 | Flavonoids | 623.2 | 315.1 | + | 8.232166667 | 0.000102722 | 5.5901E-05 | 3.40597E-05 | 6.42274E-05 | 9.45807E-05 | 9.59216E-05 | 0.000128187 | 0.00010623 | 0.000104871 | 6.94954E-05 | 8.7183E-05 |
| 876 | Pedalitin | 22384-63-0 | C10119 | C16H12O7 | 316.05830500000002 | Flavonoids | 317.1 | 109 | + | 12.08393333 | 0.001223082 | 0.0003151 | 0.000306566 | 0.000614916 | 0.000740399 | 0.000398336 | 0.000302182 | 0.000480306 | 0.000629247 | 0.00054697 | 0.000588108 |
| 877 | Pelargonidin | 7690-51-9;134-04-3 | C05904 | C15H10O5 | 270.05282499999998 | Flavonoids | 271.1 | 121 | + | 8.983633333 | 7.20354E-05 | 0.00011617 | 9.21996E-05 | 9.34683E-05 | 0.000265352 | 0.00021245 | 4.91369E-05 | 0.000175646 | 0.000107609 | 9.99557E-05 | 0.000103782 |
| 878 | Pelargonidin-3-O-glucoside | 18466-51-8 | C12137 | C21H20O10 | 432.10565000000003 | Flavonoids | 433.1 | 271.1 | + | 5.935383333 | 0.000163062 | 0.000177804 | 0.000127205 | 0.000156024 | 0.00016958 | 0.000311966 | 0.000163577 | 0.000215041 | 0.000139884 | 0.000101203 | 0.000120544 |
| 879 | Pelargonidin-3,5-O-diglucoside chloride | 17334-58-6 | C08725 | C27H30O15 | 594.15847499999995 | Flavonoids | 595.2 | 271.1 | + | 5.670866667 | 7.88898E-05 | 2.7551E-05 | 3.72432E-05 | 4.78946E-05 | 5.76241E-05 | 1.26522E-06 | 7.98814E-05 | 4.62569E-05 | 7.05911E-05 | 2.92844E-05 | 4.99378E-05 |
| 880 | Pentadecanoic acid | 1002-84-2 | C16537 | C15H30O2 | 242.22458 | Fatty Acyls | 243.2 | 43.1 | + | 10.02048333 | 6.30135E-05 | 6.13686E-05 | 0.000130776 | 8.50528E-05 | 6.51978E-05 | 5.39579E-05 | 4.12638E-05 | 5.34732E-05 | 0.00010008 | 4.2783E-05 | 7.14315E-05 |
| 881 | Peonidin-3-glucoside | 68795-37-9 | C12141 | C22H22O11 | 462.116215 | Flavonoids | 463.1 | 301.1 | + | 6.927333333 | 0.000535051 | 0.000432245 | 0.000473717 | 0.000480337 | 0.000514311 | 0.000372079 | 0.000278629 | 0.00038834 | 0.000355869 | 0.00045985 | 0.000407859 |
| 882 | Perakine | 4382-56-3 | C19932 | C21H22N2O3 | 350.16304300000002 | Alkaloids | 351.2 | 291.1 | + | 7.19185 | 0.000302976 | 0.000212547 | 0.000585824 | 0.000367116 | 0.000243639 | 0.000222241 | 0.000247686 | 0.000237855 | 0.000371018 | 0.00037454 | 0.000372779 |
| 883 | Petasitenine | 60102-37-6 | C10359 | C19H27NO7 | 381.17875400000003 | Alkaloids | 382.2 | 364.2 | + | 4.682683333 | 0.000124462 | 8.95519E-05 | 6.1072E-05 | 9.16953E-05 | 5.16127E-05 | 6.60985E-05 | 4.76182E-05 | 5.51098E-05 | 0.000122921 | 4.35198E-05 | 8.32206E-05 |
| 884 | Phendimetrazine | 634-03-7 | C07904 | C12H17NO | 191.13101399999999 | Oxazinanes | 192.1 | 91.1 | + | 10.56366667 | 0.000438895 | 0.000387916 | 0.000564666 | 0.000463826 | 0.000524267 | 0.000641289 | 0.000454105 | 0.000539887 | 0.000382904 | 0.000471385 | 0.000427144 |
| 885 | Phenethylamine | 64-04-0 | C05332 | C8H11N | 121.08914900000001 | Benzene and substituted derivatives | 122.1 | 105.1 | + | 2.819783333 | 0.001424243 | 0.001497323 | 0.001431007 | 0.001450858 | 0.001444389 | 0.001536342 | 0.00098285 | 0.001321194 | 0.001389529 | 0.001519959 | 0.001454744 |
| 886 | Phenylacetonitrile | 140-29-4 | C16074 | C8H7N | 117.057849 | Benzene and substituted derivatives | 118.1 | 101 | + | 3.759616667 | 1.02035E-05 | 9.21362E-05 | 2.35757E-05 | 4.19718E-05 | 4.68359E-05 | 2.2568E-05 | 3.38921E-05 | 3.4432E-05 | 5.64159E-06 | 2.05856E-05 | 1.31136E-05 |
| 887 | Phenylacetyl-L-glutamine | 28047-15-6 | C04148 | C13H16N2O4 | 264.11100800000003 | Amino acid and derivatives | 265.1 | 91.1 | + | 4.711983333 | 0.000365233 | 0.000381448 | 0.000402936 | 0.000383206 | 0.000484584 | 0.000346834 | 0.000245473 | 0.000358964 | 0.000481928 | 0.000327054 | 0.000404491 |
| 888 | Phenylacetylglycine | 500-98-1 | C05598 | C10H11NO3 | 193.073894 | Amino acid and derivatives | 194.1 | 91.1 | + | 4.918116667 | 0.000235176 | 0.000284272 | 0.000296059 | 0.000271836 | 0.000158235 | 0.000189865 | 0.000394892 | 0.000247664 | 0.000325062 | 0.000449294 | 0.000387178 |
| 889 | Phillyrin;Phillyroside | 487-41-2 | C17048 | C27H34O11 | 534.21011499999997 | Phenylpropanoids | 535.2 | 373.2 | + | 7.76101825 | 0.000158503 | 0.000109732 | 8.73074E-05 | 0.000118514 | 0.000231587 | 0.000305403 | 0.000126936 | 0.000221309 | 0.000117836 | 0.000286736 | 0.000202286 |
| 890 | Phloretic acid;Ethylparaben;3-(2-Hydroxyphenyl)propanoic acid | 501-97-3;120-47-8;495-78-3 | C01744;C01198 | C9H10O3 | 166.062995 | Phenols;Phenylpropanoic acids | 167.1 | 121.0666667 | + | 2.840438889 | 0.051513191 | 0.04444227 | 0.046118795 | 0.047358085 | 0.047158808 | 0.04060403 | 0.038114471 | 0.041959103 | 0.046544366 | 0.044987343 | 0.045765854 |
| 891 | Phloretin | 60-82-2 | C00774 | C15H14O5 | 274.08409999999998 | flavonoids | 274.9 | 169 | + | 8.85 | 0.000431271 | 0.00035196 | 0.000402029 | 0.000395087 | 0.0002728 | 0.000310847 | 0.000188188 | 0.000257278 | 0.000295128 | 0.000398169 | 0.000346648 |
| 892 | Phosphonoacetate | 4408-78-0 | C05682 | C2H5O5P | 139.98746199999999 | Organic acids | 141 | 95 | + | 1.879933333 | 0.007216163 | 0.00699741 | 0.006308436 | 0.00684067 | 0.00887208 | 0.009370342 | 0.008609847 | 0.008950756 | 0.008268762 | 0.008736738 | 0.00850275 |
| 893 | Phosphoric acid | 7664-38-2 | C00009 | H3O4P | 97.976896999999994 | Organic acids | 99 | 81 | + | 0.64835 | 0.001333306 | 0.0026325 | 0.002372411 | 0.002112739 | 0.004816345 | 0.00359143 | 0.002533241 | 0.003647005 | 0.002996413 | 0.002769037 | 0.002882725 |
| 894 | Phosphorylcholine | 107-73-3 | C00588 | C5H14NO4P | 183.066046 | Cholines | 184.1 | 86.1 | + | 0.665866667 | 0.00366398 | 0.005699232 | 0.004545611 | 0.004636274 | 0.016785485 | 0.011014178 | 0.007302383 | 0.011700682 | 0.006068035 | 0.008662505 | 0.00736527 |
| 895 | Physalin A | 23027-91-0 |  | C28H30O10 | 526.18389999999999 | Steroids and steroid derivatives | 527.2 | 509.2 | + | 12.10071667 | 0.004049108 | 0.00292188 | 0.002133207 | 0.003034732 | 0.002392798 | 0.001842164 | 0.001775035 | 0.002003332 | 0.003761916 | 0.00242161 | 0.003091763 |
| 896 | Physalin C | 27503-33-9 |  | C28H30O9 | 510.188985 | Steroids | 511.2 | 493.2 | + | 3.121866667 | 3.39759E-05 | 9.03384E-05 | 4.21141E-05 | 5.54761E-05 | 5.32868E-05 | 6.36365E-05 | 7.54104E-05 | 6.41113E-05 | 5.57955E-05 | 0.00010777 | 8.17829E-05 |
| 897 | Physalin D | 54980-22-2 |  | C28H32O11 | 544.19446500000004 | Steroids and steroid derivatives | 545.2 | 527.2 | + | 12.10071667 | 0.009368521 | 0.007593282 | 0.004982993 | 0.007314932 | 0.007237663 | 0.008724103 | 0.006150447 | 0.007370738 | 0.006736378 | 0.00611027 | 0.006423324 |
| 898 | Physalin G | 76045-38-0 |  | C28H30O10 | 526.18389999999999 | Steroids and steroid derivatives | 527.2 | 509.2 | + | 12.16785 | 0.004049108 | 0.00292188 | 0.004520311 | 0.003830433 | 0.002392798 | 0.001842164 | 0.001775035 | 0.002003332 | 0.003761916 | 0.00242161 | 0.003091763 |
| 899 | Physcion 1-O-beta-D-glucoside;Physcion 8-O-beta-D-monoglucoside;Sissotrin | 26296-54-8;23451-01-6;5928-26-7 | C10384;C05376 | C22H22O10 | 446.12130000000002 | Anthraquinones;Flavonoids | 447.1 | 267.1 | + | 6.701383333 | 6.05707E-05 | 0.000193618 | 0.000221341 | 0.00015851 | 0.000224645 | 0.000176204 | 0.000137194 | 0.000179347 | 0.000251298 | 0.000235756 | 0.000243527 |
| 901 | Phytanic acid | 14721-66-5 | C01607 | C20H40O2 | 312.30282999999997 | Lipids | 313.3 | 57.1 | + | 11.20246667 | 0.003915877 | 0.003276044 | 0.002295382 | 0.003162434 | 0.002649669 | 0.002155105 | 0.002793814 | 0.002532863 | 0.002962272 | 0.002004377 | 0.002483324 |
| 902 | Picrocrocin | 138-55-6 | C17055 | C16H26O7 | 330.16785499999997 | Monoterpenoids | 331.2 | 151.1 | + | 9.247616667 | 3.31592E-05 | 9.35518E-05 | 7.42028E-05 | 6.69713E-05 | 0.000163515 | 6.04914E-05 | 3.84502E-05 | 8.74854E-05 | 8.23855E-05 | 0.000115657 | 9.90213E-05 |
| 903 | Pilocarpine | 92-13-7 | C07474 | C11H16N2O2 | 208.12117799999999 | Alkaloids | 209.1 | 95.1 | + | 5.037916667 | 0.000286496 | 0.001055021 | 0.00051847 | 0.000619996 | 0.00125385 | 0.000533945 | 0.001200259 | 0.000996018 | 0.000904639 | 0.000678048 | 0.000791343 |
| 904 | Pimelic acid | 111-16-0 | C02656 | C7H12O4 | 160.07355999999999 | Fatty Acyls | 161.1 | 69.1 | + | 0.551616667 | 0.007862436 | 0.005301857 | 0.011167328 | 0.00811054 | 0.012866193 | 0.011067973 | 0.011187231 | 0.011707132 | 0.009851437 | 0.009662532 | 0.009756984 |
| 905 | Pinoresinol dimethyl ether | 29106-36-3 | C10561 | C22H26O6 | 386.17293999999998 | Lignans | 387.2 | 121.1 | + | 5.1526 | 0.001372295 | 0.001700444 | 0.002505263 | 0.001859334 | 0.005067576 | 0.002266492 | 0.003536516 | 0.003623528 | 0.001428181 | 0.002787977 | 0.002108079 |
| 906 | Pinostilbenoside | 58762-96-2 |  | C21H24O8 | 404.14711999999997 | Phenols | 405.2 | 225.1 | + | 11.19018333 | 6.65015E-05 | 5.78509E-05 | 5.8602E-05 | 6.09848E-05 | 9.86151E-05 | 4.62214E-05 | 6.95797E-05 | 7.14721E-05 | 0.00010472 | 0.000117922 | 0.000111321 |
| 907 | Piperidine | 110-89-4 | C01746 | C5H11N | 85.089149000000006 | Alkaloids | 86.1 | 69.1 | + | 2.24915 | 0.000188747 | 0.000160651 | 0.000124318 | 0.000157905 | 0.000179608 | 0.000162368 | 9.91768E-05 | 0.000147051 | 5.89888E-05 | 8.37126E-05 | 7.13507E-05 |
| 908 | Piperitenone | 491-09-8 | C01951 | C10H14O | 150.104465 | Prenol lipids | 151.1 | 39 | + | 9.918933333 | 2.87677E-05 | 8.68846E-05 | 5.18874E-05 | 5.58466E-05 | 4.03202E-05 | 6.34286E-05 | 4.04549E-05 | 4.80679E-05 | 0.00010165 | 4.70462E-05 | 7.43482E-05 |
| 909 | Piperlonguminine | 5950-12-9 |  | C16H19NO3 | 273.13649400000003 | Alkaloids | 274.1 | 57.1 | + | 10.57346667 | 0.000472703 | 0.000115246 | 0.000203952 | 0.000263967 | 0.000289151 | 0.000389733 | 0.000256742 | 0.000311875 | 0.000239651 | 0.000548447 | 0.000394049 |
| 910 | Pipermethystine | 71627-22-0 |  | C16H17NO4 | 287.11575900000003 | Alkaloids | 288.1 | 228.1 | + | 7.569333333 | 2.41779E-05 | 9.9953E-05 | 4.32626E-05 | 5.57978E-05 | 6.71271E-05 | 0.000110216 | 0.000134519 | 0.000103954 | 5.58557E-05 | 8.34007E-05 | 6.96282E-05 |
| 911 | Plantagoside | 78708-33-5 | C17531 | C21H22O12 | 466.11113 | Flavonoids | 467.1 | 287.1 | + | 6.847666667 | 0.000220965 | 0.000187815 | 0.000152334 | 0.000187038 | 0.000450827 | 0.000239313 | 7.60895E-05 | 0.00025541 | 0.000221325 | 0.000193254 | 0.000207289 |
| 912 | Plumbagin | 481-42-5 | C10387 | C11H8O3 | 188.04734500000001 | Quinones | 189.1 | 41 | + | 8.72735 | 3.1017E-05 | 0.000154643 | 7.06007E-05 | 8.54203E-05 | 0.000127781 | 8.83628E-05 | 0.000118796 | 0.000111647 | 4.25901E-05 | 0.000109634 | 7.61119E-05 |
| 913 | Plumieride | 511-89-7 | C09797 | C21H26O12 | 470.14242999999999 | Iridoids | 471.1 | 435.1 | + | 6.595933333 | 6.02394E-06 | 3.43155E-05 | 6.99013E-05 | 3.67469E-05 | 7.7514E-05 | 4.65168E-05 | 3.9775E-05 | 5.46019E-05 | 0.000197042 | 6.67806E-05 | 0.000131911 |
| 914 | Podophyllotoxin | 518-28-5 | C10874 | C22H22O8 | 414.13146999999998 | Lignans | 415.1 | 397.1 | + | 9.6560814 | 9.47564E-05 | 8.52254E-05 | 6.60864E-05 | 8.20227E-05 | 9.60305E-05 | 6.32563E-05 | 6.60702E-05 | 7.5119E-05 | 0.000132086 | 0.00012504 | 0.000128563 |
| 915 | Podophyllotoxinone | 477-49-6 | C10875 | C22H20O8 | 412.11581999999999 | Lignans | 413.1 | 395.1 | + | 5.421116667 | 0.000157786 | 4.38659E-05 | 0.000125432 | 0.000109028 | 0.000412271 | 9.50729E-05 | 0.000106861 | 0.000204735 | 0.000295343 | 0.000317283 | 0.000306313 |
| 916 | Poncirin | 14941-08-3 | C09830 | C28H34O14 | 594.19485999999995 | Flavonoids | 595.2 | 269.1 | + | 8.156733333 | 0.000136282 | 0.000114246 | 8.88007E-05 | 0.000113109 | 4.6658E-05 | 5.65292E-05 | 1.75091E-05 | 4.02321E-05 | 7.27932E-05 | 9.10782E-05 | 8.19357E-05 |
| 918 | Porson | 56222-03-8 |  | C22H26O6 | 386.17293999999998 | Phenols | 387.2 | 313.1 | + | 7.45185 | 5.93674E-05 | 6.95383E-05 | 3.07101E-05 | 5.32053E-05 | 0.000124641 | 5.22528E-05 | 3.70959E-05 | 7.13298E-05 | 3.15238E-05 | 5.99168E-05 | 4.57203E-05 |
| 919 | Practolol | 6673-35-4 | C11696 | C14H22N2O3 | 266.16304300000002 | Benzene and substituted derivatives | 267.2 | 56.1 | + | 2.735866667 | 6.68157E-05 | 0.000121614 | 0.000112455 | 0.000100295 | 6.50436E-05 | 9.97894E-05 | 0.000101588 | 8.88072E-05 | 5.09981E-05 | 7.6451E-05 | 6.37245E-05 |
| 920 | Pregabalin | 148553-50-8 |  | C8H17NO2 | 159.12592900000001 | Miscellaneous | 160.1 | 55.1 | + | 0.68835 | 1.9227E-05 | 4.21776E-05 | 0.000142132 | 6.78456E-05 | 0.000384249 | 0.000117037 | 0.000222231 | 0.000241172 | 0.000169558 | 0.000257098 | 0.000213328 |
| 921 | Primin | 15121-94-5 | C10390 | C12H16O3 | 208.10994500000001 | Quinones | 209.1 | 43.1 | + | 5.169383333 | 0.000461064 | 0.000864599 | 0.000674736 | 0.0006668 | 0.001022954 | 0.001103524 | 0.000777263 | 0.000967914 | 0.00059731 | 0.000528895 | 0.000563102 |
| 922 | Pristimerin | 1258-84-0 | C08633 | C30H40O4 | 464.29266000000001 | Triterpenoids | 465.3 | 201.1 | + | 6.8515 | 0.000115703 | 9.22059E-05 | 7.1204E-05 | 9.30377E-05 | 0.000119678 | 8.83497E-05 | 0.000133142 | 0.000113723 | 4.49404E-05 | 9.70847E-05 | 7.10125E-05 |
| 923 | Procyanidin B2 | 29106-49-8 | C17639 | C30H26O12 | 578.14239999999995 | flavonoids | 579 | 427 | + | 4.78 | 5.68002E-06 | 1.27649E-06 | 1.12648E-06 | 2.69433E-06 | 7.9032E-06 | 2.271E-05 | 0.000125413 | 5.20089E-05 | 3.25824E-05 | 5.16852E-05 | 4.21338E-05 |
| 924 | Procyanidin B4 | 29106-51-2 | C10238 | C30H26O12 | 578.14242999999999 | Flavonoids | 579.1 | 123 | + | 11.84896667 | 0.000444657 | 0.00063598 | 0.000775382 | 0.000618673 | 0.000465703 | 0.000751709 | 0.002007137 | 0.001074849 | 0.002870734 | 0.001980287 | 0.00242551 |
| 926 | Proline betaine | 471-87-4 | C10172 | C7H13NO2 | 143.094629 | Carboxylic acids and derivatives | 144.1 | 41 | + | 0.7387 | 0.021838196 | 0.008800945 | 0.01235063 | 0.014329924 | 0.023012868 | 0.019223175 | 0.000551353 | 0.014262465 | 0.014818535 | 0.014613272 | 0.014715903 |
| 927 | Proline;L-Proline | 147-85-3 | C00148 | C5H9NO2 | 115.063329;115.0633 | Amino acid and derivatives;amino acids | 116.1 | 70.1 | + | 0.73 | 0.085511244 | 0.110738114 | 0.091153827 | 0.095801062 | 0.12822575 | 0.107252023 | 0.0974512 | 0.110976325 | 0.106280669 | 0.099763498 | 0.103022083 |
| 928 | Prostaglandin E3 | 802-31-3 | C06439 | C20H30O5 | 350.20932499999998 | Fatty Acyls | 349.2 | 269.2 | - | 4.810516667 | 4.25429E-05 | 0.000135681 | 9.70643E-05 | 9.17629E-05 | 4.88769E-05 | 0.000244576 | 8.48089E-05 | 0.000126087 | 8.80975E-05 | 5.77786E-05 | 7.29381E-05 |
| 929 | Prostaglandin I2 | 35121-78-9 | C01312 | C20H32O5 | 352.22497499999997 | Fatty Acyls | 353.2 | 317.2 | + | 9.389483333 | 4.01257E-05 | 8.06216E-05 | 4.99528E-05 | 5.69E-05 | 9.53408E-05 | 4.53009E-05 | 1.9246E-05 | 5.32959E-05 | 6.19257E-05 | 3.06643E-05 | 4.6295E-05 |
| 930 | Protocatechualdehyde | 139-85-5 | C16700 | C7H6O3 | 138.0317 | flavonoids | 138.9 | 120.8 | + | 4.2 | 0.049541386 | 0.047042574 | 0.044989669 | 0.04719121 | 0.067007893 | 0.059646033 | 0.032749503 | 0.053134476 | 0.056801421 | 0.057900923 | 0.057351172 |
| 931 | Protopine | 130-86-9 | C05189 | C20H19NO5 | 353.12632400000001 | Alkaloids | 354.1 | 188.1 | + | 11.44618333 | 0.002754824 | 0.003759958 | 0.002804002 | 0.003106261 | 1.59752E-06 | 0.00273284 | 0.003089618 | 0.001941352 | 0.002908552 | 0.004132963 | 0.003520757 |
| 934 | Psychosine | 2238-90-6 | C01747 | C24H47NO7 | 461.33525400000002 | Sphingolipids | 460.3 | 59 | - | 7.6553 | 0.000149303 | 5.25742E-05 | 3.91953E-05 | 8.03576E-05 | 8.41E-05 | 5.09498E-05 | 6.70388E-05 | 6.73629E-05 | 0.000144465 | 0.00012032 | 0.000132393 |
| 935 | Pterosin D | 34169-70-5 |  | C15H20O3 | 248.141245 | Sesquiterpenoids | 249.1 | 231.1 | + | 3.2902 | 4.59587E-05 | 9.45712E-05 | 5.77343E-05 | 6.60881E-05 | 5.69544E-05 | 4.18578E-05 | 1.88757E-05 | 3.92293E-05 | 4.957E-05 | 5.88098E-05 | 5.41899E-05 |
| 936 | Pyridoxal 5'-phosphate | 54-47-7 | C00018 | C8H10NO6P | 247.024576 | Pyridines and derivatives | 248 | 150.1 | + | 1.80455 | 1.93087E-05 | 3.16924E-05 | 2.2454E-05 | 2.44851E-05 | 3.98123E-05 | 2.3824E-05 | 1.67809E-05 | 2.68057E-05 | 4.4636E-05 | 1.84813E-05 | 3.15587E-05 |
| 937 | Pyridoxine | 65-23-6 | C00314 | C8H11NO3 | 169.073894 | Vitamins | 170.1 | 134.1 | + | 1.55165 | 0.007401769 | 0.008676388 | 0.007084208 | 0.007720788 | 0.007250208 | 0.006707692 | 0.006026968 | 0.006661622 | 0.006715623 | 0.007429757 | 0.00707269 |
| 939 | Pyrogallol;1,2,3-Trihydroxybenzene | 87-66-1 | C01108 | C6H6O3 | 126.031695 | Phenols | 127 | 81 | + | 3.52465 | 0.000570598 | 0.001289588 | 0.000251094 | 0.00070376 | 0.000416155 | 0.000309911 | 0.00124962 | 0.000658562 | 0.000307269 | 0.001211223 | 0.000759246 |
| 940 | Pyrrolidonecarboxylic acid | 4042-36-8 | C02237 | C5H7NO3 | 129.04259400000001 | Carboxylic acids and derivatives | 130 | 84 | + | 0.671566667 | 0.037522944 | 0.035692494 | 0.040331209 | 0.037848883 | 0.087631726 | 0.055948419 | 0.09091796 | 0.078166035 | 0.056374605 | 0.068405252 | 0.062389929 |
| 941 | Quercetagetin | 90-18-6 | C10122 | C15H10O8 | 318.03757000000002 | Flavonoids | 319 | 109 | + | 11.58045 | 0.000373019 | 0.000569806 | 0.000545583 | 0.000496136 | 0.000985824 | 0.000720544 | 0.000609265 | 0.000771877 | 0.00113707 | 0.000492723 | 0.000814897 |
| 942 | Quercetin | 117-39-5 | C00389 | C15H10O7 | 302.04265500000002 | Flavonoids | 303 | 153 | + | 8.07 | 6.51528E-05 | 8.28536E-05 | 0.000125644 | 9.12168E-05 | 0.000109838 | 0.000128385 | 8.63346E-05 | 0.000108186 | 0.000113728 | 8.30819E-05 | 9.84052E-05 |
| 944 | Quercetin-3-O-glucuronide | 22688-79-5 |  | C21H18O13 | 478.07474500000001 | Flavonoids | 479.1 | 303.1 | + | 5.924616667 | 0.000132797 | 3.37201E-05 | 5.69508E-05 | 7.44893E-05 | 3.44256E-05 | 1.3166E-05 | 3.84898E-05 | 2.86938E-05 | 4.39669E-05 | 0.000136727 | 9.03471E-05 |
| 945 | Quercetin-3-O-sophoroside | 18609-17-1 | C12667 | C27H30O17 | 626.14830500000005 | Flavonoids | 627.2 | 303.1 | + | 6.243483333 | 4.16814E-05 | 4.44975E-05 | 3.38969E-05 | 4.00253E-05 | 3.76889E-05 | 7.02124E-05 | 5.36679E-05 | 5.38564E-05 | 6.36427E-05 | 4.13359E-05 | 5.24893E-05 |
| 946 | Quercetin-7-O-beta-D-glucopyranoside | 491-50-9 | C12639 | C21H20O12 | 464.09548000000001 | Flavonoids | 465.1 | 285 | + | 6.1007 | 0.000135248 | 0.000235229 | 0.00022592 | 0.000198799 | 0.00023306 | 0.00024027 | 0.000338475 | 0.000270602 | 0.000352338 | 0.00015974 | 0.000256039 |
| 947 | Quercitrin | 522-12-3 | C01750 | C21H20O11 | 448.10059999999999 | flavonoids | 446.9 | 300.8 | - | 6.59 | 0.000265096 | 0.00033684 | 0.000353113 | 0.00031835 | 0.000293175 | 0.000267081 | 0.000305114 | 0.000288457 | 0.000224076 | 0.000254837 | 0.000239456 |
| 948 | Queuine | 72496-59-4 | C01449 | C12H15N5O3 | 277.11748999999998 | Pyrrolopyrimidines | 278.1 | 163.1 | + | 11.54688333 | 1.18251E-06 | 1.27649E-06 | 0.000260993 | 8.78174E-05 | 1.59752E-06 | 1.26522E-06 | 1.23455E-06 | 1.36576E-06 | 0.000113911 | 0.000113209 | 0.00011356 |
| 949 | Raffinose | 512-69-6 | C00492 | C18H32O16 | 504.16904 | Organooxygen compounds | 503.2 | 89 | - | 1.305266667 | 2.22546E-05 | 1.27649E-06 | 0.000123541 | 4.90241E-05 | 2.29892E-05 | 7.22397E-05 | 0.000303219 | 0.000132816 | 0.000200542 | 0.000138426 | 0.000169484 |
| 950 | Rapanone | 573-40-0 | C10399 | C19H30O4 | 322.21440999999999 | Quinones | 323.2 | 43.1 | + | 12.82238333 | 0.000206597 | 0.000512928 | 6.80357E-05 | 0.00026252 | 0.000901035 | 0.000172066 | 0.000188705 | 0.000420602 | 0.000380542 | 0.000613821 | 0.000497182 |
| 951 | Resokaempferol | 2034-65-3 | C10037 | C15H10O5 | 270.05282499999998 | Flavonoids | 271.1 | 105 | + | 8.794483333 | 0.000452138 | 0.000369856 | 0.000386957 | 0.000402984 | 0.000579939 | 0.000581262 | 0.000174443 | 0.000445215 | 0.000215462 | 0.000252243 | 0.000233853 |
| 952 | Reticuline;(R)-Reticuline | 485-19-8;3968-19-2 | C02105;C12328;C05178 | C19H23NO4 | 329.16270900000001 | Alkaloids | 330.2 | 149.1 | + | 5.239766667 | 0.000111956 | 4.60298E-05 | 1.50711E-05 | 5.76857E-05 | 0.000113334 | 6.69592E-05 | 6.51537E-05 | 8.18156E-05 | 6.36843E-05 | 7.18885E-05 | 6.77864E-05 |
| 953 | Retronecine | 480-85-3 | C06177 | C8H13NO2 | 155.094629 | Alkaloids | 156.1 | 138.1 | + | 3.004383333 | 7.47278E-05 | 0.000113745 | 7.19745E-05 | 8.68159E-05 | 0.000198759 | 3.15061E-05 | 1.10039E-05 | 8.04229E-05 | 0.000115311 | 4.64079E-05 | 8.08593E-05 |
| 954 | Rhein | 478-43-3 | C10401 | C15H8O6 | 284.03208999999998 | Anthraquinones | 283 | 239 | - | 8.03205 | 3.07954E-05 | 1.79425E-05 | 3.55812E-05 | 2.81064E-05 | 2.80287E-05 | 4.43522E-05 | 5.25169E-05 | 4.16326E-05 | 3.44603E-05 | 4.1214E-05 | 3.78371E-05 |
| 955 | Rhoifolin | 17306-46-6 | C12627 | C27H30O14 | 578.16359999999997 | flavonoids | 579 | 433 | + | 6.5 | 0.515872766 | 0.039082186 | 0.064741384 | 0.206565445 | 0.125669171 | 0.502909992 | 0.475038871 | 0.367872678 | 0.738583838 | 0.624396079 | 0.681489959 |
| 956 | Rhombifoline | 529-78-2 | C10781 | C15H20N2O | 244.15756300000001 | Alkaloids | 245.2 | 55.1 | + | 8.744133333 | 8.9016E-05 | 0.000202935 | 0.000437215 | 0.000243055 | 0.000318652 | 0.000175118 | 0.000134924 | 0.000209564 | 0.000198478 | 0.000148908 | 0.000173693 |
| 958 | Riboflavine | 83-88-5 | C00255 | C17H20N4O6 | 376.13828599999999 | Vitamins | 377.1 | 243.1 | + | 5.0561 | 0.136204514 | 0.146225302 | 0.136911423 | 0.139780413 | 0.171941945 | 0.108918343 | 0.119713925 | 0.133524738 | 0.157524249 | 0.159363796 | 0.158444022 |
| 959 | Riddelline | 23246-96-0 | C10375 | C18H23NO6 | 349.15253899999999 | Alkaloids | 350.2 | 332.1 | + | 13.65601667 | 0.000102408 | 0.00011141 | 0.000129224 | 0.000114348 | 0.000183511 | 0.000196297 | 0.000129841 | 0.000169883 | 0.000214533 | 0.00014799 | 0.000181262 |
| 960 | Rivularine | 723-78-4 | C10278 | C13H19NO3 | 237.136494 | Alkaloids | 238.1 | 55.1 | + | 4.347016667 | 3.6455E-05 | 3.42824E-05 | 2.85567E-05 | 3.30981E-05 | 6.60491E-05 | 9.02205E-05 | 5.92052E-05 | 7.18249E-05 | 4.09424E-05 | 0.000136309 | 8.86255E-05 |
| 961 | Robinetin | 490-31-3 | C10177 | C15H10O7 | 302.04265500000002 | Flavonoids | 303 | 137 | + | 6.828133333 | 0.005600391 | 0.007033591 | 0.013162995 | 0.008598992 | 0.001793434 | 0.015925939 | 0.017358267 | 0.011692547 | 0.001729034 | 0.008225475 | 0.004977254 |
| 962 | Rosmarinic acid | 537-15-5;20283-92-5 | C01850 | C18H16O8 | 360.08452 | Phenylpropanoids | 361.1 | 163 | + | 6.989966667 | 0.000166388 | 0.000109155 | 0.000143821 | 0.000139788 | 0.000128882 | 8.13814E-05 | 0.000127714 | 0.000112659 | 0.00016721 | 0.000162923 | 0.000165066 |
| 963 | Rosmarinine | 520-65-0 | C10380 | C18H27NO6 | 353.18383899999998 | Alkaloids | 354.2 | 336.2 | + | 9.381883333 | 5.36151E-05 | 3.74602E-05 | 3.02603E-05 | 4.04452E-05 | 3.55403E-05 | 3.62444E-05 | 4.72874E-05 | 3.96907E-05 | 5.97948E-05 | 2.62785E-05 | 4.30367E-05 |
| 964 | Rotenone | 83-79-4 | C07593 | C23H22O6 | 394.14164 | Flavonoids | 395.1 | 213.1 | + | 12.92374167 | 0.000123345 | 0.000137422 | 9.92213E-05 | 0.000119996 | 0.000203706 | 0.000225004 | 6.66112E-05 | 0.000165107 | 0.000308595 | 0.000146673 | 0.000227634 |
| 965 | Ruscogenin | 472-11-7 | C08909 | C27H42O4 | 430.30831000000001 | Steroids and steroid derivatives | 431.3 | 269.2 | + | 12.804993 | 0.000119801 | 0.000117263 | 0.000112107 | 0.000116391 | 7.60589E-05 | 6.26679E-05 | 2.92253E-05 | 5.5984E-05 | 0.00010083 | 6.49489E-05 | 8.28896E-05 |
| 966 | Rutacridone | 17948-33-3 | C10738 | C19H17NO3 | 307.12084399999998 | Alkaloids | 308.1 | 252.1 | + | 12.87273333 | 2.46378E-05 | 1.27649E-06 | 7.95083E-05 | 3.51409E-05 | 1.76613E-05 | 3.48207E-05 | 1.96133E-05 | 2.40317E-05 | 2.86905E-05 | 6.06274E-05 | 4.46589E-05 |
| 967 | Rutaevin | 33237-37-5 | C08779 | C26H30O9 | 486.188985 | Triterpenoids | 487.2 | 469.2 | + | 12.5814 | 0.003242202 | 0.003384769 | 0.001816825 | 0.002814598 | 0.004154513 | 0.004501447 | 0.004257393 | 0.004304451 | 0.006423152 | 0.005782671 | 0.006102911 |
| 968 | Rutin | 153-18-4 | C05625 | C27H30O16 | 610.15338999999994 | Flavonoids | 611.2 | 303.1 | + | 5.93544905 | 0.001475638 | 0.001821666 | 0.001082606 | 0.00145997 | 0.001805274 | 0.001219814 | 0.000598122 | 0.001207737 | 0.001610616 | 0.001187857 | 0.001399237 |
| 969 | S-(Phenylacetothiohydroximoyl)-L-cysteine | | C17237 | C11H14N2O3S | 254.07251400000001 |  | 255.1 | 91.1 | + | 11.56366667 | 0.000269699 | 0.001000488 | 0.000551541 | 0.000607243 | 0.000453711 | 0.000322682 | 0.000580447 | 0.00045228 | 0.000379214 | 0.000273455 | 0.000326334 |
| 970 | S-Adenosylmethionine | 29908-03-0 | C00019 | C15H22N6O5S | 398.13724000000002 | Amino acid and derivatives | 399.1 | 250.1 | + | 0.655383333 | 0.00035977 | 0.000584425 | 0.000587094 | 0.00051043 | 0.000775111 | 0.000841852 | 0.00064282 | 0.000753261 | 0.001092394 | 0.000952831 | 0.001022612 |
| 971 | S-Lactoylglutathione | 41656-56-8 | C03451 | C13H21N3O8S | 379.104938 | Carboxylic acids and derivatives | 380.1 | 262.1 | + | 4.092216667 | 1.81826E-05 | 0.000181522 | 3.62445E-05 | 7.86496E-05 | 4.5149E-05 | 4.98132E-05 | 0.000153519 | 8.2827E-05 | 3.55878E-05 | 0.000164224 | 9.99058E-05 |
| 972 | Safflor Yellow A | 85532-77-0 |  | C27H30O15 | 594.15847499999995 | Chalcones | 595.2 | 577.2 | + | 12.33566667 | 0.003341187 | 0.008611493 | 0.005654193 | 0.005868958 | 0.009105089 | 0.010016828 | 0.011129402 | 0.010083773 | 0.018806086 | 0.006520115 | 0.012663101 |
| 973 | Saikosaponin A | 20736-09-8 | C08975 | C42H68O13 | 780.46599500000002 | Triterpenoids | 781.5 | 455.4 | + | 10.82462406 | 1.18251E-06 | 6.37364E-05 | 1.60358E-05 | 2.69849E-05 | 5.40974E-05 | 0.000108037 | 8.16241E-06 | 5.67656E-05 | 0.000109633 | 2.37205E-05 | 6.66769E-05 |
| 974 | Sakuranetin | 2957-21-3 | C09833 | C16H14O5 | 286.08412499999997 | Flavonoids | 287.1 | 105 | + | 9.935716667 | 0.000766374 | 0.001120354 | 0.001020967 | 0.000969231 | 0.000717334 | 0.000950969 | 0.000837638 | 0.000835314 | 0.000625932 | 0.000738223 | 0.000682078 |
| 975 | Salannin | 992-20-1 | C08780 | C34H44O9 | 596.29853500000002 | Triterpenoids | 597.3 | 147.1 | + | 5.974966667 | 4.42837E-05 | 1.91544E-05 | 9.12182E-05 | 5.15521E-05 | 0.000243311 | 5.17038E-05 | 0.000174486 | 0.0001565 | 8.9677E-05 | 0.000247049 | 0.000168363 |
| 976 | Salidroside | 10338-51-9 | C06046 | C14H20O7 | 300.12090499999999 | Phenols | 299.1 | 59 | - | 4.176933333 | 0.000446222 | 0.000575688 | 0.000424025 | 0.000481978 | 0.000539279 | 0.000206371 | 0.001037865 | 0.000594505 | 0.000601051 | 0.000382192 | 0.000491621 |
| 978 | Salviaflaside | 178895-25-5 |  | C24H26O13 | 522.13734499999998 | Phenylpropanoids | 523.1 | 181.1 | + | 7.1162 | 7.12804E-05 | 0.000179122 | 0.000176542 | 0.000142315 | 0.000208226 | 0.00011498 | 0.000158272 | 0.000160493 | 0.00026159 | 6.65663E-05 | 0.000164078 |
| 979 | Salvianolic acid A | 96574-01-5 | C10492 | C26H22O10 | 494.12130000000002 | Phenylpropanoids | 495.1 | 181.1 | + | 7.745978835 | 2.4154E-05 | 6.58904E-05 | 1.61056E-05 | 3.53834E-05 | 2.63203E-05 | 1.45147E-05 | 1.7369E-05 | 1.94013E-05 | 2.79851E-05 | 5.43277E-05 | 4.11564E-05 |
| 980 | Samidin | 477-33-8 | C09310 | C21H22O7 | 386.13655499999999 | Coumarins | 387.1 | 55.1 | + | 5.935383333 | 0.000109139 | 4.27693E-05 | 7.90801E-05 | 7.69962E-05 | 9.76469E-05 | 6.70683E-05 | 4.34783E-05 | 6.93978E-05 | 6.0234E-05 | 4.58706E-05 | 5.30523E-05 |
| 981 | Saponarin | 20310-89-8 | C08064 | C27H30O15 | 594.15847499999995 | Flavonoids | 595.2 | 415.1 | + | 4.93855 | 0.000152942 | 0.000226527 | 0.000206226 | 0.000195232 | 0.000344933 | 9.84881E-05 | 5.76405E-05 | 0.000167021 | 0.00017665 | 0.000111526 | 0.000144088 |
| 982 | Sarracine | 2492-09-3 | C10383 | C18H27NO5 | 337.18892399999999 | Alkaloids | 338.2 | 55.1 | + | 13.14125 | 0.047967464 | 0.500611226 | 0.405966042 | 0.318181577 | 0.681353692 | 0.511195114 | 0.506309717 | 0.566286174 | 0.545050232 | 0.43878212 | 0.491916176 |
| 983 | Sarsasapogenin | 126-19-2 | C03963 | C27H44O3 | 416.32904500000001 | Steroids | 417.3 | 273.2 | + | 12.08393333 | 0.000335406 | 0.000632416 | 0.000193664 | 0.000387162 | 0.000368246 | 0.000363536 | 0.000409039 | 0.000380273 | 0.000225826 | 0.000149956 | 0.000187891 |
| 984 | Schaftoside | 51938-32-0 | C10181 | C26H28O14 | 564.14791000000002 | Flavonoids | 565.2 | 547.1 | + | 5.38755 | 0.000208386 | 0.000149584 | 0.000177633 | 0.000178534 | 0.000472139 | 0.000384584 | 0.000187606 | 0.00034811 | 0.000219762 | 0.000158672 | 0.000189217 |
| 985 | Schisandrol A | 7432-28-2 | C17064 | C24H32O7 | 432.21480500000001 | Lignans | 433.2 | 415.2 | + | 11.5798426 | 0.000181241 | 0.000392242 | 0.000634931 | 0.000402804 | 0.000283488 | 0.000371223 | 0.000234706 | 0.000296472 | 0.000344139 | 0.000243183 | 0.000293661 |
| 986 | Schisantherin B | 58546-55-7 | C17814 | C28H34O9 | 514.22028499999999 | Lignans | 515.2 | 83 | + | 12.358109 | 0.000495545 | 0.000518578 | 0.000472149 | 0.000495424 | 0.000771495 | 0.000956753 | 0.000635953 | 0.000788067 | 0.000968686 | 0.000604183 | 0.000786435 |
| 987 | Sciadopitysin | 521-34-6 | C10182 | C33H24O10 | 580.13694999999996 | Flavonoids | 581.1 | 563.1 | + | 12.82238333 | 0.006529198 | 0.003857824 | 0.009603423 | 0.006663481 | 0.024307104 | 0.005676944 | 0.006346695 | 0.012110248 | 0.020898493 | 0.005098495 | 0.012998494 |
| 988 | Scoparone | 120-08-1 | C09311 | C11H10O4 | 206.05790999999999 | Coumarins | 207.1 | 121 | + | 5.059166667 | 0.009267504 | 0.009863314 | 0.00764465 | 0.008925156 | 0.00990938 | 0.01024644 | 0.006798874 | 0.008984898 | 0.008838769 | 0.008607788 | 0.008723279 |
| 989 | Scopolamine | 51-34-3 | C01851 | C17H21NO4 | 303.14705900000001 | Alkaloids | 304.2 | 138.1 | + | 6.858283333 | 0.000285548 | 0.000188026 | 0.000139277 | 0.000204283 | 0.000151001 | 0.000164867 | 0.000244807 | 0.000186892 | 0.000308494 | 0.000731163 | 0.000519829 |
| 990 | Scopolin | 531-44-2 | C01527 | C16H18O9 | 354.09508499999998 | Phenylpropanoids | 355.1 | 193.1 | + | 9.986066667 | 7.08944E-05 | 0.000198268 | 0.000217956 | 0.000162373 | 5.14063E-05 | 6.70096E-05 | 0.00035353 | 0.000157315 | 0.000152835 | 6.43308E-05 | 0.000108583 |
| 991 | Scutellarein | 529-53-3 | C10184 | C15H10O6 | 286.04773999999998 | Flavonoids | 287.1 | 269 | + | 8.123166667 | 0.000476071 | 0.000450671 | 0.000119645 | 0.000348795 | 0.000859066 | 0.000571192 | 0.000126305 | 0.000518854 | 0.000423736 | 0.000836899 | 0.000630318 |
| 992 | Secoisolariciresinol monoglucoside;Mascaroside | 63320-67-2;55465-97-9 | C09132 | C26H36O11 | 524.22576500000002 | Lignans;Naphthofurans | 525.2 | 489.2 | + | 7.8966 | 0.000278355 | 0.000269034 | 6.46683E-05 | 0.000204019 | 0.000220957 | 0.000368119 | 0.000230872 | 0.000273316 | 0.000180884 | 0.000227359 | 0.000204122 |
| 993 | Senecionine | 130-01-8 | C06176 | C18H25NO5 | 335.17327399999999 | Alkaloids | 336.2 | 120.1 | + | 11.73148333 | 0.000289569 | 0.001073379 | 0.0007517 | 0.000704883 | 0.001000927 | 0.000965338 | 0.001256075 | 0.001074113 | 0.000382961 | 0.001060365 | 0.000721663 |
| 994 | Seneciphylline | 480-81-9 | C10391 | C18H23NO5 | 333.157624 | Alkaloids | 334.2 | 316.2 | + | 9.06 | 6.79897E-05 | 7.80716E-05 | 0.000134075 | 9.33789E-05 | 0.000149317 | 0.000151651 | 6.81587E-05 | 0.000123042 | 0.000355897 | 7.11548E-05 | 0.000213526 |
| 995 | Serotonin | 50-67-9 | C00780 | C10H12N2O | 176.09496300000001 | Tryptamine derivatives | 177.1 | 160.1 | + | 2.612366667 | 0.0001607 | 0.000450903 | 0.000607117 | 0.00040624 | 0.000794252 | 0.000587204 | 0.000357376 | 0.00057961 | 0.000295131 | 0.000887703 | 0.000591417 |
| 996 | Sesamin | 607-80-7 | C10882 | C20H18O6 | 354.11034000000001 | Lignans | 355.1 | 135 | + | 10.09158257 | 0.00011615 | 5.06898E-05 | 6.42905E-05 | 7.70436E-05 | 0.000126389 | 0.000146807 | 7.80681E-05 | 0.000117088 | 0.000127948 | 0.000159027 | 0.000143488 |
| 997 | Sesartemin | 77394-27-5 | C10884 | C23H26O8 | 430.16277000000002 | Lignans | 431.2 | 169.1 | + | 11.90358333 | 0.00020978 | 0.000368395 | 0.000165752 | 0.000247975 | 6.91626E-05 | 0.000113783 | 0.000273897 | 0.000152281 | 0.00029322 | 0.00020897 | 0.000251095 |
| 1001 | Shionone | 10376-48-4 | C17966 | C30H50O | 426.38616500000001 | Triterpenoids | 427.4 | 409.4 | + | 12.00001667 | 0.000345367 | 0.000361683 | 0.000402465 | 0.000369838 | 0.000591326 | 0.000203818 | 0.000421621 | 0.000405588 | 0.000622758 | 0.000110453 | 0.000366606 |
| 1002 | Silibinin | 22888-70-6 | C07610 | C25H22O10 | 482.12130000000002 | flavonoids | 483.5 | 464.8 | + | 8.65 | 0.000457591 | 0.000302797 | 0.0002188 | 0.000326396 | 0.000428185 | 0.000331858 | 0.000330062 | 0.000363368 | 0.000502325 | 0.000405051 | 0.000453688 |
| 1003 | Simiarenol | 1615-94-7 | C17901 | C30H50O | 426.38616500000001 | Triterpenoids | 427.4 | 409.4 | + | 12.65455 | 0.000535214 | 0.0011432 | 0.000230994 | 0.00063647 | 0.000465122 | 0.000389808 | 0.000274165 | 0.000376365 | 0.000473454 | 0.000261074 | 0.000367264 |
| 1004 | Simvastatin | 79902-63-9 | C07262 | C25H38O5 | 418.27192500000001 | Diterpenoids | 419.3 | 199.1 | + | 8.074633333 | 3.12545E-05 | 6.52863E-05 | 5.81859E-05 | 5.15756E-05 | 6.44392E-05 | 0.000107551 | 7.08327E-05 | 8.09409E-05 | 4.73572E-05 | 7.4146E-05 | 6.07516E-05 |
| 1005 | Sinapic acid | 530-59-6;7362-37-0 | C00482 | C11H12O5 | 224.06847500000001 | Phenylpropanoids | 223.1 | 208 | - | 6.237101 | 0.004192463 | 0.007171973 | 0.009230886 | 0.006865108 | 0.010861035 | 0.008689125 | 0.007069688 | 0.008873282 | 0.006571144 | 0.009197647 | 0.007884396 |
| 1006 | Sinapyl alcohol | 537-33-7 | C02325 | C11H14O4 | 210.08921000000001 | Hydroxycinnamoyl derivatives | 193.1 | 161.1 | + | 4.430933333 | 0.000721401 | 0.00066272 | 0.00050988 | 0.000631334 | 0.001034053 | 0.001381814 | 0.001117867 | 0.001177911 | 0.001121391 | 0.000839396 | 0.000980393 |
| 1007 | sinensetin | 2306-27-6 | C10186 | C20H20O7 | 372.12090000000001 | flavonoids | 373 | 357.4 | + | 10.34 | 5.13726E-05 | 2.95077E-05 | 4.47687E-05 | 4.1883E-05 | 5.48177E-05 | 4.40046E-05 | 4.17428E-05 | 4.6855E-05 | 0.000112323 | 5.34241E-05 | 8.28735E-05 |
| 1008 | Sinigrin | 3952-98-5 | C08427 | C10H17NO9S2 | 359.03447599999998 | Organooxygen compounds | 358 | 97 | - | 5.092183333 | 3.61089E-05 | 3.4422E-05 | 6.27687E-05 | 4.44332E-05 | 7.79031E-05 | 8.18366E-05 | 0.00011819 | 9.26433E-05 | 3.38815E-05 | 0.000100359 | 6.71204E-05 |
| 1010 | Solasodine | 126-17-0 | C10822 | C27H43NO2 | 413.32937900000002 | Alkaloids | 414.3 | 396.3 | + | 11.8389 | 0.000140476 | 0.00028562 | 8.10754E-05 | 0.000169057 | 0.00013656 | 0.000394642 | 0.00019497 | 0.000242057 | 0.000230286 | 0.000360222 | 0.000295254 |
| 1011 | Sphingosine | 123-78-4 | C00319 | C18H37NO2 | 299.28242899999998 | Organonitrogen compounds | 300.3 | 282.3 | + | 11.94763333 | 6.33125E-05 | 0.000216975 | 0.000268433 | 0.000182907 | 1.59752E-06 | 6.76354E-05 | 1.23455E-06 | 2.34892E-05 | 0.00015452 | 0.000308295 | 0.000231408 |
| 1012 | Sphondin | 483-66-9 | C18081 | C12H8O4 | 216.04226 | Coumarins | 217 | 175 | + | 8.741166667 | 3.71921E-05 | 0.000284934 | 3.70979E-05 | 0.000119741 | 0.000164569 | 3.23986E-05 | 6.2795E-05 | 8.65877E-05 | 9.27769E-05 | 4.69151E-05 | 6.9846E-05 |
| 1013 | Stachyose | 10094-58-3;470-55-3 | C01613 | C24H42O21 | 666.22186499999998 | Organooxygen compounds | 689.2 | 527.2 | + | 0.727683333 | 0.000113578 | 2.25212E-05 | 1.93712E-05 | 5.18234E-05 | 0.000335211 | 0.000117204 | 0.000219129 | 0.000223848 | 0.000117694 | 7.81446E-05 | 9.79192E-05 |
| 1015 | Sterebin E | 114343-74-7 |  | C20H34O4 | 338.24570999999997 | Diterpenoids | 339.3 | 303.2 | + | 13.1931 | 9.16589E-05 | 4.5361E-05 | 5.4919E-05 | 6.39796E-05 | 8.37842E-05 | 6.81518E-05 | 7.83728E-05 | 7.67696E-05 | 0.000145074 | 0.000147363 | 0.000146219 |
| 1016 | Stigmasterol | 83-48-7 | C05442 | C29H48O | 412.37051500000001 | Steroids and steroid derivatives | 395.4 | 83.1 | + | 13.12098333 | 0.000639583 | 0.000401212 | 0.000381395 | 0.000474063 | 0.000379745 | 0.000200906 | 0.000367298 | 0.000315983 | 0.000315227 | 0.000426871 | 0.000371049 |
| 1017 | Streptomycin | 57-92-1 | C00413 | C21H39N7O12 | 581.26567299999999 | Organooxygen compounds | 582.3 | 263.1 | + | 12.83896667 | 0.000922309 | 0.001602305 | 0.001155871 | 0.001226829 | 0.002918864 | 0.001364552 | 0.001464798 | 0.001916071 | 0.003003619 | 0.001687831 | 0.002345725 |
| 1018 | Streptozotocin | 18883-66-4 | C07313 | C8H15N3O7 | 265.091002 | Organooxygen compounds | 266.1 | 140.1 | + | 0.71665 | 0.002220887 | 0.00342023 | 0.002243889 | 0.002628335 | 0.005255061 | 0.004308058 | 0.005189052 | 0.00491739 | 0.003651831 | 0.004859598 | 0.004255714 |
| 1019 | Strychnine | 57-24-9 | C06522 | C21H22N2O2 | 334.16812800000002 | Alkaloids | 335.2 | 184.1 | + | 3.674 | 8.27358E-05 | 0.000110396 | 2.536E-05 | 7.28306E-05 | 5.6117E-05 | 4.18295E-05 | 7.42443E-05 | 5.73969E-05 | 8.57246E-05 | 3.9642E-05 | 6.26833E-05 |
| 1020 | Styrene-cis-2,3-dihydrodiol | | C07084 | C8H10O2 | 138.06808000000001 |  | 139.1 | 39 | + | 5.119033333 | 0.000124911 | 0.000207192 | 0.000114964 | 0.000149022 | 0.000161397 | 0.000114279 | 0.000108342 | 0.000128006 | 0.000201577 | 0.000157329 | 0.000179453 |
| 1021 | Sucrose | 57-50-1 | C00089 | C12H22O11 | 342.11621500000001 | Carbohydrates | 365.1 | 203.1 | + | 0.8918 | 0.005102065 | 0.005008031 | 0.005061938 | 0.005057345 | 0.017284764 | 0.013223925 | 0.011440045 | 0.013982911 | 0.012389093 | 0.008000565 | 0.010194829 |
| 1022 | Sulforaphane | 4478-93-7 |  | C6H11NOS2 | 177.02820600000001 | Miscellaneous | 178 | 72 | + | 0.66155 | 0.006603476 | 0.007531065 | 0.007892665 | 0.007342402 | 0.009186122 | 0.001386571 | 0.008920032 | 0.006497575 | 0.005735763 | 0.006845607 | 0.006290685 |
| 1023 | Sulfuretin | 120-05-8 | C08730 | C15H10O5 | 270.05282499999998 | Flavonoids | 271.1 | 121 | + | 8.895183333 | 7.82157E-05 | 0.000218685 | 0.00025619 | 0.000184364 | 0.000208808 | 0.000109826 | 0.000183603 | 0.000167413 | 9.38227E-05 | 9.99557E-05 | 9.68892E-05 |
| 1024 | Sweroside | 14215-86-2 | C17071 | C16H22O9 | 358.12638500000003 | Iridoids;Terpene | 359.1 | 109 | + | 5.272940667 | 0.00034808 | 0.000185959 | 0.000400353 | 0.000311464 | 0.000620686 | 0.000268399 | 0.000659769 | 0.000516284 | 0.000152032 | 0.000707382 | 0.000429707 |
| 1025 | Swertiajaponin | 6980-25-2 | C10187 | C22H22O11 | 462.11621500000001 | Flavonoids | 463.1 | 445.1 | + | 13.86291667 | 0.001406076 | 0.001113412 | 0.001191851 | 0.001237113 | 0.001294894 | 0.000468382 | 0.001101735 | 0.000955004 | 0.00096701 | 0.001109059 | 0.001038034 |
| 1026 | Swertiamarin | 17388-39-5 | C09800 | C16H22O10 | 374.12130000000002 | Iridoids | 375.1 | 195.1 | + | 4.822477585 | 0.000160695 | 0.000256175 | 0.000110172 | 0.00017568 | 0.000248725 | 0.000317188 | 0.000238188 | 0.000268034 | 0.000137513 | 0.000128228 | 0.000132871 |
| 1027 | Swertiaperennin | 22172-17-4 | C10083 | C15H12O6 | 288.06339000000003 | Xanthones | 289.1 | 165 | + | 5.119033333 | 0.00028074 | 0.000259204 | 5.15999E-05 | 0.000197181 | 0.000702134 | 0.000202394 | 4.04744E-05 | 0.000315001 | 0.000123802 | 0.000333463 | 0.000228633 |
| 1028 | Symlandine;Symphytine | 74410-74-5;22571-95-5 | C10408;C10409 | C20H31NO6 | 381.21513900000002 | Alkaloids | 382.2 | 55.1 | + | 13.00698333 | 0.00025157 | 0.000141005 | 0.000127061 | 0.000173212 | 0.000424541 | 0.00026401 | 0.000311379 | 0.00033331 | 0.000255772 | 0.000221384 | 0.000238578 |
| 1029 | Synephrine | 94-07-5 | C04548 | C9H13NO2 | 167.094629 | Alkaloids | 168.1 | 150.1 | + | 2.627833333 | 6.19864E-05 | 5.4206E-05 | 0.000155208 | 9.04667E-05 | 9.67923E-05 | 0.000114246 | 0.000127345 | 0.000112794 | 0.000176548 | 0.000159332 | 0.00016794 |
| 1030 | Syringic acid | 530-57-4 | C10833 | C9H10O5 | 198.05282500000001 | Phenols | 199.1 | 140 | + | 3.5697 | 0.015516114 | 0.017156634 | 0.013513557 | 0.015395435 | 0.018295889 | 0.015425813 | 0.014989212 | 0.016236971 | 0.017389844 | 0.015105441 | 0.016247642 |
| 1031 | Syringin | 118-34-3 | C01533 | C17H24O9 | 372.14203500000002 | Phenylpropanoids | 373.1 | 193.1 | + | 4.614893658 | 0.00051908 | 0.000788434 | 0.000516012 | 0.000607842 | 0.000552825 | 0.000385422 | 0.000512494 | 0.00048358 | 0.000397013 | 0.000684241 | 0.000540627 |
| 1032 | Tabersonine | 4429-63-4 | C09244 | C21H24N2O2 | 336.18377800000002 | Alkaloids | 337.2 | 305.2 | + | 9.784683333 | 2.30003E-05 | 1.24145E-05 | 4.21558E-05 | 2.58569E-05 | 3.29104E-05 | 1.66488E-05 | 4.11134E-05 | 3.02242E-05 | 4.55102E-05 | 1.65627E-05 | 3.10365E-05 |
| 1033 | Tacrolimus | 104987-11-3 | C01375 | C44H69NO12 | 803.48197900000002 | Alkaloids | 786.5 | 768.5 | + | 12.39613993 | 0.001651145 | 0.001355429 | 0.001546419 | 0.001517664 | 0.006455089 | 0.002478267 | 0.002339871 | 0.003757742 | 0.006601645 | 0.002642104 | 0.004621874 |
| 1034 | Talatisamine | 20501-56-8 | C08713 | C24H39NO5 | 421.28282400000001 | Alkaloids | 422.3 | 404.3 | + | 4.662383333 | 0.002163623 | 0.002322377 | 0.002133142 | 0.002206381 | 0.002576324 | 0.000453204 | 0.002012652 | 0.001680727 | 0.002590886 | 0.002702172 | 0.002646529 |
| 1037 | Taurohyocholate |  | C15516 | C26H45NO7S | 515.29167500000005 |  | 516.3 | 109 | + | 11.4294 | 0.001804178 | 0.001882115 | 0.002107717 | 0.001931337 | 0.001528889 | 0.001562968 | 0.001655417 | 0.001582425 | 0.001485025 | 0.004241085 | 0.002863055 |
| 1038 | Taurolithocholate | 516-90-5 | C02592 | C26H45NO5S | 483.30184500000001 | Steroids and steroid derivatives | 484.3 | 109 | + | 13.779 | 0.000100068 | 0.00010376 | 4.21025E-05 | 8.19769E-05 | 5.4223E-05 | 5.12972E-05 | 0.000123233 | 7.62511E-05 | 8.23429E-05 | 0.000128816 | 0.00010558 |
| 1039 | Taxifolin | 480-18-2 | C01617 | C15H12O7 | 304.05829999999997 | flavonoids | 304.8 | 286.8 | + | 6.28 | 0.004562649 | 0.005630856 | 0.004486053 | 0.004893186 | 0.007230278 | 0.004107187 | 0.003788946 | 0.005042137 | 0.004962248 | 0.004443723 | 0.004702986 |
| 1040 | Taxiphyllin | 21401-21-8 | C01855 | C14H17NO7 | 311.100504 | Phenols | 312.1 | 132 | + | 13.76221667 | 0.001516261 | 0.001239073 | 0.001313664 | 0.001356333 | 0.001458206 | 0.001324689 | 0.001495514 | 0.001426136 | 0.00153902 | 0.001299504 | 0.001419262 |
| 1041 | Tectochrysin | 520-28-5 | C11621 | C16H12O4 | 268.07355999999999 | Flavonoids | 269.1 | 251.1 | + | 9.87735 | 8.75519E-05 | 6.51631E-05 | 5.38459E-05 | 6.88536E-05 | 9.58864E-05 | 9.83224E-05 | 0.000142518 | 0.000112242 | 5.96249E-05 | 0.000135222 | 9.74233E-05 |
| 1042 | Tectorigenin | 548-77-6 | C10534 | C16H12O6 | 300.0634 | flavonoids | 300.9 | 285.5 | + | 9.03 | 3.358E-05 | 3.67498E-05 | 4.60043E-05 | 3.87781E-05 | 0.000142685 | 0.000105893 | 5.44992E-05 | 0.000101026 | 7.06675E-05 | 5.10331E-05 | 6.08503E-05 |
| 1043 | Testosterone | 58-22-0 | C00535 | C19H28O2 | 288.20893000000001 | Steroids | 289.2 | 271.2 | + | 5.9835 | 0.000103634 | 0.000239096 | 0.000126815 | 0.000156515 | 0.000207137 | 0.000168883 | 0.00018618 | 0.0001874 | 0.000249296 | 0.00023599 | 0.000242643 |
| 1045 | Tetrahydrocurcumin | 36062-04-1 |  | C21H24O6 | 372.15728999999999 | Phenols | 373.2 | 137.1 | + | 9.381883333 | 0.000110601 | 0.000125917 | 9.40307E-05 | 0.000110183 | 8.82011E-05 | 0.000104575 | 5.60566E-05 | 8.29444E-05 | 0.000234518 | 0.000153706 | 0.000194112 |
| 1046 | Tetrahymanol | 2130-17-8 | C06083 | C31H54O | 442.41746499999999 | Triterpenoids | 443.4 | 425.4 | + | 10.57346667 | 4.84996E-05 | 8.99815E-05 | 2.92417E-05 | 5.59076E-05 | 5.04975E-05 | 6.49492E-05 | 5.52316E-05 | 5.68928E-05 | 2.50855E-05 | 7.01032E-05 | 4.75944E-05 |
| 1047 | Tetrandrine | 518-34-3 | C09654 | C38H42N2O6 | 622.30428800000004 | Alkaloids | 623.3 | 381.2 | + | 11.89931667 | 0.001248183 | 0.001367208 | 0.002219244 | 0.001611545 | 1.59752E-06 | 0.00073934 | 0.001703834 | 0.000814924 | 0.001129856 | 0.001773573 | 0.001451714 |
| 1048 | Thiamine | 59-43-8 | C00378 | C12H16N4OS | 264.10448200000002 | Vitamins | 265.1 | 122.1 | + | 0.700883333 | 0.000306106 | 0.000515283 | 0.000233261 | 0.00035155 | 0.000199686 | 0.000279843 | 0.000369802 | 0.00028311 | 0.000229922 | 0.000308113 | 0.000269018 |
| 1049 | Thromboxane A2 | 57576-52-0 | C02198 | C20H32O5 | 352.22497499999997 | Fatty Acyls | 353.2 | 71.1 | + | 11.4294 | 0.00388737 | 0.008382473 | 0.005118892 | 0.005796245 | 0.003926314 | 0.001983293 | 0.004078767 | 0.003329458 | 0.005026672 | 0.004391937 | 0.004709304 |
| 1050 | Thymidine | 50-89-5 | C00214 | C10H14N2O5 | 242.090273 | Nucleotide and its derivates | 243.1 | 127 | + | 3.0404191 | 0.000390557 | 0.000453093 | 0.000528325 | 0.000457325 | 0.000567747 | 0.000389261 | 0.000465261 | 0.00047409 | 0.000438836 | 0.000637766 | 0.000538301 |
| 1051 | Tombozine | 604-99-9 | C11635 | C19H22N2O | 294.17321299999998 | Alkaloids | 295.2 | 277.2 | + | 8.307783333 | 8.17595E-05 | 7.8991E-05 | 0.000102346 | 8.76987E-05 | 2.37524E-05 | 7.88095E-05 | 5.99347E-05 | 5.41656E-05 | 7.94235E-05 | 3.29914E-05 | 5.62075E-05 |
| 1052 | Torachrysone 8-O-glucoside | 64032-49-1 |  | C20H24O9 | 408.14203500000002 | Miscellaneous | 409.1 | 229.1 | + | 12.30035 | 0.001309652 | 0.001142451 | 0.000850659 | 0.00110092 | 0.001401901 | 0.000804212 | 0.000772569 | 0.000992894 | 0.000554781 | 0.00058685 | 0.000570816 |
| 1054 | Trachelanthamidine | 526-64-7 | C12440 | C8H15NO | 141.115364 | Alkaloids | 142.1 | 124.1 | + | 2.93725 | 3.3777E-05 | 3.00592E-05 | 4.0813E-05 | 3.4883E-05 | 4.58729E-05 | 4.17783E-05 | 3.72341E-05 | 4.16284E-05 | 5.83956E-05 | 4.71174E-05 | 5.27565E-05 |
| 1055 | Trachelogenin | 34209-69-3 | C10891 | C21H24O7 | 388.15220499999998 | Lignans | 389.2 | 137.1 | + | 5.00155 | 0.000100221 | 0.000151333 | 0.000110374 | 0.000120643 | 0.000187985 | 0.000220268 | 8.30352E-05 | 0.000163763 | 0.000137502 | 0.000116895 | 0.000127198 |
| 1056 | trans-3,5-Dimethoxy-4-hydroxy cinnamaldehydee | 4206-58-0 | C05610 | C11H12O4 | 208.07355999999999 | Phenylpropanoids | 209.1 | 55 | + | 5.169383333 | 0.001305226 | 0.000787306 | 0.001240674 | 0.001111069 | 0.001467229 | 0.001217726 | 0.001013796 | 0.001232917 | 0.001009298 | 0.001025943 | 0.00101762 |
| 1057 | Trans-caffeic acid | 501-16-6 | C01197 | C9H8O4 | 180.04226 | Phenylpropanoids | 181 | 121 | + | 3.306733333 | 0.000204854 | 0.000234948 | 0.000136371 | 0.000192057 | 0.000324993 | 0.000236688 | 0.000144764 | 0.000235481 | 0.000176798 | 0.000208836 | 0.000192817 |
| 1058 | trans-Caryophyllene | 87-44-5 | C09629 | C15H24 | 204.18780000000001 | Sesquiterpenoids | 205.2 | 95.1 | + | 12.1185 | 0.000625387 | 0.000418618 | 0.000396502 | 0.000480169 | 0.000587015 | 0.000398229 | 0.000345544 | 0.000443596 | 0.000717706 | 0.000287859 | 0.000502782 |
| 1059 | trans-Piceid | 27208-80-6 | C10275 | C21H24O8 | 404.14711999999997 | Stilbenes | 403.1 | 45 | - | 6.238010795 | 6.56003E-05 | 0.000230599 | 4.20862E-05 | 0.000112762 | 0.000345745 | 0.000291048 | 0.000295474 | 0.000310756 | 4.46901E-05 | 7.26224E-05 | 5.86562E-05 |
| 1060 | trans-Zeatin | 1637-39-4 | C00371 | C10H13N5O | 219.11199999999999 | phytohormone | 220 | 136 | + | 3.41 | 0.000160542 | 0.000203897 | 0.000436672 | 0.000267037 | 0.00013687 | 0.000125747 | 0.000175985 | 0.0001462 | 0.000112617 | 0.000225626 | 0.000169121 |
| 1061 | trans-Zeatin-riboside | 6025-53-2 | C16431 | C15H21N5O5 | 351.15429999999998 | phytohormone | 352 | 220 | + | 4.31 | 0.011764928 | 0.001318196 | 0.001223651 | 0.004768925 | 0.014140852 | 0.012734899 | 0.012819053 | 0.013231601 | 0.009932989 | 0.001622884 | 0.005777937 |
| 1063 | Trifolirhizin | 6807-83-6 | C10538 | C22H22O10 | 446.12130000000002 | Flavonoids | 447.1 | 267.1 | + | 8.211246 | 7.08689E-05 | 8.65238E-05 | 0.000103418 | 8.69368E-05 | 0.000162485 | 0.000248458 | 8.54454E-05 | 0.000165463 | 7.85836E-05 | 0.000149497 | 0.00011404 |
| 1064 | Trimethoprim | 738-70-5 | C01965 | C14H18N4O3 | 290.13789100000002 | Phenol ethers | 291.1 | 123.1 | + | 5.08325 | 7.42023E-05 | 0.000150962 | 9.13925E-05 | 0.000105519 | 0.000109976 | 0.000177461 | 0.000119769 | 0.000135735 | 3.15238E-05 | 0.000112761 | 7.21424E-05 |
| 1065 | Tripdiolide | 38647-10-8 | C09202 | C20H24O7 | 376.15220499999998 | Diterpenoids | 377.2 | 359.1 | + | 5.135816667 | 0.010467534 | 0.013838803 | 0.012250983 | 0.012185773 | 0.013918193 | 0.006245634 | 0.011729132 | 0.010630986 | 0.010782732 | 0.011272187 | 0.01102746 |
| 1066 | Tropacocaine | 537-26-8 | C10848 | C15H19NO2 | 245.14157900000001 | Alkaloids | 246.1 | 105 | + | 8.72735 | 0.000245905 | 0.000329822 | 0.000336562 | 0.000304096 | 0.000671836 | 0.000337122 | 0.000338258 | 0.000449072 | 0.000486297 | 0.000511172 | 0.000498734 |
| 1067 | Tropine acetate;3-Acetoxytropane | 3423-27-6;3423-26-5 | C12452;C12453 | C10H17NO2 | 183.12592900000001 | Alkaloids | 184.1 | 124.1 | + | 3.23935 | 0.002031129 | 0.003251642 | 0.00244645 | 0.002576407 | 0.003831198 | 0.002831773 | 0.003187812 | 0.003283594 | 0.002710469 | 0.00296273 | 0.002836599 |
| 1068 | Troxeruti | 7085-55-4 |  | C33H42O19 | 742.23202900000001 | flavonoids | 743.1 | 596.9 | + | 6.04 | 3.59954E-05 | 1.27649E-06 | 0.000106984 | 4.80854E-05 | 6.19134E-05 | 0.00012981 | 5.93413E-05 | 8.36883E-05 | 0.000163336 | 7.64289E-05 | 0.000119882 |
| 1069 | Tryptamine | 61-54-1 | C00398 | C10H12N2 | 160.10004799999999 | Tryptamine derivatives | 161.1 | 144.1 | + | 2.855683333 | 0.000182005 | 0.000291476 | 0.000172429 | 0.000215304 | 0.000309734 | 0.000245835 | 0.000110296 | 0.000221955 | 0.000228103 | 0.000170965 | 0.000199534 |
| 1070 | Tryptophan;L-Tryptophan;D-Tryptophan | 54-12-6;73-22-3;153-94-6 | C00806;C00078;C00525 | C11H12N2O2 | 204.089878;204.0899 | Alkaloids;Amino acid and derivatives;Indoles and derivatives;amino acids | 205.1 | 188.1 | + | 3.769372222 | 0.002197556 | 0.001532638 | 0.001934408 | 0.001888201 | 0.002213449 | 0.001643675 | 0.001591254 | 0.001816126 | 0.002067624 | 0.001616171 | 0.001841897 |
| 1071 | Turanose | 547-25-1 | C19636 | C12H22O11 | 342.11621500000001 | Fatty Acyls | 365.1 | 203.1 | + | 0.78905 | 0.005102065 | 0.005008031 | 0.005061938 | 0.005057345 | 0.017284764 | 0.013223925 | 0.011440045 | 0.013982911 | 0.012389093 | 0.008000565 | 0.010194829 |
| 1072 | Tussilagine | 80151-77-5 | C10411 | C10H17NO3 | 199.12084400000001 | Alkaloids | 200.1 | 182.1 | + | 3.8523 | 6.60371E-05 | 3.37299E-05 | 2.56081E-05 | 4.17917E-05 | 0.000121803 | 5.458E-05 | 6.95631E-05 | 8.19821E-05 | 7.2098E-05 | 5.7879E-05 | 6.49885E-05 |
| 1073 | Tyramine | 51-67-2 | C00483 | C8H11NO | 137.08406400000001 | Amino acid and derivatives | 138.1 | 121.1 | + | 3.256133333 | 4.81029E-05 | 6.50368E-05 | 8.97171E-05 | 6.7619E-05 | 7.88696E-05 | 6.81072E-05 | 5.20097E-05 | 6.63288E-05 | 3.94249E-05 | 0.000125257 | 8.23411E-05 |
| 1074 | Undecanolactone | 710-04-3 |  | C11H20O2 | 184.14633000000001 | Miscellaneous | 185.2 | 43.1 | + | 0.570866667 | 0.000730502 | 0.000362138 | 0.000984469 | 0.00069237 | 0.000862426 | 0.000744666 | 0.000875077 | 0.00082739 | 0.00102299 | 0.001141367 | 0.001082178 |
| 1075 | Uplandicine | 74202-10-1 | C10412 | C17H27NO7 | 357.17875400000003 | Alkaloids | 358.2 | 298.2 | + | 3.47205 | 1.02496E-05 | 5.47237E-05 | 2.25593E-05 | 2.91775E-05 | 5.31171E-05 | 6.87067E-05 | 8.54364E-05 | 6.90867E-05 | 1.20242E-05 | 3.3517E-05 | 2.27706E-05 |
| 1076 | Uracil | 66-22-8 | C00106 | C4H4N2O2 | 112.027278 | Nucleotide and its derivates | 113 | 70 | + | 1.9101 | 0.00564174 | 0.00780503 | 0.000105433 | 0.004517401 | 0.000231485 | 0.007013986 | 0.005007092 | 0.004084188 | 0.007783668 | 0.007428091 | 0.007605879 |
| 1077 | Uridine 5'-diphospho-D-glucose;UDP-D-galactose;Uridine diphosphategalactose | 133-89-1;2956-16-3 | C00029;C00052 | C15H24N2O17P2 | 566.055027 | Nucleotide and its derivates;Pyrimidine nucleotides | 565 | 323 | - | 0.709633333 | 0.00916378 | 0.005980865 | 0.003869596 | 0.006338081 | 0.009795426 | 0.015459661 | 0.005389843 | 0.010214977 | 0.007035708 | 0.007807698 | 0.007421703 |
| 1078 | Uridine 5'-monophosphate | 58-97-9 | C00105 | C9H13N2O9P | 324.03586999999999 | Nucleotide and its derivates | 323 | 79 | - | 1.392816667 | 0.000208872 | 8.85586E-05 | 0.000119921 | 0.000139117 | 0.000160826 | 0.000140745 | 0.000162389 | 0.000154653 | 0.00010701 | 0.000154672 | 0.000130841 |
| 1079 | Urocanic acid | 104-98-3 | C00785 | C6H6N2O2 | 138.04292799999999 | Azoles | 139.1 | 93 | + | 4.205033333 | 0.029598396 | 0.03101106 | 0.026055652 | 0.02888837 | 0.039597387 | 0.028314675 | 0.023363556 | 0.030425206 | 0.038126796 | 0.028272225 | 0.03319951 |
| 1080 | Ursodeoxycholic acid | 128-13-2 | C07880 | C24H40O4 | 392.29266000000001 | Steroids | 375.3 | 357.3 | + | 11.47265973 | 0.000763168 | 0.001133521 | 0.001125427 | 0.001007372 | 0.00092186 | 0.00080961 | 0.00099294 | 0.000908137 | 0.001005996 | 0.000747828 | 0.000876912 |
| 1081 | Valechlorine | 51771-49-4 |  | C22H31ClO8 | 458.170748 | Iridoids | 459.2 | 85.1 | + | 0.625483333 | 0.000853011 | 0.000754757 | 0.000824652 | 0.000810807 | 0.001089827 | 0.000795052 | 0.000563505 | 0.000816128 | 0.000791794 | 0.000904163 | 0.000847978 |
| 1082 | Valerenic acid | 3569-10-6 | C09743 | C15H22O2 | 234.16198 | Sesquiterpenoids | 235.2 | 161.1 | + | 11.24478333 | 0.000242607 | 8.39105E-05 | 0.000688044 | 0.000338187 | 0.001098238 | 1.26522E-06 | 0.000939303 | 0.000679602 | 0.00137754 | 0.000793645 | 0.001085593 |
| 1083 | Vanillic acid | 121-34-6 | C06672 | C8H8O4 | 168.04226 | Phenols | 169 | 65 | + | 3.7764 | 0.000451121 | 0.000501761 | 0.000346714 | 0.000433199 | 0.000341998 | 0.000332381 | 0.000424293 | 0.000366224 | 0.000279917 | 0.00029347 | 0.000286694 |
| 1084 | Vanillin | 121-33-5 | C00755 | C8H8O3 | 152.04734500000001 | Phenols | 153.1 | 65 | + | 5.895917043 | 0.001056137 | 0.000907738 | 0.000922577 | 0.000962151 | 0.001048379 | 0.000848355 | 0.001809484 | 0.001235406 | 0.001023645 | 0.000909846 | 0.000966745 |
| 1085 | Vasicine | 6159-55-3 | C10733 | C11H12N2O | 188.09496300000001 | Alkaloids | 189.1 | 171.1 | + | 3.759616667 | 0.002116324 | 0.002018923 | 0.001823647 | 0.001986298 | 0.002899199 | 0.002137176 | 0.001905119 | 0.002313831 | 0.002124731 | 0.002277236 | 0.002200983 |
| 1086 | Vasicinone | 486-64-6 | C10744 | C11H10N2O2 | 202.07422800000001 | Alkaloids | 203.1 | 185.1 | + | 8.72735 | 0.000123528 | 9.99633E-05 | 6.20998E-05 | 9.5197E-05 | 0.000293952 | 0.000703337 | 2.81325E-05 | 0.000341807 | 0.000304287 | 7.20468E-05 | 0.000188167 |
| 1087 | Veraguensin | 19950-55-1 | C10892 | C22H28O5 | 372.19367499999998 | Lignans | 373.2 | 137.1 | + | 6.746966667 | 0.000581735 | 0.000363108 | 0.000539632 | 0.000494825 | 0.002005056 | 0.00118223 | 0.001831216 | 0.001672834 | 0.001563366 | 0.000308406 | 0.000935886 |
| 1088 | Vestitol | 35878-41-2 | C16225 | C16H16O4 | 272.10485999999997 | Flavonoids | 273.1 | 123 | + | 6.512016667 | 0.000200215 | 0.000240346 | 0.000230203 | 0.000223588 | 8.29828E-05 | 8.22566E-05 | 7.60218E-05 | 8.04204E-05 | 0.000260449 | 0.000105787 | 0.000183118 |
| 1089 | Vicenin 2 | 23666-13-9 | C10195 | C27H30O15 | 594.15847499999995 | Flavonoids | 595.2 | 577.2 | + | 4.984766667 | 0.000134786 | 0.00011253 | 0.000110824 | 0.00011938 | 0.000160538 | 3.45773E-05 | 0.000158012 | 0.000117709 | 0.000148369 | 0.000100332 | 0.000124351 |
| 1090 | Vidarabine | 5536-17-4;24356-66-9 | C07195 | C10H13N5O4 | 267.09675499999997 | Purine nucleosides | 268.1 | 136.1 | + | 2.2804 | 0.39464774 | 0.650095444 | 0.606274604 | 0.550339263 | 0.588246345 | 0.493878738 | 0.358366011 | 0.480163698 | 1.011476326 | 0.476774722 | 0.744125524 |
| 1091 | Vinblastine | 865-21-4 | C07201 | C46H58N4O9 | 810.42038100000002 | Alkaloids | 811.4 | 355.2 | + | 11.62253333 | 0.000166064 | 0.000201339 | 1.12648E-06 | 0.000122843 | 0.000182874 | 8.37458E-05 | 0.000219615 | 0.000162078 | 0.000210261 | 0.000129621 | 0.000169941 |
| 1092 | Vitamin A | 68-26-8;11103-57-4 | C00473 | C20H30O | 286.22966500000001 | Vitamins | 269.2 | 93.1 | + | 9.86265 | 0.000591611 | 0.000442044 | 0.000674103 | 0.000569252 | 0.000629452 | 0.000641575 | 0.000316395 | 0.000529141 | 0.000474001 | 0.000532458 | 0.000503229 |
| 1093 | Vitamin A2 aldehyde | 472-87-7 | C05918 | C20H26O | 282.19836500000002 | Prenol lipids | 283.2 | 173.1 | + | 11.24781667 | 0.000171132 | 0.000233856 | 6.98468E-05 | 0.000158278 | 0.000109708 | 0.00015364 | 7.93003E-05 | 0.000114216 | 0.000207359 | 9.62189E-05 | 0.000151789 |
| 1095 | vitamin K2 | 11032-49-8 |  | C41H56O2 | 580.42803000000004 | Vitamins | 581.4 | 563.4 | + | 5.056016667 | 9.48599E-05 | 8.6105E-06 | 3.03703E-05 | 4.46136E-05 | 8.05803E-05 | 0.000130119 | 7.49207E-05 | 9.52065E-05 | 5.45596E-05 | 0.000102235 | 7.83975E-05 |
| 1096 | Vitexin | 3681-93-4 | C01460 | C21H20O10 | 432.10559999999998 | flavonoids | 433.4 | 414.9 | + | 5.92 | 0.00017377 | 0.000149406 | 0.00011421 | 0.000145795 | 0.000595441 | 0.000172762 | 0.000510653 | 0.000426285 | 0.000171824 | 0.000766657 | 0.00046924 |
| 1097 | Vitexin 2''-glucoside | 61360-94-9 | C04024 | C27H30O15 | 594.15847499999995 | Flavonoids | 595.2 | 415.1 | + | 4.910366667 | 0.000152942 | 0.000226527 | 0.000206226 | 0.000195232 | 0.000344933 | 9.84881E-05 | 5.76405E-05 | 0.000167021 | 0.000144601 | 0.000111526 | 0.000128063 |
| 1098 | Vitexin-2''-O-rhamnoside;Vitexin 2''-O-beta-L-rhamnoside | 64820-99-1 | C12628 | C27H30O14 | 578.16355999999996 | Flavonoids | 579.2 | 415.1 | + | 12.11373333 | 0.000563588 | 0.000446667 | 0.000342432 | 0.000450896 | 0.000652866 | 0.000193991 | 0.000249232 | 0.000365363 | 0.000379919 | 0.00035744 | 0.000368679 |
| 1099 | Vomicine | 125-15-5 | C09255 | C22H24N2O4 | 380.173608 | Alkaloids | 381.2 | 363.2 | + | 5.370783333 | 5.24609E-05 | 0.000104859 | 8.49173E-05 | 8.07459E-05 | 7.6797E-05 | 0.000121789 | 6.34516E-05 | 8.73459E-05 | 6.83395E-05 | 0.000147166 | 0.000107753 |
| 1100 | Wighteone | 51225-30-0 | C10542 | C20H18O5 | 338.11542500000002 | Flavonoids | 339.1 | 55.1 | + | 13.00698333 | 0.003840896 | 0.044033176 | 0.066556131 | 0.038143401 | 0.051506924 | 0.041018186 | 0.033375855 | 0.041966988 | 0.048564881 | 0.047428112 | 0.047996496 |
| 1101 | Withanolide A | 32911-62-9 |  | C28H38O6 | 470.26684 | Steroids and steroid derivatives | 471.3 | 453.3 | + | 12.26855 | 0.000487472 | 0.000735679 | 0.000290798 | 0.00050465 | 0.00034966 | 0.000502155 | 0.000283487 | 0.000378434 | 0.000589563 | 0.000418591 | 0.000504077 |
| 1102 | Xanthohumol | 6754-58-1 | C16417 | C21H22O5 | 354.146725 | Flavanone | 353.1 | 119.1 | - | 10.9575 | 3.07474E-05 | 5.50272E-05 | 4.46276E-05 | 4.34674E-05 | 4.09206E-05 | 2.6449E-05 | 4.78152E-05 | 3.83949E-05 | 1.55348E-05 | 4.41408E-05 | 2.98378E-05 |
| 1103 | Xanthosine | 146-80-5 | C01762 | C10H12N4O6 | 284.07568600000002 | Nucleotide and its derivates | 285.1 | 153 | + | 2.610383333 | 0.001893995 | 0.001612712 | 0.001376109 | 0.001627606 | 0.00189565 | 0.001435417 | 0.001500725 | 0.001610597 | 0.002130026 | 0.001912025 | 0.002021025 |
| 1104 | Xanthotoxol | 2009-24-7 | C00841 | C11H6O4 | 202.02661000000001 | Phenylpropanoids | 203 | 175 | + | 8.742883333 | 0.000488482 | 0.000767735 | 0.000833802 | 0.000696673 | 0.003926852 | 0.003405914 | 0.003076513 | 0.00346976 | 0.001524717 | 0.000943798 | 0.001234257 |
| 1105 | Yatein | 40456-50-6 | C10557 | C22H24O7 | 400.15220499999998 | Lignans | 401.2 | 161.1 | + | 12.6042 | 0.001189484 | 0.00038841 | 0.000755603 | 0.000777833 | 0.001316714 | 0.000637025 | 0.000699094 | 0.000884278 | 0.000893661 | 0.000485493 | 0.000689577 |
| 1106 | Yohimbic acid monohydrate | 522-87-2 |  | C20H24N2O3 | 340.17869300000001 | Alkaloids | 341.2 | 144.1 | + | 13.74883333 | 0.000172979 | 0.000459976 | 0.000186641 | 0.000273199 | 0.000394557 | 0.000216168 | 0.000336334 | 0.000315686 | 0.000405609 | 0.00046176 | 0.000433685 |
| 1107 | Zeorin;Dammarenediol II | 22570-53-2;14351-29-2 | C19829 | C30H52O2 | 444.39672999999999 | Triterpenoids | 445.4 | 409.4 | + | 12.61713333 | 0.000112455 | 8.34293E-05 | 0.000190477 | 0.000128787 | 9.50573E-05 | 7.37295E-05 | 0.000129522 | 9.94361E-05 | 0.000123471 | 0.00012813 | 0.000125801 |
| 1108 | Zeranol | 26538-44-3 | C14752 | C18H26O5 | 322.17802499999999 | Macrolides and analogues | 323.2 | 305.2 | + | 11.84896667 | 1.78749E-05 | 1.27649E-06 | 0.00010381 | 4.09872E-05 | 1.59752E-06 | 0.000152733 | 0.000117167 | 9.04993E-05 | 0.0001887 | 0.000226767 | 0.000207733 |
| 1109 | Zingerone | 122-48-5 | C17497 | C11H14O3 | 194.09429499999999 | Phenols | 195.1 | 137.1 | + | 4.598766667 | 0.000146231 | 9.27643E-05 | 0.000208082 | 0.000149026 | 8.13179E-05 | 0.000137774 | 0.000103291 | 0.000107461 | 0.000106552 | 8.78406E-05 | 9.71964E-05 |
|  |  |  |  |  |  |  |  |  |  |  |  |  |  |  |  |  |  |  |  |  |  |

**Table S2 KEGG pathway enrichment of the differentially expressed metabolites**

| **Pathway** | **Total** | **Hits** | **Raw p** | **-ln(p)** | **Holm adjust** | **FDR** | **Impact** | **Hits Cpd** | **Total Cpd** |
| --- | --- | --- | --- | --- | --- | --- | --- | --- | --- |
| Valine, leucine and isoleucine biosynthesis | 26 | 3 | 0.076399 | 2.5718 | 1 | 1 | 0.01865 | L-Threonine cpd:C00188; L-Leucine cpd:C00123; L-Isoleucine cpd:C00407 | (R)-2-Methylmalate cpd:C02612; Citraconic acid cpd:C02226; D-erythro-3-Methylmalate cpd:C06032; L-Threonine cpd:C00188; 3-Methyl-2-oxovaleric acid cpd:C00671; L-Leucine cpd:C00123; (R) 2,3-Dihydroxy-3-methylvalerate cpd:C06007; 2-Isopropylmalic acid cpd:C02504; 3-Isopropylmalate cpd:C04411; Alpha-ketoisovaleric acid cpd:C00141; L-Valine cpd:C00183; (R)-2,3-Dihydroxy-isovalerate cpd:C04272; (S)-2-Aceto-2-hydroxybutanoic acid cpd:C06006; (S)-2-Acetolactate cpd:C06010; 2-(a-Hydroxyethyl)thiamine diphosphate cpd:C05125; L-Isoleucine cpd:C00407; Pyruvic acid cpd:C00022; 2-Ketobutyric acid cpd:C00109; L-Leucyl-tRNA cpd:C02047; 4-Methyl-2-oxopentanoate cpd:C00233; (R)-3-Hydroxy-3-methyl-2-oxopentanoate cpd:C14463; Isopropylmaleate cpd:C02631; 2-Isopropyl-3-oxosuccinate cpd:C04236; L-Valyl-tRNA(Val) cpd:C02554; 3-Hydroxy-3-methyl-2-oxobutanoic acid cpd:C04181; L-Isoleucyl-tRNA(Ile) cpd:C03127 |
| Monoterpenoid biosynthesis | 4 | 1 | 0.14663 | 1.9198 | 1 | 1 | 0.5 | (-)-alpha-Terpineol cpd:C11393 | Geranyl-PP cpd:C00341; (-)-alpha-Terpineol cpd:C11393; Myrcene cpd:C06074; Eucalyptol cpd:C09844 |
| Tyrosine metabolism | 18 | 2 | 0.15237 | 1.8815 | 1 | 1 | 0 | Gentisate aldehyde cpd:C05585; Hordenine cpd:C06199 | 3,4-Dihydroxyphenylglycol cpd:C05576; L-Dopa cpd:C00355; Homogentisic acid cpd:C00544; 4-Fumarylacetoacetic acid cpd:C01061; L-Tyrosine cpd:C00082; 4-Hydroxyphenylpyruvic acid cpd:C01179; Succinic acid semialdehyde cpd:C00232; Tyramine cpd:C00483; Dopamine cpd:C03758; N-Methyltyramine cpd:C02442; 3,4-Dihydroxymandelaldehyde cpd:C05577; Gentisate aldehyde cpd:C05585; Fumaric acid cpd:C00122; Acetoacetic acid cpd:C00164; Succinic acid cpd:C00042; 4-Hydroxyphenylacetaldehyde cpd:C03765; 3,4-Dihydroxyphenylacetaldehyde cpd:C04043; Hordenine cpd:C06199 |
| Aminoacyl-tRNA biosynthesis | 67 | 4 | 0.25976 | 1.348 | 1 | 1 | 0 | L-Asparagine cpd:C00152; L-Isoleucine cpd:C00407; L-Leucine cpd:C00123; L-Threonine cpd:C00188 | L-Asparagine cpd:C00152; tRNA(Asn) cpd:C01637; L-Histidine cpd:C00135; tRNA(His) cpd:C01643; L-Phenylalanine cpd:C00079; tRNA(Phe) cpd:C01648; L-Arginine cpd:C00062; tRNA(Arg) cpd:C01636; L-Glutamine cpd:C00064; tRNA(Gln) cpd:C01640; L-Cysteine cpd:C00097; tRNA(Cys) cpd:C01639; Glycine cpd:C00037; tRNA(Gly) cpd:C01642; tRNA(Asp) cpd:C01638; L-Aspartic acid cpd:C00049; L-Serine cpd:C00065; tRNA(Ser) cpd:C01650; L-Methionine cpd:C00073; tRNA(Met) cpd:C01647; L-Valine cpd:C00183; tRNA(Val) cpd:C01653; L-Alanine cpd:C00041; tRNA(Ala) cpd:C01635; L-Lysine cpd:C00047; tRNA(Lys) cpd:C01646; L-Isoleucine cpd:C00407; tRNA(Ile) cpd:C01644; tRNA(Leu) cpd:C01645; L-Leucine cpd:C00123; L-Threonine cpd:C00188; tRNA(Thr) cpd:C01651; tRNA(Trp) cpd:C01652; L-Tryptophan cpd:C00078; N10-Formyl-THF cpd:C00234; L-Methionyl-tRNA cpd:C02430; L-Tyrosine cpd:C00082; tRNA(Tyr) cpd:C00787; L-Proline cpd:C00148; tRNA(Pro) cpd:C01649; L-Glutamic acid cpd:C00025; tRNA(Glu) cpd:C01641; Glutaminyl-tRNA cpd:C02282; L-Asparaginyl-tRNA(Asn) cpd:C03402; tRNA(Sec) cpd:C16636; L-Histidyl-tRNA(His) cpd:C02988; L-Phenylalanyl-tRNA(Phe) cpd:C03511; L-Arginyl-tRNA(Arg) cpd:C02163; L-Cysteinyl-tRNA(Cys) cpd:C03125; Glycyl-tRNA(Gly) cpd:C02412; L-Aspartyl-tRNA(Asp) cpd:C02984; L-Seryl-tRNA(Ser) cpd:C02553; L-Valyl-tRNA(Val) cpd:C02554; L-Alanyl-tRNA cpd:C00886; L-Lysyl-tRNA cpd:C01931; L-Isoleucyl-tRNA(Ile) cpd:C03127; L-Leucyl-tRNA cpd:C02047; L-Threonyl-tRNA(Thr) cpd:C02992; L-Tryptophanyl-tRNA(Trp) cpd:C03512; Tetrahydrofolic acid cpd:C00101; N-Formylmethionyl-tRNA cpd:C03294; L-Tyrosyl-tRNA(Tyr) cpd:C02839; L-Prolyl-tRNA(Pro) cpd:C02702; L-Glutamyl-tRNA(Glu) cpd:C02987; L-Glutamyl-tRNA(Gln) cpd:C06112; L-Aspartyl-tRNA(Asn) cpd:C06113; L-Seryl-tRNA(Sec) cpd:C06481 |
| Valine, leucine and isoleucine degradation | 34 | 2 | 0.38377 | 0.95772 | 1 | 1 | 0 | L-Isoleucine cpd:C00407; L-Leucine cpd:C00123 | Enzyme N6-(lipoyl)lysine cpd:C15972; 2-Methyl-1-hydroxybutyl-ThPP cpd:C15978; Enzyme N6-(dihydrolipoyl)lysine cpd:C15973; 2-Methyl-1-hydroxypropyl-ThPP cpd:C15976; 3-Methyl-1-hydroxybutyl-ThPP cpd:C15974; Acetyl-CoA cpd:C00024; Acetoacetyl-CoA cpd:C00332; 3-Hydroxy-3-methylglutaryl-CoA cpd:C00356; 3-Methylcrotonyl-CoA cpd:C03069; 3-Hydroxyisovaleryl-CoA cpd:C05998; Isovaleryl-CoA cpd:C02939; 3-Methyl-2-oxovaleric acid cpd:C00671; Thiamine pyrophosphate cpd:C00068; L-Valine cpd:C00183; 2-Methylacetoacetyl-CoA cpd:C03344; Tiglyl-CoA cpd:C03345; S-(2-Methylbutanoyl)-dihydrolipoamide cpd:C15979; Alpha-ketoisovaleric acid cpd:C00141; L-Isoleucine cpd:C00407; (S)-Methylmalonic acid semialdehyde cpd:C06002; (S)-3-Hydroxyisobutyrate cpd:C06001; Methacrylyl-CoA cpd:C03460; Butyryl-CoA cpd:C00630; S-(2-Methylpropionyl)-dihydrolipoamide-E cpd:C15977; 4-Methyl-2-oxopentanoate cpd:C00233; S-(3-Methylbutanoyl)-dihydrolipoamide-E cpd:C15975; L-Leucine cpd:C00123; (S)-2-Methylbutanoyl-CoA cpd:C15980; Acetoacetic acid cpd:C00164; 3-Methylglutaconyl-CoA cpd:C03231; Propionyl-CoA cpd:C00100; 2-Methyl-3-hydroxybutyryl-CoA cpd:C04405; Methylmalonic acid cpd:C02170; (S)-3-Hydroxyisobutyryl-CoA cpd:C06000 |
| Glyoxylate and dicarboxylate metabolism | 17 | 1 | 0.49206 | 0.70916 | 1 | 1 | 0.09524 | cis-Aconitic acid cpd:C00417 | cis-Aconitic acid cpd:C00417; Isocitric acid cpd:C00311; Formamide cpd:C00488; N10-Formyl-THF cpd:C00234; Glycolic acid cpd:C00160; Formic acid cpd:C00058; Citric acid cpd:C00158; L-Malic acid cpd:C00149; D-Ribulose 1,5-bisphosphate cpd:C01182; Glyoxylic acid cpd:C00048; Succinic acid cpd:C00042; Carbon dioxide cpd:C00011; H+ cpd:C00080; Acetyl-CoA cpd:C00024; Oxalacetic acid cpd:C00036; 3-Phospho-D-glycerate cpd:C00197; Phosphoglycolic acid cpd:C00988 |
| Flavonoid biosynthesis | 43 | 2 | 0.50476 | 0.68368 | 1 | 1 | 0.13022 | Cyanidin cpd:C05905; Naringenin chalcone cpd:C06561 | Pinobanksin cpd:C09826; p-Coumaroyl quinic acid cpd:C12208; 4-Coumaroylshikimate cpd:C02947; Kaempferol cpd:C05903; Delphinidin cpd:C05908; Cyanidin cpd:C05905; Pelargonidin cpd:C05904; Leucodelphinidin cpd:C05909; Leucocyanidin cpd:C05906; cis-3,4-Leucopelargonidin cpd:C03648; Garbanzol cpd:C09751; Dihydrokaempferol cpd:C00974; Naringenin cpd:C00509; Liquiritigenin cpd:C09762; Pentahydroxyflavanone cpd:C05911; Eriodictyol cpd:C05631; Butin cpd:C09614; Pinocembrin cpd:C09827; p-Coumaroyl-CoA cpd:C00223; Dihydromyricetin cpd:C02906; Caffeoyl-CoA cpd:C00323; Naringenin chalcone cpd:C06561; Feruloyl-CoA cpd:C00406; Apigenin cpd:C01477; Pinocembrin chalcone cpd:C16404; Butein cpd:C08578; Cinnamoyl-CoA cpd:C00540; Isoliquiritigenin cpd:C08650; Taxifolin cpd:C01617; Galangin cpd:C10044; Chlorogenic acid cpd:C00852; 5-O-Caffeoylshikimic acid cpd:C10434; Quercetin cpd:C00389; (-)-Epigallocatechin cpd:C12136; Epicatechin cpd:C09727; (-)-Epiafzelechin cpd:C12128; Fustin cpd:C01378; Myricetin cpd:C10107; Luteoforol cpd:C05907; Eriodictyol chalcone cpd:C15525; Apiforol cpd:C12124; Homoeriodictyol chalcone cpd:C16405; Luteolin cpd:C01514 |
| Phenylpropanoid biosynthesis | 45 | 2 | 0.5296 | 0.63563 | 1 | 1 | 0.06385 | Ferulate cpd:C01494; Sinapyl alcohol cpd:C02325 | 4-Hydroxycinnamyl aldehyde cpd:C05608; p-Coumaroyl-CoA cpd:C00223; 4-Hydroxycinnamic acid cpd:C00811; Sinapoyl aldehyde cpd:C05610; Coniferyl aldehyde cpd:C02666; Coniferyl alcohol cpd:C00590; Ferulate cpd:C01494; p-Coumaroyl quinic acid cpd:C12208; 4-Coumaroylshikimate cpd:C02947; cis-beta-D-Glucosyl-2-hydroxycinnamate cpd:C05839; L-Phenylalanine cpd:C00079; 5-Hydroxyferuloyl-CoA cpd:C12203; Sinapate cpd:C00482; 5-Hydroxyferulate cpd:C05619; 3,4-Dihydroxy-trans-cinnamate cpd:C01197; Caffeoyl-CoA cpd:C00323; Sinapoyl-CoA cpd:C00411; Feruloyl-CoA cpd:C00406; 5-Hydroxyconiferaldehyde cpd:C12204; Sinapyl alcohol cpd:C02325; 5-Hydroxyconiferyl alcohol cpd:C12205; 4-Coumaryl alcohol cpd:C02646; trans-Cinnamic acid cpd:C00423; Cinnamoyl-CoA cpd:C00540; 1-O-Sinapoyl-beta-D-glucose cpd:C01175; Caffeic aldehyde cpd:C10945; N1,N5,N10-Tricoumaroyl spermidine cpd:C18069; N1,N5,N10-Triferuloyl spermidine cpd:C18071; N1,N5,N10-Tri-(hydroxyferuloyl)-spermidine cpd:C18072; Chlorogenic acid cpd:C00852; 5-O-Caffeoylshikimic acid cpd:C10434; cis-2-Hydroxycinnamate cpd:C05838; Syringyl lignin cpd:C15806; 5-Hydroxy-guaiacyl lignin cpd:C15807; Guaiacyl lignin cpd:C15805; Syringin cpd:C01533; Coniferin cpd:C00761; 4-Hydroxycinnamyl alcohol 4-D-glucoside cpd:C05855; Cinnamaldehyde cpd:C00903; Sinapoyl malate cpd:C02887; Caffeyl alcohol cpd:C12206; p-Hydroxyphenyl lignin cpd:C15804; Sinapine cpd:C00933; N1,N5,N10-Tricaffeoyl spermidine cpd:C18070; N1,N5-Tri-di(hydroxyferuloyl)-N10-sinapoyl-spermidine cpd:C18073 |
| Citrate cycle (TCA cycle) | 20 | 1 | 0.54972 | 0.59834 | 1 | 1 | 0.04728 | cis-Aconitic acid cpd:C00417 | Enzyme N6-(dihydrolipoyl)lysine cpd:C15973; Oxoglutaric acid cpd:C00026; Thiamine pyrophosphate cpd:C00068; Enzyme N6-(lipoyl)lysine cpd:C15972; 3-carboxy-1-hydroxypropylthiamine diphosphate cpd:C05381; Succinyl-CoA cpd:C00091; Succinic acid cpd:C00042; Oxalosuccinic acid cpd:C05379; Isocitric acid cpd:C00311; Oxalacetic acid cpd:C00036; Acetyl-CoA cpd:C00024; L-Malic acid cpd:C00149; cis-Aconitic acid cpd:C00417; Citric acid cpd:C00158; Pyruvic acid cpd:C00022; 2-(a-Hydroxyethyl)thiamine diphosphate cpd:C05125; [Dihydrolipoyllysine-residue succinyltransferase] S-succinyldihydrolipoyllysine cpd:C16254; Fumaric acid cpd:C00122; S-Acetyldihydrolipoamide-E cpd:C16255; Phosphoenolpyruvic acid cpd:C00074 |
| Alanine, aspartate and glutamate metabolism | 22 | 1 | 0.58455 | 0.53692 | 1 | 1 | 0 | L-Asparagine cpd:C00152 | 2-Oxosuccinamate cpd:C02362; L-Aspartic acid cpd:C00049; Argininosuccinic acid cpd:C03406; Adenylsuccinic acid cpd:C03794; L-Alanine cpd:C00041; Succinic acid semialdehyde cpd:C00232; Oxoglutaric acid cpd:C00026; L-Glutamine cpd:C00064; L-Glutamic acid cpd:C00025; 2-Keto-glutaramic acid cpd:C00940; (S)-1-Pyrroline-5-carboxylate cpd:C03912; Oxalacetic acid cpd:C00036; L-Asparagine cpd:C00152; Fumaric acid cpd:C00122; Pyruvic acid cpd:C00022; Ureidosuccinic acid cpd:C00438; Succinic acid cpd:C00042; Ammonia cpd:C00014; Gamma-Aminobutyric acid cpd:C00334; Carbamoylphosphate cpd:C00169; Glucosamine 6-phosphate cpd:C00352; 5-Phosphoribosylamine cpd:C03090 |
| alpha-Linolenic acid metabolism | 23 | 1 | 0.60095 | 0.50924 | 1 | 1 | 0.15 | 9(S)-HPOT cpd:C16321 | 13(S)-HPOT cpd:C04785; OPC4-CoA cpd:C16335; OPC6-CoA cpd:C16331; 3-Oxo-OPC4-CoA cpd:C16338; 3-Oxo-OPC6-CoA cpd:C16334; Alpha-Linolenic acid cpd:C06427; 12-OPDA cpd:C01226; (-)-Jasmonic acid cpd:C08491; 3-Oxo-OPC8-CoA cpd:C16330; OPC8-CoA cpd:C16327; (9Z,15Z)-(13S)-12,13-Epoxyoctadeca-9,11,15-trienoic acid cpd:C04672; 9(S)-HPOT cpd:C16321; 8-[(1R,2R)-3-Oxo-2-{(Z)-pent-2-enyl}cyclopentyl]octanoate cpd:C04780; 12-Oxo-9(Z)-dodecenoic acid cpd:C16311; 3-Hexenal cpd:C16310; trans-2-Enoyl-OPC4-CoA cpd:C16336; trans-2-Enoyl-OPC6-CoA cpd:C16332; (+)-7-Isojasmonic acid CoA cpd:C16339; Methyl jasmonate cpd:C11512; trans-2-Enoyl-OPC8-CoA cpd:C16328; 9,10-EOT cpd:C16324; 9-Oxononanoic acid cpd:C16322; 3,6-Nonadienal cpd:C16323 |
| Glucosinolate biosynthesis | 54 | 2 | 0.63096 | 0.46051 | 1 | 1 | 0 | L-Leucine cpd:C00123; L-Isoleucine cpd:C00407 | L-Methionine cpd:C00073; Homomethionine cpd:C17213; S-(4-Methylthiobutylthiohydroximoyl)-L-cysteine cpd:C17242; 3-Methylthiopropyl-desulfoglucosinolate cpd:C17244; 2-Oxo-5-methylthiopentanoic acid cpd:C17211; 4-Methylthiobutanaldoxime cpd:C17241; 4-Methylthiobutylthiohydroximate cpd:C17243; 2-Oxo-4-methylthiobutanoic acid cpd:C01180; 2-Oxo-6-methylthiohexanoic acid cpd:C17216; 2-Oxo-7-methylthioheptanoic acid cpd:C17220; 2-Oxo-8-methylthiooctanoic acid cpd:C17224; 2-Oxo-9-methylthiononanoic acid cpd:C17228; 2-Oxo-10-methylthiodecanoic acid cpd:C17232; Dihomomethionine cpd:C17217; 4-Methylthiobutyl-desulfoglucosinolate cpd:C17248; Trihomomethionine cpd:C17221; Tetrahomomethionine cpd:C17225; Pentahomomethionine cpd:C17229; Hexahomomethionine cpd:C17233; L-Phenylalanine cpd:C00079; S-(Phenylacetothiohydroximoyl)-L-cysteine cpd:C17237; (Z)-Phenylacetaldehyde oxime cpd:C16075; Phenylacetothiohydroximate cpd:C03719; L-Tryptophan cpd:C00078; S-(Indolylmethylthiohydroximoyl)-L-cysteine cpd:C16518; Indolylmethyl-desulfoglucosinolate cpd:C16517; Indole-3-acetaldehyde oxime cpd:C02937; Indolylmethylthiohydroximate cpd:C16516; S-(Hydroxyphenylacetothiohydroximoyl)-L-cysteine cpd:C17238; p-Hydroxybenzyldesulphoglucosinolate cpd:C17240; (Z)-4-Hydroxyphenylacetaldehyde-oxime cpd:C04353; L-Valine cpd:C00183; L-Leucine cpd:C00123; L-Isoleucine cpd:C00407; Glucoiberverin cpd:C08412; 2-(2'-Methylthio)ethylmalic acid cpd:C17210; 2-(3'-Methylthio)propylmalic acid cpd:C17214; 2-(4'-Methylthio)butylmalic acid cpd:C17218; 2-(5'-Methylthio)pentylmalic acid cpd:C17222; 2-(6'-Methylthio)hexylmalic acid cpd:C17226; 2-(7'-Methylthio)heptylmalic acid cpd:C17230; 5-Methylthiopentanaldoxime cpd:C17245; Glucoerucin cpd:C08409; 6-Methylthiohexanaldoxime cpd:C17246; 7-Methylthioheptanaldoxime cpd:C17249; 8-Methylthiooctanaldoxime cpd:C17251; 9-Methylthiononanaldoxime cpd:C17253; Desulfoglucotropeolin cpd:C01069; Glucobrassicin cpd:C05837; p-Hydroxyphenylacetothiohydroximate cpd:C17239; Sinalbin cpd:C08426; Alpha-ketoisovaleric acid cpd:C00141; 4-Methyl-2-oxopentanoate cpd:C00233; 3-Methyl-2-oxovaleric acid cpd:C00671 |
| Tryptophan metabolism | 27 | 1 | 0.66047 | 0.41481 | 1 | 1 | 0 | N-Hydroxyl-tryptamine cpd:C17203 | Indoleacrylic acid cpd:C00331; 5-Hydroxykynurenine cpd:C05651; 5-Hydroxy-L-tryptophan cpd:C00643; Acetoacetyl-CoA cpd:C00332; Crotonoyl-CoA cpd:C00877; Oxoadipic acid cpd:C00322; L-Tryptophan cpd:C00078; 3-Hydroxyanthranilic acid cpd:C00632; Indoleacetaldehyde cpd:C00637; 5-Hydroxyindoleacetaldehyde cpd:C05634; 3-Indoleacetonitrile cpd:C02938; Glucobrassicin cpd:C05837; Tryptamine cpd:C00398; Indole-3-acetaldehyde oxime cpd:C02937; S-(Indolylmethylthiohydroximoyl)-L-cysteine cpd:C16518; Indolylmethyl-desulfoglucosinolate cpd:C16517; Indolylmethylthiohydroximate cpd:C16516; 5-Hydroxykynurenamine cpd:C05638; Serotonin cpd:C00780; Acetyl-CoA cpd:C00024; (S)-3-Hydroxybutanoyl-CoA cpd:C01144; Glutaryl-CoA cpd:C00527; L-Tryptophanyl-tRNA(Trp) cpd:C03512; Cinnavalininate cpd:C05640; Indoleacetic acid cpd:C00954; 5-Hydroxyindoleacetic acid cpd:C05635; N-Hydroxyl-tryptamine cpd:C17203 |
| Starch and sucrose metabolism | 30 | 1 | 0.69931 | 0.35767 | 1 | 1 | 0.08725 | D-Maltose cpd:C00208 | 1,4-beta-D-Glucan cpd:C00760; Cellobiose cpd:C00185; beta-D-Fructose cpd:C02336; Sucrose-6-phosphate cpd:C16688; Sucrose cpd:C00089; D-Glucoside cpd:C01798; 1,3-beta-D-Glucan cpd:C00965; UDP-D-Xylose cpd:C00190; Uridine diphosphate glucose cpd:C00029; Uridine diphosphate glucuronic acid cpd:C00167; Glucose 1-phosphate cpd:C00103; Pectin cpd:C00714; Alpha-D-Glucose cpd:C00267; Glucose 6-phosphate cpd:C00668; Amylose cpd:C00718; Trehalose 6-phosphate cpd:C00689; Trehalose cpd:C01083; Starch cpd:C00369; D-Maltose cpd:C00208; Pectic acid cpd:C00470; D-Glucose cpd:C00031; ADP-glucose cpd:C00498; Beta-D-Glucose cpd:C00221; Beta-D-Fructose 6-phosphate cpd:C05345; 1,4-beta-D-Xylan cpd:C02352; Sucrose 6-phosphate cpd:C02591; UDP-D-galacturonate cpd:C00617; Maltodextrin cpd:C01935; Dextrin cpd:C00721; D-Galacturonate cpd:C00333 |
| Glycine, serine and threonine metabolism | 30 | 1 | 0.69931 | 0.35767 | 1 | 1 | 0.13697 | L-Threonine cpd:C00188 | L-Serine cpd:C00065; Choline cpd:C00114; Betaine aldehyde cpd:C00576; 3-Phospho-D-glycerate cpd:C00197; Glycine cpd:C00037; L-Aspartic acid cpd:C00049; Phosphoserine cpd:C01005; 5,10-Methylene-THF cpd:C00143; L-Threonine cpd:C00188; O-Phosphohomoserine cpd:C01102; L-Aspartyl-4-phosphate cpd:C03082; L-Homoserine cpd:C00263; Lipoylprotein cpd:C02051; Aminoacetone cpd:C01888; Tetrahydrofolic acid cpd:C00101; S-Aminomethyldihydrolipoylprotein cpd:C01242; Dihydrolipoylprotein cpd:C02972; D-Serine cpd:C00740; Betaine cpd:C00719; Hydroxypyruvic acid cpd:C00168; Phosphohydroxypyruvic acid cpd:C03232; L-Allothreonine cpd:C05519; 2-Ketobutyric acid cpd:C00109; L-Aspartate-semialdehyde cpd:C00441; Carbon dioxide cpd:C00011; Pyruvaldehyde cpd:C00546; Ammonia cpd:C00014; Pyruvic acid cpd:C00022; L-Tryptophan cpd:C00078; Glyoxylic acid cpd:C00048 |
| Biosynthesis of unsaturated fatty acids | 42 | 1 | 0.81558 | 0.20386 | 1 | 1 | 0 | (4Z,7Z,10Z,13Z,16Z,19Z)-Docosahexaenoic acid cpd:C06429 | (13Z,16Z)-Docosadi-13,16-enoyl-CoA cpd:C16645; Tetracosenoyl-CoA cpd:C16532; Docosenoyl-CoA cpd:C16531; Icosenoyl-CoA cpd:C16530; Tetracosanoyl-CoA cpd:C16529; Docosanoyl-CoA cpd:C16528; (7Z,10Z,13Z,16Z)-Docosatetraenoyl-CoA cpd:C16170; (11Z,14Z)-Icosadienoyl-CoA cpd:C16180; (7Z,10Z,13Z,16Z,19Z)-Docosapentaenoyl-CoA cpd:C16166; (11Z,14Z,17Z)-Icosatrienoyl-CoA cpd:C16179; Palmityl-CoA cpd:C00154; Stearoyl-CoA cpd:C00412; Eicosanoyl-CoA cpd:C02041; Oleoyl-CoA cpd:C00510; Linoleoyl-CoA cpd:C02050; Arachidonyl-CoA cpd:C02249; 8,11,14-Eicosatrienoyl-CoA cpd:C03595; Gamma-linolenoyl-CoA cpd:C03035; (4Z,7Z,10Z,13Z,16Z,19Z)-Docosahexaenoyl-CoA cpd:C16169; (5Z,8Z,11Z,14Z,17Z)-Icosapentaenoyl-CoA cpd:C16165; Alpha-Linolenoyl-CoA cpd:C16162; 13,16-Docosadienoic acid cpd:C16533; Nervonic acid cpd:C08323; Erucic acid cpd:C08316; Icosenoic acid cpd:C16526; Tetracosanoic acid cpd:C08320; Behenic acid cpd:C08281; 7,10,13,16-Docosatetraenoic acid cpd:C16527; Icosadienoic acid cpd:C16525; Clupanodonic acid cpd:C16513; Icosatrienoic acid cpd:C16522; Palmitic acid cpd:C00249; Stearic acid cpd:C01530; Arachidic acid cpd:C06425; Oleic acid cpd:C00712; Linoleic acid cpd:C01595; Arachidonic acid cpd:C00219; 8,11,14-Eicosatrienoic acid cpd:C03242; Gamma-Linolenic acid cpd:C06426; (4Z,7Z,10Z,13Z,16Z,19Z)-Docosahexaenoic acid cpd:C06429; Eicosapentaenoic acid cpd:C06428; Alpha-Linolenic acid cpd:C06427 |

Table S3 Differentially expressed Metabolites between *PmACRE1*-OX and the vector control line

| **id** | **compound name** | **CAS** | **KEGG_ID** | **FORMULA** | **EXACT_MASS** | **CLASS_EN** | **Q1** | **Q3** | **ionmode** | **rt** | **MEAN PmACRE_OX** | **MEAN vector_CT** | **VIP** | **P-VALUE** | **Q-VALUE** | **FOLD CHANGE** | **LOG_FOLDCHANGE** |
| --- | --- | --- | --- | --- | --- | --- | --- | --- | --- | --- | --- | --- | --- | --- | --- | --- | --- |
| 1 | (-)-Anonaine | 1862-41-5 | C09339 | C17H15NO2 | 265.11027899999999 | Alkaloids | 266.1 | 192.1 | + | 7.4233 | 3.84784E-05 | 9.11919E-05 | 1.722711994 | 0.049332231 | 0.418784479 | 0.421950275 | -1.244855101 |
| 2 | (-)-Borneol;(+)-Borneol | 464-45-9;464-43-7 | C01766;C01765 | C10H18O | 154.13576499999999 | Monoterpenoids | 155.1 | 137.1 | + | 3.423966667 | 0.001882151 | 0.00321014 | 1.650689236 | 0.045023545 | 0.408532946 | 0.586314272 | -0.770253919 |
| 17 | (±)-Jasmonic acid | 77026-92-7 | C08491 | C12H18O3 | 210.12559999999999 | phytohormone | 209 | 59 | - | 8.94 | 0.008709892 | 0.001986303 | 1.781867479 | 0.029922324 | 0.360518321 | 4.384976437 | 2.13256909 |
| 63 | 1beta-Hydroxyalantolactone | 68776-47-6 |  | C15H20O3 | 248.141245 | Sesquiterpenoids | 249.1 | 231.1 | + | 5.25755 | 0.000225849 | 0.000121461 | 1.739774339 | 0.025575454 | 0.340901248 | 1.85943948 | 0.894867792 |
| 77 | 2-Hydroxyethanesulfonate | 107-36-8 | C05123 | C2H6O4S | 125.998681 | Organic acids | 127 | 109 | + | 0.755483333 | 0.000547431 | 0.000862126 | 1.730580221 | 0.025626281 | 0.341154692 | 0.634978455 | -0.655220452 |
| 84 | 2,3-Dihydro-2-phenyl-4H-benzopyran-4-one | 487-26-3 | C02099 | C15H12O2 | 224.08373 | Flavonoids | 225.1 | 121 | + | 4.928733333 | 0.003004257 | 0.004909142 | 1.860673247 | 0.01027995 | 0.245173842 | 0.611971872 | -0.708462751 |
| 85 | 2,5-Dihydroxybenzaldehyde | 1194-98-5 | C05585 | C7H6O3 | 138.03169500000001 | Phenols | 139 | 65 | + | 5.085466667 | 0.001047153 | 0.001916297 | 1.820872993 | 0.007264587 | 0.220971204 | 0.546445889 | -0.871849452 |
| 87 | 2,6-Dimethylaniline | 87-62-7 | C11004 | C8H11N | 121.08914900000001 | Benzene and substituted derivatives | 122.1 | 105.1 | + | 2.01845 | 0.00011946 | 0.000246041 | 1.85013414 | 0.002157457 | 0.13182968 | 0.48552888 | -1.042370983 |
| 118 | 3-Nitro-L-tyrosine | 621-44-3 |  | C9H10N2O5 | 226.05897300000001 | Miscellaneous | 227.1 | 181.1 | + | 8.72735 | 0.000127519 | 6.0262E-05 | 1.707443188 | 0.019758911 | 0.307045649 | 2.116074306 | 1.081390288 |
| 124 | 3,4-Dihydrocoumarin | 119-84-6 | C02274 | C9H8O2 | 148.05242999999999 | Coumarins | 149.1 | 105.1 | + | 6.679833333 | 0.003407558 | 0.005228676 | 1.777726322 | 0.018180376 | 0.295829267 | 0.651705656 | -0.61770758 |
| 132 | 4-Aminobutyric acid | 56-12-2 | C00334 | C4H9NO2 | 103.0633 | amino acids | 104.1 | 87.1 | + | 0.66 | 5.99995E-05 | 0.000275991 | 1.92564824 | 0.004528618 | 0.187175684 | 0.217396448 | -2.201599727 |
| 175 | 5'-Deoxyadenosine | 4754-39-6 | C05198 | C10H13N5O3 | 251.10184000000001 | Nucleotide and its derivates | 252.1 | 136.1 | + | 2.408591667 | 0.007333791 | 0.010553442 | 1.84728051 | 0.005232826 | 0.197319193 | 0.694919396 | -0.525082445 |
| 178 | 6-Aminocaproic acid | 60-32-2 | C02378 | C6H13NO2 | 131.094629 | Fatty Acyls | 132.1 | 41 | + | 1.8374 | 0.02585467 | 0.017150722 | 1.706636998 | 0.015870426 | 0.279101324 | 1.507497415 | 0.592155529 |
| 190 | 7-Ethoxycoumarin | 31005-02-4 | C11052 | C11H10O3 | 190.062995 | Coumarins | 191.1 | 163 | + | 10.5655 | 2.02235E-05 | 0.000142121 | 1.528541254 | 0.00907056 | 0.236841103 | 0.142297493 | -2.813017853 |
| 207 | 9(S)-HPOT | 111004-08-1 | C16321 | C18H30O4 | 310.21440999999999 | Fatty acyls | 309.2 | 121.1 | - | 11.13306667 | 0.000912507 | 0.000495119 | 1.83641372 | 0.004809442 | 0.191445799 | 1.843003399 | 0.882058732 |
| 214 | Acetovanillone | 498-02-2 | C11380 | C9H10O3 | 166.062995 | Phenols | 167.1 | 43 | + | 5.236516667 | 0.000522189 | 0.000837346 | 1.676144015 | 0.032388991 | 0.370060287 | 0.623623217 | -0.681253455 |
| 219 | Aconitine | 302-27-2 | C06091 | C34H47NO11 | 645.31491400000004 | Alkaloids | 646.3 | 586.3 | + | 12.06715 | 0.003124485 | 0.013513217 | 1.965997789 | 0.007737824 | 0.225614944 | 0.231216964 | -2.112680846 |
| 227 | Agomelatine | 138112-76-2 |  | C15H17NO2 | 243.12592900000001 | Miscellaneous | 244.1 | 185.1 | + | 10.00285 | 6.28938E-05 | 2.38802E-05 | 1.717170263 | 0.005160402 | 0.196358785 | 2.633720548 | 1.397102276 |
| 245 | Alpha-Terpineol;Patulin | 10482-56-1;149-29-1 | C11393;C16748 | C10H18O;C7H6O4 | 154.13576499999999;154.02661000000001 | Monoterpenoids;Pyrans | 155.05 | 137.05 | + | 3.456525 | 0.001882151 | 0.00321014 | 1.650689236 | 0.045023545 | 0.408532946 | 0.586314272 | -0.770253919 |
| 266 | Arecaidine | 499-04-7 | C10128 | C7H11NO2 | 141.078979 | Alkaloids | 142.1 | 96.1 | + | 1.544283333 | 0.002263608 | 0.001508942 | 1.813358462 | 0.006087621 | 0.207471786 | 1.500129526 | 0.585087073 |
| 273 | Asp-Phe methyl ester;Aspartame | 22839-47-0 | C11045 | C14H18N2O5 | 294.12157300000001 | Carboxylic acids and derivatives | 295.1 | 120.1 | + | 3.05875 | 0.009016073 | 0.007514944 | 1.90681947 | 0.001857395 | 0.120808083 | 1.199752653 | 0.262737003 |
| 275 | Astragalin | 480-10-4 | C12249 | C21H20O11 | 448.10059999999999 | flavonoids | 449 | 286.9 | + | 6.52 | 0.008013696 | 0.015146335 | 1.82983284 | 0.012309049 | 0.256323608 | 0.529084823 | -0.918429062 |
| 317 | Betulin | 473-98-3 | C08618 | C30H50O2 | 442.38108 | Triterpenoids | 425.4 | 95.1 | + | 12.3169 | 0.000707374 | 0.000408297 | 1.635768325 | 0.027137015 | 0.348410797 | 1.732500097 | 0.792855433 |
| 354 | Carnosol | 5957-80-2 | C09069 | C20H26O4 | 330.18311 | Diterpenoids | 329.2 | 285.2 | - | 12.14696707 | 1.04865E-05 | 3.24526E-05 | 1.346516172 | 0.043034781 | 0.403309156 | 0.32313201 | -1.629804421 |
| 357 | Cassythicine | 5890-28-8 | C09389 | C19H19NO4 | 325.13140900000002 | Alkaloids | 326.1 | 252.1 | + | 9.903166667 | 4.31691E-05 | 2.00152E-05 | 1.587289962 | 0.028354249 | 0.353892011 | 2.156820421 | 1.108906061 |
| 382 | cis-Aconitic acid | 499-12-7;585-84-2 | C00417 | C6H6O6 | 174.01643999999999 | Organic acids and derivatives | 175 | 69 | + | 1.4555 | 0.007991525 | 0.003473543 | 1.544286183 | 0.029021985 | 0.356770999 | 2.300684358 | 1.202063067 |
| 393 | Coproporphyrin III | 14643-66-4 | C05770 | C36H38N4O8 | 654.26896599999998 | Tetrapyrroles and derivatives | 655.3 | 619.3 | + | 11.84896667 | 0.001876521 | 0.00312124 | 1.661930635 | 0.048204512 | 0.416231165 | 0.601210249 | -0.73405849 |
| 394 | Cordycepin | 73-03-0 | C08431 | C10H13N5O3 | 251.10184000000001 | Nucleotide and its derivates | 252.1 | 136.1 | + | 2.34785 | 0.007333791 | 0.010553442 | 1.84728051 | 0.005232826 | 0.197319193 | 0.694919396 | -0.525082445 |
| 399 | Crotaline | 315-22-0 | C10350 | C16H23NO6 | 325.15253899999999 | Alkaloids | 326.2 | 120.1 | + | 11.42833333 | 0.003322206 | 1.36576E-06 | 1.978234297 | 0.002292581 | 0.136368724 | 2432.490719 | 11.24821859 |
| 409 | Cyanidin | 528-58-5 | C05905 | C15H10O6 | 286.04773999999998 | Flavonoids | 287.1 | 137 | + | 5.898142 | 0.022982557 | 0.031367467 | 1.750706719 | 0.026970668 | 0.347637229 | 0.732687691 | -0.448729716 |
| 423 | D-Glucose 6-phosphate;Glucose 6-phosphate | 56-73-5 | C00092 | C6H13O9P | 260.02972199999999 | Carbohydrates | 259 | 97 | - | 0.6463 | 0.000114703 | 0.000378951 | 1.666864857 | 0.049056251 | 0.418167616 | 0.30268547 | -1.724108673 |
| 425 | D-Maltose | 69-79-4 | C00208 | C12H22O11 | 342.11621500000001 | Carbohydrates | 341.1 | 59 | - | 0.764066667 | 0.000539267 | 0.001514202 | 1.724310845 | 0.028631838 | 0.355099478 | 0.356139703 | -1.489484815 |
| 431 | D-Xylulose | 551-84-8 | C00310 | C5H10O5 | 150.05282500000001 | Carbohydrates | 133.1 | 43 | + | 1.76245 | 0.00192189 | 0.001296804 | 1.754516476 | 0.013282287 | 0.260725185 | 1.48202046 | 0.567565365 |
| 440 | Decursinol | 23458-02-8 | C09259 | C14H14O4 | 246.08921000000001 | Coumarins | 247.1 | 175 | + | 7.9889 | 0.000631921 | 0.000204478 | 1.730440017 | 0.00715291 | 0.219816206 | 3.090403323 | 1.627795134 |
| 451 | Denudatine | 26166-37-0 | C08680 | C22H33NO2 | 343.25112899999999 | Alkaloids | 344.3 | 326.2 | + | 1.587366667 | 0.005064198 | 0.007852099 | 1.77010188 | 0.012199065 | 0.25579227 | 0.644948343 | -0.632744482 |
| 462 | Diallyl disulfide | 2179-57-9 | C08369 | C6H10S2 | 146.022392 | Miscellaneous | 147 | 41 | + | 0.678083333 | 0.003456326 | 0.008112794 | 1.94758034 | 0.014410576 | 0.269177235 | 0.426034003 | -1.230959514 |
| 473 | Diosmetin | 520-34-3 | C10038 | C16H12O6 | 300.0634 | flavonoids | 301.3 | 285.8 | + | 9.15 | 0.000155423 | 6.69216E-05 | 1.538641194 | 0.048699418 | 0.417362408 | 2.322458252 | 1.215652663 |
| 481 | Docosahexaenoic acid | 6217-54-5 | C06429 | C22H32O2 | 328.24023 | Fatty Acyls | 329.2 | 93.1 | + | 12.60253333 | 0.000346937 | 0.00073828 | 1.783230787 | 0.027783169 | 0.351358986 | 0.469925288 | -1.089496691 |
| 506 | Ethyl 3,4,5-trimethoxybenzoate | 6178-44-5 |  | C12H16O5 | 240.09977499999999 | Phenols | 241.1 | 195.1 | + | 0.529283333 | 6.23108E-05 | 9.98038E-05 | 1.578164082 | 0.033405863 | 0.373712677 | 0.624332748 | -0.679612954 |
| 507 | Ethyl acrylate | 140-88-5 | C19238 | C5H8O2 | 100.05243 | Carboxylic acids and derivatives | 101.1 | 55 | + | 1.73615 | 0.001670223 | 0.003528403 | 1.899466838 | 0.00580831 | 0.204376146 | 0.47336516 | -1.078974569 |
| 520 | Ferulic acid; Trans-Ferulic acid;trans-Ferulic acid | 1135-24-6;537-98-4 | C01494 | C10H10O4 | 194.05790999999999 | Phenylpropanoids | 195.1 | 117 | + | 4.85895 | 0.000617821 | 0.001189792 | 1.743101914 | 0.040271095 | 0.395453083 | 0.519267981 | -0.945448827 |
| 529 | Fucoxanthin | 3351-86-8 | C08596 | C42H58O6 | 658.42334000000005 | Miscellaneous | 659.4 | 109.1 | + | 12.49073333 | 0.000320603 | 0.000998617 | 1.913454875 | 0.008137201 | 0.229244648 | 0.321047218 | -1.639142598 |
| 532 | Furfuryl acetate | 623-17-6 |  | C7H8O3 | 140.04734500000001 | Miscellaneous | 141.1 | 81 | + | 5.471466667 | 0.000413008 | 0.000208053 | 1.834045056 | 0.012396916 | 0.256742872 | 1.985111009 | 0.989219686 |
| 538 | Galanthaminone | 510-77-0 | C08534 | C17H19NO3 | 285.13649400000003 | Alkaloids | 286.1 | 242.1 | + | 6.9108 | 9.4469E-06 | 4.85201E-05 | 1.552667752 | 0.010310862 | 0.245367955 | 0.194700559 | -2.360671068 |
| 541 | gamma-Hydroxy-3-pyridinebutanoate | 15569-97-8 | C19579 | C9H11NO3 | 181.073894 | Pyridines and derivatives | 182.1 | 164.1 | + | 4.3638 | 6.16977E-05 | 0.000379915 | 1.86950528 | 0.000678284 | 0.059075587 | 0.162398693 | -2.622388075 |
| 599 | Guaiacol | 90-05-1 | C15572;C01502 | C7H8O2 | 124.05243 | Phenols | 125.1 | 65 | + | 5.622516667 | 0.000261742 | 0.000397477 | 1.698533004 | 0.030480508 | 0.362767563 | 0.658507854 | -0.602727448 |
| 603 | Guanosine 3',5'-cyclic monophosphate | 7665-99-8 | C00942 | C10H12N5O7P | 345.047437 | Nucleotide and its derivates | 346.1 | 152.1 | + | 2.25995 | 0.001251657 | 0.000875668 | 1.771698897 | 0.016669581 | 0.284057394 | 1.429374784 | 0.515384242 |
| 611 | Hesperidin | 520-26-3 | C09755 | C28H34O15 | 610.18979999999999 | flavonoids | 609 | 300.8 | - | 6.65 | 0.005205718 | 9.08664E-05 | 1.911229276 | 4.77322E-05 | 0.009456029 | 57.28981864 | 5.840206866 |
| 612 | Hinokiflavone | 19202-36-9 | C10057 | C30H18O10 | 538.09 | Flavonoids | 539.1 | 521.1 | + | 11.93288333 | 0.001584605 | 0.000933878 | 1.741432124 | 0.036655898 | 0.38442773 | 1.696801925 | 0.762818162 |
| 613 | Homobaldrinal | 67910-07-0 | C16812 | C15H16O4 | 260.10485999999997 | Miscellaneous | 261.1 | 159 | + | 10.00285 | 0.003494151 | 0.00017172 | 1.96058468 | 2.93667E-05 | 0.009456029 | 20.34798814 | 4.346814254 |
| 615 | Homoeriodictyol | 446-71-9 | C09756 | C16H14O6 | 302.07904000000002 | Flavonoids | 303.1 | 153 | + | 6.830883333 | 0.016976692 | 0.000253538 | 1.970679967 | 0.015567115 | 0.277136157 | 66.95927539 | 6.065212011 |
| 619 | Hordenine | 539-15-1 | C06199 | C10H15NO | 165.115364 | Alkaloids | 166.1 | 121.1 | + | 3.03795 | 7.89926E-05 | 0.000169611 | 1.778372964 | 0.020539504 | 0.312227274 | 0.465726943 | -1.102443748 |
| 637 | Isobergapten | 482-48-4 | C18082 | C12H8O4 | 216.04226 | Coumarins | 217 | 202 | + | 9.937166667 | 0.004287798 | 0.000248691 | 1.930446008 | 0.000315936 | 0.038909437 | 17.24143687 | 4.107808106 |
| 649 | Isotetrandrine | 477-57-6 | C17060 | C38H42N2O6 | 622.30428800000004 | Alkaloids | 623.3 | 578.3 | + | 12.18463333 | 3.76923E-05 | 0.000158848 | 1.305465305 | 0.013310865 | 0.260846812 | 0.237285573 | -2.075303709 |
| 663 | Kirenol | 52659-56-0 |  | C20H34O4 | 338.24570999999997 | Diterpenoids | 321.2 | 81.1 | + | 13.15803333 | 0.008918031 | 0.036718478 | 1.598863866 | 0.036865438 | 0.385072761 | 0.242875846 | -2.041709073 |
| 672 | L-Asparagine | 70-47-3 | C00152 | C4H8N2O3 | 132.053493;132.05350000000001 | Amino acid and derivatives;amino acids | 133.1 | 74 | + | 0.64 | 0.000302663 | 0.000560983 | 1.750629611 | 0.035424171 | 0.380528371 | 0.539522163 | -0.89024587 |
| 681 | L-Isoleucine;L-Leucine | 73-32-5;61-90-5 | C00407;C00123 | C6H13NO2 | 131.094629 | Amino acid and derivatives | 132.1 | 86.1 | + | 1.779341667 | 0.008483232 | 0.004681103 | 1.598838282 | 0.038225566 | 0.389136932 | 1.812229523 | 0.857765687 |
| 694 | L-Threonine | 72-19-5 | C00188 | C4H9NO3 | 119.058244;119.0582 | Amino acid and derivatives;amino acids | 120.1 | 56.1 | + | 0.64 | 0.022223176 | 0.010561815 | 1.816383087 | 0.00521757 | 0.197118324 | 2.104105846 | 1.073207281 |
| 699 | Lactulose | 4618-18-2 | C07064 | C12H22O11 | 342.11621500000001 | Organooxygen compounds | 365.1 | 203.1 | + | 0.772266667 | 0.005057345 | 0.013982911 | 1.922381718 | 0.035522751 | 0.380847408 | 0.361680386 | -1.467212734 |
| 713 | Ligustilide | 4431-01-0 | C16987 | C12H14O2 | 190.09938 | Miscellaneous | 191.1 | 67.1 | + | 2.803 | 0.00022093 | 0.000278817 | 1.617927912 | 0.034976266 | 0.379063054 | 0.792382065 | -0.335731869 |
| 731 | Lumichrome | 1086-80-2 | C01727 | C12H10N4O2 | 242.080376 | Alkaloids | 243.1 | 198.1 | + | 6.696616667 | 0.00215814 | 0.00148615 | 1.594607063 | 0.043384992 | 0.404253781 | 1.45216861 | 0.538208973 |
| 732 | Lupanine | 550-90-3 | C10772 | C15H24N2O | 248.188863 | Alkaloids | 249.2 | 136.1 | + | 2.958883333 | 0.001957678 | 0.000496007 | 1.94979313 | 0.017796285 | 0.292938324 | 3.946872131 | 1.98070978 |
| 748 | Maltotriose | 1109-28-0 | C01835 | C18H32O16 | 504.16904 | Organooxygen compounds | 527.2 | 365.1 | + | 1.226433333 | 0.001117644 | 0.003584665 | 1.962533305 | 0.000488233 | 0.048524438 | 0.311784758 | -1.681377694 |
| 765 | Methyl benzoate | 93-58-3 | C20645 | C8H8O2 | 136.05242999999999 | Phenols | 137.1 | 105 | + | 10.72676667 | 0.00020464 | 9.53822E-05 | 1.682713964 | 0.049941247 | 0.420127886 | 2.145468841 | 1.101292949 |
| 790 | Mulberrofuran C | 77996-04-4 | C08928 | C34H28O9 | 580.17333499999995 | Phenols | 581.2 | 137 | + | 12.8056 | 0.031031792 | 0.053229968 | 1.645712321 | 0.048562553 | 0.417051261 | 0.582975972 | -0.778491673 |
| 796 | N-((-)-jasmonoyl)-S-isoleucine | 120330-92-9 | C18699 | C18H29NO4 | 323.20965799999999 | phytohormone | 322 | 130 | - | 10.41 | 0.003197981 | 0.000349338 | 1.973749154 | 1.22633E-05 | 0.009456029 | 9.154407818 | 3.194466564 |
| 806 | N-hydroxy tryptamine |  | C17203 | C10H12N2O | 176.09496300000001 | Tryptamine derivatives | 177.1 | 144.1 | + | 2.618383333 | 0.001704777 | 0.005319049 | 1.852191695 | 0.002309603 | 0.136923483 | 0.320504115 | -1.641585215 |
| 819 | Naringenin chalcone | 25515-46-2 | C06561 | C15H12O5 | 272.06847499999998 | Flavonoids | 273.1 | 153 | + | 6.54065 | 0.008736126 | 0.002349985 | 1.730699009 | 0.029634849 | 0.35933813 | 3.717525006 | 1.894342447 |
| 826 | Neohesperidin | 13241-33-3 | C09806 | C28H34O15 | 610.18979999999999 | flavonoids | 609 | 300.8 | - | 6.83 | 0.005205718 | 0.000119861 | 1.894802226 | 4.82536E-05 | 0.009456029 | 43.43111348 | 5.440657036 |
| 830 | NG,NG-Dimethylarginine dihydrochloride | 220805-22-1;30315-93-6 | C03626 | C8H18N4O2 | 202.142976 | Carboxylic acids and derivatives | 203.2 | 70.1 | + | 0.7442 | 0.001450799 | 0.002716227 | 1.858219918 | 0.005904246 | 0.205461815 | 0.53412308 | -0.904755869 |
| 838 | Nobiletin | 478-01-3 | C10112 | C21H22O8 | 402.13146999999998 | Flavonoids | 403.1 | 373.1 | + | 11.03 | 0.033314812 | 0.000712516 | 1.558312648 | 0.008493393 | 0.232281027 | 46.75657086 | 5.547097221 |
| 856 | Oleuroside | 116383-31-4 |  | C25H32O13 | 540.18429500000002 | Iridoids | 541.2 | 137.1 | + | 12.730075 | 0.000638936 | 0.001542589 | 1.665458175 | 0.037609996 | 0.387323435 | 0.41419709 | -1.271610676 |
| 858 | Orobol (5,7,3',4'-tetrahydroxyisoflavone) | 480-23-9 | C10510 | C15H10O6 | 286.04773999999998 | Flavonoids | 287.1 | 269 | + | 7.904983333 | 0.000333981 | 0.000694568 | 1.523109544 | 0.040109111 | 0.394969257 | 0.480847013 | -1.056350138 |
| 891 | Phloretin | 60-82-2 | C00774 | C15H14O5 | 274.08409999999998 | flavonoids | 274.9 | 169 | + | 8.85 | 0.000395087 | 0.000257278 | 1.610575389 | 0.032783341 | 0.371495039 | 1.535638325 | 0.618838472 |
| 892 | Phosphonoacetate | 4408-78-0 | C05682 | C2H5O5P | 139.98746199999999 | Organic acids | 141 | 95 | + | 1.879933333 | 0.00684067 | 0.008950756 | 1.857546087 | 0.00393271 | 0.176934418 | 0.764256093 | -0.387871946 |
| 898 | Physalin G | 76045-38-0 |  | C28H30O10 | 526.18389999999999 | Steroids and steroid derivatives | 527.2 | 509.2 | + | 12.16785 | 0.003830433 | 0.002003332 | 1.765806093 | 0.023551827 | 0.330271984 | 1.912030919 | 0.935105853 |
| 916 | Poncirin | 14941-08-3 | C09830 | C28H34O14 | 594.19485999999995 | Flavonoids | 595.2 | 269.1 | + | 8.156733333 | 0.000113109 | 4.02321E-05 | 1.648648997 | 0.015603599 | 0.277375097 | 2.811423086 | 1.491300579 |
| 960 | Rivularine | 723-78-4 | C10278 | C13H19NO3 | 237.136494 | Alkaloids | 238.1 | 55.1 | + | 4.347016667 | 3.30981E-05 | 7.18249E-05 | 1.841348349 | 0.016220639 | 0.281311754 | 0.460815819 | -1.117737854 |
| 965 | Ruscogenin | 472-11-7 | C08909 | C27H42O4 | 430.30831000000001 | Steroids and steroid derivatives | 431.3 | 269.2 | + | 12.804993 | 0.000116391 | 5.5984E-05 | 1.609314029 | 0.012834226 | 0.258763201 | 2.078996619 | 1.055887412 |
| 967 | Rutaevin | 33237-37-5 | C08779 | C26H30O9 | 486.188985 | Triterpenoids | 487.2 | 469.2 | + | 12.5814 | 0.002814598 | 0.004304451 | 1.474173733 | 0.04344663 | 0.404418915 | 0.653880977 | -0.612900042 |
| 1006 | Sinapyl alcohol | 537-33-7 | C02325 | C11H14O4 | 210.08921000000001 | Hydroxycinnamoyl derivatives | 193.1 | 161.1 | + | 4.430933333 | 0.000631334 | 0.001177911 | 1.807015967 | 0.011077896 | 0.249924163 | 0.535977311 | -0.899756165 |
| 1008 | Sinigrin | 3952-98-5 | C08427 | C10H17NO9S2 | 359.03447599999998 | Organooxygen compounds | 358 | 97 | - | 5.092183333 | 4.44332E-05 | 9.26433E-05 | 1.6962993 | 0.037775798 | 0.387816033 | 0.479616051 | -1.060048154 |
| 1018 | Streptozotocin | 18883-66-4 | C07313 | C8H15N3O7 | 265.091002 | Organooxygen compounds | 266.1 | 140.1 | + | 0.71665 | 0.002628335 | 0.00491739 | 1.781456761 | 0.01019822 | 0.244656457 | 0.534497947 | -0.903743688 |
| 1021 | Sucrose | 57-50-1 | C00089 | C12H22O11 | 342.11621500000001 | Carbohydrates | 365.1 | 203.1 | + | 0.8918 | 0.005057345 | 0.013982911 | 1.922381718 | 0.035522751 | 0.380847408 | 0.361680386 | -1.467212734 |
| 1037 | Taurohyocholate |  | C15516 | C26H45NO7S | 515.29167500000005 |  | 516.3 | 109 | + | 11.4294 | 0.001931337 | 0.001582425 | 1.738246041 | 0.02400493 | 0.332746999 | 1.220491865 | 0.287462679 |
| 1059 | trans-Piceid | 27208-80-6 | C10275 | C21H24O8 | 404.14711999999997 | Stilbenes | 403.1 | 45 | - | 6.238010795 | 0.000112762 | 0.000310756 | 1.540516098 | 0.03285996 | 0.371771078 | 0.362863103 | -1.462502726 |
| 1071 | Turanose | 547-25-1 | C19636 | C12H22O11 | 342.11621500000001 | Fatty Acyls | 365.1 | 203.1 | + | 0.78905 | 0.005057345 | 0.013982911 | 1.922381718 | 0.035522751 | 0.380847408 | 0.361680386 | -1.467212734 |
| 1087 | Veraguensin | 19950-55-1 | C10892 | C22H28O5 | 372.19367499999998 | Lignans | 373.2 | 137.1 | + | 6.746966667 | 0.000494825 | 0.001672834 | 1.862517587 | 0.010456757 | 0.246272638 | 0.295800541 | -1.757303404 |
| 1088 | Vestitol | 35878-41-2 | C16225 | C16H16O4 | 272.10485999999997 | Flavonoids | 273.1 | 123 | + | 6.512016667 | 0.000223588 | 8.04204E-05 | 1.963317893 | 0.000306355 | 0.038238017 | 2.780237004 | 1.475207872 |
| 1104 | Xanthotoxol | 2009-24-7 | C00841 | C11H6O4 | 202.02661000000001 | Phenylpropanoids | 203 | 175 | + | 8.742883333 | 0.000696673 | 0.00346976 | 1.936097235 | 0.000501036 | 0.049092881 | 0.200784145 | -2.316282742 |
|  |  |  |  |  |  |  |  |  |  |  |  |  |  |  |  |  |  |


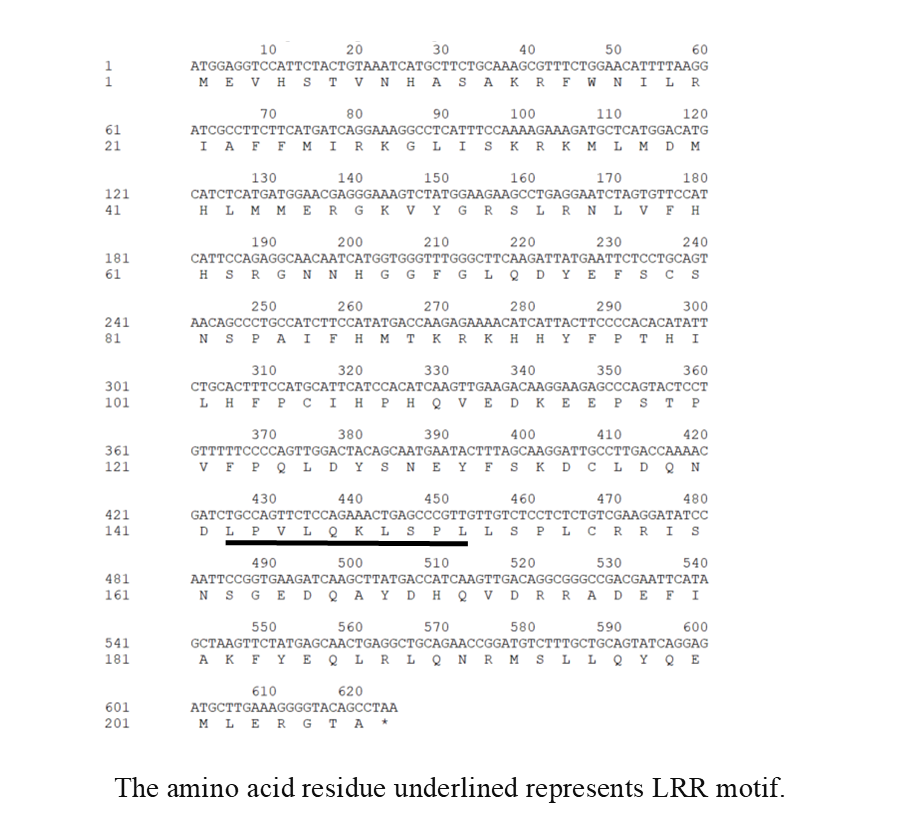


**Fig. S1** The coding sequence of *PmACRE1*


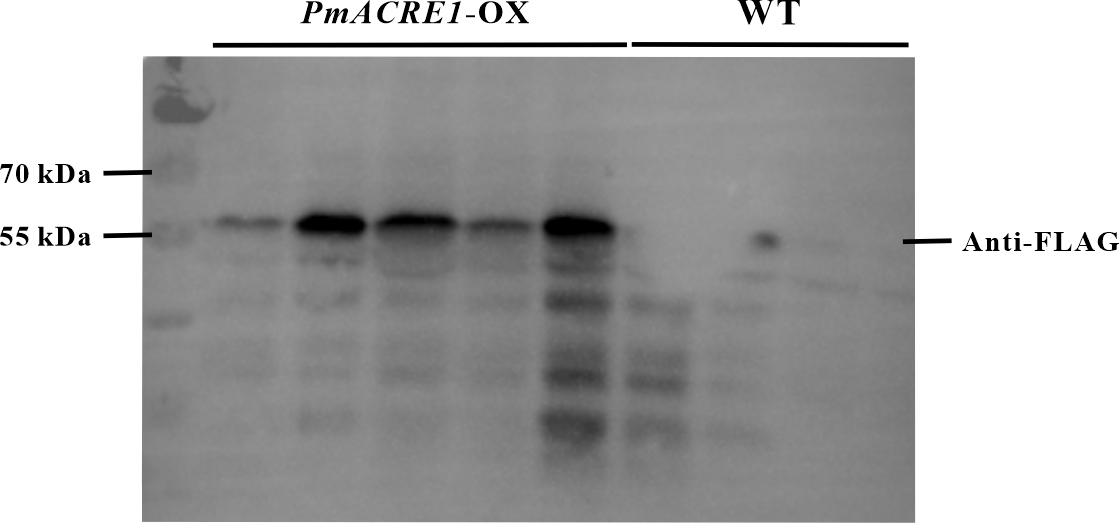


**Fig. S2** PmACRE1 protein expression in the transgenic *Arabidopsis thaliana*


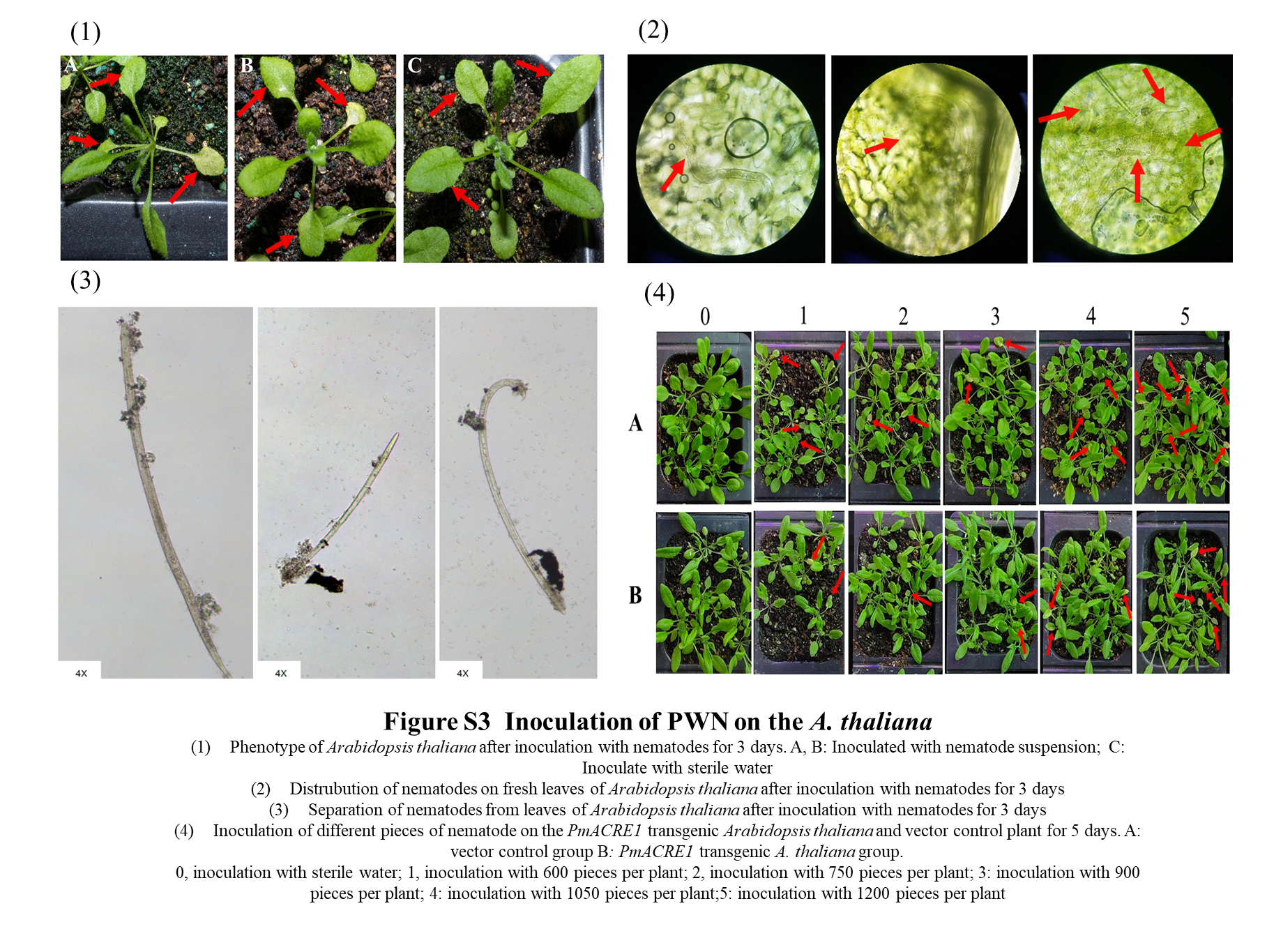


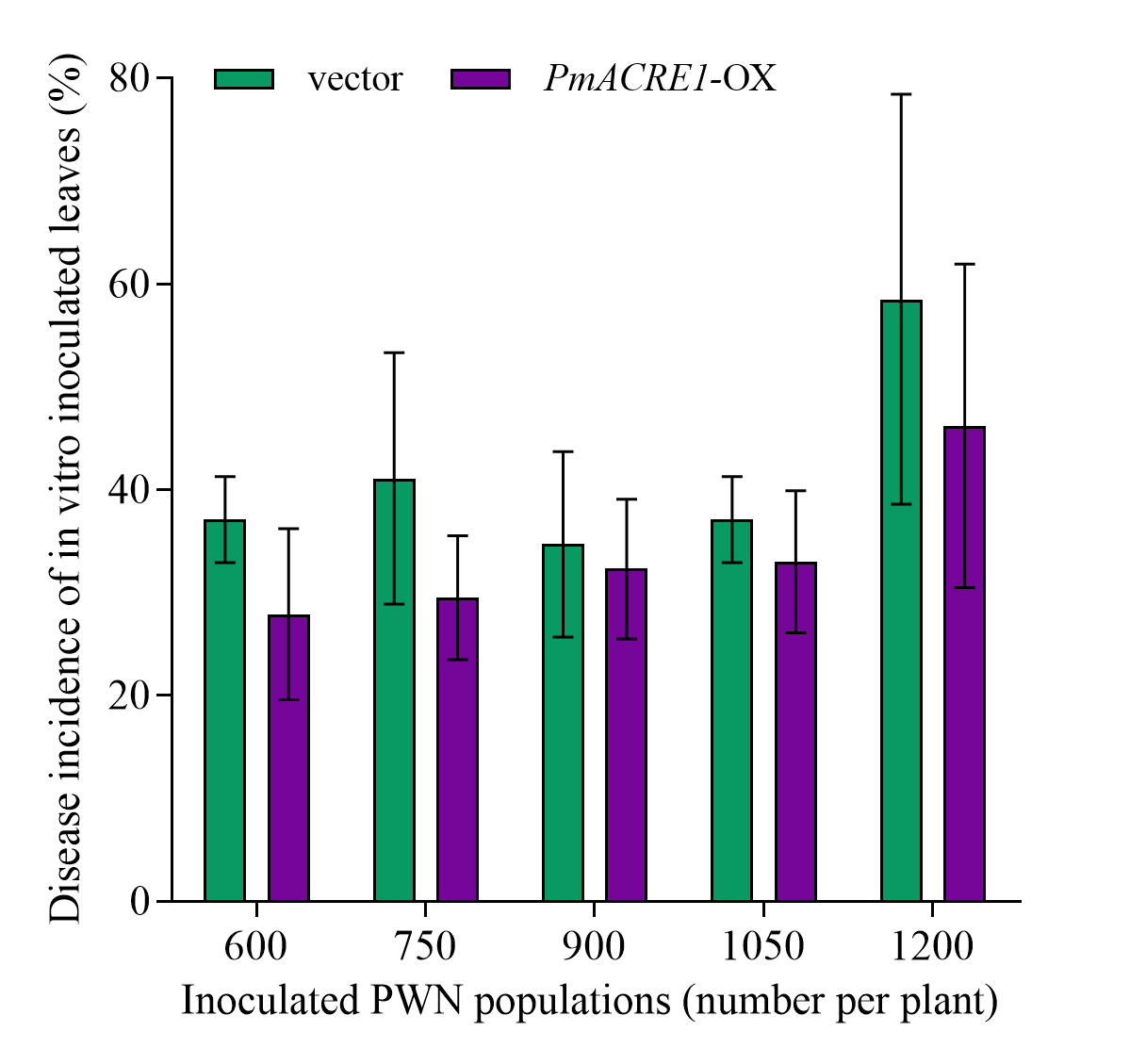


**Fig. S4** The incidence of *Arabidopsis thaliana* inoculated with different population of pine wood nematodes


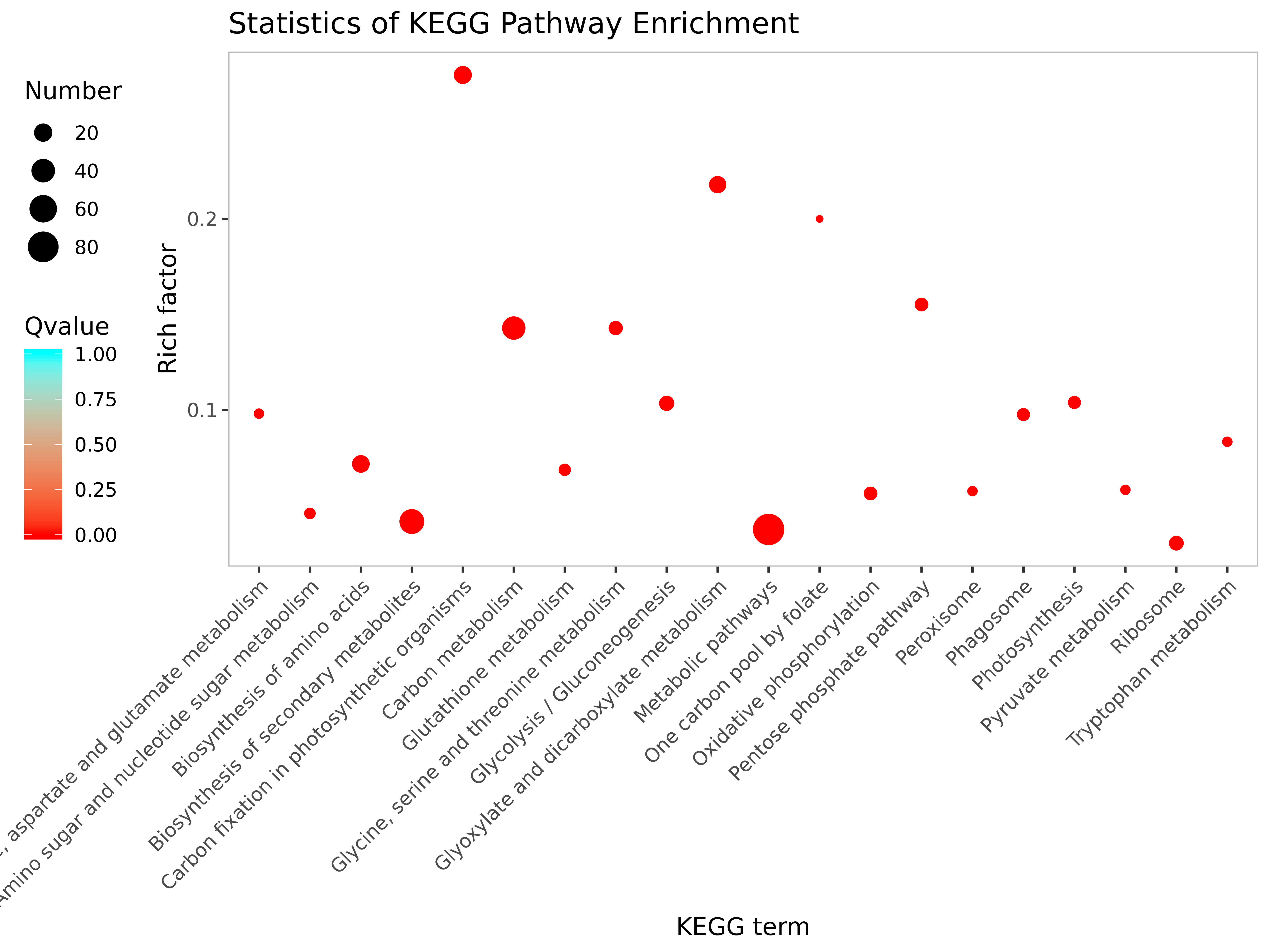


**Fig. S5** KEGG enrichment of the interacted proteins of ACRE1
